# Mechanistic Exploration of Half-Sandwich Iridium(III) Anticancer Compounds through Integrated Cellular, Proteomic, and In Vivo Analyses

**DOI:** 10.1021/acs.jmedchem.5c03448

**Published:** 2026-06-16

**Authors:** Pavel Štarha, Jaroslava Friedecká, Renata Héžová, Ondřej Bárta, Rea Jarošová, Josef Mašek, Ivan Nemec, David Milde, Adam Novobilský, Ladislav Novotný, Jaroslav Ondruš, Slavomíra Šterbinská, Nicol Straková, René Lenobel, Jan Hošek

**Affiliations:** † Department of Inorganic Chemistry, Faculty of Science, Palacký University Olomouc, 17. listopadu 12, 77146 Olomouc, Czech Republic; ‡ Laboratory of Growth Regulators, Institute of Experimental Botany of the Czech Academy of Sciences, and Faculty of Science, Palacký University, Šlechtitelů 27, 783 71 Olomouc, Czech Republic; § Department of Pharmacology and Toxicology, 48357 Veterinary Research Institute, Hudcova 296/70, 62100 Brno, Czech Republic; ∥ Department of Analytical Chemistry, Faculty of Science, Palacký University Olomouc, 17. listopadu 12, 77146 Olomouc, Czech Republic; ⊥ Department of Veterinary Sciences, Faculty of Agrobiology, Food and Natural Resources, Czech University of Life Sciences, Kamýcká 129, 16500 Prague, Czech Republic; # Department of Pathobiology, School of Veterinary Medicine, St. George’s University, True Blue Campus, P.O. Box 7, Saint George, Grenada, West Indies

## Abstract

New half-sandwich iridium­(III) compounds [Ir­(η^5^-Cp^x^)­Cl­(L1–3)]­PF_6_ (**1**–**6**), combining Cp* or Cp^ph^ with N,P-coordinated phosphinoalkylamines L1–L3, were tested in different cancer cells (2D and 3D cultures), including MOR/CPR cisplatin-resistant lung carcinoma. Best-performing compound **3** outperformed its Cp^ph^ analogue **6** and cisplatin in MOR/CPR cells while sparing noncancerous cells. Multiomics profiling shows a non-DNA-targeted mechanism: rapid integrated stress response with ER stress (DDIT3/CHOP) and oxidative stress (HMOX1, ATF3), nucleolar stress, and primary inhibition of ribosome biogenesis and mitochondrial translation. These changes drive translational shutdown, suppression of oxidative phosphorylation with a glycolytic shift, and G_1_ arrest, alongside endolysosomal remodeling (enhanced vesicular uptake, reduced degradative capacity) that favors intracellular retention. The phenotype is predominantly cytostatic with apoptotic priming. In vivo, **3** suppressed tumor growth and activated apoptosis with low systemic toxicity. Compound **3** thus emerges as a promising prototype Ir­(III) metallodrug that disrupts nucleolar, mitochondrial, and lysosomal homeostasis to overcome resistance.

## Graphical abstract

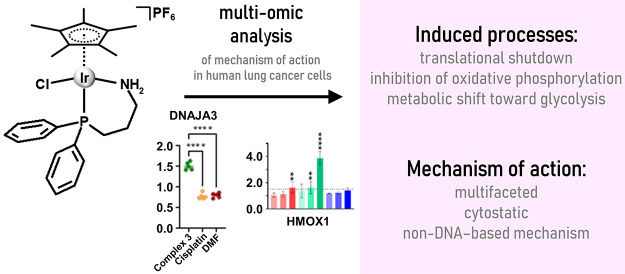

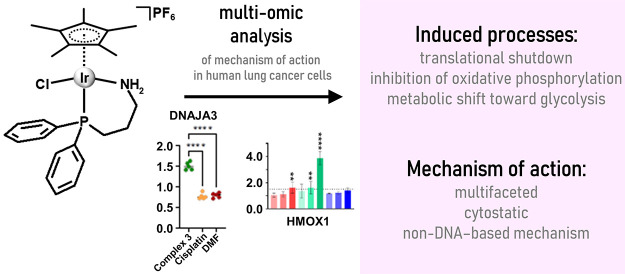

## Introduction

Platinum-based cytostatics represent etalon in cancer treatment. However, they cause many adverse side effects, such as nephrotoxicity and neurotoxicity.^1^ This fact led to the development of new nonplatinum metallodrugs with different mechanisms of action (MoA), as exemplified by nonplatinum anticancer compounds, which have recently entered clinical trials.^2,3^ Half-sandwich compounds derived from the platinum metals Ru, Rh, Os and Ir represent a prominent chemotype.^4–6^ Regarding Ir­(III) cyclopentadienyl (Ir-Cp^x^) compounds,^7^ many representatives have shown high antitumor activity connected with different MoA from cisplatin (CDDP) or other Pt-based drugs.^5,8–10^

Organometallic compounds containing various metals are generally recognized by cells as foreign entities, thereby activating multiple defense pathways. When the induced cellular stress exceeds a critical threshold, it results in cell death - the desired outcome of cytostatic treatment. In contrast, healthy noncancerous cells are typically able to mitigate or recover from such stress. A major component of this defense response is the activation of stress-related genes, leading to elevated synthesis of protective proteins.^11,12^ Interestingly, apart from platinum-based drugs (e.g., refs 13,14 ), relatively few studies have explored how other metallodrugs affect gene expression. Notably, in vitro investigations of half-sandwich Ir­(III) and Os­(II) complexes^15,16^ demonstrated that increased oxidative stress was accompanied by enhanced expression of antioxidant genes, including nuclear factor erythroid 2-related factor 2 (NRF2) and heme oxygenase 1 (HMOX1, a.k.a. HO-1). A deeper understanding of how metallodrugs alter gene expression could provide valuable insight into their MoA.

The MoA of anticancer half-sandwich Ir­(III) cyclopentadienyl (Ir–Cp^x^) compounds is believed to be closely associated with the generation of ROS, leading to oxidative stress-induced cell death.^17,18^ Substantial increases in ROS levels have been consistently observed in various cancer cell lines exposed to Ir–Cp^x^ compounds.^19–21^ The ROS production is attributed to their catalytic ability to mediate intracellular transfer hydrogenation reactions (e.g., utilizing NADH as a hydride source)^22^ or to oxidize key biomolecules such as GSH.^23^ Furthermore, Ir–Cp^x^ compounds preferentially accumulate in lysosomes and mitochondria, where excessive ROS generation causes membrane disruption and ultimately triggers apoptotic pathways.^24,25^

In this study, we synthesized a new series of Ir–Cp^x^ compounds with the general formula [Ir­(η^5^-Cp^x^)­Cl­(L1–3)]­PF_6_ (**1**–**6**; Figures 1 and S1A). These compounds feature two types of cyclopentadienyl ligands (Cp^x^ = Cp* for **1**–**3** or Cp^ph^ for **4**–**6**; HCp* = pentamethylcyclopentadiene, HCp^ph^ = (2,3,4,5-tetramethylcyclopenta-2,4-dien-1-yl)­benzene) combined with three phosphinoalkylamine (aminophosphine) ligands, namely L1 = 2-(di-*tert*-butylphosphanyl)­ethanamine (for **1** and **4**), L2 = 2-(diphenylphosphanyl)­ethanamine (for **2** and **5**) and L3 = 3-(diphenylphosphanyl)­propan-1-amine (for **3** and **6**). For the first time, we investigated the global transcriptional response (*sensu lato*) of stress-related genes in cancer cells treated with Ir–Cp^x^ compounds, whereas earlier studies focused exclusively on genes linked to oxidative stress.^16^ Furthermore, potential off-target effects were systematically evaluated in healthy primary porcine chondrocytes (PPCs) and human peripheral blood mononuclear cells (PBMCs).

**Figure 1:**
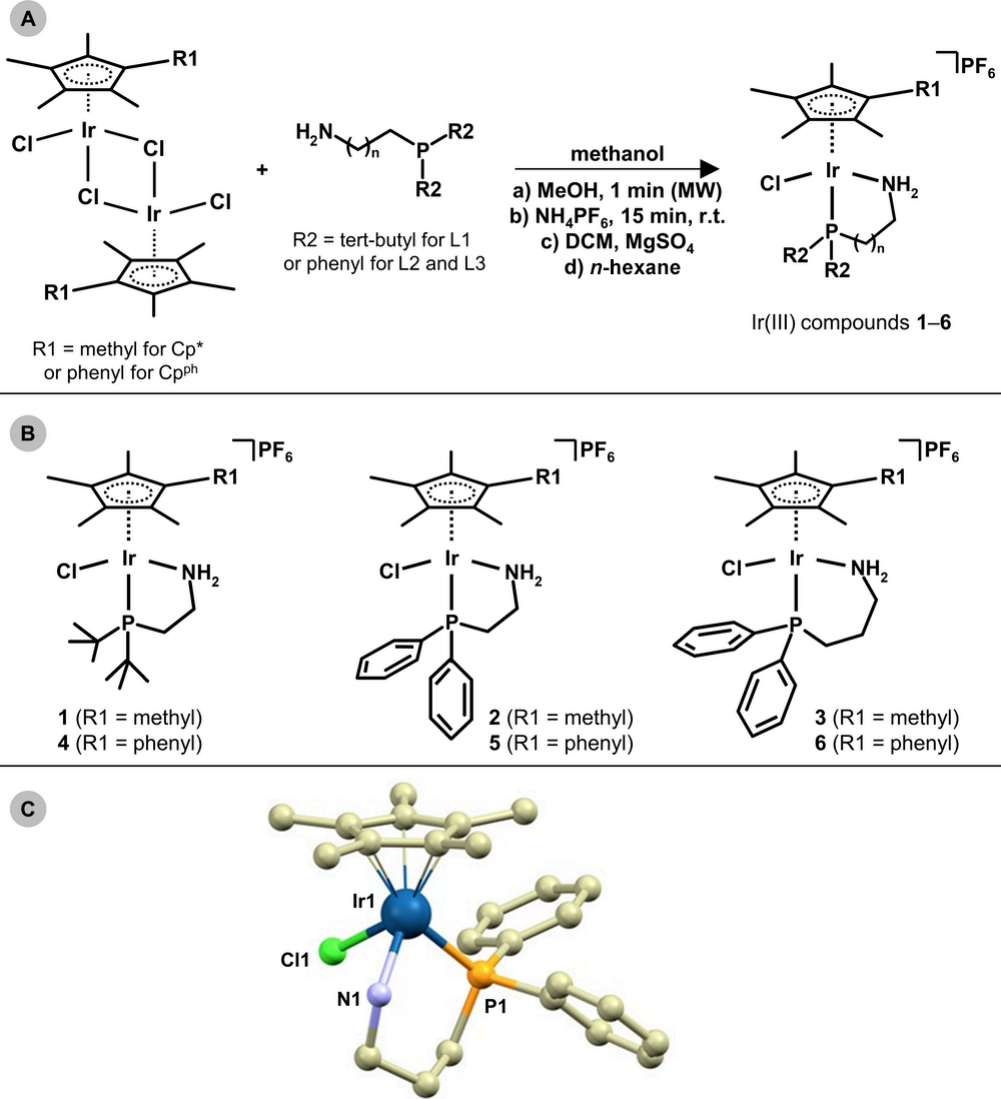
(A) General reaction scheme for the synthesis of compounds **1**–**6**. (B) Structural formulas of the studied Ir­(III) compounds **1**–**6**. (C) Molecular structure of the complex cation in **3** (hydrogen atoms were omitted for clarity).

The most promising metallocomplex **3** was further evaluated using a quantitative label-free proteomic strategy to investigate its effects on the proteome of A549 cells. This approach was designed to capture global alterations in protein expression and to identify key biochemical pathways and molecular targets perturbed by the compound. Through this analysis, we aim to provide insights into the cellular processes underlying its cytotoxic activity and to delineate potential MoA. The results should contribute to a broader understanding of how such metallocomplexes interact with cancer cell proteomes and support their future development as prospective anticancer agents.

## Results and Discussion

### Design and Synthesis

The MoA of anticancer Ir–Cp^x^ compounds is reported to be closely related to their ability to disrupt redox homeostasis in cancer cells, which is linked to transfer hydrogenation reactions catalyzed by these compounds.^5,26,27^ Being aware of this fact, we designed a series of new Ir–Cp^x^ compounds [Ir­(η^5^-Cp^x^)­Cl­(L1–3)]­PF_6_ (**1**–**6**; Figures 1 and S1A) that contain simple, commercially available phosphinoalkylamines L1–3, which act as N,P-donor ligands in various Ru catalysts, including half-sandwich ones.^28–30^ Compounds **1**–**6** represent the first examples of half-sandwich Ir–Cp^x^ compounds involving N,P-coordinated phosphinoalkylamines, specifically 2-(di-*tert*-butylphosphanyl)­ethanamine (L1 for **1** and **4**), 2-(diphenylphosphanyl)­ethanamine (L2 for **2** and **5**) and 3-(diphenylphosphanyl)­propan-1-amine (L3 for **3** and **6**). Very recently, we used L1–3 for the preparation of highly antiproliferative active half-sandwich Rh­(III) complexes.^31^ Furthermore, for the design of **1**–**6**, it is known that a donor set of the chelating ligand,^32,33^ chelate ring size^34^ or Cp ring extension (e.g., Cp*- vs Cp^ph^-based compounds in this work)^35^ affect the resulting biological activity,^5^ which was also taken into account by the authors.

Compounds **1**–**6** were synthesized through a two-step process. First, intermediate products with the general formula [Ir­(η^5^-Cp^x^)­(L1–3)­Cl]­Cl (1*–6*) were prepared as chloride salts by a typical microwave-assisted synthetic procedure using appropriate dimers [Ir­(μ-Cl)­(η^5^-Cp*)­Cl]_2_ (for 1*–3*) and [Ir­(μ-Cl)­(η^5^-Cp^ph^)­Cl]_2_ (for 4*–6*), and their interaction with L1–3 (see Experimental Section). Subsequently, metathesis of the chloride anion with PF_6_^–^ was performed by adding an excess of NH_4_PF_6_, providing the final compounds **1**–**6**. Substitution of the counter-anions and their potential influence on the resulting biological activity is not expected, as this effect has not been reported in the literature, as compounds [Ir­(η^5^-Cp^bph^)­Cl­(phen)]Cl and [Ir­(η^5^-Cp^bph^)­Cl­(phen)]­PF_6_ exhibited comparable cytotoxicity in A549 cells.^36^

### General Characterization

The purity of the obtained products was verified by elemental analysis and RP-HPLC. Elemental analysis showed less than 0.4% differences between the calculated and experimental percentage of C, H and N (see Experimental Section). HPLC revealed >95% purity of **1**–**6** in TFA-acidified water/ACN mixtures (Figure S1B). The HPLC results indicated that the lipophilicity of Cp* compounds (*t*_R_ = 7.85, 14.25, and 15.09 min for **1**–**3**) is lower as compared with their Cp^ph^ analogues (*t*_R_ = 9.88, 16.28, and 17.10 min for **4**–**6**). Analogically, L1-containing compounds with *t*-butyl substituents (**1**, **4**) are markedly less lipophilic than other studied compounds containing L2 or L3 with a phenyl-substituted phosphane group.

ESI-MS of **1**–**6** contained signals assignable (*m*/*z*, isotopic pattern) to their complex cations [IrCl­(Cp^x^)­(L1–3)]^+^ or their dehalogenated fragments {[Ir­(Cp^x^)­(L1–3)] – H}^+^ (Figures S2–S7). ESI-MS proved the structural integrity of complex cations of **1**–**6**, while the presence of PF_6_^–^ in **1**–**6** was proven by FTIR spectroscopy (peaks centered at ca. 830 and 555 cm^–1^) and by ^31^P NMR (a multiplet at δ = −143.8–(−144.4) ppm) (Figures S8–S13).

^1^H NMR spectra contain a characteristic singlet of Cp* hydrogens at 1.46–1.73 ppm (for **1**–**3**) or a set of signals at 1.51–2.09 ppm assignable to methyl groups of the Cp^ph^ ligand of **4**–**6** (Figures S8–S13). The aliphatic hydrogens of L1–3 involved in **1**–**6** were detected as an AB system at 1.59–3.45 ppm, which is consistent with the reported Ru-pcym analogues (pcym = *p*-cymene).^30^ Resonances of the aromatic hydrogens of L2, L3 and Cp^ph^ were found at 7.13–7.75 ppm. Amine hydrogens were observed as a pair of resonances at 3.68–4.32 ppm and 4.48–5.52 ppm. These −N*H*_2_ resonances were markedly shifted as compared with free L1–3,^37^ which was caused by coordination to Ir through the nitrogen donor atom. The ^13^C NMR spectra of **1**–**3** exhibit characteristic Cp* signals at 92.8–93.4 ppm (Cp ring) and 8.8–9.9 ppm (methyl groups). For Cp^ph^ compounds, two sets of aromatic (ca. 129 ppm) and aliphatic (8.3–12.8 ppm) signals were detected. ^13^C NMR signals of phosphinoalkylamines (L1–3) were also detected, as exemplified for L^3^-containing compounds **3** and **6**, which showed signals corresponding to aromatic carbon atoms at 131.2–134.1 ppm, while resonances of the propyl carbon atoms were found at 22.6–39.3 ppm. The ^31^P NMR spectra contained two resonances belonging to the above-mentioned PF_6_^–^ counteranion (ca. −144 ppm) and to the coordinated L1–3 of **1**–**6** (δ = −6.6–58.8 ppm). Large changes in the ^31^P NMR chemical shifts of the phosphorus atom (Δδ = 33.8–58.7 ppm) proved that L1–3 are also coordinated through their phosphorus atom.

### Crystallography

Crystals suitable for single-crystal X-ray analysis were prepared for one intermediate ([Ir­(η^5^-Cp*)­(L3)­Cl]­Cl·MeOH; 3*·MeOH) and one final product (**3**), differing in terms of the counteranion (i.e., Cl^–^ vs PF_6_^–^). Both **3** and 3* feature the [Ir­(η^5^-Cp*)­Cl­(L3)]^+^ complex cation, which adopts a pseudo-octahedral piano-stool arrangement with a Cp* ring, a chlorido ligand, and a bidentate-coordinated N,P-donor ligand L3 (Figure 1; Tables S1–S3). The metal–ligand bond lengths in both compounds are similar (Table S3). However, there are significant differences in the crystalline environment and supramolecular assemblies. 3* crystallizes in the orthorhombic *Pbca* space group, forming a centrosymmetric dimer with chloride anions and cocrystallized methanol molecules. The dimer is interconnected by N–H···Cl and O–H···Cl hydrogen bonds. In contrast, **3** crystallizes in the monoclinic *P*2_1_/*c* space group. Here, the centrosymmetric assembly involves disordered PF_6_^–^ anions, and the dimerization is mediated by N–H···F hydrogen bonds (Figure S14).

### Stability Studies

The stability of **1**–**6** (10 μM final concentration) in aqueous media was investigated by UV–vis spectroscopy in 5% DMF/95% PBS in H_2_O. Compounds **2**, **3**, **5** and **6** are stable in the presence of water, because no spectral changes were observed (Figure S15). The decrease in intensity is due to their partial precipitation. Stability of **2**, **3**, **5** and **6** was confirmed by control ^1^H NMR experiments, which did not show any new resonances even after standing for 24 h in a similar medium (10% DMF-*d*_7_/90% PBS in D_2_O; Figure S16). In contrast, L1-containing compounds **1** and **4** hydrolyzed in the used mixture of solvents, as indicated by the changes of their UV–vis spectra, where new maxima were observed (Figure S15).

The samples studied by UV–vis were also analyzed by ESI-MS, which showed the [IrCl­(Cp^x^)­(L)]^+^ chlorido species for the stable compounds **2**, **3**, **5** and **6**. On the other hand, the [IrCl­(Cp^x^)­(L)]^+^ chlorido species were missing in the mass spectra of hydrolytically unstable compounds **1** and **4** after 24 h of standing in the used medium (Figure S17). Surprisingly, the dominant ESI-MS peaks detected for **1** and **4** after 24 h are not assignable to {[Ir­(Cp^x^)­(L1)] – H}^+^ as one would expect from the results obtained for MeOH solution (516.2 and 578.3 *m*/*z*, respectively; see Experimental Section), but to the {[Ir­(Cp^x^)­(L1)] + H}^+^ species detected with appropriate *m*/*z* (518.3 and 580.3 *m*/*z*, respectively) and isotopic pattern. This suggests that stable Ir­(III) hydrido species form under the aqueous conditions used, a subject we will investigate further in advanced experiments and a follow-up manuscript.

The stability studies were also performed by RP-HPLC in DMEM testing medium (without serum), specifically in 10% DMF/90% DMEM, for which the control experiments were carried out in 10% DMF/90% PBS in H_2_O (Figures S18–S23). The obtained results were similar to those from UV–vis. In particular, **1** and **4** were less stable than **2**, **3**, **5** and **6**. For **1** and **4**, the HPLC peaks of the initial compounds showed at *t*_R_ = 3.10 and 3.81 min, respectively (ESI-MS: {[Ir­(Cp^x^)­(L1)] – H}^+^ at 516.3 and 578.3 *m*/*z*, respectively). Along these HPLC peaks, another one was found even in fresh solutions of **1** (*t*_R_ = 6.00 min) and **4** (*t*_R_ = 6.59 min), whose intensity increased with time (Figures S18 and S21). In analogy with the ESI-MS experiments discussed above (Figure S17), this HPLC peak corresponds to [Ir­(Cp^x^)­(L1)] + H]^+^ according to their *m*/*z* values of 518.3 (**1**; Figure S18) and 580.3 (**4**; Figure S21). Regarding **2**, **3**, **5** and **6** incubated in the presence of DMEM (*t* = 0–24 h), the obtained results showed that **3** and **6** are more stable than their analogues **2** and **5**. Specifically, no relevant changes were detected for **3** and **6** even after 24 h of standing in the presence of DMEM (Figures S20 and S23). **2** and **5** remained unchanged for 6 h, but a new peak appeared at longer incubation times at *t*_R_ = 3.67 and 4.40 min, respectively (ESI-MS: {[Ir­(Cp^x^)­(L2)] – H}^+^; Figures S19 and S22).

Finally, no evidence supporting the decoordination of the used phosphinoalkylamines and the subsequent opening of the chelate ring (i.e., hemilability) was obtained by the used analytical techniques. This suggests that the used N,P-ligands remained bidentate coordinated within the initial chelate ring under the experimental conditions used (water with various organic solvents; biological medium).

### In Vitro Cytotoxicity

The cytotoxic and potentially anticancer effects of the compounds, their precursors (N,P-ligands, Ir dimers) and the reference drug CDDP were evaluated in a panel of human cancer cell lines (THP-1 leukemia, HeLa cervical, SW982 synovial, A549 lung, MOR lung, and MOR/CPR CDDP-resistant lung cells).^38^ Further, possible off-target effects were assessed in primary (noncancer) porcine chondrocytes (PPCs) and human peripheral blood mononuclear cells (PBMCs). The obtained IC_50_ values are shown in Table 1.

**Table 1:** Determination of the Cytotoxic Effects of Ir­(III) Compounds **1**–**6** and the Reference Drug Cisplatin (CDDP) Expressed as IC_50_ Values for 72 h of Treatment^a^

	**cancer cell lines**						**primary noncancer cells**	
	THP-1	HeLa	SW982	A549	MOR	MOR/CPR	PPCs	PBMCs
**1**	>10	>10	>10	>10	>10	>10	>20	>20
**2**	>10	>10	>10	>10	>10	>10	>20	>20
**3**	6.5 ± 1.1	>10	7.8 ± 1.2	7.9 ± 1.3	8.1 ± 1.2	3.1 ± 1.1	>20	2.7 ± 1.2
**4**	6.8 ± 1.1	>10	4.7 ± 1.0	>10	7.3 ± 1.1	6.7 ± 1.0	7.1 ± 1.4	3.8 ± 1.2
**5**	2.9 ± 1.0	9.3 ± 1.1	4.4 ± 1.0	9.3 ± 1.2	8.4 ± 1.2	4.8 ± 1.1	14.7 ± 1.1	9.6 ± 2.0
**6**	5.4 ± 1.0	6.9 ± 1.2	6.9 ± 1.1	9.1 ± 1.2	>10	6.2 ± 1.1	7.0 ± 1.2	3.8 ± 1.1
CDDP	1.3 ± 0.1^b^	14.2 ± 1.2	4.9 ± 1.2	10.4 ± 1.1^c^	6.3 ± 1.1^c^	>20^c^	4.5 ± 1.2	8.6 ± 1.1^c^

a The data are shown as the mean ± SE (μM). The cancerous cell lines used were human leukemia (THP-1), cervical carcinoma (HeLa), synovial cells (SW982), lung carcinoma (A549, MOR), and CDDP-resistant lung carcinoma cells (MOR/CPR), as well as primary non-cancerous cell lines of porcine chondrocytes (PPCs) and human peripheral blood mononuclear cells (PBMCs).

b Data taken from ref 39.

c Data taken from ref 38.

Compounds **1** and **2** were inactive in the tested cancer cell lines (IC_50_ > 10 μM). **3**–**6** had detectable cytotoxic effects on all (**5**) or some (**3**, **4**, **6**) of the cancer cell lines used. The used N,P-donor ligands (L2, L3), cyclopentadienyls (HCp*, HCp^ph^) and Ir precursors ([Ir­(μ-Cl)­(η^5^-Cp*)­Cl]_2_, [Ir­(μ-Cl)­(η^5^-Cp^ph^)­Cl]_2_) were not toxic against A549 cells (IC_50_ > 20 μM);^40^ *note:* L1 was not tested because it is highly unstable when prepared and commercially available only as a 10% solution in THF. This proved that the observed cytotoxic effect was due to the whole complex, not its individual parts. Importantly, CDDP-resistant MOR/CPR cells were highly sensitive to **3**–**6** (IC_50_ = 3.1–6.7 μM; IC_50_ > 20 μM for CDDP). This observation indicates that the MoA of **3**–**6** is different from that of CDDP. Although a pharmacological perspective has been reported for Ir–Cp^x^ compounds in terms of their high activity against lung cancer at the National Cancer Institute for NCI-60 human tumor cell panel screening,^41^ this phenomenon has not been proven in an advanced model involving a variant with acquired resistance against some conventional anticancer drugs (e.g., CDDP), as discussed in this work.

The THP-1 cell line was also very sensitive to treatment with **3**–**6** (IC_50_ = 2.9–6.8 μM). However, all tested compounds were less effective than CDDP (IC_50_ = 1.3 μM) in THP-1 cells. In HeLa cells, compound **6** showed a lower IC_50_ value than CDDP (6.9 μM vs 14.2 μM), whereas in A549 cells compound **3** displayed only a modest numerical advantage over CDDP (7.9 μM vs 10.4 μM). However, no significant and consistent specificity against cancer cell lines was observed for the studied compounds when considering the full panel of primary noncancer cells tested.

Notably, compound **3** also exhibited pronounced cytotoxicity in PBMCs (IC_50_ = 2.7 μM), in the same concentration range as its activity in the most sensitive cancer model MOR/CPR (IC_50_ = 3.1 μM) and at a lower IC_50_ than CDDP in PBMCs (Table 1). Therefore, PBMC cytotoxicity represents a significant limitation for interpreting compound **3** as “cancer-selective” and suggests a potentially narrow in vitro safety margin with respect to immune cells.

Compounds **1**–**6** represent direct analogues of [Rh­(η^5^-Cp^x^)­Cl­(L1–3)]­PF_6_ complexes involving the same phosphinoalkylamines (L1–3).^31^ In contrast to the Ir complexes studied in the present study, the Rh analogues showed only moderate antiproliferative activity in A549 lung cancer cells but were more potent in A2780 ovarian carcinoma or MOLT-4 acute lymphoblastic leukemia.

Importantly, **3** had a limited effect in noncancerous adherent PPC cells (IC_50_ > 20 μM), which was not the case for **6** or CDDP (Table 1). Moreover, **3** was not cytotoxic to human liver-differentiated HepaRG cells (*vide infra*). Nevertheless, both compounds selected for further evaluation (**3** and **6**) exhibited higher cytotoxic effects on PBMCs than cisplatin, which needs to be considered as a key safety limitation.

With respect to the results of the in vitro cytotoxic activity testing, compound **3** emerges as a strong hit primarily due to its potency in the CDDP-resistant MOR/CPR model and its reduced cytotoxicity in selected adherent noncancer cell models (e.g., PPCs, HepaRG). This prioritization was based on the overall activity profile rather than on marginal differences in IC_50_ values between closely ranked compounds. However, the marked PBMC cytotoxicity indicates that any apparent selectivity is cell-type dependent and does not extend to primary immune cells. Thus, **3** (together with its Cp^ph^ analogue **6** for comparative purposes), was selected for a deeper analysis of MoA in MOR/CPR cells, as these cells were the most sensitive to it, and to inform future optimization aimed at improving the safety margin. An extended discussion regarding cytotoxicity is provided in Supporting Information.

### Stability Studies in the Presence of Small Biomolecules

The best-performing compounds **3** and **6** were tested for their stability in the presence of excess His, GSH and NADH. His is frequently involved in the covalent binding of metallodrugs with proteins, including blood transport proteins albumins.^42^ Thus, His can be used as a model amino acid for the studies of possible covalent interaction of new metallodrugs with albumins.^43^ The results obtained by ^1^H NMR on the mixtures of **3** and **6** with His indicated that they do not interact covalently, as no changes were detected in the spectra neither for His nor for the Ir compounds (Figure S24). Since this experiment was performed to investigate a possible interaction of the compounds with HSA in blood, we used lower concentrations of the chloride ions in PBS (5.5 mM Cl^–^) to better mimic the extracellular conditions. Therefore, this experiment also demonstrated the stability of **3** and **6** even when exposed to extracellular chloride ion concentration.

Further, GSH and NADH were used, because it is well established that the fate of similar Ir–Cp^x^ compounds in the physiological environment can be affected by interactions with these biomolecules.^5,22,44–46^ Again, no new signals were detected in the ^1^H NMR spectra of **3** and **6** up to 24 h of standing in the dark (r.t.), proving that these compounds are stable even in the presence of NADH or GSH. These ^1^H NMR experiments also excluded a covalent interaction between the Ir compounds and GSH, as well as no oxidation of GSH in the presence of the Ir compounds, because no ^1^H NMR signals of its oxidized form (i.e., glutathione disulfide, GSSG) were detected. On the other hand, NADH was partially oxidized by **3** and **6** (0.65 and 0.58 molar equiv of the complex, respectively; Figure S25). No hydride resonance was observed during these experiments with NADH. This moderate oxidative ability of both compounds toward NADH was suppressed by the addition of other biomolecules (GSH and ascorbate were used) mimicking the intracellular conditions in the treated cells.^46^ This is particularly important in terms of possible MoA of **3** and **6**, because an ability of Ir–Cp^x^ compounds to oxidize NADH is usually linked with their ability of the formation of ROS within the redox-mediated MoA.^15,19–22,24,25^ We demonstrated that NADH is most likely not oxidized by **3** and **6** in cells, due to the presence of numerous biomolecules (represented here by GSH and ascorbate) that scavenge such an intracellular redox process (*vide infra*).

### In Vitro Cytotoxicity in 3D Spheroids

Compounds **3** and **6** were also tested for their ability to influence A549 spheroids (i.e., in three-dimensional (3D) lung cancer cell cultures), which fill the gap between 2D cell monolayers (*vide supra*) and in vivo anticancer activity (*vide infra*) models (Figure 2). They mimic tumor behavior better than 2D cell cultures and thus are used for screening novel chemotherapeutics before activating animal protocols.^47^

**Figure 2:**
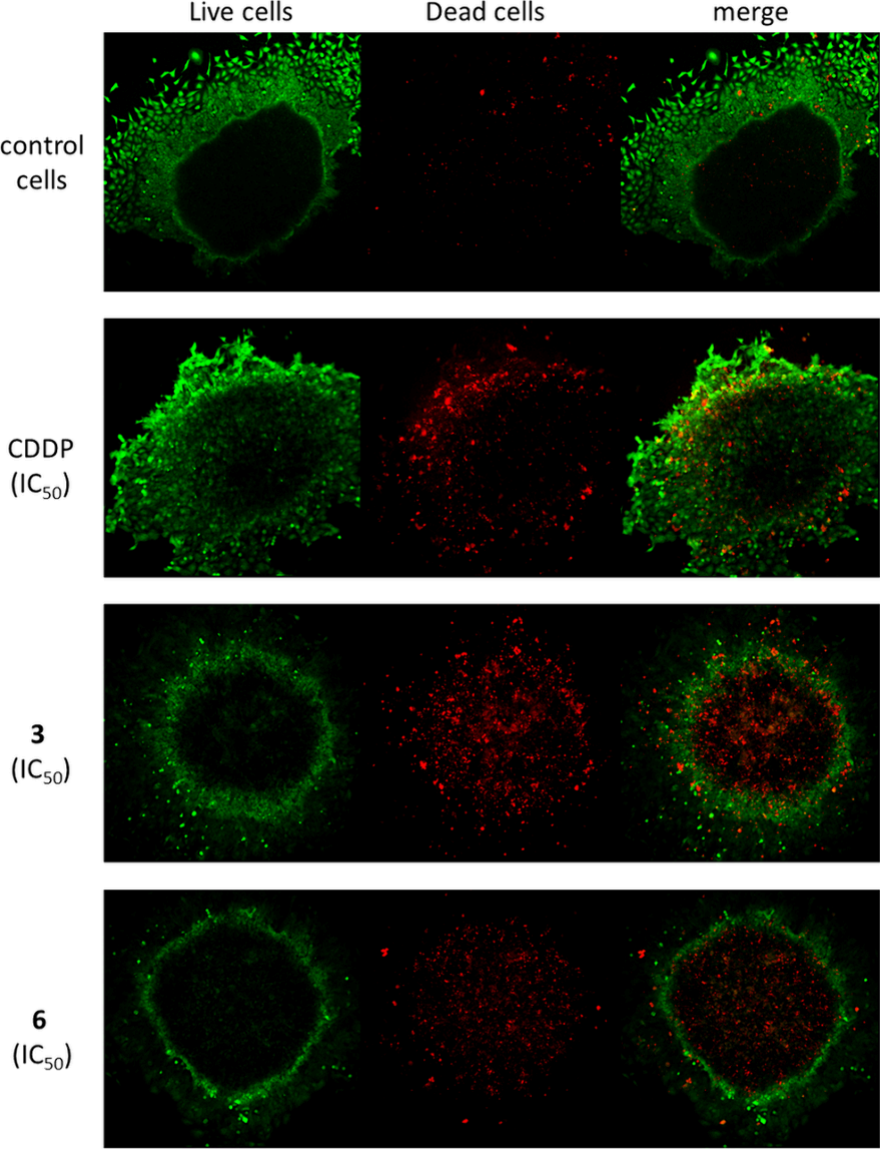
3D spheroids from A549 cells were treated with **3**, **6** and CDDP at concentrations corresponding to the IC_50_ values obtained from 2D experiments (i.e., 8 μM for **3** and 9 μM for **6**). After 72 h of incubation, the live (green) and dead (red) cells were visualized, and confocal microscopy was used to capture the images.

Both tested compounds significantly increased the number of dead cells in a similar way as CDDP. Moreover, **3** and **6** also apparently reduced the cell migration from parental spheroids. This effect was not observed for CDDP. These findings are in agreement with previous studies in which Ir­(III) complexes were shown to influence the viability and morphology of 3D cell spheroids.^48,49^ To the best of our knowledge, we demonstrated for the first time that Ir­(III) compounds can inhibit cell migration in 3D spheroids. The ability of cells to escape from the parental compact mass of cells (e.g., solid tumors) is the initial and necessary step in metastasis.^50^ Hence, compounds with the ability to diminish the migration of cells from primary tumors (such as **3** and **6**) are needed to avoid the formation of metastases and thus decrease the chance of disease relapse.

### Cell Cycle and Apoptosis Evaluation

It was previously described that platinum drugs can induce cell cycle arrest.^51^ Targeting mechanisms controlling cell cycle and cell death is a common effect of Ir–Cp^x^ compounds as well.^5,8^ Therefore, we suggested that Ir–Cp^x^ compounds could induce cell cycle arrest in CDDP-resistant MOR/CPR cell lines, which were highly sensitive to **3** and **6** (Table 1). Our results showed no significant effect after 24 h incubation of MOR/CPR cells with **3** and **6** (Figures 3A and S26). In the comparison, CDDP caused a considerable reduction of cells in the G1 phase (32.2% for CDDP vs 56.3% for untreated cells). Longer exposure to **3** and **6** (72 h) caused an increase in the number of cells in the G1 phase of the cell cycle (59.1 and 59.6%, respectively) compared to control MOR/CRP cells (51.4%) (Figures 3B and S26; 29.5% for CDDP). These results (i.e., G1 arrest) were consistent with those of Liu et al. and Mondal et al., who described the blockade of the cell cycle in the G1 phase after treatment with similar Ir–Cp^x^ compounds^52,53^ but essentially different from other Ir analogues that induce S and G2 arrest.^15^ The cell cycle analysis revealed that CDDP arrested cells in the G2/M phases of the cell cycle, as nearly 56% of the CDDP-treated cells were in the G2/M phases of the cell cycle in comparison to the cells treated by **3** (22.6%) or **6** (18.8%) (26.8% for control cells). The distribution of cells in the S phase was the lowest after treatment with CDDP and **3** (13.9 and 17.9%, respectively). Furthermore, the cells were also treated with 0.5 × IC_50_ concentrations of **3** and **6**. The cell cycle analysis showed that the cell cycle was arrested in the G1 phase in a concentration-dependent manner for **3** and **6**.

**Figure 3:**
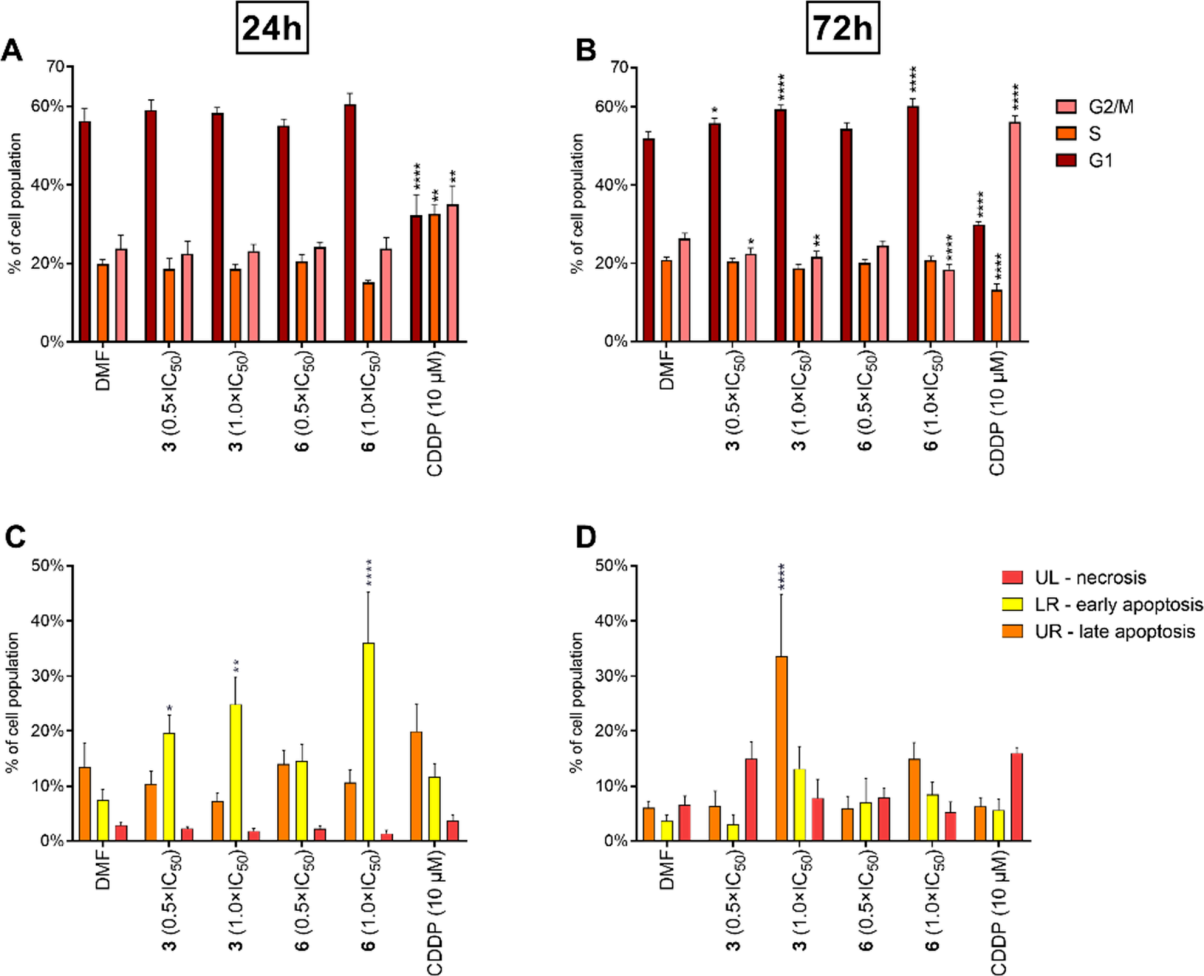
Effect of **3** and **6** on the cell cycle (A, B) and on cell death (C, D) in MOR/CPR cells. The cells were incubated with **3** and **6** at the IC_50_ and half-IC_50_ (i.e., 1.5 and 3.0 μM for **3**; 3.0 and 6.0 μM for **6**). Cisplatin (CDDP) at a concentration 10 μM served as the positive control. The analysis was performed after 24 h (A, C) and 72 h (B, D). Analysis of cell death and apoptosis (C, D) was performed using double staining with Annexin V Dyomics 642 and Live/Dead Violet. Next, the dot plots were divided into four quadrants, such as early apoptosis - lower right quadrant (LR; Annexin V positive, Live/Dead Violet negative); late apoptosis - upper right quadrant (UR; Annexin V positive, Live/Dead Violet positive); and necrotic/dead cells - upper left quadrant (UL; Annexin V negative, Live/Dead Violet positive). The data are shown as the mean ± SEM; * indicates statistical significance (*p* < 0.05) compared with the DMF group; ** indicates statistical significance (*p* < 0.01) compared with the DMF group; *** indicates statistical significance (*p* < 0.001) compared with the DMF group; **** indicates statistical significance (*p* < 0.0001) compared with the DMF group. The analyses were performed in three independent repetitions.

To determine whether **3** and **6** could induce apoptosis, the translocation of phosphatidylserine from the inner side of the plasma membrane to the outer layer was analyzed by flow cytometry. The studies were performed at two concentrations (i.e., IC_50_ and 0.5 × IC_50_), and the results confirmed the concentration-dependent effects of **3** and **6** in terms of their ability to induce apoptosis. Our results showed that **3** and **6** caused early stage of apoptosis after 24 h treatment of cells, which was not detected in cells treated by CDDP (Figures 3C and S27). Longer exposure (72 h) caused necrosis in cells treated by CDDP and by lower concentration of **3**. Higher concentrations of **3** and **6** kept the number of necrotic cells on the same level as in control untreated cells. However, apoptosis was detectable after treatment by **3** and **6**, whereas only necrosis was observed after 72 h incubation with CDDP.

To confirm the ability of compounds **3** and **6** to induce apoptosis in MOR/CPR cells, the activities of apoptosis-related caspases (Casp-3, −8, and −9) were evaluated, together with the cleavage of the Casp-3 target protein poly­(ADP-ribose) polymerase (PARP) (Figures 4 and S28). The level of cleaved Casp-8 (extrinsic pathway) remained unchanged after treatment with **3**, **6**, or CDDP. In contrast, cleavage of Casp-9 by **3** and **6**, but not by CDDP, indicates activation of the mitochondrial apoptotic pathway only in this CDDP-resistant cell line. This finding correlates with the level of cleaved PARP. It should be noted that a decreased PARP level correlated with a decreased Casp-3 level only in untreated control cells. In CDDP-treated cells, the level of cleaved PARP was lower, whereas the level of activated Casp-3 remained unchanged. In contrast, MOR/CPR cells are CDDP-resistant, and their response to cisplatin differs from that of other CDDP-sensitive cells. This finding is suggestive for the involvement of caspase-independent cell-death types (e.g., necroptosis, ferroptosis or autophagic cell death), as reported for similar Ir–Cp^x^ compounds.^54–58^

**Figure 4:**
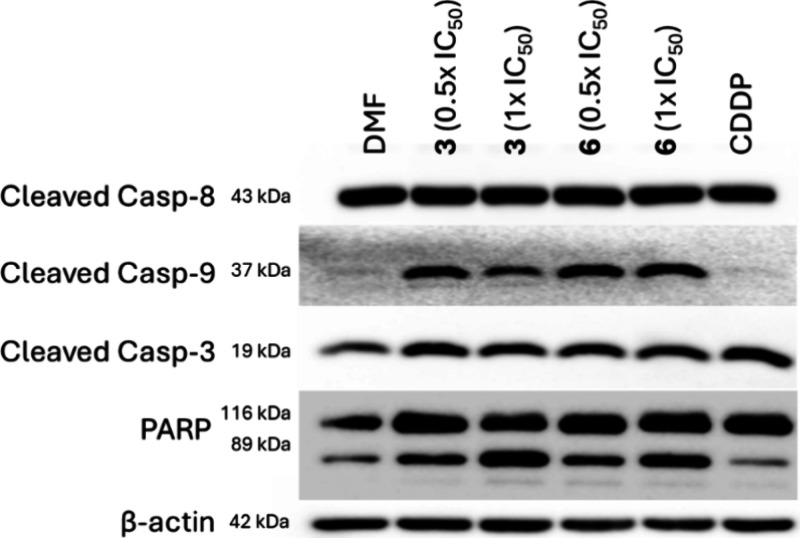
Effect of **3** and **6** on the level of apoptosis-related proteins in MOR/CPR cells. The cells were incubated with **3** and **6** at the IC_50_ and half-IC_50_, (i.e., 1.5 and 3.0 μM for **3**; 3.0 and 6.0 μM for **6**) for 24 h. Cisplatin (CDDP) at a concentration 10 μM served as the positive control. Shown membranes represent one of three independent replicates. Casp - caspase; PARP - poly­(ADP-ribose) polymerase.

From a general point of view, the ability to induce apoptosis, mostly due to disruption of ROS-mediated mitochondrial membrane function, is a well-described phenomenon for Ir–Cp^x^ compounds.^10,15,17^ This aligns with reports that Ir compounds can induce apoptosis through a combination of DNA and mitochondrial damage. For instance, Ir compounds were found to intercalate DNA minor grooves and simultaneously cause mitochondrial ROS formation, G0/G1 arrest and apoptosis in cancer cells.^59^ The lack of strong G1 arrest implies that probably DNA damage is not the dominant lethal event and the cells likely undergo apoptosis before cell-cycle checkpoints fully engage.

### Stress-Related Gene Expression in A549 Lung Cancer Cells

The cellular stress response is activated when cellular homeostasis is disrupted and can be detected before cytotoxicity is visible/measurable. Therefore, we employed qRT–PCR to reveal the changes in the expression of genes involved in cellular stress responses in A549 cells treated with **3** and **6**, or CDDP for 5 or 24 h (Figure 5).

**Figure 5:**
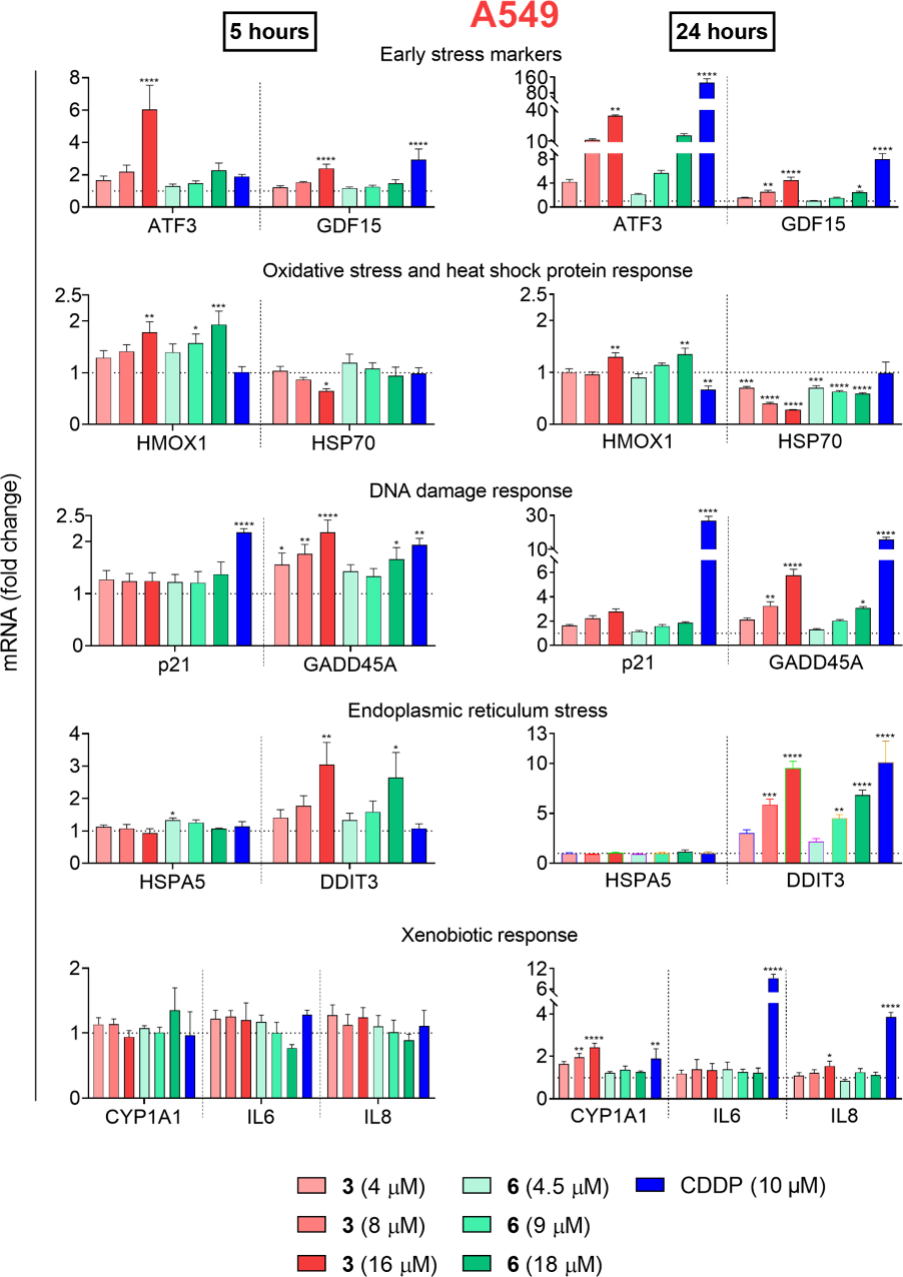
Induction of cellular stress responses. A549 cells were exposed to **3** (red), **6** (green), or CDDP (blue) for 5 or 24 h. Total RNA was isolated, and the mRNA levels of cellular stress-related genes were determined via qRT–PCR. The data are expressed as the mean ± SD of three independent experiments, each performed in duplicate. * *p* < 0.05, ** *p* < 0.01, *** *p* < 0.001, and **** *p* < 0.0001 compared to vehicle (DMF)-treated controls.

Experimental data indicate that **3** and **6** elicit rapid cellular stress responses distinct from CDDP. For example, **3** causes an early up-regulation (up to 6×) of the ATF3 gene (a stress-inducible transcription factor) in A549 cells within 5 h of treatment, whereas CDDP shows no effect at that early time point. By 24 h, both **3** and CDDP strongly up-regulate ATF3 (up to 34×, and 132×, respectively), while **6** shows a lower increase (up to 15×). Similarly, DDIT3 (CHOP), a key mediator of the unfolded protein/ER stress response, is up-regulated by **3** and **6** as early as 5 h (both up to ∼3×), but not by CDDP even at 24 h. This suggests that **3** and **6** trigger integrated stress response pathways (likely via PERK/eIF2α/ATF4) more quickly than CDDP, which is consistent with activation of ER stress genes like ATF3 and CHOP.^60^ In contrast, CDDP’s primary damage response is slower to engage these pathways.

CDDP’s well-known mechanism involves DNA platination and p53-mediated damage response, reflected in strong induction of p21 (CDKN1A = CDK inhibitor 1A) and GADD45A in treated cells. Indeed, by 24 h CDDP strongly up-regulates p21 (a cell cycle inhibitor) and GADD45A (a DNA damage-inducible gene) in cancer cells (up to 27×, and 16×, respectively). In contrast, Ir compounds induce little (**3**) or even no (**6**) p21 after 24 h. GADD45A is induced to a lesser degree by **3** (up to 6×) and only weakly by **6** (up to 3×), compared to CDDP’s robust up-regulation. These differences indicate that **3** and **6** inflict less direct DNA damage or p53 activation than CDDP. Instead of causing G1/S arrest via the p21 pathway, **3** and **6** may bypass classic DNA damage checkpoints. The relatively muted p21 response to **3** and **6** suggests they do not heavily rely on p53-mediated DNA damage signaling to kill cells. Instead, apoptosis likely occurs via intrinsic (mitochondrial) pathways or other stress-response pathways. Some Ir complexes are known to trigger mitochondria-dependent apoptosis as a downstream result of ROS and mitochondrial disruption.^59^

Compound **3** (and to a lesser extent **6**) caused a down-regulation of HSP70 gene expression in A549 cells by 24 h (up to 0.3×, and up to 0.6×, respectively), whereas CDDP did not. HSP70 is typically induced under proteotoxic stress, so its suppression suggests an altered proteostasis response. One interpretation is that **3** and **6** might impair the heat shock response or overwhelm protein folding mechanisms, preventing up-regulation of chaperones. Meanwhile, HSPA5 (GRP78/BiP), a marker of ER unfolded protein response (UPR), remained largely unchanged in cancer cells for all treatments – implying that while CHOP was induced, the upstream chaperone BiP was not significantly elevated by these doses, possibly due to transient or partial UPR activation. Overall, the suppression of HSP70 alongside early DDIT3 (CHOP) up-regulation in **3**- and **6**-treated cells suggests a protein misfolding stress that may proceed to apoptosis before a full heat-shock/UPR protective response can mount.

HMOX1, an oxidative stress indicator, was weakly up-regulated at 5 h by both Ir compounds (up to 1.8× for **3**, and up to 1.9× for **6**) (and not by CDDP) in A549 cells. By 24 h this effect subsided (no change with **3** and **6**; CDDP showed a slight decrease in HMOX1, up to 0.7×). This transient HMOX1 induction hints that **3** and **6** may cause an early burst of ROS or redox stress. This also relates to the above-discussed results on the ability of **3** and **6** to oxidize NADH, which is known to be closely related to the ROS formation within the redox-mediated MoA of anticancer Ir–Cp^x^ compounds.^15,21,22^ Since the NADH-to-NAD^+^ oxidation process was suppressed by the presence of other small biomolecules (GSH or ascorbate),^46^ it is most likely not occurring within the real physiological environment of treated cells. Thus, the origin of the induced ROS and HMOX1 induction is most likely different from the redox process involving the NADH coenzyme.

In contrast to HMOX1, pro-inflammatory cytokine genes behaved differently: CDDP treatment led to significant up-regulation of IL-6 and IL-8 by 24 h in cancer cells (up to 9×, and 4×, respectively), whereas **3** and **6** did induce neither IL-6 nor IL-8 (if anything, **6** showed a minor transient IL-6 decrease). This is a striking divergence – CDDP’s DNA-damage can trigger NF-κB-mediated cytokine secretion (part of senescence or damage responses), but the Ir compounds do not strongly activate these inflammatory pathways in the cancer cells. In fact, the absence of IL-6/IL-8 induction might indicate that **3**- and **6**-treated cells undergo faster death or a form of cell death (e.g., immunogenic apoptosis) that does not elicit the same pro-inflammatory profile. Consistent with this idea, recent in vivo studies found that a cyclometalated organelle-targeted Ir compound induced an antitumor immune response while dampening tumor-induced inflammation, unlike conventional treatments.^61^

### Stress-Related Gene Expression in HepaRG Noncancerous Cells

To study the off-target toxicity of Ir–Cp^x^ compounds, we used liver-differentiated HepaRG cells, an in vitro model closest to human primary hepatocytes. The liver is the primary organ for the metabolism of xenobiotics, including drugs. Thus, liver cells are suitable for evaluating the effects of anticancer agents on normal cells. Moreover, the liver is an organ, where Ir was detected at high concentration in vivo (*vide infra*). To evaluate liver cytotoxicity and cellular stress responses, **3** and **6** were studied, and CDDP was used for comparison (Figure 6). First, we applied an LDH cytotoxicity assay (Roche) and found no significant cytotoxicity in HepaRG cells exposed to 10 μM **3**, **6** or CDDP for 24 h (Figure S29).

**Figure 6:**
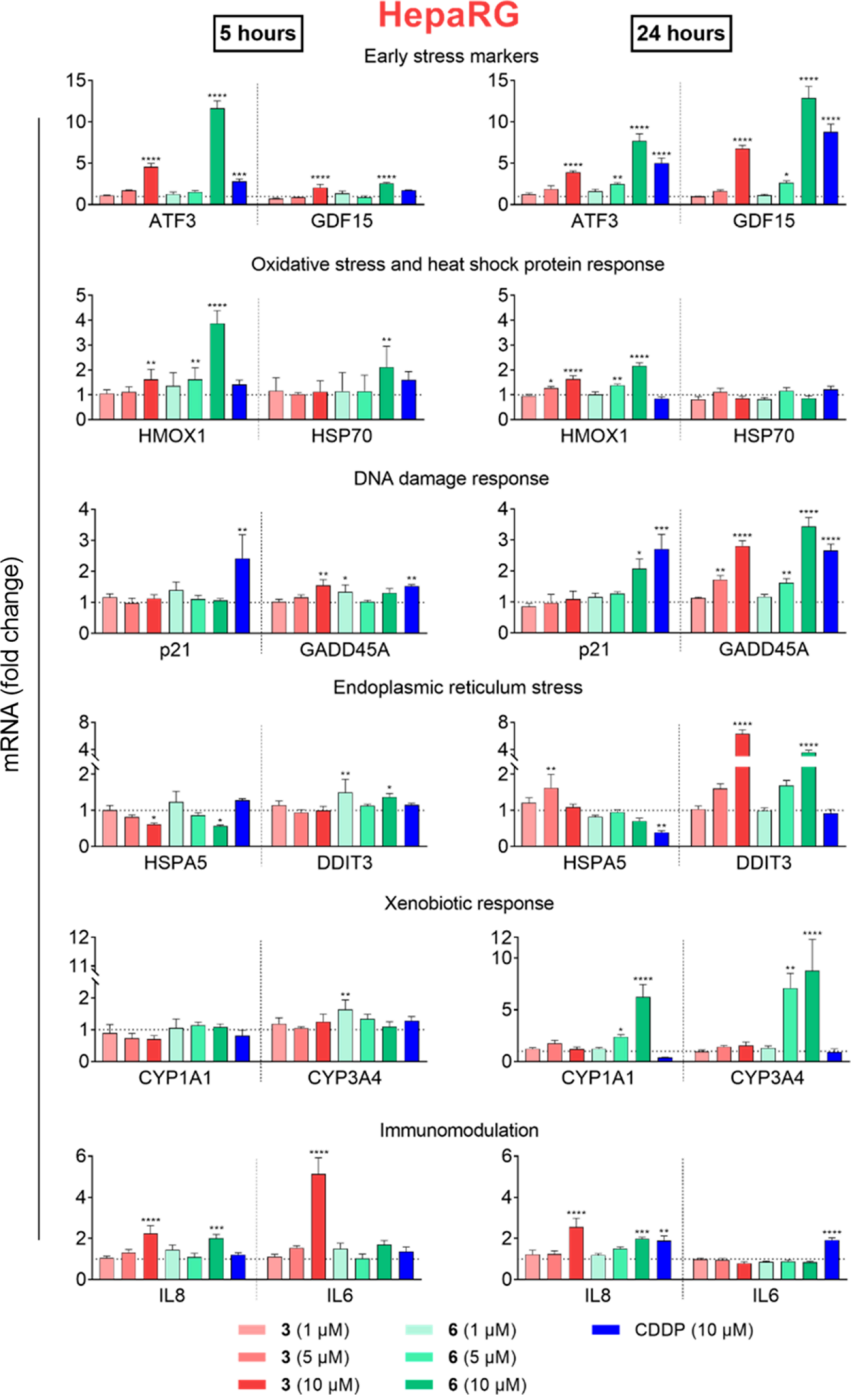
Induction of cellular stress responses. Differentiated HepaRG cells were exposed to **3** (red), **6** (green), or CDDP (blue) for 5 or 24 h. Total RNA was isolated, and the mRNA levels of cellular stress-related genes were determined via qRT–PCR. The data are expressed as the mean ± SD of three independent experiments, each performed in duplicate. * *p* < 0.05, ** *p* < 0.01, *** *p* < 0.001, and **** *p* < 0.0001 compared to vehicle (DMF)-treated controls.

The early stress toxicity markers ATF3 and GDF15 were induced in a concentration-dependent manner by all three tested substances. Induction of this gene was detected as early as after 5 h of exposure and persisted for 24 h. On the other hand, the oxidative stress marker HMOX1 was elevated only by treatment with **6** (4×), while only a slight effect was induced by **3** (1.6×) after 5 h of incubation. The expression of the HSP response marker in the cytoplasm, HSP70, increased only transiently and only by 10 μM **6** (2×) after 5 h of incubation.

CDDP, a typical inducer of DNA damage, increased the expression of both the DNA damage response marker p21 and GADD45A. GADD45A mRNA level was also elevated by **3** and **6** at 10 μM concentrations after 24 h (2.8× and 3.4×, respectively), whereas CDDP increased its level by only a factor of 2.6. The p21 gene was upregulated only by 10 μM **6** after 24 h 2.1× (2.7× for CDDP).

Importantly, only **3** and **6** (but not CDDP) were able to induce ER stress in HepaRG cells, as suggested by the elevated expression of one of the key ER stress markers DDIT3 after 24 h exposure to both **3** (6.3×) and **6** (3.6×).

Compound **6** (containing a phenyl-extended Cp^ph^ ring) but not **3** exhibited increased expression of the xenobiotic response markers CYP1A1 and CYP3A4 (members of the cytochrome P450 family) after 24 h, indicating activation of the intracellular aryl hydrocarbon receptor (AhR) and the pregnane X receptor (PXR), respectively. **3** induced the expression of both markers of immunomodulation, interleukins IL6 and IL8, after 5 h of exposure. IL8 mRNA levels were slightly increased by **3**, **6** and CDDP after 24 h.

### Discussion of the Results of Stress-Related Gene Expression

The gene expression trends strongly suggest that **3** and **6** do not primarily act by metalation of nuclear DNA (unlike CDDP). This is corroborated by extensive research on nonplatinum half-sandwich metallodrugs: they tend to kill cancer cells through mechanisms other than direct DNA cross-linking.^4,5^ Ir–Cp^x^ compounds often accumulate in specific organelles such as mitochondria, disrupting their function. Indeed, a recent study of a cyclometalated Ir complex showed it preferentially accumulated in mitochondria and ER of tumor cells, where it triggered mitochondrial membrane depolarization and pronounced ER stress.^61^ We propose that **3** and **6** follow a similar paradigm: upon entering cells, they may localize to mitochondria and/or ER membranes. As for **3** and **6**, the induced activation of UPR caused by ER stress was not observed for the reference drug CDDP. Our screening also suggested that PERK-ATF4 arm of the UPR was activated and involved in ER stress response to **3** and **6**, which may be associated also with the induction of antioxidant enzyme HMOX1 (*vide infra*).^62^ In general, ER stress might be a subject of future investigation in the field of anticancer Ir–Cp^*x*^ compounds. Regarding all types of Ir compounds, ER has been studied as a target for several cyclometalated (i.e., structurally different from **3** and **6**) compounds,^63^ but only one study had discussed ER as one of the target organelles for Ir–Cp^x^ compounds.^64^ A great promise of ER as a target of innovative anticancer metallodrugs was recently proved for compounds of other d-block metals.^38,65,66^

The early loss of mitochondrial potential and ROS burst is a known consequence of many Ir agents,^59^ and the upregulation of HMOX1 and ATF3 by 5 h with these compounds is consistent with an acute oxidative stress response. The HMOX1 upregulation induced by **3** and **6** (but not by CDDP) corresponds with the effect of similar Ir­(III) half-sandwich compounds on gene transcription, where the authors used RNA sequencing to identify the MoA and they concluded that stress, especially oxidative stress (proved by high differential expressions of various markers including HMOX1, response pathways are activated during Ir­(III) complex action in cells.^15^ Oxidative stress-induced damage of HSPs in the cytoplasm could be associated with the observed transient slight increase in the expression of HSP70 slightly by **3**.

Finally, **6** but not **3** activated AhR and PXR receptors. Both receptors are activated by xenobiotic compounds, including drugs, and are involved in detoxification reactions in the liver.^67^ The different potential of AhR and PXR activation between the compounds might be associated with the differences in a Cp^x^ ring, where **6** bears more lipophilic Cp^ph^ moiety.

Complexes **3** and **6** trigger a distinct spectrum of biological effects compared to the reference drug CDDP.^68–70^ Rather than behaving as simple DNA-damaging agents, they act as cellular disruptors, provoking early stress responses (ATF3, DDIT3, GDF15) while only weakly activating DNA repair pathways.^71,72^ This suggests their MoA involves, likely causing mitochondrial dysfunction, and unfolded protein stress that lead to apoptosis (and possibly immunogenic cell death) without heavily relying on p53/p21 signaling. Finally, comparing **3** vs **6**, subtle differences in their gene perturbation profiles suggest that ligand structure modulates their behavior (e.g., the tendency of **6** to induce xenobiotic metabolism genes vs the faster stress signaling of **3**). Such nuances underscore the tunability of Ir–Cp^*x*^ compounds by altering ligands.

### Mitochondrial Function Evaluation

The changed expression of stress-related genes suggested that **3** and **6** could dysregulate mitochondrial function. Therefore, we focused on mitochondrial function in A549 cells treated by **3** and **6** (and CDDP for comparative purposes).

The results clearly revealed that **3** and **6** caused mitochondrial membrane depolarization (Figure 7). Interestingly, **6** exhibited a strong effect. Our results showed that more than 60% of the treated cancer cells possessed mitochondria with depolarized membrane. On the other hand, **3** and the reference drug CDDP displayed comparable effects in the treated cells, where these compounds affected only 7% of cells. This difference may be caused by higher lipophilicity of **6**, which facilitates transport across cell and organelle membranes. Subsequently, this could disrupt mitochondrial function.

**Figure 7:**
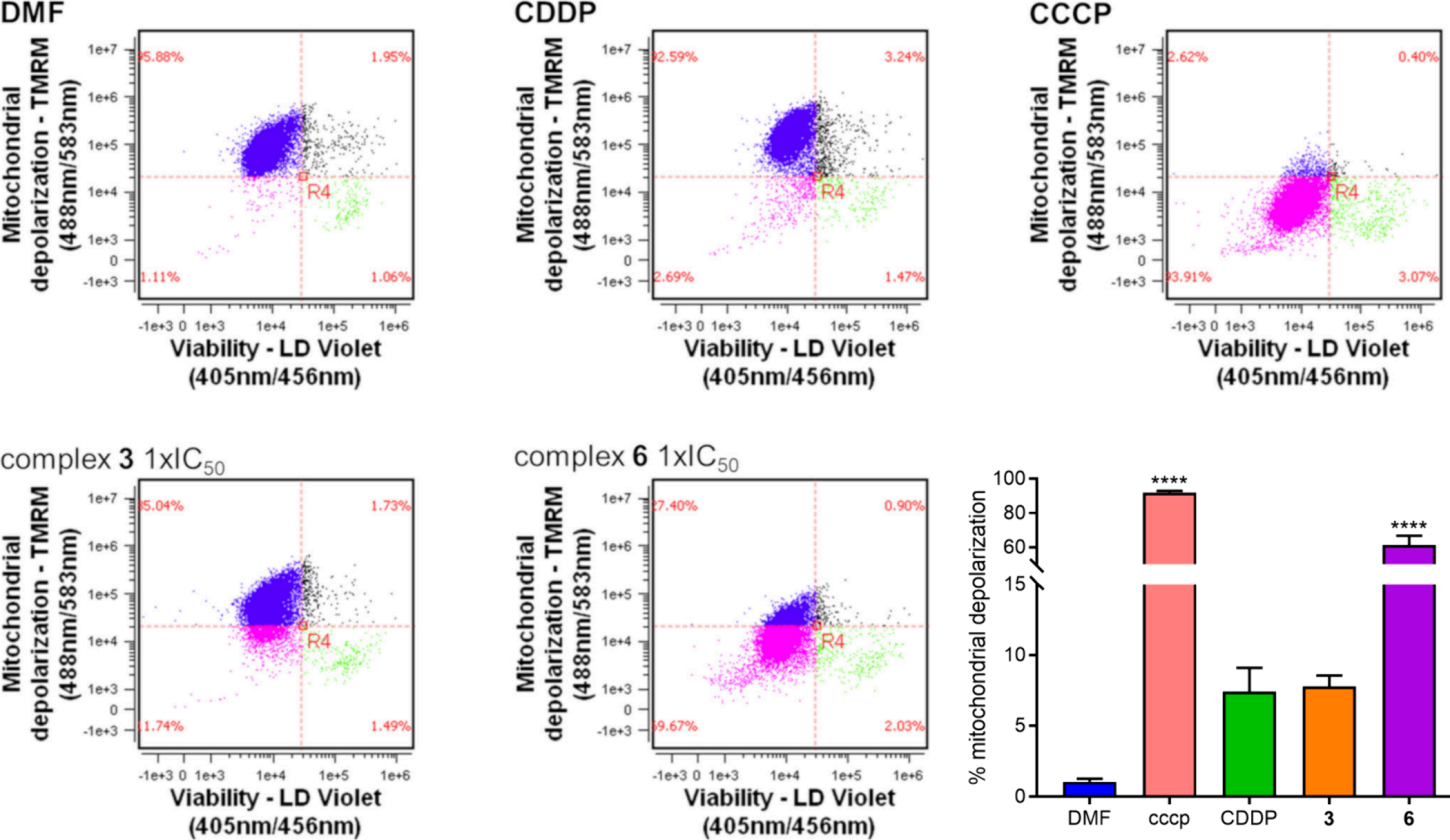
Effect of **3** and **6** on mitochondrial membrane depolarization in the A549 cell line. The cells were incubated with the compounds at the IC_50_ concentrations (i.e., 8.0 μM for **3** and 9.0 μM for **6**). Cisplatin (CDDP; 10 μM) and carbonyl cyanide chlorophenylhydrazone (cccp; 50 μM) were used as controls for impaired mitochondrial membrane potential. The analysis was performed after 24 h. Tetramethylrhodamine methyl ester (TMRM) staining and flow cytometry analysis was used for the detection of changes of mitochondrial membrane potential. Dot-plots show distribution of signals obtained during flow cytometry analysis - *X* axis represents number of dead cells, and *Y* axis represents TMRM signal intensity. The data in the bar chart shows the mean ± SEM; **** indicates statistical significance (*p* < 0.0001) compared with the DMF group. The analyses were performed in three independent repetitions.

Ir­(III) compounds – especially Ir–Cp^x^ ones – have emerged as potent anticancer agents that uniquely disrupt mitochondrial function in tumor cells.^5,15^ These cationic complexes often accumulate in mitochondria (drawn by the organelle’s negative membrane potential), where they can induce mitochondrial dysfunction and cell death.^8^ Current studies also highlight that Ir compounds can be tuned to destabilize mitochondria in cancer cells by collapsing membrane potential, overproducing ROS, and sabotaging oxidative phosphorylation. By exploiting these mitochondrial vulnerabilities, Ir compounds induce cell death through intrinsic apoptotic pathways, offering a promising strategy to overcome drug resistance and kill cancer cells via mitochondrial dysfunction.^8,73^

### Proteomic Profiling Reveals a Multiorganelle Stress Response Induced by **3**

A concise summary of the principal mechanistic insights obtained from proteomic profiling is presented below. Detailed protein-level alterations, full protein names, statistical parameters (including *p* values), pathway enrichment outputs, and extended literature-supported discussion are provided in the Supporting Information (Figures S30–S52).

#### Distinct Mode of Action of **3** vs CDDP

Principal component analysis (PCA) of the proteomic data (Figure 8) clearly demonstrated that **3** acts through a MoA distinct from CDDP. The proteomic profiles of cells treated with **3** and **6** clustered near each other, indicating related but not identical cellular responses. Consistent with previous reports, Ir–Cp^x^ compounds primarily induce mitochondrial dysfunction and ROS, whereas CDDP acts predominantly through DNA cross-linking and direct genotoxic stress.^74,75^ Because **3** caused the stronger global proteomic perturbation, in agreement with the stress-related gene expression profile (Figure S30), subsequent mechanistic analyses focused on this compound (Figure S31).

**Figure 8:**
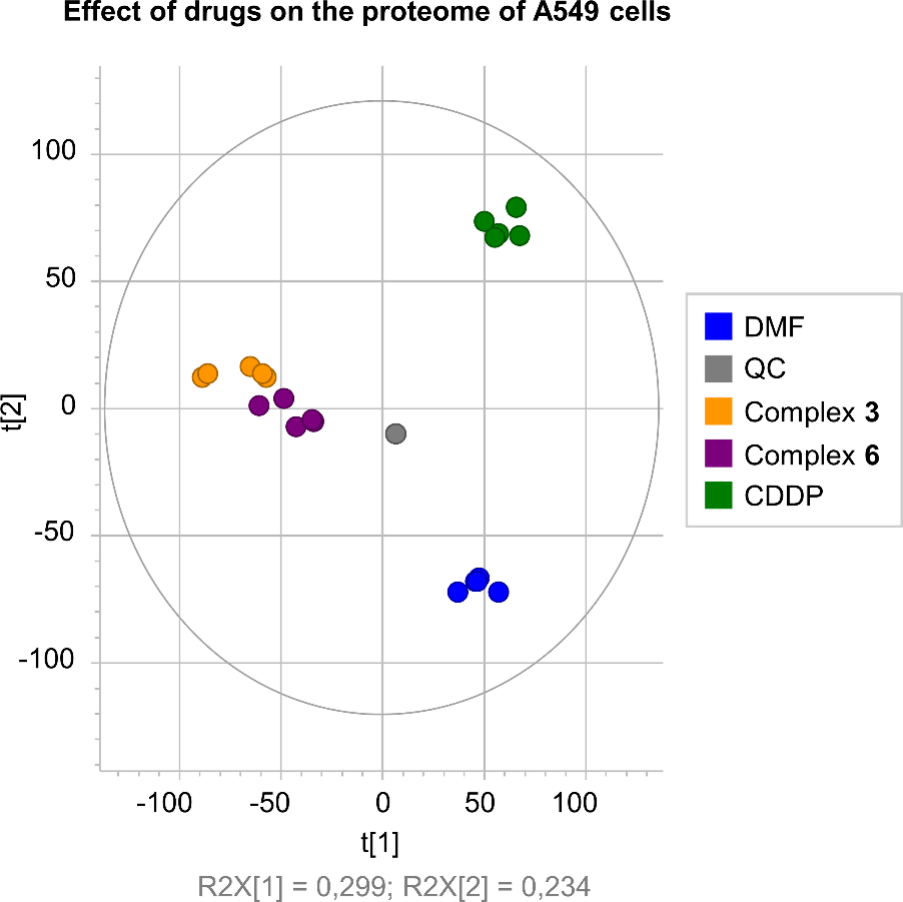
Proteomic analysis at the PCA level for **3** and **6** (and CDDP for comparative purposes). The number of QC samples is **6**. After LOESS and normalization, they clustered in close proximity (SIMCA 18.0.1).

#### Nucleolar/Ribosome Biogenesis Stress

Proteomic profiling identified the nucleolus and ribosome biogenesis machinery as major intracellular targets of **3**. Gene ontology (GO) enrichment among increased proteins revealed strong activation of pathways associated with ribosomal RNA (rRNA) processing, rRNA metabolism, preribosomal complexes, and ribosome assembly (Figure S32A–D). This pattern suggests induction of a compensatory response aimed at maintaining ribosome production under stress conditions.

Despite this apparent activation, multiple mature ribosomal components were reduced, including both cytoplasmic and mitochondrial ribosomal proteins (Figure S33), with mitochondrial ribosomes being particularly affected (Figure S32E–H). These findings indicate that **3** disrupts ribosome biogenesis and ribosomal stability, leading to translational suppression, especially of mitochondrial protein synthesis. Such effects are consistent with nucleolar/ribosomal stress and may involve mammalian target of rapamycin (mTOR) inhibition, p53 activation, or direct perturbation of nucleolar homeostasis.^76–78^ Additional protein-level details are provided in the Supporting Information (Figures S33–S36).

#### Endolysosomal Remodeling and Trafficking

As a bulky lipophilic cation, **3** is expected to enter cells through both passive membrane permeation and endocytic uptake (Figures 9, S37 and S38). Proteomic evidence supports the latter route, including increased abundance of phosphatidylinositol-binding clathrin assembly protein (PICALM) and adaptor protein complex AP-2, together with enrichment of vesicle-trafficking GO terms (Figure S32). This profile is consistent with active internalization through clathrin-associated pathways.^79–81^

**Figure 9:**
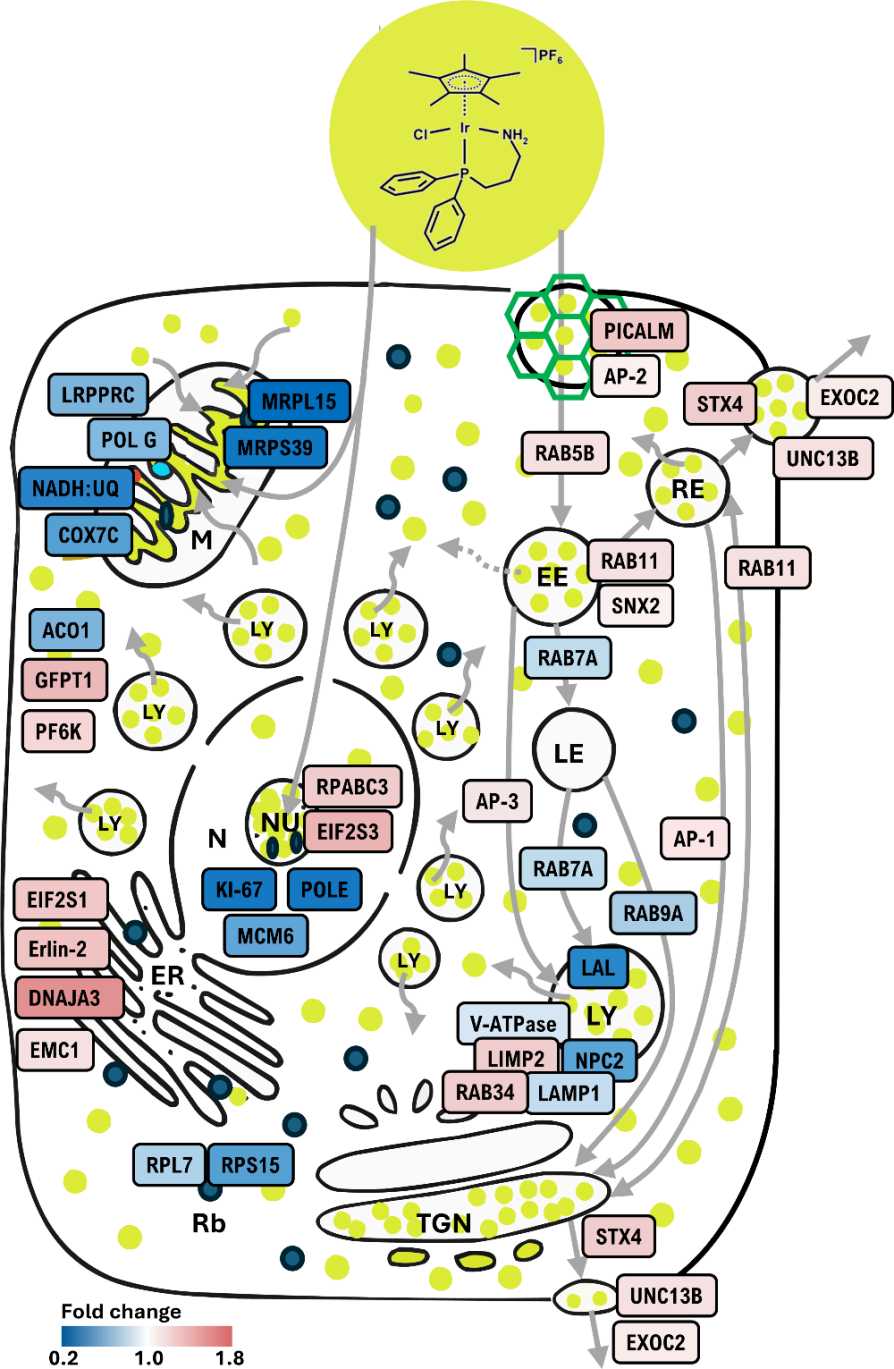
Probable mechanism of action of **3**: Schematic overview of the intracellular trafficking and fate of the compound. The drug enters cells via PICALM/AP-2-mediated endocytosis and is directed to early endosomes (EE) via RAB5B. It proceeds to recycling endosomes (RE) via RAB11 and to lysosomes (LY) via AP-3. The late endosomal (LE) pathway is suppressed, as indicated by reduced RAB7 and RAB9. From RE, the compound is trafficked to the trans-Golgi network (TGN) via RAB11 and AP-1, and returns via AP-1. Lysosomal accumulation is driven by high cholesterol content (NPC2↓), impaired degradation (LAL↓), and defective autophagosome maturation (LAMP1↓). Lysosomes accumulate perinuclearly, and acidification is impaired (V-ATPase↓), enabling cytoplasmic escape via passive diffusion and LIMP2. The compound enters the nucleus and accumulates in the nucleolus. Upregulation of RPABC3 and EIF2S indicates enhanced transcriptional and translational activity, while downregulation of *K*_i_-67, POLE, and MCM proteins reflects cell cycle arrest and reduced DNA replication. Upregulation of DNAJA3, EIF2S1, Erlin-2, and EMS1 indicates activation of stress response pathways and cellular remodeling. The compound enters mitochondria driven by membrane potential, where downregulation of MRPL15, MRPS39, LRPPRC, POLG, Complex I, and COX7C indicates impaired mitochondrial translation, mtDNA replication, and oxidative phosphorylation. Downregulation of ACO1 and upregulation of GFPT1 and PFKFB suggest a metabolic shift toward glycolysis and hexosamine biosynthesis. Partial exocytosis is initiated, as indicated by increased STX4, UNC13B, and EXOC2.

Once internalized, **3** profoundly remodeled the endolysosomal system (Figures 9, S39 and S40). Increased AP-1/AP-3 complexes, RAB5 family proteins, and recycling-associated factors indicate enhanced early endosome fusion, cargo sorting, and vesicle turnover. In contrast, reduced RAB7A and RAB9A suggest impaired maturation toward late degradative compartments. Multiple vacuolar-type ATPase (V-ATPase) subunits and lysosomal hydrolases were decreased, indicating defective acidification and reduced degradative capacity. Changes in membrane proteins (LAMP1↓, LIMP-2↑) and lipid-handling factors (NPC2↓, PSAP↓) further support lysosomal dysfunction and retention of lipophilic Ir species.

At the same time, increased abundance of exocyst components (EXOC2, EXOC4, EXOC7), soluble N-ethylmaleimide-sensitive factor attachment protein receptor (SNARE) proteins such as STX4, and vesicle-priming factors (UNC119B, UNC13B) suggests compensatory attempts to expel retained cargo through exocytosis. Overall, these data support a trafficking model in which **3** enters cells partly by endocytosis, accumulates in lysosomes and recycling endosomes, and induces a state of increased uptake but impaired degradation (Figure 9).

#### Mitochondrial Dysfunction and Metabolic Adaptation

Mitochondria emerged as the principal functionally affected organelle. **3** strongly suppressed mitochondrial translation, as evidenced by reduced mitoribosomal proteins (Figure S33) and decreased abundance of translation-supporting factors such as SLIRP, LRPPRC, and NOA1. Proteins involved in mitochondrial DNA maintenance, including POLG and MRM3, were likewise decreased, suggesting impaired mitochondrial genome stability and reduced synthesis of mitochondrially encoded respiratory subunits (Figure S41).

Accordingly, multiple components of the respiratory chain were reduced, with particularly strong effects on complexes I and IV (Figures S41 and S42), indicating impaired oxidative phosphorylation and decreased adenosine triphosphate (ATP) production. This mitochondrial dysfunction was accompanied by induction of proteostasis pathways, including mitochondrial chaperones and proteases (CLPX, LONP1, CLPB, DNAJA3), consistent with activation of the mitochondrial unfolded protein response (UPRmt).^82^

Proteostasis stress was not restricted to mitochondria. Increased abundance of endoplasmic reticulum (ER) chaperones and protein-folding factors, including HSPA5/BiP, HYOU1, SEC61A1, and ERLIN2, suggests concomitant activation of ER quality-control pathways and unfolded protein response signaling (Figures S43 and S44).

Cells simultaneously underwent marked metabolic rewiring (Figures S45–S47). Glycolytic enzymes, including HKDC1 and PFKP, were increased, whereas multiple tricarboxylic acid (TCA) cycle proteins, including CS, ACO1, SDHA, and SDHB, were reduced, indicating a shift from mitochondrial oxidation toward cytosolic ATP production. Such glycolytic compensation is a common response to impaired oxidative phosphorylation.^83,84^ Increased abundance of oxidative pentose phosphate pathway enzymes (H6PD, 6PGL), together with glutathione-associated factors (GSS, SLC25A40, MGST1), supports nicotinamide adenine dinucleotide phosphate (NADPH)-dependent redox defense. Activation of PPP-derived NADPH production is a well-established mechanism for maintaining glutathione homeostasis during oxidative stress.^85,86^

Strong upregulation of the serine synthesis pathway (PHGDH, PSAT1), together with induction of mitochondrial one-carbon enzymes (MTHFD1L, MTHFD2, SHMT2), suggests rerouting of carbon flux toward antioxidant buffering, nucleotide production, and selective biosynthesis. Serine/one-carbon metabolism is closely linked to glutathione synthesis, folate cycling, and proliferative stress adaptation.^87–90^ In parallel, nucleotide biosynthesis pathways were activated, likely to sustain repair and survival under oxidative and replicative stress. Enhanced nucleotide metabolism commonly accompanies DNA damage responses and replication stress.^91,92^

Together, these findings suggest that **3**-treated cells enhance mitochondria–purinosome coupling, redirecting serine-derived one-carbon units through the mitochondrial arm of the pathway to support de novo purine biosynthesis despite impaired cytosolic one-carbon metabolism. This metabolic rewiring likely reflects a compensatory response to **3**-induced DNA damage and replicative stress, supporting nucleotide replenishment for repair and survival. Increased abundance of AK3 and nicotinamide phosphoribosyltransferase (NAMPT) further supports tight regulation of nucleotide pools and redox balance under oxidative pressure.

#### Replication Arrest and p53-Associated Cytostasis

Compound **3** induced broad cell-cycle suppression, primarily through inhibition of the G1/S transition and reduced mitotic activity (Figures S48–S50). Core components of DNA replication licensing and elongation, including MCM2–7, proliferating cell nuclear antigen (PCNA), replication polymerases, and replication factor C (RFC) subunits, were significantly decreased. Concomitant reduction of cyclin-dependent kinases (CDK1/2/4/6) and CDK7 indicates weakened retinoblastoma protein phosphorylation and reduced E2F-dependent S-phase entry.

This profile is consistent with a predominantly cytostatic, quasi-quiescent phenotype rather than acute mitotic catastrophe. Mitotic markers such as TOP2A, KIF2C, TTK, and *K*_i_-67 were also reduced, indicating fewer cells progressing into mitosis.

Cell-cycle arrest was accompanied by activation of a p53-dependent DNA damage and replication-stress response (Figures S51 and S52). Increased PDRG1, TP53I3, PUMA, and p53R2 indicate checkpoint activation and stress adaptation. In parallel, elevated abundance of several proteins linked to mitochondrial apoptosis (Figure S52), including BAX, DIABLO/SMAC, AIFM1, BAD, HTRA2, VDAC1/2, ANT1/2, FIS1, OPA1, and MFF, is consistent with substantial priming of the intrinsic apoptotic pathway.^93,94^ Upregulation of both BAX and VDAC2 indicates a mitochondria-centered stress response in which cells become primed for BAX-dependent apoptosis while simultaneously stabilizing the outer mitochondrial membrane through VDAC2-mediated regulation.^95–97^ Concurrent upregulation of VDAC1, VDAC2, and BAX further suggests a mitochondrial state combining sustained metabolite flux and membrane adaptation with increased susceptibility to apoptotic permeabilization.

At the same time, proteins associated with death receptor signaling, including TNFRSF10A (DR4), TNFRSF10B (DR5), TRADD, and TRAF2, were also detected, indicating that contribution of the extrinsic pathway cannot be excluded.^98–100^ Together, these data support a model in which mitochondrial apoptosis predominates, while receptor-mediated apoptotic signaling may contribute under selected conditions.

#### Integrated Mechanistic Model

Collectively, the proteomic data indicate that **3** exerts a multifactorial, noncisplatin-like mode of action centered on interconnected organelle stress pathways. The compound perturbs nucleolar function and ribosome biogenesis, remodels the endolysosomal system toward enhanced uptake but reduced degradative capacity, impairs mitochondrial translation and respiration, and induces pronounced metabolic rewiring together with replication-licensing defects. These convergent stresses activate p53-associated checkpoints and apoptotic priming, resulting in a predominantly cytostatic phenotype characterized by reduced proliferation, metabolic adaptation, and increased vulnerability to secondary stress (Figures 9; S30–S52).

This integrated mechanism expands the established view of Ir–Cp^x^ anticancer agents as primarily mitochondria-targeting ROS inducers.^58,101,102^ In particular, the combined occurrence of endolysosomal remodeling, nucleolar stress, and mitochondrial dysfunction suggests a broader organelle-centered pharmacology for half-sandwich Ir complex **3** and highlights ribosome biogenesis as a potentially exploitable vulnerability in A549 cells.^103–110^

### In Vivo Anticancer Effect Evaluation

As a final step in the evaluation of the antitumor potential of **3**, which showed the most promising activity in the preceding in vitro assays and was therefore selected for detailed proteomic analysis, **3** was tested in an in vivo tumor model.

Under the applied dosing schedule, both **3** and CDDP produced only a modest reduction in tumor burden (Figure 10). Therefore, these results should be interpreted cautiously and viewed primarily as a preliminary proof of concept that **3** is biologically active in vivo, rather than as strong validation of antitumor efficacy. Mice treated with **3** lost less body weight than mice treated with CDDP, suggesting a lower acute systemic burden under the tested conditions. No overt adverse effects, such as hemorrhage or diarrhea, were observed during the experiment. In line with previous reports that some Ir–Cp^x^ compounds may be better tolerated than cisplatin in vivo,^24,55^ the present findings support further investigation of **3**; however, this apparent tolerability advantage should not be overinterpreted as evidence of therapeutic benefit in the absence of a more pronounced antitumor effect. The higher Ir levels detected in kidney tissue (Figure 10E) are consistent with substantial renal handling of this compound, but the present experiment does not establish whether this pharmacokinetic behavior is sufficient to support therapeutically meaningful tumor exposure.

**Figure 10:**
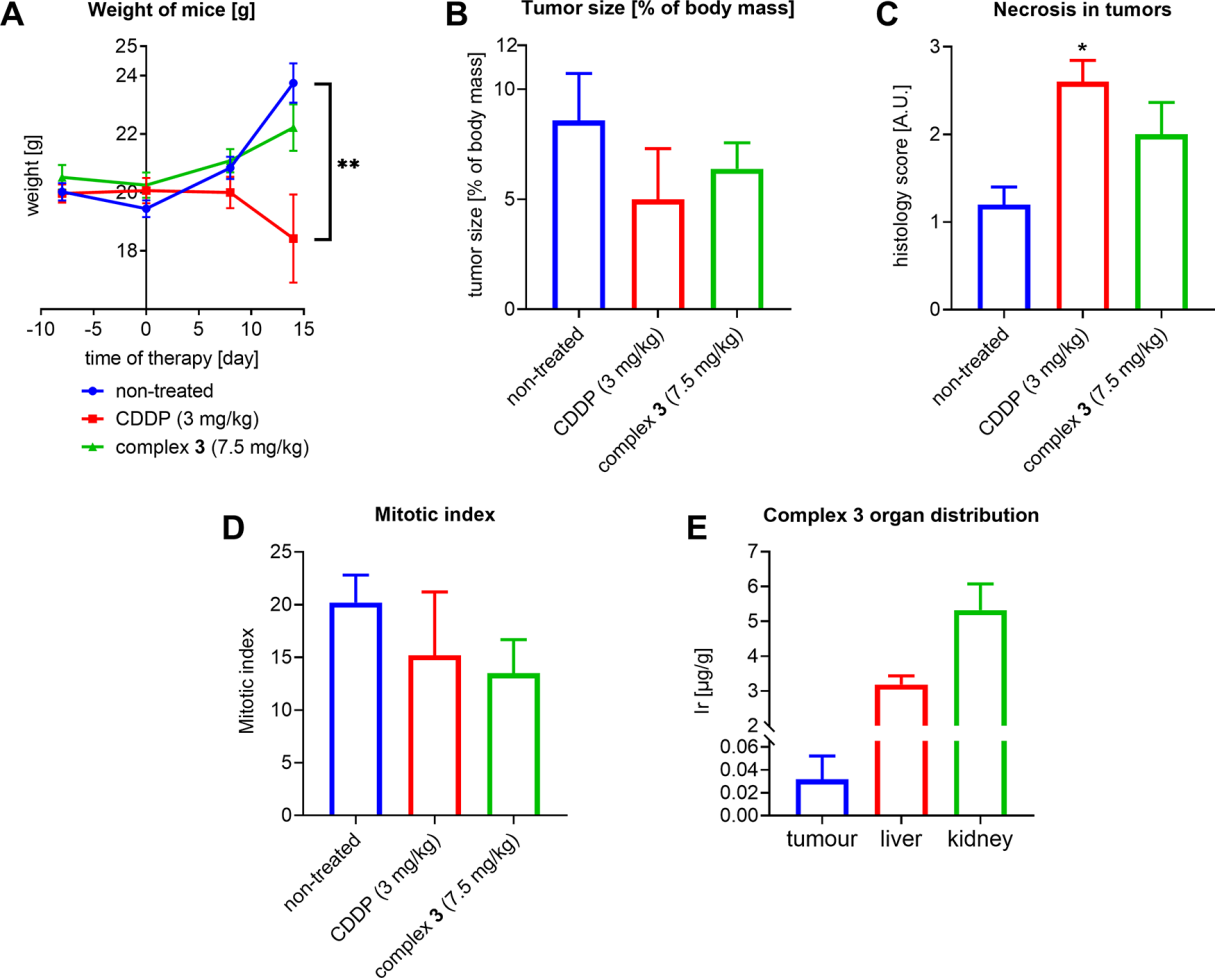
Clinical and histological parameters of the performed in vivo study. Mice bore subcutaneous tumors induced by injection of LL-2/Luc2 cells (day −7). When tumors were formed (day 0), they were started treating either by CDDP (3 mg/kg) or **3** (7.5 mg/kg). After 14 days of treatment, mice were sacrificed and tumor samples were analyzed. (A) Body weight of mice during the experiment. (B) Tumor weight is represented as a percentage of total body weight. (C) Histological evaluation of necrosis in tumors. (D) Analysis of mitotic index in tumor samples. (E) Organ distribution of Ir atoms. The data are shown as the mean ± SEM; * indicates statistical significance (*p* < 0.05) compared with the nontreated group, ** indicates statistical significance (*p* < 0.01) compared with the nontreated group.

Several Ir–Cp^x^ compounds have been studied for their in vivo anticancer activity, which was comparable or better than for conventional Pt-based drugs involved in the studies as reference drugs. For example, dinuclear Ir–Cp^x^ thiosemicarbazone-based compounds showed greater tumor suppression than cisplatin in an A549 lung cancer xenograft.^111^ Some advanced designs of Ir–Cp^x^ compounds were also studied for their in vivo activity - for example, a complex with a biphenyl-extended Cp^x^ ligand tested in vivo in CT26 colon carcinoma mice (3 and 5 mg/kg) inhibited final tumor volume and weight by 31 and 62%, respectively.^24^ Compared with these reports, the activity of **3** observed in the present study appears more limited. Accordingly, the current in vivo data place **3** among biologically active, but not yet strongly efficacious, members of this compound class under the tested conditions.

Generally said, a key mode of action for Ir–Cp^x^ compounds (such as **3**) is the induction of apoptosis in tumor cells.^5,8,15^ Mice with induced tumors treated by **3** showed evidence of caspase 3 (Cas-3) activation, indicating induction of apoptosis similar to cisplatin’s effects (Figures 11 and S53). Indeed, mechanistic studies show that Ir–Cp^x^ compounds trigger the mitochondrial (intrinsic) apoptotic pathway, leading to caspase activation.^24,34^ Nevertheless, these mechanistic changes should be interpreted in the context of the only modest overall reduction in tumor burden. Similarly, the lower mitotic index observed after treatment with **3** (Figure 10D) supports intratumoral biological activity, but the unchanged *K*_i_-67 staining and the absence of marked tumor regression indicate that this activity was insufficient, under the applied conditions, to produce a robust therapeutic response. The lower necrosis score relative to CDDP may reflect differences in tissue response and/or tolerability, but it should not be interpreted as evidence of superior antitumor efficacy.

**Figure 11:**
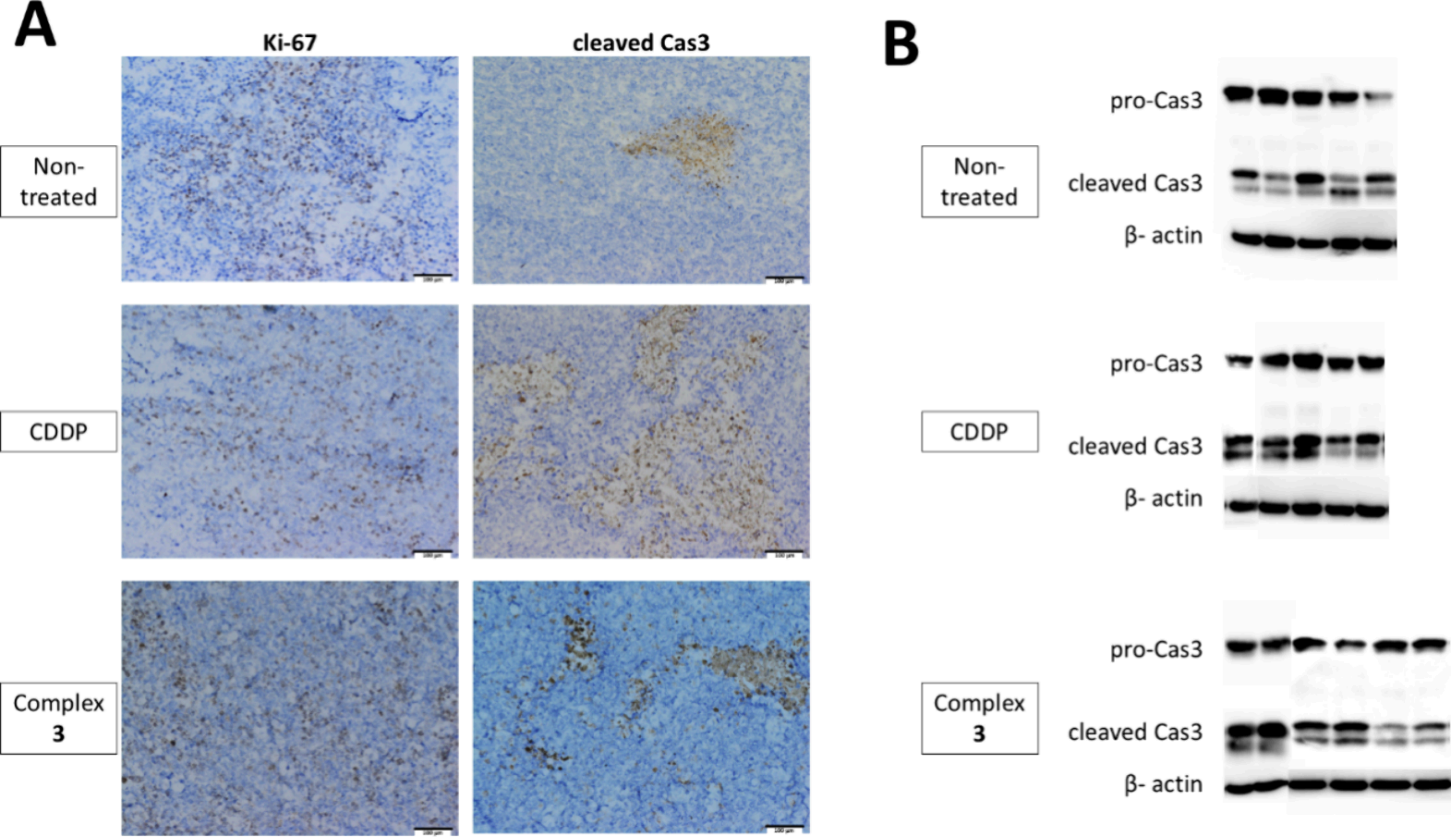
Immunoanalysis of cancer samples. (A) Tissue samples for immunohistochemistry analysis were cut to a thickness of 5–10 μm. Antibodies anti-*K*_i_67 and anti-Cleaved Caspase 3 were applied on processed slides. Then, samples were stained with Liquid DAB+ substrate chromogen system. Finally, the slides were lightly counterstained with Mayer hematoxylin. The black bar represents 100 μm. Magnification 10×. (B) Neoplasma samples were homogenized in RIPA buffer for Western blot analysis and incubated with primary antibodies. After washing, membranes were incubated with HRP-conjugated secondary antibodies. Protein bands were visualized using enhanced chemiluminescence (ECL) detection reagents.

This phenomenon can be explained by MoA of **3**. Tumor cells typically exhibit hyperpolarized mitochondria (more negative Δψ_m_),^112^ leading to a stronger accumulation of cationic Ir compounds compared to normal tissues. However, the present in vivo study does not directly demonstrate selective tumor targeting or sparing of normal tissues. Therefore, the smaller loss of body weight should be regarded as a preliminary tolerability signal rather than as proof of a favorable therapeutic index. Proteomic and GO analyses indicate that the mode of action of **3** is predominantly cytostatic, with suppression of replication licensing and initiation together with attenuation of the CDK axis. This mechanistic profile is consistent with slowed tumor growth rather than frank tumor regression and may help explain why the in vivo effect remained limited under the tested exposure conditions.

This persistence reflects the metabolic plasticity of tumors.^113^ Inhibition of oxidative phosphorylation can be compensated by a shift toward glycolysis (the Warburg effect), allowing cells to remain viable, particularly when the p53/DDR pathway is compromised. In vivo, part of the compound binds to plasma proteins and erythrocytes, while another fraction is rapidly sequestered in the liver and kidneys. As a result, only a limited free fraction reaches the tumor, and intratumoral penetration is further hampered by high interstitial pressure and poor perfusion. The effective concentration may therefore remain below the threshold required for tumor regression, while still being low enough to avoid systemic toxicity.

In addition, tumor cells often harbor genetic or functional suppression of the p53/DDR axis,^114^ which biases the response toward survival rather than apoptosis. Consequently, the “p53 arrest/priming” profile of **3** is likely weaker in vivo tumors than in A549 cells. Consistently, serine/threonine-protein kinase 11-interacting protein (LKB1; p = 3.76 × 10^–3^) was reduced, which may further facilitate tumor survival under metabolic stress.

Taken together, the present in vivo findings suggest that **3** has measurable but limited antitumor activity under the applied conditions, accompanied by apparently lower acute systemic toxicity than CDDP as assessed mainly by body-weight changes. Thus, the current in vivo data are best interpreted as a preliminary proof of concept rather than as strong validation of efficacy. Further work will be required to determine whether the antitumor effect of **3** can be improved by dose and schedule optimization, enhanced tumor delivery, or rational combination strategies.

## Conclusions

Six new half-sandwich Ir–Cp^x^ compounds with the general formula [Ir­(η^5^-Cp^x^)­Cl­(L1–3)]­PF_6_ (**1**–**6**) were synthesized, combining two cyclopentadienyl derivatives (Cp*, Cp^ph^) with three phosphinoalkylamines (L1–3). Among them, **3** displayed the most potent antiproliferative potential, particularly in cisplatin-resistant MOR/CPR lung carcinoma cells, where it outperformed its Cp^ph^ analogue **6** and cisplatin (CDDP). Both **3** and **6** exerted evident cytotoxic activity with MoA distinct from that of classical platinum drugs. From a translational perspective, compound **3** should currently be viewed as a mechanistically interesting lead for cisplatin-resistant disease rather than as a selectively tumor-targeted candidate, because its activity in MOR/CPR cells is accompanied by comparable cytotoxicity toward human PBMCs, indicating no meaningful in vitro safety margin with respect to immune cells. This finding weakens any broad claim of cancer selectivity and raises concern about possible leukocyte/immunological toxicity, which is highly relevant for preclinical progression because unintended immunosuppression is a recognized nonclinical safety issue and in vitro leukotoxicity is considered an important warning signal in immunotoxicity assessment (EMA guideline ICH S8).^115^ Accordingly, further development of **3** should focus on mechanistic validation and medicinal-chemistry optimization aimed at retaining activity in platinum-resistant tumor models while reducing PBMC toxicity, followed by dedicated immunotoxicity evaluation within a weight-of-evidence preclinical framework (EMA guideline ICH S8).^115^

Detailed mechanistic studies, integrating stress-related gene expression and global proteomic profiling, revealed that **3** triggers a rapid integrated stress response encompassing endoplasmic reticulum (ER) stress (DDIT3/CHOP up-regulation) and oxidative stress (HMOX1, ATF3 induction), culminating in G_1_ cell-cycle arrest. The proteomic data further established that **3** primarily targets ribosome biogenesis and mitochondrial translation, causing depletion of mitoribosomal proteins and respiratory-chain subunits while inducing nucleolar stress. This dual stress leads to a translational shutdown, inhibition of oxidative phosphorylation, and a compensatory metabolic shift toward glycolysis. In addition, remodeling of the endolysosomal system - manifested as enhanced vesicular uptake and reduced degradative capacity - likely contributes to intracellular retention and prolonged organellar stress. Collectively, proteomic signatures define a multifaceted, non-DNA–based mechanism in which **3** perturbs nucleolar, mitochondrial, and lysosomal pathways, producing a predominantly cytostatic phenotype with apoptotic priming. In vivo, **3** produced only a modest reduction in tumor growth under the applied regimen, although this was associated with caspase 3 activation in tumor tissue and lower body-weight loss than CDDP. These findings support in vivo biological activity and justify further optimization, but they do not yet constitute strong evidence of therapeutic efficacy.

Overall, the present study identifies **3** as a mechanistically distinctive prototype of half-sandwich Ir­(III) compounds with a multiorganelle mode of action. Through integrated gene-expression and proteomic analyses, **3** was shown to perturb nucleolar, mitochondrial, and endolysosomal pathways, collectively inducing a predominantly cytostatic phenotype characterized by translational shutdown, metabolic reprogramming, and apoptotic priming. In tumor-bearing mice, **3** showed preliminary in vivo proof of concept with limited tumor-growth suppression and apparently improved tolerability relative to CDDP under the tested conditions. Accordingly, the current data support further mechanistic and pharmacological development of **3**, but not yet firm conclusions regarding its therapeutic relevance or a favorable therapeutic index. However, by coupling organelle-specific stress induction with cancer-cell targeting, this work establishes a framework for the rational design of next-generation nonplatinum metallodrugs capable of overcoming resistance through coordinated disruption of cancer cell homeostasis.

## Experimental Section

### Materials

Chemicals (IrCl_3_·*x*H_2_O (99%), 2,3,4,5-tetramethyl-2-cyclopent-2-en-1-one (95%; mixture of *cis*- and *trans*-), phenylmagnesium bromide (3.0 M solution in diethyl ether), 1,2,3,4,5-pentamethylcyclopenta-1,3-diene (95%), 2-(diphenylphosphanyl)­ethanamine (≥95%), 3-(diphenylphosphanyl)­propan-1-amine (≥90%), 2-chloroethylamine hydrochloride (97%), triethylamine (≥99%), trimethylsilyl chloride (99%), di-*tert*-butylphosphine (98%), *n*-butyllithium (1.6 M solution in hexanes), hydrochloric acid (36%), sulfuric acid (98%), sodium hydroxide (powder), magnesium sulfate (>99.5%), ammonium hexafluorophosphate (≥95%), phosphate-buffered saline (PBS; powder), l-glutathione reduced (GSH; ≥98.0%), β-nicotinamide adenine dinucleotide reduced, disodium salt (97%), ascorbic acid (98%)), nitric acid, 70%, purified by redistillation, ≥99.999% trace metals basis, hydrogen peroxide solution (30% (w/w) puriss. p.a.), solvents (THF, MeOH, *n*-hexane, DCM, diethyl ether) and deuterated solvents (CDCl_3_, DMF-*d*_7_, D_2_O) were purchased from Merck (Prague, Czech Republic), VWR International (Stříbrná Skalice, Czech Republic) and Litolab (Chudobín, Czech Republic) and they were used as received.

All cell culture media and supplements [fetal bovine serum (FBS), l-glutamine, antibiotics], collagenase I, and Histopaque 1077 were purchased from Merck (Darmstadt, Germany). A Cell Counting Kit 8 (CCK-8) was obtained from Abcam (Cambridge, UK). Vitrogel was obtained from InvivoGen (San Diego, CA, USA). A Live/Dead Cell Imaging Kit and LIVE/DEAD Fixable Violet Dead Cell Stain Kit were obtained from Invitrogen (Eugene, OR, USA). An ApoflowEx FITC kit was produced by Exbio (Prague, Czech Republic). Cytotoxicity Detection Kit^PLUS^ and Universal Probe Library probes were obtained from Roche (Mannheim, Germany). TaqMan Gene Expression Assays were produced by Thermo Fisher Scientific (Waltham, MA, USA); the Kapa Probe Fast One-Step reaction mixture was purchased from Kapa Biosystems Pty (Cape Town, South Africa); and reference gene qPCR assays were obtained from Generi Biotech (Hradec Králové, Czech Republic).

Organic proligands 2-(di-*tert*-butylphosphanyl)­ethanamine and (2,3,4,5-tetramethylcyclopenta-2,4-dien-1-yl)­benzene (HCp^ph^) was prepared as reported previously.^116,117^ The starting dinuclear Ir­(III) complexes [Ir­(μ-Cl)­(η^5^-Cp*)­Cl]_2_ and [Ir­(μ-Cl)­(η^5^-Cp^ph^)­Cl]_2_ were formerly reported in the literature,^35,118^ and they were prepared using the microwave reactor Monowave 300 (Anton Paar).^116,119^

### Synthesis

The [Ir­(μ-Cl)­(η^5^-Cp*)­Cl]_2_ or [Ir­(μ-Cl)­(η^5^-Cp^ph^)­Cl]_2_ (0.1 mmol) complexes were suspended in MeOH (2 mL) in a microwave reaction vial, and 0.25 mmol of the appropriate aminophosphine (L1–L3) was slowly added (under a N_2_ atmosphere). The reaction mixture was heated in a microwave reactor (1 min, 100 °C, N_2_ atmosphere), left to cool to ambient temperature and centrifuged (6000 rpm, 1 min), leading to clear yellow solutions of [Ir­(η^5^-Cp^x^)­(L1–3)­Cl]Cl (intermediates 1*–6*). After that, NH_4_PF_6_ (0.50 mmol) was added to these solutions, and these mixtures were stirred (r.t., 5 min) followed by the evaporation of MeOH (with nitrogen gas). The obtained solid was dissolved in DCM (ca. 2–3 mL), and these DCM solutions were washed with water (3 × 1 mL) and dried with MgSO_4_ for 5 min. The solutions were centrifuged (6000 rpm, 1 min), concentrated (to ca. 0.5 mL) and diluted with an excess of *n*-hexane until a yellow solid formed. Solid products (compounds **1**–**6**) were collected by centrifugation, washed with *n*-hexane (1 mL) and dried at 40 °C (overnight). The yields reached 40–65% (calculated for the starting dimers). Compounds are stable at r.t. and do not require special storage conditions.

Attempts to isolate chloride salts 1*–6* were not performed. Only 3* was isolated from a mother liquor in MeOH as crystals suitable for a single-crystal X-ray analysis.

#### [Ir­(η^5^-Cp*)­Cl­(L1)]­PF_6_ (**1**)

CHN calc. (found) for C_20_H_39_NClF_6_P_2_Ir: C, 34.5 (34.4); H, 5.6 (5.7); N, 2.0 (1.9) %. Mass spec. (ESI^+^, *m*/*z*): 552.0 (calc. 552.2 for [IrCl­(Cp*)­(L1)]^+^; 45% RI), 516.3 (calc. 516.2 for {[Ir­(Cp*)­(L1)]–H}^+^; 100% RI). ^1^H NMR (CDCl_3_, 300 K): δ 4.87 (bs, 1H, −N*H*_2_), 3.98 (bs, 1H, −N*H*_2_), 3.05 (bs, 1H, −C*H*_2_), 2.94 (m, 1H, −C*H*_2_), 2.32 (m, 1H, −C*H*_2_), 1.98 (m, 1H, −C*H*_2_), 1.73 (s, 15H, −C*H*_3_), 1.39 (d, *J* = 13.8 Hz, 9H, −C­(C*H*_3_)_3_), 1.32 (d, *J* = 13.8 Hz, 9H, −C­(C*H*_3_)_3_) ppm. ^13^C NMR (CDCl_3_, 300 K): δ 92.8, 45.6, 43.4, 30.9, 29.5, 25.9, 25.2, 9.7 ppm. ^31^P NMR (CDCl_3_, 300 K): 58.4, −144.0 δ ppm.

#### [Ir­(η^5^-Cp*)­Cl­(L2)]­PF_6_ (**2**)

CHN calc. (found) for C_24_H_31_NClF_6_P_2_Ir: C, 39.1 (38.9); H, 4.2 (4.4); N, 1.9 (1.8) %. Mass spec. (ESI^+^, *m*/*z*): 592.1 (calc. 592.2 for [IrCl­(Cp*)­(L2)]^+^; 100% RI), 556.3 (calc. 556.2 for {[Ir­(Cp*)­(L2)]–H}^+^; 90% RI). ^1^H NMR (CDCl_3_, 300 K): δ 7.57–7.13 (m, 10H, – C6*H*5), 5.35 (bs, 1H, −N*H*_2_), 3.95 (bs, 1H, −N*H*_2_), 2.72 (bs, 2H, −C*H*_2_), 2.54 (bs, 2H, −C*H*_2_), 1.59 (s, 15H, −C*H*_3_) ppm. ^13^C NMR (CDCl_3_, 300 K): δ 134.2, 131.8, 128.7, 93.4, 45.0, 32.4, 32.2, 8.2 ppm. ^31^P NMR (CDCl_3_, 300 K): δ 31.4, −144.1 ppm.

#### [Ir­(η^5^-Cp*)­Cl­(L3)]­PF_6_ (**3**)

CHN calc. (found) for C_25_H_33_NClF_6_P_2_Ir: C, 40.0 (40.3); H, 4.4 (4.6); N, 1.9 (2.1) %. Mass spec. (ESI^+^, *m*/*z*): 606.2 (calc. 606.2 for [IrCl­(Cp*)­(L3)]^+^; 100% RI), 570.3 (calc. 570.2 for {[Ir­(Cp*)­(L3)]–H}^+^; 50% RI). ^1^H NMR (CDCl_3_, 300 K): δ 7.75–7.46 (m, 10H, −C6*H*5), 4.48 (bs, 1H, −N*H*_2_), 3.68 (bs, 1H, −N*H*_2_), 3.45 (m, 1H, −C*H*_2_), 2.94 (m, 2H, −C*H*_2_), 2.38 (m, 1H, −C*H*_2_), 1.59 (m, −C*H*_2_), 1.46 (s, −C*H*_3_) ppm. ^13^C NMR (CDCl_3_, 300 K): δ 133.9, 132.3, 131.2, 129.3, 128.3, 93.4, 39.2, 24.8, 24.6, 23.2, 8.0 ppm. ^31^P NMR (CDCl_3_, 300 K): δ −6.0, −144.3 ppm.

#### [Ir­(η^5^-Cp^ph^)­Cl­(L1)]­PF_6_ (**4**)

CHN calc. (found) for C_25_H_41_NClF_6_P_2_Ir: C, 39.6 (39.5); H, 5.4 (5.6); N, 1.8 (1.8) %. Mass spec. (ESI^+^, *m*/*z*): 614.0 (calc. 614.2 for [IrCl­(Cp^ph^)­(L1)]^+^; 10% RI), 578.3 (calc. 578.3 for {[Ir­(Cp^ph^)­(L1)]–H}^+^; 100% RI). ^1^H NMR (CDCl_3_, 300 K): δ 7.33–7.49 (m, 5H, −C6*H*5), 5.27 (bs, 1H, −N*H*_2_), 4.02 (bs, 1H, −N*H*_2_), 3.09 (bs, 1H, −C*H*_2_), 2.97 (m, 1H, −C*H*_2_), 2.55 (m, 1H, −C*H*_2_), 2.09 (d, *J* = 1.8 Hz, 3H, −C*H*_3_), 2.03 (m, 1H, −C*H*_2_), 1.88 (d, *J* = 1.8 Hz, 3H, −C*H*_3_), 1.74–1.78 (m, 6H, −C*H*_3_), 1.27 (d, *J* = 13.8 Hz, 9H, −C­(C*H*_3_)_3_), 1.25 (d, *J* = 13.8 Hz, 9H, −C­(C*H*_3_)_3_) ppm. ^31^P NMR (CDCl_3_, 300 K): δ 54.7, −144.4 ppm.

#### [Ir­(η^5^-Cp^ph^)­Cl­(L2)]­PF_6_ (**5**)

CHN calc. (found) for C_29_H_33_NClF_6_P_2_Ir: C, 43.6 (44.0); H, 4.2 (3.9); N, 1.8 (1.8) %. Mass spec. (ESI^+^, *m*/*z*): 654.1 (calc. 654.2 for [IrCl­(Cp^ph^)­(L2)]^+^; 65% RI), 618.2 (calc. 618.2 for {[Ir­(Cp^ph^)­(L2)]–H}^+^; 100% RI). ^1^H NMR (CDCl_3_, 300 K): δ 7.51–7.14 (m, 10H, −C6*H*5), 6.82–7.02 (m, 5H, −C6*H*5), 5.52 (bs, 1H, −N*H*_2_), 4.32 (bs, 1H, −N*H*_2_), 2.77 (bs, 2H, −C*H*_2_), 2.64 (bs, 2H, −C*H*_2_), 2.08 (m, 3H, −C*H*_3_), 1.65–1.51 (m, 9H, −C*H*_3_) ppm. ^31^P NMR (CDCl_3_, 300 K): δ 30.8, −143.8 ppm.

#### [Ir­(η^5^-Cp^ph^)­Cl­(L3)]­PF_6_ (**6**)

CHN calc. (found) for C_30_H_35_NClF_6_P_2_Ir: C, 44.3 (44.2); H, 4.3 (4.2); N, 1.7 (1.9) %. Mass spec. (ESI^+^, *m*/*z*): 668.1 (calc. 668.2 for [IrCl­(Cp^ph^)­(L3)]^+^; 100% RI), 632.2 (calc. 632.2 for {[Ir­(Cp^ph^)­(L3)]–H}^+^; 90% RI). ^1^H NMR (CDCl_3_, 300 K): δ 7.75–7.36 (m, 15H, −C6*H*5), 4.51 (bs, 1H, −N*H*_2_), 3.75 (bs, 1H, −N*H*_2_), 3.06 (bs, 1H, −C*H*_2_), 2.97 (bs, 1H, −C*H*_2_), 2.40 (bs, 1H, −C*H*_2_), 2.28 (m, 1H, −C*H*_2_), 1.70 (s, 3H, −C*H*_3_), 1.62 (s, 3H, −C*H*_3_), 1.60 (s, 3H, −C*H*_3_), 1.53 (s, 3H, −C*H*_3_) ppm. ^31^P NMR (CDCl_3_, 300 K): δ −6.6, −144.3 ppm.

### General Methods

ESI-MS was carried out on methanol solutions in a positive ionization mode (ESI^+^); LCQ Fleet ion trap spectrometer (Thermo Scientific; QualBrowser software, version 2.0.7). Elemental analysis was performed by a Flash 2000 CHNS Elemental Analyzer (Thermo Scientific). RP-HPLC was performed on a UHPLC device (Dionex/Thermo Fisher Scientific) equipped with a Phenomenex Luna LC column (C18 stationary phase; 3 μm particle size, 100 Å pore size, 125 × 4 mm). A mixture of 0.1% TFA in H_2_O (A) and ACN (B) was used as the mobile phase at gradients of 10% B (*t* = 0 min), 60% B (*t* = 15 min), 60% B (*t* = 20 min), followed by 10 min equilibration (0.6 mL min^–1^ flow rate). ^1^H, ^13^C and ^31^P NMR spectroscopy, and ^1^H–^1^H COSY, ^1^H–^13^C HMQC and ^1^H–^13^C HMBC correlation experiments were recorded using CDCl_3_ solutions at 298 K on a Varian-400 spectrometer (^1^H, 400 MHz; ^13^C, 101 MHz; ^31^P, 162 MHz). ^1^H and ^13^C NMR spectra were calibrated against the residual signals of the solvent used (^1^H at 7.26 ppm and ^13^C at 77.2 ppm). ^31^P NMR spectra were calibrated externally against the signals of 85% H_3_PO_4_ in D_2_O (δ = 0.0 ppm). The splitting of proton resonances in the reported ^1^H spectra is defined as s = singlet, d = doublet, t = triplet, m = multiplet and bs = broad signal. A Jasco FT/IR-4700 spectrometer was used for the collection of FTIR spectra in the range of 400–4000 cm^–1^ by using the attenuated total reflection (ATR) technique on a diamond plate.

### X-ray Crystallography

The data were collected using an XtaLAB Synergy-I diffractometer with a HyPix3000 hybrid pixel array detector and a microfocused PhotonJet-I X-ray source (Cu Kα). All the crystal structures were solved using the SHELXT program^120^ and refined by the full matrix least-squares procedure with Olex2.refine in OLEX2 (version 1.5).^121^ Multiscan absorption corrections were applied using the program CrysAlisPro 1.171.40.82a.^122^ The molecular structures and packing diagram were drawn with MERCURY.^123^

Nonroutine aspects of refinement: To improve modeling of the charge density in the vicinity of the Ir, P (only on L3) and Cl atoms, anharmonic refinement of their displacements was used. The aliphatic part of the L3 ligand exhibited positional disorders (ratio of occupation factors = 0.81:0.19). The PF_6_^–^ anion exhibits rotational disorder, which was modeled as disorder over two positions with a ratio of occupation factors = 0.72:0.28. It must be noted that this approach did not lead to fully satisfactory modeling of the electron density.

### Stability Studies

The stability of all compounds (10 μM final concentration) in water-containing media was studied by UV–vis spectroscopy in 5% DMF/95% PBS in H_2_O (*note:* the solubility of the compounds in water is very low and insufficient for UV–vis spectroscopy. Therefore, DMF was added, in which the compounds are highly soluble and which increases their solubility in water-containing mixtures. DMF was selected with respect to biological experiments, as it was used to dissolve the compounds during the preparation of stock solutions.). UV–vis spectra were recorded on the fresh solutions at various time points (*t* = 0–24 h).

Stability in DMEM cell culture media were performed by RP-HPLC for all the compounds (50 μM final concentration in 1 mL). The samples were prepared in 10% DMF mixtures either with DMEM (without serum) or with PBS in water (control experiments). The mixtures were monitored by analytical RP-HPLC using the Acclaim column (Dionex; C18 stationary phase, 5 μm particle size, 120 Å pore size, 50 mm column length × 2.1 mm internal diameter). The mixture of acetonitrile (MeCN; A) and 0.1% TFA in H_2_O (B) was used as the mobile phase at the gradients of 0% A (*t* = 2 min for equilibration), 0 to 100% A (*t* = 10 min), 100% A (*t* = 2 min), 100 to 0% A (*t* = 2 min) and 0% A (*t* = 2 min) over a 18 min period (1.0 mL/min flow rate). Stock solutions were kept at r.t. between the individual experiments injected at different time points (0–24 h).

The stability of **3** and **6** was also checked by ^1^H NMR in 10% DMF-*d*_7_/90% PBS in D_2_O. The appropriate amount for 600 μL of 1 mM solutions was dissolved in DMF-*d*_7_ (60 μL), and PBS in D_2_O (540 μL) was added. ^1^H NMR spectra were recorded on the fresh solutions at various time points (*t* = 0–24 h). The obtained spectra were calibrated against the residual signal of D_2_O (δ_H_ 4.75 ppm).

The stability of **3** and **6** (1 mM final concentration) was studied in the presence of selected intracellular biomolecules (i.e., reduced glutathione (GSH) and reduced nicotinamide adenine dinucleotide (NADH)) in 10% DMF-*d*_7_/90% PBS in D_2_O (v/v; 140 mM concentration of chloride ions). The molar ratio of the compounds to biomolecules was 1:5 in all the cases. In the case of **3** (1 mM final concentration), additional experiments were performed in the same medium supplemented with a mixture of NADH and GSH (5 molar equiv for both biomolecules) or with NADH, GSH and ascorbate (5 molar equiv for each biomolecule).

Similar ^1^H NMR experiment was performed for the mixtures of **3** and **6** (1 mM final concentration) with 5 molar equiv of His in 10% DMF-*d*_7_/90% PBS in D_2_O (v/v; 5.5 mM concentration of chloride ions).

### Cell Culture

The cytotoxic effects of the tested compounds were evaluated on the human leukemia cell line THP-1, the cervical cell line HeLa, the synovial cell line SW982, the lung cell lines A549 and MOR, and the CDDP-resistant MOR/CPR (all obtained from ECACC, Salisbury, UK). THP-1, SW982, and MOR cells were cultured in RPMI 1640 medium; HeLa and A549 cells, in DMEM High glucose medium; both media were supplemented with 10% fetal bovine serum (FBS) and antibiotics (100 U/mL penicillin and 100 μg/mL streptomycin) (both from Merck), and antimicrobial agent Normocin. The cells were kept at 37 °C in a humidified atmosphere containing 5% CO_2_. MOR/CPR cells were cultivated in the presence of 1 μg/mL CDDP, as recommended by the supplier. Cells were usually passaged twice a week, and their viability was checked by vital staining with trypan blue.

Primary porcine chondrocytes (PPC) were used as noncancer cells. They were isolated from the cartilage tissue obtained from the porcine elbow joint from slaughtered pigs in a local slaughterhouse, as we described previously.^124^ Briefly, cartilage was cut by a scalpel, obtained pieces were immersed in a solution of collagenase I (6 mg/mL) and incubated at 37 °C for 2–3 h. The enzyme was subsequently inactivated by adding DMEM/F12 medium supplemented with 10% FBS and a 1% penicillin/streptomycin mixture. The cell suspension was passed through a 70 μm nylon membrane and centrifuged for 5 min at 150 × *g*. The cell pellets were resuspended in fresh DMEM/F12 medium and split into cultivation plates coated with collagen I (Corning; Kennebunk, ME, USA) at a density of 5 × 10^3^ cells/cm^2^. The cells were incubated for 9 days at 37 °C in a humidified atmosphere with 5% CO_2_. After this period, the medium was exchanged, and the cells were ready for further experiments.

Human peripheral blood mononuclear cells (PBMCs) were isolated from the buffy coat prepared from the whole blood of healthy volunteers at the Department of Transfusion & Tissue Medicine of the University Hospital Brno. The buffy coat was mixed with PBS (NaCl, 137 mM; KCl, 2.7 mM; Na_2_HPO_4,_ 10 mM; KH_2_PO_4,_ 1.8 mM; pH 7.4) at a ratio of 1:1 and subsequently transferred into a cuvette containing Histopaque 1077 with half the volume of the buffy coat/PBS mixture. After centrifugation (at 500 × *g*/30 min/r.t.), the layer containing the PBMCs was aspirated, transferred to a new cuvette, and washed twice with cold PBS (followed by centrifugation at 500 × *g*/10 min/4 °C). The washed cells were resuspended in complete RPMI 1640 medium [containing 10% FBS, antibiotics (100 U/mL penicillin and 100 μg/mL streptomycin), and Normocin] and counted.

### In Vitro Cytotoxicity Testing

The effect of the tested compounds on cell viability was evaluated using a Cell Counting Kit 8 (CCK8) according to the manufacturer’s manual. Floating THP-1 cells and PBMCs were seeded at a concentration of 5 × 10^4^ cells/well in a 96-well plate, and the tested compounds dissolved in DMF (the concentration of DMF did not exceed 0.1% v/v in the presence of cells) were added after a 2 h recovery span. CDDP was used as the positive control. Adherent HeLa, SW982, A549, MOR, and MOR/CPR cells were seeded at a density of 1 × 10^4^ cells/well and allowed to adhere overnight. The next day, the cultivation medium was changed (MOR/CPR cell obtained medium without CDDP), and the tested compounds were added in the same manner as in the case of the floating cells. Primary chondrocytes were treated with the indicated material after medium exchange, as described above. Cell viability was measured 72 h later, and the IC_50_ values (the concentrations of the tested compounds that caused a 50% decrease of metabolic active cells in comparison with the number of untreated cells) were calculated according to four-parameter logistic (4PL) analysis, excluding outstanding values (ROUT algorithms, *Q* = 5%) in Prism 7.05 software (GraphPad Software, Inc., San Diego, CA, USA).

### 3D Cellular Cancer In Vitro Model

To evaluate the anticancer potential of the tested Ir compounds in a 3D cell model, which is more closely related to real cancer than 2D models,^125^ spheroids formed from A549 cells were used. A549 cells resuspended in serum-free DMEM were seeded at a density of 16 × 10^3^ cells/well into a 96-well U-shaped bottom microtiter plate with a low cell binding surface (Thermo Scientific, Roskilde, Denmark). After 4 days, the medium was exchanged for medium containing 10% FBS, and the spheroids were cultivated for another 3 days. Then, the grown spheroids (diameter of 0.5–1.0 mm) were transferred to a 96-well plate with a flat bottom, which was covered with Vitrogel matrix, forming a wide U-shaped bed. During the transfer, the medium was exchanged. **3** and **6** and CDDP at concentrations corresponding to their IC_50_ values obtained from 2D experiments (i.e., 8 μM for **3**, 9 μM for **6**, and 10 μM for CDDP) were added after 2 h of acclimatization to the spheroids in a CO_2_ incubator. After 3 days of incubation, the spheroids were stained with a Live/Dead Cell Imaging Kit containing calcein AM and BOBO-3 iodide dyes to distinguish live and dead cells. After 30 min of incubation in the dark at r.t., the shape of the spheroids and the amount and distribution of dye were analyzed via confocal microscopy (Leica TCS SP 8). At least 3 spheroids were analyzed for each compound.

### Flow Cytometry Analysis

The protocol was described previously.^126^ Briefly, MOR/CPR cells were seeded into a 6-well cultivation plate at a concentration of 3 × 10^5^ cells/well and allowed to adhere overnight. The cultivation medium was changed to CDDP-free medium for 2 h. The tested compounds and CDDP as a control were subsequently added at concentrations corresponding to one-half of their IC_50_ (i.e., 1.5 μM for **3**, 3.0 μM for **6**) and 10.0 μM for CDDP (as a positive control) for 72 h. Flow cytometry was used to evaluate the number of cells in the particular phases of the cell cycle and for the subsequent analysis of cell death. Control and treated cells were washed with PBS, collected by trypsin, and centrifuged at 200 × *g* for 5 min at 4 °C. For cell cycle analysis, the cells were washed in cold PBS and fixed with chilled −20 °C ethanol (70%; v/v) by low-speed vortexing. Annexin V-FITC and viability staining (propidium iodide, PI) were used for the detection of apoptotic cells. The treated cells were stained with an ApoflowEx FITC Kit at a dilution of 1:20 and a LIVE/DEAD Fixable Violet Dead Cell Stain Kit at a dilution of 1:1000 for 20 min. Finally, the cells were analyzed by an Amnis CellStream flow cytometer (Luminex, USA), and the data were evaluated by CellStream Analysis 1.2.55 software. A minimum of 15,000 cells excluding doublets and debris were subjected to analysis. The final dot plots were divided into four quadrants. Live cells (PI^–^/Annexin V^–^; low left quadrant; LL), early apoptotic cells (PI^–^/Annexin V^+^; low right quadrant; LR), late apoptotic cells (PI^+^/Annexin V^+^; upper right quadrant; UR), and necrotic/dead cells (PI^+^/Annexin V^–^; upper left quadrant; UL) were distinguished after double staining. Experiments were performed in duplicate and in three independent repetitions.

### Western Blot Analysis of MOR/CPR Cell Line

MOR/CPR cells were seeded into a 6-well cultivation plate at a concentration of 3 × 10^5^ cells/well and allowed to adhere overnight. The cultivation medium was replaced with fresh CDDP-free medium, and the cells were treated with compound **3** (1.5 and 3 μM), compound **6** (3 and 6 μM), CDDP (10 μM; positive control) or DMF (negative control) for 24 h. Cells were washed with cold PBS and lysed in RIPA buffer (50 mM Tris-HCl pH 7.4, 150 mM NaCl, 1% NP-40, 0.5% sodium deoxycholate, 0.1% SDS) supplemented with HALT protease and phosphatase inhibitors (ThermoFisher Scientific, USA). Lysates were incubated on ice for 30 min and centrifuged at 12,000*g* for 15 min at 4 °C. Protein concentration was determined using the bicinchoninic acid (BCA) assay (Thermo Fisher Scientific). Equal amounts of proteins (15 μg) were mixed with Laemmli sample buffer, boiled for 5 min at 95 °C, separated by 10% SDS-PAGE, and transferred onto PVDF membranes (Millipore). Membranes were blocked with 5% nonfat milk in TBS-T buffer and incubated with primary antibodies against caspase-3 (#9662), caspase-8 (#9746), cleaved caspase-9 (#9505), PARP (#9542) (all from Cell Signaling Technology), and β-actin (#A1978, Merck) overnight at 4 °C. After washing, membranes were incubated with HRP-conjugated secondary antibodies (antimouse IgG #7076 and antirabbit IgG #7074, Cell Signaling Technology) for 1 h at r.t. Protein bands were detected using enhanced chemiluminescence reagents (ECL Prime Western Blotting Detection Reagent, GE Healthcare, and Femto ECL, Vazyme). Experiments were performed three times independently and representative blots are shown.

### Determination of Mitochondrial Membrane Potential

Mitochondrial membrane potential (ΔΨ_m_) changes following treatment with **3** and **6**, cisplatin (CDDP), and dimethylformamide (DMF) for 24 h were assessed using the cationic dye tetramethylrhodamine methyl ester (TMRM), as we described previously.^38^ A549 cells were seeded in 6-well culture plates at a density of 3 × 10^5^ cells per well and allowed to adhere overnight. Then, the medium was replaced, and cells were treated by tested compounds at IC_50_ concentrations (i.e., 8.0 μM for **3**, 9.0 μM for **6**, and 10 μM for CDDP). Following 24 h of treatment, cells were harvested and resuspended in culture medium containing TMRM at a final concentration of 100 nM. Cells were incubated with TMRM at 37 °C for 30 min in the dark to facilitate dye accumulation in polarized mitochondria. After incubation, cells were centrifuged at 500*g* for 5 min at room temperature, washed once with prewarmed PBS to remove excess dye, and stained using the LIVE/DEAD Fixable Violet Dead Cell Stain Kit (ThermoFisher Scientific, USA) at a 1:1000 dilution. After a 15 min incubation at 37 °C, the samples were washed, centrifuged at 500*g* for 5 min, and resuspended in PBS for analysis. Flow cytometry was performed using the Amnis CellStream flow cytometer (Luminex, USA). Carbonyl cyanide chlorophenylhydrazone (cccp, 50 μM) served as a control for impaired mitochondrial membrane potential. Unstained cells and cells treated with carbocyanine iodide were used as controls for proper flow cytometry setup. A minimum of 1.5 × 10^5^ live cells, excluding doublets and debris, were analyzed. TMRM fluorescence was detected with appropriate excitation and emission settings (Ex/Em: 548/574 nm). Cells with diminished TMRM fluorescence were gated as depolarized, indicative of ΔΨ_m_ loss, characteristic of early apoptosis. Data were analyzed using CellStream Analysis 1.2.55 software. All experiments were performed in duplicate across three independent trials.

### Stress-Related Gene Expression

Changes in the gene expression of cellular stress markers were determined by qRT–PCR in A549 and HepaRG cell lines. Human liver HepaRG (Biopredic, Rennes, France) cells were differentiated in 24-well plates as described previously,^127^ with minor changes. After differentiation, the cells were exposed to **3**, **6**, or CDDP in medium without the differentiating agent (2% dimethyl sulfoxide) for 5 or 24 h. Cytotoxicity was tested after 24 h with a Cytotoxicity Detection Kit^PLUS^, according to the manufacturer’s instructions. A549 cells were plated at a density of 2 × 10^5^ cells in 24-well plates and treated with **3** and **6** at concentrations corresponding to their 0.5×, 1× and 2× IC_50_ values (i.e., 4.0, 8.0, and 16.0 μM for **3**, and 4.5, 9.0, and 18.0 μM for **6**) for 5 and 24 h. After exposure, both cell lines were washed with PBS and harvested into a lysis buffer from the NucleoSpin RNA II Purification Kit (Macherey-Nagel, Düren, Germany). Total RNA was isolated according to the manufacturer’s instructions and qRT–PCR was performed with primers, Universal Probe Library probes or TaqMan Gene Expression Assays, as described recently,^128^ with minor changes. The amplifications were carried out in 10 μL of Kapa Probe Fast One-Step reaction mixture containing 1 μL of the sample. Reference gene qPCR assays were used for the housekeeping genes human β2-microglobulin (B2M; #3030) and hydroxymethylbilane synthase (HMBS; #3032). The crossing point (*C_t_*) values of these two genes were averaged to serve as a reference, and the changes in relative gene expression were calculated based on the comparative threshold cycle method.^129^

### Proteome Analysis

A549 cells were seeded at 2 × 10^6^ in a 10 cm Petriho dish and allowed to adhere overnight. The cultivation medium was changed to fresh medium and the test compounds were subsequently added at concentrations corresponding to one-half of their IC_50_ (i.e., 4.0 μM for **3**, 4.5 μM for **6**, and 5.0 μM for CDDP) for 24 and 48 h.

A549 cells treated with compounds **3**, **6**, CDDP, or DMF (control) were lysed in 100 μL 10% SDS and 100 μL 100 mM TEAB buffer (pH 8) at 70 °C for 30 min (700 rpm). Lysates were cooled on ice, diluted with 300 μL TEAB, and nucleic acids digested by two additions of 200 U benzonase (15 min each, RT). Proteins were reduced with 15 mM DTT (56 °C, 45 min), alkylated with 30 mM iodoacetamide (RT, dark), quenched with 15 mM DTT (15 min, RT), and precipitated with five volumes of cold acetone (−20 °C) overnight at −28 °C. Pellets were collected by centrifugation (20,000 × *g*, 5 °C, 10 min), washed with acetone, dried, and resuspended in 50 μL 10% sodium deoxycholate. Proteins were redissolved in 450 μL 100 mM TEAB (pH 8) by sonication and digested in solution with 0.5 μg commercial trypsin (SoluTrypsin, Merck, Germany) for 3 h at 37 °C, followed by addition of another 1 μg enzyme and incubation overnight (37 °C, 500 rpm). After digestion, the sodium deoxycholate was removed using the phase-transfer procedure with ethyl acetate.^130^ Peptides were purified on C18 STAGE-Tips according to standardized protocol,^131^ eluted with 60% methanol, vacuum-dried, reconstituted in 30 μL mass spectrometry-grade water, quantified spectrophotometrically (NanoDrop, 205/215 nm), diluted to 300 ng/μL, and acidified with 0.1% formic acid. QC samples were prepared by pooling 5 μL of each sample.

Samples were randomized and analyzed by nanoElute2 coupled online to a timsTOF Pro 2 with captive ESI (Bruker Daltonics, Germany). Peptides were loaded and separated on a PepSep C18 column (10 cm × 75 μm, 1.9 μm particles, Bruker Daltonics, Germany) using a 70 min binary gradient (4% B at 0 min; 8% B at 4 min; 29% B at 45 min; 40% B at 55 min; 65% B at 60–65 min; 95% B at 66–69 min; followed by re-equilibration at 4% B to 70 min) at 300 nL/min at 45 °C. Mobile phase A was water with 0.1% (v/v) formic acid; mobile phase B was acetonitrile with 0.1% (v/v) formic acid. Data were acquired in DDA-PASEF for spectral library generation and DIA-PASEF for quantification. Raw data were processed with FragPipe v22.0 ^132^ using MSFragger v4.3 ^133^ for identification, validated with MSBooster v1.3.9,^134^ Percolator v3.7.1,^135^ and Philosopher v5.1.1 ^136^ against the UniProt human reference proteome (UniProt, reference proteome UP000005640, 81,791 protein sequences, downloaded in June 2024) including common contaminants and reverse sequences for FDR determination (Table S4). Quantitative analysis was performed with DIA-NN v1.8.2.^137^ Complete parameter settings for FragPipe and DIA-NN are provided in the Supplementary Data (Table S5). Statistical analysis was carried out in the R language (version 4.3.3) using the Metabol package [Version v1.0.0], SIMCA software (version 18.0, Sartorius, Germany) and MS Excel. The data processing included a QC-based locally estimated smoothing signal correction (LOESS), normalization to the median of each sample, and log transformation. Volcano plot was used to identify statistically significant differences, with the significance threshold set at α = 0.05 (Figure S52). The enriched clusters of functional annotation Gene Ontology (GO) terms related to the identified proteins were determined using ShinyGO v.0.85.^138^

### In Vivo Verification

The antitumor effect of **3** in comparison with CDDP was evaluated in vivo using a syngeneic murine tumor model with Lewis lung carcinoma cells (LL/2; ATCC code: CRL-1642, USA). All animal experiments were approved by the government authority (ethical permit ID: MZe 2338) and were conducted in agreement with ARRIVE guidelines.

A total of 18 female C57BL/6 mice (6–8 weeks old, average body weight 20 g) were acclimatized for 7 days prior to inoculation. Each mouse was subcutaneously injected into the dorsal region at the level of the lumbar vertebrae with LL/2 cells (1 × 10^6^ cells in 100 μL of physiological saline). Tumor growth was monitored clinically, and tumor dimensions (width, length) were measured with a digital caliper at 2-day intervals throughout the experiment. When tumors were formed, mice were randomly assigned into three groups of six animals each and treated intraperitoneally with compound **3** at a dose of 7.5 mg/kg (10 μmol/kg), CDDP at a dose of 3 mg/kg (10 μmol/kg), or 200 μL of physiological saline (untreated control). In two mice (untreated control: *n* = 1; CDDP group: *n* = 1), no subcutaneous tumors developed, and these animals were excluded from the study.

Body weight was recorded on the day of inoculation (day 0) and on days 7, 11, 15, and 22 postinoculation. Treatments were administered repeatedly at 48 h intervals until day 22 postinoculation (a total of 8 administrations), at which point all mice were humanely sacrificed under anesthesia. At necropsy, subcutaneous tumors were excised, weighed, and measured. Apart from tumors, kidneys and liver were collected for determination of Ir concentration in these organs. Tumor samples were fixed in 10% buffered formalin and processed using standard paraffin histology with hematoxylin and eosin staining. Sections were examined under an Olympus BX 53 optical microscope (Olympus, Japan) equipped with a digital camera (Olympus DP 71, Olympus, Japan).

Tissue reactions (necrosis) were scored as follows: 0 – absent, 1 – mild, 2 – moderate, and 3 – severe. Mitotic figures were counted in 10 high-power fields (2.37 mm^2^).

### Western Blot Analysis of Neoplasia Samples

Tissue samples from neoplastic lesions were collected from mice after in vivo experiments and immediately snap-frozen in liquid nitrogen. For protein extraction, frozen tissue samples were homogenized in RIPA lysis buffer (50 mM Tris-HCl pH 7.4, 150 mM NaCl, 1% nonyl phenoxypolyethoxylethanol (NP-40), 0.5% sodium deoxycholate, 0.1% SDS) supplemented with protease inhibitors complete (Roche) using a mechanical homogenizer. The lysates were incubated on ice for 30 min and centrifuged at 12,000 × *g* for 15 min at 4 °C to remove debris. Supernatants containing total protein were collected and quantified using the BCA protein assay (BCA = bicinchoninic acid; Thermo Fisher Scientific).

40 μg of protein were mixed with Laemmli sample buffer, boiled for 5 min at 95 °C, and separated by SDS-PAGE on 10% polyacrylamide gels. Proteins were transferred onto polyvinylidene fluoride (PVDF) membranes (Millipore) using a semidry transfer system. Membranes were blocked with 5% nonfat dry milk in TBS-T (20 mM Tris-HCl, 150 mM NaCl, 0.1% Tween-20) for 1 h at room temperature and incubated overnight at 4 °C with primary antibodies (Cas3 #9626, Cell Signaling; anti-β-Actin #A1978, Merck) diluted in blocking buffer. After washing, membranes were incubated with HRP-conjugated secondary antibodies (antimouse IgG 7076 and antirabbit IgG 7074, Cell Signaling) for 1 h at r.t. Protein bands were visualized using enhanced chemiluminescence (ECL) detection reagents (ECL Prime Western Blotting Detection Reagent, GE Healthcare UK and Femto ECL, Vazyme) and imaged using a chemiluminescence imaging system.

### Histological and Immunohistochemistry Analysis of Neoplasia Samples

Neoplasia samples were fixed in 10% buffered formalin and processed by standard histological paraffin technique with staining by hematoxylin and eosin. Slides were observed under the optical microscope Olympus BX 53 (Olympus, Japan) with digital output (camera Olympus DP 71, Olympus, Japan). Tissue reactions (necrosis, apoptosis, edema, anisocytosis/anisokaryosis) were graded as 0 - absent, 1 - mild, 2 - moderate, and 3 - severe. The numbers of mitoses were counted in 10 high power fields (2.37 mm^2^).

Immunohistochemistry (IHC) analysis was performed as described previously with minor modifications.^139^ Briefly, tumor samples were embedded in the OCT compound and frozen in precooled *n*-heptane (Penta Chemicals, Czech Republic) placed on dry ice. Then, the tissue samples were cut to a thickness of 5–10 μm on the cryostat (Leica Microsystems, CM 1900, Germany) at a temperature of −20 °C. The cuts of tissue were placed on slides, the sections allowed to dry at r.t., fixed in precooled acetone (Penta Chemicals) at −18 °C for 5 min and stained by IHC method. Slides were rehydrated and endogenous peroxidase was quenched with Dual Endogenous Enzyme-Blocking Reagent (DAKO, Denmark) for 10 min. Slides were washed and the Protein Block (DAKO) was applied for 5 min. The Protein Block was shaken off and rabbit polyclonal primary antibody anti-*K*_i_67 (dilution 1:100; Abcam, UK; cat. # ab16667) and anti-Cleaved Caspase 3 (dilution 1:500; Cell Signaling technology, USA; cat. # 9664) diluted in PBS or PBS only in the case of negative controls were applied. The slides were incubated for 90 min at 37 °C in a humid chamber. Then, the slides were washed and EnVision reagent (HRP, Rabbit, DAKO) was added. The slides were incubated for 30 min at 37 °C in a humid chamber. The slides were washed and stained with Liquid DAB+ substrate chromogen system (DAKO) for approximately 1 min. Finally, the slides were washed with distilled water, lightly counterstained with Mayer hematoxylin (Penta Chemicals) and mounted in CC/Mount (Diagnostic BioSystems, USA). All slide washings were performed 3-times for 5 min in PBS with 0.1% Tween 20.

### Iridium Organ Distribution

Tumor samples, kidneys, and livers were obtained from necropsy of sacrificed mice at the end of in vivo experiment (see above). The determination of Ir was performed using ICP-MS (Agilent 7700x, Agilent Japan) in a He mode to overcome potential interferences. External calibration was applied, and internal standard correction was used. Calibration solutions were prepared by diluting a multi-elemental certified reference material – water calibration solution (obtained from Analytika Ltd., Czech Republic) with the concentration of 100.0 ± 0.2 mg/L of Ir. The samples for measurement were prepared in the following way.

Initially, the tissue samples were lyophilized for 72 h using a Gregor L10–55 lyophilizer (Gregor Instruments) at −55 °C. The dried samples were then transferred to Teflon crucibles, and 2.5 mL of HNO_3_ and 0.5 mL of H_2_O_2_ were added to each sample. The crucibles were closed and placed in a MLS 1200 Mega (Milestone) microwave. After the selected mineralization program was completed, the crucibles were left to cool (10–15 min). The resulting clear mineralized samples were then quantitatively transferred to a volumetric flask and diluted to a final volume of 10 mL with deionized water. Blood samples were prepared for ICP-MS using the same method as tissue samples, with two key modifications: lyophilization was omitted, and 3 mL of HNO_3_ was used for mineralization instead of the 2.5 mL used for tissue samples.

## Supplementary Material











## Data Availability

The mass spectrometry proteomics data have been deposited to the ProteomeXchange Consortium (http://proteomecentral.proteomexchange.org) via the PRIDE partner with the data set identifier PXD069334. Other data will be made available on request.

## Notes

Results from protein identification and quantification (proteomic_all_results) (CSV)
Raw data used for creating box-plots (proteomic_box_plots) (CSV)
Data after bioinformatic processing used for PCA multivariate analysis (proteomic_PCA_analysis) (CSV)
SMILES codes for the studied coordination compounds **1**–**6**(CSV)
Structural formulas (Figure S1), HPLC traces (Figure S1), mass spectra (Figures S2–S7), NMR spectra (Figures S8–S13), crystal structures (Figure S14), results of stability studies (Figures S15–S25), supporting data for flow cytometry analysis of cell cycle and apoptosis (Figures S26 and S27), Western blot analysis of apoptosis-related caspases and PARP (Figure S28), results of release of lactate dehydrogenase (Figure S29), results of proteomic experiments (Figures S30–S52), immunohistochemistry results (Figure S53); crystallography (Tables S1–S3) and proteomic tables (Tables S4 and S5) (PDF)

## Notes

The CCDC reference numbers 2293608 (**3**) and 2300188 (**3***·MeOH) contain the supplementary crystallographic data for the X-ray studies reported in this work. The authors will release the atomic coordinates upon article publication.

## Abbreviations Used

δ: chemical shift (in ppm)
A549: human lung cancer
ACLY: ATP-citrate synthase
ACN: acetonitrile
ACO1: cytoplasmic aconitate hydratase
AhR: aryl hydrocarbon receptor
AK3: GTP:AMP phosphotransferase AK3, mitochondrial
ATF3: activating transcription factor 3
CDDP: cis-diammine-dichloridoplatinum­(II) complex (cisplatin)
CDK: cyclin dependent kinase
COSY: correlation spectroscopy
COX7C: cytochrome c oxidase subunit 7C, mitochondrial of complex IV
Cp^x^: deprotonated cyclopentadiene derivative
CS: mitochondrial citrate synthase
Ct: threshold cycle
DCM: dichloromethane
DDA: data-dependent acquisition
DDIT3: DNA damage inducible transcript 3 (CHOP)
DDR: DNA damage response
DIA: data-independent acquisition
DKC1: H/ACA ribonucleoprotein complex subunit DKC1
DMEM: Dulbecco’s modified Eagle’s medium
DMF: dimethylformamide
DMF-*d*_7_: deuterated DMF
DNA: deoxyribonucleic acid
DNAJA3: DnaJ homologue subfamily A member 3, mitochondrial
DTT: dithiothreitol
EA: early apoptotic (early apoptosis)
ECL: enhanced chemiluminescence
EE: early endosomes
EIF2S1: Eukaryotic translation initiation factor 2 subunit 1
ER: endoplasmic reticulum
ESI+: positive electrospray ionization mode
ESI-MS: electrospray ionization mass spectrometry
EXOC: exocyst complex component
FBS: fetal bovine serum
FTIR: Fourier-transform infrared spectroscopy
GADD45A: growth arrest and DNA-damage-inducible alpha
GDF15: growth differentiation factor 15
GFPT: glutamine--fructose-6-phosphate aminotransferase 1
GLS: glutaminase
GO: gene ontology
GOT1: aspartate aminotransferase 1 (cytoplasmic)
GOT2: aspartate aminotransferase 2 (mitochondrial)
GSH: reduced glutathione
HCp*: pentamethylcyclopentadiene
HCp^ph^: (2,3,4,5-tetramethylcyclopenta-2,4-dien-1-yl)­benzene
HeLa: human cervical carcinoma
HepaRG: differentiated human hepatocyte-like cell line
His: l-histidine
HMBC: heteronuclear multiple bond coherence
HMOX1: heme oxygenase 1
HMQC: heteronuclear multiple quantum coherence
HPLC: high-performance liquid chromatography
HSP: heat shock protein
HSPA5: endoplasmic reticulum chaperone BiP
HYOU1: hypoxia up-regulated protein 1
IC_50_: concentration of the compound reducing cell growth by 50%
ICP-MS: inductively coupled plasma mass spectrometry
IL: interleukin
*K*_i_-67: proliferation marker protein *K*_i_-67
KIF: kinesin-like protein
L1: 2-(di-*tert*-butylphosphanyl)­ethanamine
L2: 2-(diphenylphosphanyl)­ethanamine
L3: 3-(diphenylphosphanyl)­propan-1-amine
LA: late apoptotic (late apoptosis)
LAL: lysosomal acid lipase
LAMP1: lysosome-associated membrane glycoprotein 1
LDH: lactate dehydrogenase
LIMP2: lysosome membrane protein 2
LL: lower left
LR: lower right
LRPPRC: leucine-rich PPR motif-containing protein, mitochondrial
LY: lysosome
LysRS: Lysine--tRNA ligase (lysyl-tRNA synthetase)
MCM: mini-chromosome maintenance
MCM2–7: DNA replication licensing factors MCM2–7
MeOH: methanol
MGST1: microsomal glutathione S-transferase 1
MoA: mechanism of action
MOR: human lung carcinoma
MOR/CPR: human cisplatin-resistant lung carcinoma
mRNA: messenger RNA
MRPL15: large ribosomal subunit protein uL15m
MRPS39: small ribosomal subunit protein mS39
MS: methionine synthase
mtDNA: mitochondrial DNA
mTORC1: mammalian target of rapamycin complex 1
NAD^+^: nicotinamide adenine dinucleotide
NADH: nicotinamide adenine dinucleotide (reduced)
NME3: nucleoside diphosphate kinase 3
NMR: nuclear magnetic resonance
NPC: nuclear pore complex
NPC2: NPC intracellular cholesterol transporter 2
PAGE: polyacrylamide gel electrophoresis
PASEF: parallel accumulation serial fragmentation
PBMCs: human peripheral blood mononuclear cells
PBS: phosphate-buffered saline
PC: pyruvate carboxylase, mitochondrial
PCA: principal component analysis
PCNA: proliferating cell nuclear antigen
PDHB: pyruvate dehydrogenase E1 component subunit beta, mitochondrial
PDRG1: p53 and DNA damage-regulated protein 1
PERK: protein kinase R-like ER kinase
PHGDH: D-3-phosphoglycerate dehydrogenase
PI: propidium iodide
PICALM: phosphatidylinositol-binding clathrin assembly protein
POLA1: DNA polymerase alpha
POLD1: DNA polymerase delta catalytic subunit
POLE: DNA polymerase epsilon catalytic subunit A
POLG: DNA polymerase subunit gamma-1
PPC: healthy primary porcine chondrocytes
ppm: parts per million
PSAT1: phosphoserine aminotransferase 1
PUMA: p53 up-regulated modulator of apoptosis
PXR: pregnane X receptor
qRT-PCR: quantitative reverse transcription polymerase chain reaction
r.t.: room temperature
RA: relative activity (calculated as IC_50_(control)/IC_50_(compound of interest)
RAB34: ras-related protein Rab-34
RAB35: ras-related protein Rab-35
RAB5A: ras-related protein Rab-5A
RAB5B: ras-related protein Rab-5B
RAB5C: ras-related protein Rab-5C
RB1: retinoblastoma-associated protein 1
RFC1–5: replication factor C subunit 1–5
RI: relative intensity
RIPA: radioimmunoprecipitation assay buffer
RNA: ribonucleic acid
ROS: reactive oxygen species
RP-HPLC: reversed-phase HPLC
rRNA: ribosomal RNA
SDHA: succinate dehydrogenase [ubiquinone] flavoprotein subunit, mitochondrial
SDHB: succinate dehydrogenase [ubiquinone] iron–sulfur subunit, mitochondrial
SDS: sodium dodecyl sulfate
SE: standard error
STX4: syntaxin-4
SW982: human synovial cancer
TBS-T: tris-buffered saline Tween-20
TEAB: triethylammonium bicarbonate
TFA: trifluoroacetic acid
TFEB: transcription factor EB
TGN: trans-Golgi network
THF: tetrahydrofuran
THP-1: human leukemia
TOP2A: DNA topoisomerase 2-alpha
TP53I3: tumor protein p53-inducible protein 3
*t*_R_: retention time
TRAP1: heat shock protein 75 kDa, mitochondrial
TTK: dual specificity protein kinase TTK
UL: upper left
UNC13B: protein unc-13 homologue B
UPR: unfolded protein response
UR: upper right

## References

[1] Oun R., Moussa Y. E., Wheate N. J. (2018). The Side Effects of Platinum-based
Chemotherapy Drugs: A Review for Chemists. Dalton
Trans..

[2] Monro S., Colón K. L., Yin H., Roque J., Konda P., Gujar S., Thummel R. P., Lilge L., Cameron C. G., McFarland S. A. (2019). Transition
Metal Complexes and Photodynamic Therapy
from a Tumor-centered Approach: Challenges, Opportunities, and Highlights
from the Development of TLD1433. Chem. Rev..

[3] Mazor O., Brandis A., Plaks V., Neumark E., Rosenbach-Belkin V., Salomon Y., Scherz A. (2005). WST11, A Novel
Water-soluble Bacteriochlorophyll
Derivative; Cellular Uptake, Pharmacokinetics, Biodistribution and
Vascular-targeted Photodynamic Activity Using Melanoma Tumors as a
Model. Photochem. Photobiol..

[4] Meier-Menches S. M., Gerner C., Berger W., Hartinger C. G., Keppler B. K. (2018). Structure-activity Relationships
for Ruthenium and
Osmium Anticancer Agents - Towards Clinical Development. Chem. Soc. Rev..

[5] Štarha P. (2025). Anticancer
Iridium­(III) Cyclopentadienyl Complexes. Inorg.
Chem. Front..

[6] Máliková K., Masaryk L., Štarha P. (2021). Anticancer Half-Sandwich Rhodium­(III)
Complexes. Inorganics.

[7] Schafer S., Ott I., Gust R., Sheldrick W. S. (2007). Influence
of the Polypyridyl (pp)
Ligand Size on the DNA Binding Properties, Cytotoxicity and Cellular
Uptake of Organoruthenium­(II) Complexes of the Type [(η^6^-C_6_Me_6_)­Ru­(L)­(pp)]^
*n*+^ [L = Cl, *n* = 1; L = (NH_2_)_2_CS, *n* = 2]. Eur. J.
Inorg. Chem..

[8] Ma D.-L., Wu C., Wu K.-J., Leung C.-H. (2019). Iridium­(III) Complexes Targeting
Apoptotic Cell Death in Cancer Cells. Molecules.

[9] Lord R. M., McGowan P. C. (2019). Organometallic Iridium
Arene Compounds: The Effects
of *C*-Donor Ligands on Anticancer Activity. Chem. Lett..

[10] Štarha P., Trávníček Z. (2019). Non-platinum Complexes Containing
Releasable Biologically Active Ligands. Coord.
Chem. Rev..

[11] Park C., Jeong J. (2018). Synergistic Cellular Responses to Heavy Metal Exposure: A Minireview. Biochim. Biophys. Acta Gen. Subj..

[12] DeMoor J. M., Koropatnick D. J. (2000). Metals
and Cellular Signaling in Mammalian Cells. Cell
Mol. Biol..

[13] Kryczka J., Kryczka J., Czarnecka-Chrebelska K. H., Brzeziańska-Lasota E. (2021). Molecular
Mechanisms of Chemoresistance Induced by Cisplatin in NSCLC Cancer
Therapy. Int. J. Mol. Sci..

[14] Galluzzi L., Senovilla L., Vitale I., Michels J., Martins I., Kepp O., Castedo M., Kroemer G. (2012). Molecular Mechanisms
of Cisplatin Resistance. Oncogene.

[15] Hearn J. M., Hughes G. M., Romero-Canelón I., Munro A. F., Rubio-Ruiz B., Liu Z., Carragher N. O., Sadler P. J. (2018). Pharmaco-genomic Investigations of Organo-iridium Anticancer
Complexes Reveal Novel Mechanism of Action. Metallomics.

[16] Hearn J. M., Romero-Canelón I., Munro A. F., Fu Y., Pizarro A. M., Garnett M. J., McDermott U., Carragher N. O., Sadler P. J. (2015). Potent Organo-osmium
Compound Shifts
Metabolism in Epithelial Ovarian Cancer Cells. Proc. Natl. Acad. Sci. U.S.A..

[17] Pete S., Roy N., Kar B., Paira P. (2022). Construction of Homo and Heteronuclear
Ru­(II), Ir­(III) and Re­(I) Complexes for Target Specific Cancer Therapy. Coord. Chem. Rev..

[18] Sharma
S A., P S., Roy N., Paira P. (2020). Advances in Novel Iridium­(III)
Based Complexes for Anticancer Applications: A Review. Inorg. Chim. Acta.

[19] Li J., Guo L., Tian Z., Tian M., Zhang S., Xu K., Qian Y., Liu Z. (2017). Novel Half-sandwich Iridium­(III)
Imino-pyridyl Complexes Showing Remarkable *in Vitro* Anticancer Activity. Dalton Trans..

[20] Xie Y., Zhang S., Ge X., Ma W., He X., Zhao Y., Ye J., Zhang H., Wang A., Liu Z. (2020). Lysosomal-targeted Anticancer Half-sandwich
Iridium­(III) Complexes
Modified with Lonidamine Amide Derivatives. Appl. Organomet. Chem..

[21] Novohradsky V., Zerzankova L., Stepankova J., Kisova A., Kostrhunova H., Liu Z., Sadler P. J., Kasparkova J., Brabec V. (2014). A Dual-targeting, Apoptosis-inducing
Organometallic Half-sandwich Iridium Anticancer Complex. Metallomics.

[22] Betanzos-Lara S., Liu Z., Habtemariam A., Pizarro A. M., Qamar B., Sadler P. J. (2012). Organometallic
Ruthenium and Iridium Transfer-Hydrogenation Catalysts Using Coenzyme
NADH as a Cofactor. Angew. Chem., Int. Ed..

[23] Štarha P., Habtemariam A., Romero-Canelon I., Clarkson G. J., Sadler P. J. (2016). Hydrosulfide
Adducts of Organo-Iridium Anticancer Complexes. Inorg. Chem..

[24] Xu Z., Zhang Y., Zhang S., Jia X., Zhong G., Yang Y., Du Q., Li J., Liu Z. (2019). Novel Half-sandwich
Iridium ÔC (Carbene)-Complexes: *In Vitro* and *in Vivo* Tumor Growth Suppression and Pro-apoptosis via ROS-mediated
Cross-talk Between Mitochondria and Lysosomes. Cancer Lett..

[25] Bose S., Nguyen H. D., Ngo A. H., Do L. H. (2022). Fluorescent
Half-sandwich
Iridium Picolinamidate Complexes for in-Cell Visualization. J. Inorg. Biochem..

[26] Soldevila-Barreda J.
J., Sadler P. J. (2015). Approaches
to the Design of Catalytic Metallodrugs. Curr.
Opin. Chem. Biol..

[27] Yu Z., Cowan J. A. (2017). Catalytic Metallodrugs: Substrate-Selective Metal Catalysts
as Therapeutics. Chem.–Eur. J..

[28] Abdur-Rashid K., Guo R., Lough A. J., Morris R. H., Song D. (2005). Synthesis of Ruthenium
Hydride Complexes Containing beta-Aminophosphine Ligands Derived from
Amino Acids and their use in the H_2_-Hydrogenation of Ketones
and Imines. Adv. Synth. Catal..

[29] Blaquiere N., Diallo-Garcia S., Gorelsky S. I., Black D. A., Fagnou K. (2008). Ruthenium-Catalyzed
Dehydrogenation of Ammonia Boranes. J. Am. Chem.
Soc..

[30] Wingad R.
L., Gates P. J., Street S. T. G., Wass D. F. (2015). Catalytic Conversion
of Ethanol to *n*-Butanol Using Ruthenium P–N
Ligand Complexes. ACS Catal..

[31] Mrkvicová A., Peterová E., Nemec I., Křikavová R., Muthná D., Havelek R., Kazimírová P., Řezáčová M., Štarha P. (2023). Rh­(III) and
Ru­(II) Complexes with Phosphanyl-alkylamines: Inhibition of DNA Synthesis
Induced by Anticancer Rh Complex. Fut. Med.
Chem..

[32] Hu X., Guo L., Liu M., Zhang Q., Gong Y., Sun M., Feng S., Xu Y., Liu Y., Liu Z. (2022). Increasing
Anticancer Activity with Phosphine Ligation in Zwitterionic Half-Sandwich
Iridium­(III), Rhodium­(III), and Ruthenium­(II) Complexes. Inorg. Chem..

[33] Liu Z., Habtemariam A., Pizarro A. M., Clarkson G. J., Sadler P. J. (2011). Organometallic
Iridium­(III) Cyclopentadienyl Anticancer Complexes Containing C,N-Chelating
Ligands. Organometallics.

[34] Štarha P., Hošek J., Trávníček Z., Dvořák Z. (2020). Cytotoxic
Dimeric Half-sandwich Ru­(II), Os­(II) and
Ir­(III) Complexes Containing the 4,4′-Biphenyl-based Bridging
Ligands. Appl. Organomet. Chem..

[35] Liu Z., Habtemariam A., Pizarro A. M., Fletcher S. A., Kisova A., Vrana O., Salassa L., Bruijnincx P. C. A., Clarkson G. J., Brabec V., Sadler P. J. (2011). Organometallic Half-Sandwich
Iridium Anticancer Complexes. J. Med. Chem..

[36] Zhang H., Guo L., Tian Z., Tian M., Zhang S., Xu Z., Gong P., Zheng X., Zhao J., Liu Z. (2018). Significant
Effects of Counteranions on the Anticancer Activity of Iridium­(III)
Complexes. Chem. Commun..

[37] Jia W., Chen X., Guo R., Sui-Seng C., Amoroso D., Lough A. J., Abdur-Rashid K. (2009). Aminophosphine
Ligands R_2_P­(CH_2_)_
*n*
_NH_2_ and
Ruthenium Hydrogenation Catalysts RuCl_2_(R_2_P­(CH_2_)_
*n*
_NH_2_)_2_. Dalton Trans..

[38] Hošek J., Petrželová K., Héžová R., Straková N., Kajabová S., Nemec I., Šimečková P., Pěnčíková K., Mašek J., Moncol’ J., Štarha P. (2025). Highly Effective Ru­(II) and Os­(II)
Half-sandwich Complexes Induce Cytotoxicity in Cancer Cells through
Combined Mitochondrial and Endoplasmic Reticulum Stress. Eur. J. Med. Chem..

[39] Křikavová R., Héžová R., Hošek J., Otřísalová M., Nemec I., Štarha P. (2026). A Critical
Re-evaluation of the Ta­(V) Half-sandwich Scaffold: Synthesis, Hydrolytic
Behaviour, and the Challenge of Lung Cancer Cell Viability. Inorg. Chem. Commun..

[40] Petrželová K., Bárta O., Héžová R., Andrýsková A., Hošek J., Štarha P. (2025). Improved Synthesis of Dinuclear [M­(μ-X)­(η^5^-Cp*)­X]_2_ Precursors for Half-Sandwich Complexes
(M = Rh or Ir; X = Br or I). ChemistryOpen.

[41] Liu Z., Romero-Canelon I., Qamar B., Hearn J. M., Habtemariam A., Barry N. P. E., Pizarro A. M., Clarkson G. J., Sadler P. J. (2014). The Potent
Oxidant Anticancer Activity of Organoiridium Catalysts. Angew. Chem., Int. Ed..

[42] Merlino A. (2023). Metallodrug
binding to serum albumin: Lessons from biophysical and structural
studies. Coord. Chem. Rev..

[43] Hassoon A. A., Szorcsik A., Gajda T. (2025). Interaction
of half-sandwich Rh­(III)
ion and some of its complexes with endogenous imidazole derivatives. J. Inorg. Biochem..

[44] Zhang W.-Y., Banerjee S., Hughes G. M., Bridgewater H. E., Song J.-I., Breeze B. G., Clarkson G. J., Coverdale J. P. C., Sanchez-Cano C., Ponte F., Sicilia E., Sadler P. J. (2020). Ligand-centred
Redox Activation of Inert Organoiridium Anticancer Catalysts. Chem. Sci..

[45] Burgoyne A. R., Kaschula C. H., Parker M. I., Smith G. S. (2016). *In vitro* Cytotoxicity of Half-Sandwich Platinum Group Metal Complexes of
a Cationic Alkylated Phosphaadamantane Ligand. Eur. J. Inorg. Chem..

[46] Masaryk L., Orvoš J., Słoczyńska K., Herchel R., Moncol J., Milde D., Halaš P., Křikavová R., Koczurkiewicz-Adamczyk P., Pękala E., Fischer R., Šalitroš I., Nemec I., Štarha P. (2022). Anticancer Half-sandwich Ir­(III)
Complex and its Interaction with Various Biomolecules and their Mixtures
- A Case Study with Ascorbic Acid. Inorg. Chem.
Front..

[47] Zanoni M., Piccinini F., Arienti C., Zamagni A., Santi S., Polico R., Bevilacqua A., Tesei A. (2016). 3D Tumor Spheroid Models
for *in Vitro* Therapeutic Screening: A Systematic
Approach to Enhance the Biological Relevance of Data Obtained. Sci. Rep..

[48] Kozieł S., Komarnicka U. K., Ziółkowska A., Skórska-Stania A., Pucelik B., Płotek M., Sebastian V., Bieńko A., Stochel G., Kyzioł A. (2020). Anticancer
Potency of Novel Organometallic Ir­(III) Complexes with Phosphine Derivatives
of Fluoroquinolones Encapsulated in Polymeric Micelles. Inorg. Chem. Front..

[49] Štarha P., Trávníček Z., Crlíková H., Vančo J., Kašpárková J., Dvořák Z. (2018). Half-Sandwich
Ir­(III) Complex of *N*1-Pyridyl-7-azaindole Exceeds
Cytotoxicity of Cisplatin at Various
Human Cancer Cells and 3D Multicellular Tumor Spheroids. Organometallics.

[50] Fares J., Fares M. Y., Khachfe H. H., Salhab H. A., Fares Y. (2020). Molecular
Principles of Metastasis: A Hallmark of Cancer Revisited. Sig. Transduct. Target. Ther..

[51] Barnaba N., LaRocque J. R. (2021). Targeting Cell Cycle Regulation *via* the G2-M Checkpoint for Synthetic Lethality in Melanoma. Cell Cycle.

[52] Liu X., Chen S., Ge X., Zhang Y., Xie Y., Hao Y., Wu D., Zhao J., Yuan X.-A., Tian L., Liu Z. (2020). Dual Functions of Iridium­(III) 2-Phenylpyridine Complexes: Metastasis
Inhibition and Lysosomal Damage. J. Inorg. Biochem..

[53] Mondal A., Shanavas S., Sen U., Das U., Roy N., Bose B., Paira P. (2022). Mitochondria-targeted
Half-sandwich
Iridium­(III)-Cp*-arylimidazophenanthroline Complexes as Antiproliferative
and Bioimaging Agent against Triple Negative Breast Cancer Cells MDA-MB-468. RSC Adv..

[54] Gonzalo-Navarro C., Zafon E., Organero J. A., Jalón F. A., Lima J. C., Espino G., Rodríguez A. M., Santos L., Moro A. J., Barrabés S., Castro J., Camacho-Aguayo J., Massaguer A., Manzano B. R., Durá G. (2024). Ir­(III) Half-sandwich Photosensitizers
with a π-Expansive Ligand for Efficient Anticancer Photodynamic
Therapy. J. Med. Chem..

[55] Yang Y., Gao Y., Zhao J., Gou S. (2024). An Electron-accepting Half-sandwich
Iridium­(III) Complex for the Treatment of Hypoxic Tumors via Synergetic
Chemo- and Phototherapy. Inorg. Chem. Front..

[56] Ludwig G., Mijatović S., Randelović I., Bulatović M., Miljković D., Maksimović-Ivanić D., Korb M., Lang H., Steinborn D., Kaluderović G.
N. (2013). Biological Activity
of Neutral and
Cationic Iridium­(III) Complexes with κP and κP,κS
Coordinated Ph_2_PCH_2_S­(O)_x_Ph (x = 0–2)
Ligands. Eur. J. Med. Chem..

[57] Li J., Tian Z., Xu Z., Zhang S., Feng Y., Zhang L., Liu Z. (2018). Highly Potent
Half-sandwich Iridium
and Ruthenium Complexes as Lysosome-targeted Imaging and Anticancer
Agents. Dalton Trans..

[58] Hu H., Zhang F., Sheng Z., Tian S., Li G., Tang S., Niu Y., Yang J., Liu Y. (2024). Synthesis
and Mitochondria-localized Iridium (III) Complexes Induce Cell Death
Through Pyroptosis and Ferroptosis Pathways. Eur. J. Med. Chem..

[59] Zhang H., Tian L., Xiao R., Zhou Y., Zhang Y., Hao J., Liu Y., Wang J. (2021). Anticancer
Effect Evaluation *in Vitro* and *in Vivo* of Iridium­(III) Polypyridyl
Complexes Targeting DNA and Mitochondria. Bioorg.
Chem..

[60] Bonsignore G., Martinotti S., Ranzato E. (2023). Endoplasmic Reticulum Stress and
Cancer: Could Unfolded Protein Response Be a Druggable Target for
Cancer Therapy?. Int. J. Mol. Sci..

[61] Zhang Y. Q., Gong Y., Liang Z. J., Wu W., Chen J. X., Li Y. L., Chen R., Mei J., Huang Z. N., Sun J. (2024). Mitochondria- and Endoplasmic Reticulum-localizing
Iridium­(III) Complexes
Induce Immunogenic Cell Death of 143B cells. J. Inorg. Biochem..

[62] Sicari D., Delaunay-Moisan A., Combettes L., Chevet E., Igbaria A. (2020). A Guide to
Assessing Endoplasmic Reticulum Homeostasis and Stress in Mammalian
Systems. FEBS J..

[63] Cao R., Jia J., Ma X., Zhou M., Fei H. (2013). Membrane Localized
Iridium­(III) Complex Induces Endoplasmic Reticulum Stress and Mitochondria-Mediated
Apoptosis in Human Cancer Cells. J. Med. Chem..

[64] Ramos R., Gilles J.-F., Morichon R., Przybylski C., Caron B., Botuha C., Karaiskou A., Salmain M., Sobczak-Thépot J. (2021). Cytotoxic BODIPY-Appended
Half-Sandwich Iridium­(III) Complex Forms Protein Adducts and Induces
ER Stress. J. Med. Chem..

[65] Deng Z., Li H., Chen S., Wang N., Liu G., Liu D., Ou W., Xu F., Wang X., Lei D., Lo P.-C., Li Y. Y., Lu J., Yang M., He M.-L., Zhu G. (2023). Near-infrared-activated
Anticancer Platinum­(IV) Complexes Directly
Photooxidize Biomolecules in an Oxygen-independent Manner. Nat. Chem..

[66] Hernández-García A., Marková L., Santana M. D., Prachařová J., Bautista D., Kostrhunová H., Novohradský V., Brabec V., Ruiz J., Kašpárková J. (2023). Cyclometalated
Benzimidazole Osmium­(II) Complexes with Antiproliferative Activity
in Cancer Cells Disrupt Calcium Homeostasis. Inorg. Chem..

[67] Xu C., Li C. Y.-T., Kong A.-T. (2005). Induction
of Phase I, II and III
Drug Metabolism/Transport by Xenobiotics. Arch.
Pharm. Res..

[68] Fulda S., Gorman A. M., Hori O., Samali A. (2010). Cellular Stress Responses:
Cell Survival and Cell Death. Int. J. Cell.
Biol..

[69] Jennings P., Limonciel A., Felice L., Leonard M. O. (2013). An Overview of Transcriptional
Regulation in Response to Toxicological Insult. Arch. Toxicol..

[70] Simmons S. O., Fan C. Y., Ramabhadran R. (2009). Cellular Stress
Response Pathway
System as a Sentinel Ensemble in Toxicological Screening. Toxicol. Sci..

[71] Kim K. H., Lee M. S. (2021). GDF15 as a Central Mediator for Integrated Stress Response
and a Promising Therapeutic Molecule for Metabolic Disorders and NASH. Biochim. Biophys. Acta Gen. Subj..

[72] Shimizu M., Sato R. (2022). Endocrine Fibroblast
Growth Factors in Relation to Stress Signaling. Cells.

[73] Li W., Li T., Pan Y., Li S., Xu G., Zhang Z., Liang H., Yang F. (2024). Designing
a Mitochondria-Targeted
Theranostic Cyclometalated Iridium­(III) Complex: Overcoming Cisplatin
Resistance and Inhibiting Tumor Metastasis through Necroptosis and
Immune Response. J. Med. Chem..

[74] Acuña M. I., Rubio A. R., Martínez-Alonso M., Busto N., Rodríguez A. M., Davila-Ferreira N., Smythe C., Espino G., García B., Domínguez F. (2023). Targets, Mechanisms and Cytotoxicity
of Half-Sandwich Ir­(III) Complexes Are Modulated by Structural Modifications
on the Benzazole Ancillary Ligand. Cancers.

[75] Dasari S., Bernard Tchounwou P. (2014). Cisplatin
in Cancer Therapy: Molecular Mechanisms of
Action. Eur. J. Pharmacol..

[76] Iadevaia V., Zhang Z., Jan E., Proud C. G. (2012). mTOR Signaling
Regulates
the Processing of pre-rRNA in Human Cells. Nucleic
Acids Res..

[77] Potapova T. A., Unruh J. R., Conkright-Fincham J., Banks C. A., Florens L., Schneider D. A., Gerton J. L. (2023). Distinct States of Nucleolar Stress
Induced by Anti-cancer Drugs. eLife.

[78] Shcherbik N., Pestov D. G. (2019). The Impact of Oxidative Stress on Ribosomes: From Injury
to Regulation. Cells.

[79] Romashko D. N., Marban E., O’Rourke B. (1998). Subcellular Metabolic Transients
and Mitochondrial Redox Waves in Heart Cells. Proc. Natl. Acad. Sci. U.S.A..

[80] Modica-Napolitano J. S., Aprille J. R. (1987). Basis for the Selective Cytotoxicity of Rhodamine 123. Cancer Res..

[81] Payne C. K., Jones S. A., Chen C., Zhuang X. (2007). Internalization and
Trafficking of Cell Surface Proteoglycans and Proteoglycan-binding
Ligands. Traffic.

[82] Shpilka T., Haynes C. (2018). The Mitochondrial UPR:
Mechanisms, Physiological Functions
And Implications In Ageing. Nat. Rev. Mol. Cell
Biol..

[83] Schiliro C., Firestein B. L. (2021). Mechanisms of Metabolic Reprogramming in Cancer Cells
Supporting Enhanced Growth and Proliferation. Cells.

[84] Valdebenito G. E., Chacko A. R., Duchen M. R. (2023). The Mitochondrial
ATP Synthase as
an ATP Consumer - A Surprising Therapeutic Target. EMBO J..

[85] Kiesel V. A., Sheeley M. P., Coleman M. F., Cotul E. K., Donkin S. S., Hursting S. D., Wendt M. K., Teegarden D. (2021). Pyruvate Carboxylase
and Cancer Progression. Cancer Metab..

[86] Paneque A., Fortus H., Zheng J., Werlen G., Jacinto E. (2023). The Hexosamine
Biosynthesis Pathway: Regulation and Function. Genes.

[87] Kim H., Park Y. J. (2018). Links Between Serine
Biosynthesis Pathway and Epigenetics
in Cancer Metabolism. Clin. Nutr. Res..

[88] Lee C. M., Hwang Y., Kim M., Park Y.-C., Kim H., Fang S. (2024). PHGDH: A Novel Therapeutic
Target in Cancer. Exp. Mol. Med..

[89] Zhang X., Wang Z. (2024). Targeting SHMTs and
MTHFDs in Cancer: Attractive Opportunity for
Anti-Tumor Strategy. Front. Pharmacol..

[90] Sun W., Liu R., Gao X., Lin Z., Tang H., Cui H., Zhao E. (2023). Targeting Serine-Glycine-One-Carbon
Metabolism as a Vulnerability
in Cancers. Biomarker Res..

[91] Mazzarino R. C., Baresova V., Zikánová M., Duval N., Wilkinson T. G., Patterson D., Vacano G. N. (2021). Transcriptome and Metabolome Analysis of CrGART, a
Novel Cell Model of De Novo Purine Synthesis Deficiency: Alterations
in CD36 Expression and Activity. PLoS One.

[92] Park H. J., Hong Y. B., Choi Y. C., Lee J., Kim E. J., Lee J. S., Mo W. M., Ki S. M., Kim H. I., Kim H. J., Hyun Y. S., Hong H. D., Nam K., Jung S. C., Kim S. B., Kim S. H., Kim D. H., Oh K. W., Kim S. H., Yoo J. H., Lee J. E., Chung K. W., Choi B. O. (2016). ADSSL1Mutation Relevant to Autosomal
Recessive Adolescent Onset Distal Myopathy. Ann. Neurol..

[93] Lavin M. F., Gueven N. (2006). The Complexity of p53
Stabilization and Activation. Cell Death Differ..

[94] Huang C.-H., Yang T.-T., Lin K.-I. (2024). Mechanisms
and Functions of SUMOylation
in Health and Disease: A Review Focusing on Immune Cells. J. Biomed. Sci..

[95] Chin H. S., Li M. X., Tan I. K. L., Ninnis R. L., Reljic B., Scicluna K., Dagley L. F., Sandow J. J., Kelly G. L., Samson A. L., Chappaz S., Khaw S. L., Chang C., Morokoff A., Brinkmann K., Webb A., Hockings C., Hall C. M., Kueh A. J., Ryan M. T., Kluck R. M., Bouillet P., Herold M. J., Gray D. H. D., Huang D. C. S., van Delft M. F., Dewson G. (2018). VDAC2 Enables BAX to Mediate Apoptosis
and Limit Tumor Development. Nat. Commun..

[96] Ma S., Nguyen T., Tan I., Ninnis R., Iyer S., Stroud D. A., Menard M., Kluck R. M., Ryan M. T., Dewson G. (2014). Bax Targets Mitochondria
by Distinct Mechanisms Before
or During Apoptotic Cell Death: a Requirement for VDAC2 or Bak for
Efficient Bax Apoptotic Function. Cell Death
Differ..

[97] Pedley R., King L. E., Mallikarjun V., Wang P., Swift J., Brennan K., Gilmore A. P. (2020). BioID-based Proteomic Analysis of
the Bid Interactome Identifies Novel Proteins Involved in Cell-cycle-dependent
Apoptotic Priming. Cell Death Dis..

[98] Yuan X., Gajan A., Chu Q., Xiong H., Wu K., Wu G. S. (2018). Developing TRAIL/TRAIL
Death Receptor-based Cancer Therapies. Cancer
Metastasis Rev..

[99] Schneider P., Thome M., Burns K., Bodmer J.-L., Hofmann K., Kataoka T., Holler N., Tschopp J. (1997). TRAIL Receptors
1 (DR4)
and 2 (DR5) Signal FADD-Dependent Apoptosis and Activate NF-κB. Immunity.

[100] Walczak, H. ; Haas, T. L. Biochemical Analysis of the Native TRAIL Death-Inducing Signaling Complex. In: Mor, G. ; Alvero, A. B. (eds) Apoptosis and Cancer. Methods in Molecular Biology; Humana Press, 2008, 414. 10.1007/978-1-59745-339-4_16 18175822

[101] Chen B. B., Pan N. L., Liao J. X., Huang M. Y., Jiang D. C., Wang J. J., Qiu H. J., Chen J. X., Li L., Sun J. (2021). Cyclometalated Iridium­(III)
Complexes as Mitochondria-Targeted
Anticancer and Antibacterial Agents to Induce Both Autophagy and Apoptosis. J. Inorg. Biochem..

[102] Kuang S., Wei F., Karges J., Ke L., Xiong K., Liao X., Gasser G., Ji L., Chao H. (2022). Photodecaging of a Mitochondria-localized Iridium­(III) Endoperoxide
Complex for Two-photon Photoactivated Therapy under Hypoxia. J. Am. Chem. Soc..

[103] Mitchell R. J., Kriger S. M., Fenton A. D., Havrylyuk D., Pandeya A., Sun Y., Smith T., DeRouchey J. E., Unrine J. M., Oza V., Blackburn J. S., Wei Y., Heidary D. K., Glazer E. C. (2023). A Mono Adduct Generating Ru­(II) Complex
Induces Ribosome Biogenesis Stress and Is a Molecular Mimic of Phenanthriplatin. RSC Chem. Biol..

[104] Lafita-Navarro M. C., Conacci-Sorrell M. (2023). Nucleolar Stress: From Development
to Cancer. Semin. Cell Dev. Biol..

[105] Lu Y., Wang S., Jiao Y. (2023). The Effects
of Deregulated Ribosomal
Biogenesis in Cancer. Biomolecules.

[106] Zhu J., Liu Y., Zhang Z., Yang X., Qiu F. (2023). Cyclometalated
Ir­(III) Complexes as Lysosome-targeted Photodynamic Anticancer Agents. ACS Omega.

[107] Chaudhary A., Kumar A., Swain N., Chaudhary K., Sonker H., Dewan S., Patil R. A., Singh R. G. (2025). Endocytic
Uptake of Self-assembled Iridium­(III) Nanoaggregates for Holistic
Treatment of Metastatic 3D Triple-negative Breast Tumor Spheroids. Small.

[108] Jin C., Li G., Wu X., Liu J., Wu W., Chen Y., Sasaki T., Chao H., Zhang Y. (2021). Robust Packing
of a Self-assembling Iridium Complex via Endocytic Trafficking for
Long-term Lysosome Tracking. Angew. Chem., Int.
Ed..

[109] Wang F. X., Chen M. H., Hu X. Y., Ye R. R., Tan C. P., Ji L. N., Mao Z. W. (2016). Ester-modified Cyclometalated
Iridium­(III) Complexes as Mitochondria-targeting Anticancer Agents. Sci. Rep..

[110] Yang T., Zhu M., Jiang M., Yang F., Zhang Z. (2022). Current Status of Iridium-based Complexes
Against Lung Cancer. Front. Pharmacol..

[111] Shao M., Yao M., Liu X., Gao C., Liu W., Guo J., Zong J., Sun X., Liu Z. (2021). In Vitro and
In Vivo of Triphenylamine-appended Fluorescent Half-sandwich Iridium­(III)
Thiosemicarbazones Antitumor Complexes. Inorg.
Chem..

[112] Huang M., Myers C. R., Wang Y., You M. (2021). Mitochondria
as a Novel Target for Cancer Chemoprevention: Emergence of Mitochondrial-targeting
Agents. Cancer Prev. Res..

[113] Bhat P. J., Darunte L., Kareenhalli V., Dandekar J., Kumar A. (2011). Can Metabolic Plasticity Be a Cause
for Cancer? Warburg–Waddington Legacy Revisited. Clin. Epigenetics.

[114] Sionov, R. V. ; Hayon, I. L. ; Haupt, Y. The Regulation of p53 Growth Suppression. In Madame Curie Bioscience Database [Online]; Landes Bioscience: Austin, TX, 2000–2013. Available from: https://www.ncbi.nlm.nih.gov/books/NBK6412/, https://www.ema.europa.eu/en/ich-s8-immunotoxicity-studies-human-pharmaceuticals-scientific-guideline.

[115] Available from: https://www.ema.europa.eu/en/ich-s8-immunotoxicity-studies-human-pharmaceuticals-scientific-guideline.

[116] Morris D. M., McGeagh M., De Peña D., Merola J. S. (2014). Extending the Range of Pentasubstituted Cyclopentadienyl
Compounds: The Synthesis of a Series of Tetramethyl­(alkyl or aryl)­cyclopentadienes
(Cp*R), their Iridium Complexes and their Catalytic Activity for Asymmetric
Transfer Hydrogenation. Polyhedron.

[117] Fu S., Chen N.-Y., Liu X., Shao Z., Luo S.-P., Liu Q. (2016). Ligand-Controlled Cobalt-Catalyzed
Transfer Hydrogenation of Alkynes:
Stereodivergent Synthesis of *Z*- and *E*-Alkenes. J. Am. Chem. Soc..

[118] White C., Yates A., Maitlis P. M., Heinekey D. M., Grimes R. N. (1992). (η^5^-Pentamethylcyclopentadienyl)­Rhodium
and -Iridium Compounds. Inorg. Synth..

[119] Tönnemann J., Risse J., Grote Z., Scopelliti R., Severin K. (2013). Efficient and Rapid Synthesis of
Chlorido-Bridged Half-Sandwich
Complexes of Ruthenium, Rhodium, and Iridium by Microwave Heating. Eur. J. Inorg. Chem..

[120] Sheldrick G. M. (2015). SHELXT - Integrated Space-group and Crystal-structure
Determination. Acta Crystallogr..

[121] Bourhis L. J., Dolomanov O. V., Gildea R., Howard J. A. K., Puschmann H. (2015). The Anatomy
of a Comprehensive Constrained, Restrained
Refinement Program for the Modern Computing Environment - Olex2 Dissected. Acta Crystallogr..

[122] Rigaku Oxford Diffraction, CrysAlisPro 1.171.40.82a, 2020, rigaku.com.

[123] Macrae C. F., Sovago I., Cottrell S. J., Galek P. T. A., McCabe P., Pidcock E., Platings M., Shields G. P., Stevens J. S., Towler M., Wood P. A. (2020). Mercury
4.0: From
Visualization to Analysis, Design and Prediction. J. Appl. Crystallogr..

[124] Pisárčik M., Lukáč M., Jampílek J., Pašková L’., Bilka F., Bilková A., Devínsky F., Val’ko J., Horáková R., Hošek J., Březina M., Opravil T. (2022). Controlled Synthesis of Gemini Surfactant-capped
Gold Nanoparticles. Gemini Structure-nanoparticle Properties Relationship
Study. J. Mol. Liq..

[125] Kimlin L. C., Casagrande G., Virador V. M. (2013). *In Vitro* Three-dimensional (3D) Models in Cancer Research: An Update. Mol. Carcinog..

[126] Hofmanová J., Slavík J., Ovesná P., Tylichová Z., Vondráček J., Straková N., Vaculová A. H., Ciganek M., Kozubík A., Knopfová L., Šmarda J., Machala M. (2017). Dietary Fatty Acids
Specifically Modulate Phospholipid Pattern in Colon Cells with Distinct
Differentiation Capacities. Eur. J. Nutr..

[127] Šimečková P., Hubatka F., Kotouček J., Turánek Knötigová P., Mašek J., Slavík J., Kováč O., Neča J., Kulich P., Hrebík D., Stráská J., Pěnčíková K., Procházková J., Diviš P., Macaulay S., Mikulík R., Raška M., Machala M., Turánek J. (2020). Gadolinium
Labelled Nanoliposomes as the Platform for MRI Theranostics: *In Vitro* Safety Study in Liver Cells and Macrophages. Sci. Rep..

[128] Šimečková P., Pěnčíková K., Kováč O., Slavík J., Pařenicová M., Vondráček J., Machala M. (2022). *In Vitro* Profiling of Toxic Effects
of Environmental Polycyclic Aromatic
Hydrocarbons on Nuclear Receptor Signaling, Disruption of Endogenous
Metabolism and Induction of Cellular Stress. Sci. Total Environ..

[129] Schmittgen T. D., Livak K. J. (2008). Analyzing Real-time
PCR Data by the
Comparative C­(T) Method. Nat. Protoc..

[130] Masuda T., Tomita T., Ishihama Y. (2008). Phase Transfer
Surfactant-Aided
Trypsin Digestion for Membrane Proteome Analysis. J. Proteome Res..

[131] Rappsilber J., Mann M., Ishihama Y. (2007). Protocol for Micro-purification,
Enrichment, Pre-fractionation and Storage of Peptides for Proteomics
Using StageTips. Nat. Protoc..

[132] Yu F., Haynes S. E., Teo G. C., Avtonomov D. M., Polasky D. A., Nesvizhskii A. I. (2020). Fast Quantitative
Analysis of timsTOF
PASEF Data with MSFragger and IonQuant. Mol.
Cell. Proteomics.

[133] Kong A. T., Leprevost F. V., Avtonomov D. M., Mellacheruvu D., Nesvizhskii A. I. (2017). MSFragger:
Ultrafast and Comprehensive
Peptide Identification in Mass Spectrometry-based Proteomics. Nat. Methods.

[134] Yang K. L., Yu F., Teo G. C., Li K., Demichev V., Ralser M., Nesvizhskii A. I. (2023). MSBooster:
Improving Peptide Identification Rates Using Deep Learning-based Features. Nat. Commun..

[135] Käll L., Canterbury J. D., Weston J., Noble W. S., MacCoss M. J. (2007). Semi-supervised
Learning for Peptide Identification
from Shotgun Proteomics Datasets. Nat. Methods.

[136] da Veiga Leprevost F., Haynes S. E., Avtonomov D. M., Chang H. Y., Shanmugam A. K., Mellacheruvu D., Kong A. T., Nesvizhskii A. I. (2020). Philosopher: A Versatile Toolkit
for Shotgun Proteomics Data Analysis. Nat. Methods.

[137] Demichev V., Szyrwiel L., Yu F., Teo G. C., Rosenberger G., Niewienda A., Ludwig D., Decker J., Kaspar-Schoenefeld S., Lilley K. S., Mülleder M., Nesvizhskii A. I., Ralser M. (2022). dia-PASEF Data Analysis Using FragPipe
and DIA-NN for Deep Proteomics of Low Sample Amounts. Nat. Commun..

[138] Ge S. X., Jung D., Yao R. (2020). ShinyGO: A
Graphical
Gene-set Enrichment Tool for Animals and Plants. Bioinformatics.

[139] Jarošová R., Ondráčková P., Patočka Z., Sládek Z. (2021). Comparison of Cryoprotective Methods
for Histological Examination of Rat and Porcine Lung Tissue. Acta Vet. Brno.

