# Supplementary material for: Mechanistic Exploration of Half-Sandwich Iridium(III) Anticancer Compounds through Integrated Cellular, Proteomic, and In Vivo Analyses
Source: J Med Chem. 2026 Jun 16;69(13):15166–90. doi: 10.1021/acs.jmedchem.5c03448 (PMC13370875; doi:10.1021/acs.jmedchem.5c03448)
Supplement: Supplementary file 5 [file jm5c03448_si_007.pdf]

Supporting Information

for

Mechanistic Exploration of Half-Sandwich Iridium(III) Anticancer Compounds through Integrated Cellular, Proteomic, and In Vivo Analyses

Pavel Štarha [a,¶,*], Jaroslava Friedecká [b,¶], Renata Héžová [c], Ondřej Bárta [a], Rea Jarošová [c], Josef Mašek [c], Ivan Nemec [a], David Milde [d], Adam Novobilský [c], Ladislav Novotný [e,f], Jaroslav Ondruš [c], Slavomíra Šterbinská [a], Nicol Straková [c], René Lenobel [b, **], Jan Hošek [c,***]

[a] *Department of Inorganic Chemistry, Faculty of Science, Palacký University Olomouc, 17. listopadu 12, 77146 Olomouc, Czech Republic*

[b] *Laboratory of Growth Regulators, Institute of Experimental Botany of the Czech Academy of Sciences, and Faculty of Science, Palacký University, Šlechtitelů 27, 783 71 Olomouc, Czech Republic*

[c] *Department of Pharmacology and Toxicology, Veterinary Research Institute, Hudcova 296/70, 62100 Brno, Czech Republic*

[d] *Department of Analytical Chemistry, Faculty of Science, Palacký University Olomouc, 17. listopadu 12, 77146 Olomouc, Czech Republic*

[e] *Department of Veterinary Sciences, Faculty of Agrobiology, Food and Natural Resources, Czech University of Life Sciences, Kamýcká 129, 16500 Prague, Czech Republic*

[f] *Department of Pathobiology, School of Veterinary Medicine, St. George's University, True Blue Campus, P.O. Box 7, Saint George, Grenada, West Indies*

Corresponding Authors:

* Pavel Štarha - pavel.starha@upol.cz

** René Lenobel - rene.lenobel@upol.cz

*** Jan Hošek - jan.hosek@vri.cz

Table of Contents

Discussion of *in vitro* cytotoxicity S3
Figure S1 - Structural formulas and HPLC traces for complexes **1–6** S4
Figure S2–S7 - Mass spectra (ESI+) of complexes **1–6** S5
Figure S8–S13 - ^{1}H and ^{31}P NMR spectra of complexes **1–6** S11
Figure S14 - X-ray crystal structures and non-covalent interactions of **3** and **3*** S17
Figure S15,S16 - UV-Vis, ^{1}H NMR and mass spectrometry stability studies S18
Figure S18–23 - HPLC stability studies in DMEM S21
Figure S24,S25 - Stability studies in the presence of biomolecules S27
Figure S26,S27 - Flow cytometry analysis of cell cycle and apoptosis S29
Figure S28 - Western blot analysis of apoptosis-related caspases and PARP S31
Figure S29 - Release of lactate dehydrogenase after the incubation with **3** and **6** S32
Proteome analysis discussion - general considerations S33
Figure S30 - Comparison of the effects of **3** and **6** on the proteome of A549 cells S33
Figure S31 - Volcano plot of proteomic differences between **3** and DMF samples S34
Proteome analysis discussion - primary target S34
Figure S32 - Gene Ontology enrichment analysis of proteins S35
Figure S33 - Effect of **3** on ribosomes S36
Proteome analysis discussion - nucleolus S37
Figure S34 - Effect of complex **3** on ribosome biogenesis in eukaryotes S39
Figure S35 - Effect of complex **3** on nucleocytoplasmic transport S40
Figure S36 - Effect of **3** on proteins included in ribosome biogenesis S41
Proteome analysis discussion - cellular entry S42
Figure S37,S38 - Effect of **3** on proteins in endocytosis pathway S42
Proteome analysis discussion - endolysosomal system S44
Figure S39,40 - Effect of **3** on proteins in lysozome organelle S46
Proteome analysis discussion - mitochondria S48
Figure S41,42 - Effect of **3** on proteins in respiratory chain and mitochondria S50
Proteome analysis discussion - endoplasmic reticulum S52
Figure S43,44 - Effect of **3** on proteins in endoplasmatic reticulum S53
Proteome analysis discussion - metabolic rewiring S55
Figure S45,S46 - Effect of **3** on proteins in metabolic remodeling S59
Figure S47 - Effect of **3** on aminoacyl-tRNA biosynthesis S61
Proteome analysis discussion - cell-cycle and apoptosis S62
Figure S48,S49 - Effect of **3** on proteins in cell cycle processes S65
Figure S50 - Effect of **3** on replication complex S67
Figure S51 - Effect of **3** on processes of mismatch repair of DNA S68
Figure S52 - Effect of **3** in intrinsic and extrinsic apoptotic pathways S69
Proteomics-based mechanistic summary S70
Figure S53 - Immunohistochemistry of cancer samples S71
Table S1,S2 - Crystallographic data for **3** and **3***·MeOH S72
Table S3 - Selected bond lengths and angles for **3** and **3*** S74
Table S4 - Summary of protein information from UniProt database S75
Table S5 - Search settings for protein identification and quantification S86
References S87

Results and discussion

***In vitro* cytotoxicity.** A series of new compounds **1–6** was synthesized using various cyclopentadienyl derived (Cp*, Cp^{ph}) and N,P-donor ligands - a combination rarely explored in the field of anticancer iridium-cyclopentadienyl complexes. It has been well demonstrated on multiple examples of Ir-Cp^x compounds that Cp-ring extension by phenolic groups often results in increased cytotoxic activity.[1–3] Interestingly, a similar tendency was observed for the compounds reported herein, but only for Cp* compounds **1** and **2**, which were less active than their Cp^{ph} analogues **4** and **5**. On the other hand, the L3-containing Cp* compound **3** was more effective toward the MOR/CPR CDDP-resistant lung carcinoma cells (IC_{50} = 3.1 μM) than its Cp^{ph} analogue **6** (IC_{50} = 6.2 μM) and CDDP (IC_{50} > 20 μM). These results suggest that extending the Cp ring does not necessarily ensure higher activity of Ir-Cp^x compounds, as formerly reported for their representatives.[4,5] Furthermore, for the applied design of **1–6**, it seems that the chelate-ring size has an effect on the resulting biological activity, especially for Cp* compounds, where **3** (with a six-membered ring) exceeded the potency of **2** (with a five-membered).[6] The effect of the N,P-donor set cannot be discussed with direct analogues of **1–6** derived from, *e.g.*, *N,N*-donor ligands since similar Ir compounds containing such ligands have not yet been reported. Close Ir-Cp^x analogues involving *N,N*-donor ethane-1,2-diamine or propane-1,3-diamine were reported to be inactive or markedly less effective than CDDP in various human cancer cells.[7,8]

Regarding the potency of structurally similar ionic chlorido Ir–Cp* complexes involving chelating *N,N*-donor ligands against human lung cancer cells, this can be demonstrated by the widely studied derivatives of 2,2′-bipyridine. To date, numerous structurally related Ir(III) compounds of the general formula $[Ir(\eta^5\text{-}Cp^*)Cl(L)]^+$ have been synthesized and evaluated, exhibiting a broad range of relative activities compared to cisplatin. Several reported compounds show higher potency against the A549 cell line than cisplatin (e.g. IC_{50} = 4.8 μM and RA = 4.4 for L = (*E*)-4-methoxy-*N*-(4-methoxyphenyl)-*N*-{4-[2-(4′-methyl-[2,2′-bipyridin]-4-yl)vinyl]phenyl}anilin),[9] while others exhibit lower activity (e.g. IC_{50} >50 μM and RA < 1 for L = 4-amino-*N*-(2,2′-bipyridyn-5-yl)benzenesulfonamide).[10] In other words, the structural motif of Ir–Cp^x compounds cannot be considered inherently cytotoxic, rather, the resulting activity depends on a multitude of factors that remain difficult to predict.

High activity has been observed in Ir–Cp^x compounds featuring ancillary ligands other than *N,N*-donors (e.g. IC_{50} = 2.2 μM and RA = 4.0 for $[Ir(\eta^5\text{-}Cp^*)Cl(pbpn)]$; pbpn = *C,N*-coordinated 4,9,16-triazadibenzo[a,c]naphthacene), monodentate ligands other than chloride (e.g. IC_{50} = 0.15 μM and RA = 59.3 for $[Ir(\eta^5\text{-}Cp^*)(bzim)(pbpn)]^+$; bzim = *N*-benzylimidazole)[11] or when evaluated against different sets of cancer cell lines (e.g. IC_{50} = 2.0 μM and RA = 42.0 for $[Ir(\eta^5\text{-}Cp^*)Cl(nobpy)]PF_6$; nobpy = 4,4′-dinonyl-2,2′-bipyridine).[12] Further improvements in cytotoxicity can be achieved through Cp^x ring extension.[7,9,13,14] In this context, for example the compound $[Ir(\eta^5\text{-}Cp^{bph})(bphen)Cl]PF_6$ exhibited, similarly to the aforementioned compound $[Ir(\eta^5\text{-}Cp^*)(bzim)(pbpn)]^+$, nanomolar activity in A549 human lung cancer cells (IC_{50} = 0.09 μM and RA = 22.2); bphen = 4,7-diphenyl-1,10-phenanthroline.[14] Despite its extreme cytotoxicity, this compound did not exhibit enhanced activity against the cisplatin-resistant A549 cell line (RF = 0.9; A549 model), in contrast to the results discussed above for compound **3** (RF = 0.4; MOR/CPR model); RF = Resistance Factor (calculated as IC_{50}(resistant cell line)/IC_{50}(sensitive cell line)).

Compounds **1–6** represent direct analogues of $[Rh(\eta^5\text{-}Cp^x)Cl(L1\text{-}3)]PF_6$ complexes involving the same phosphinoalkylamines (L1–3).[15] In contrast to the Ir complexes studied in the present study, the Rh analogues showed only moderate antiproliferative activity in A549 lung cancer cells but were more potent in A2780 ovarian carcinoma or MOLT-4 acute lymphoblastic leukemia.

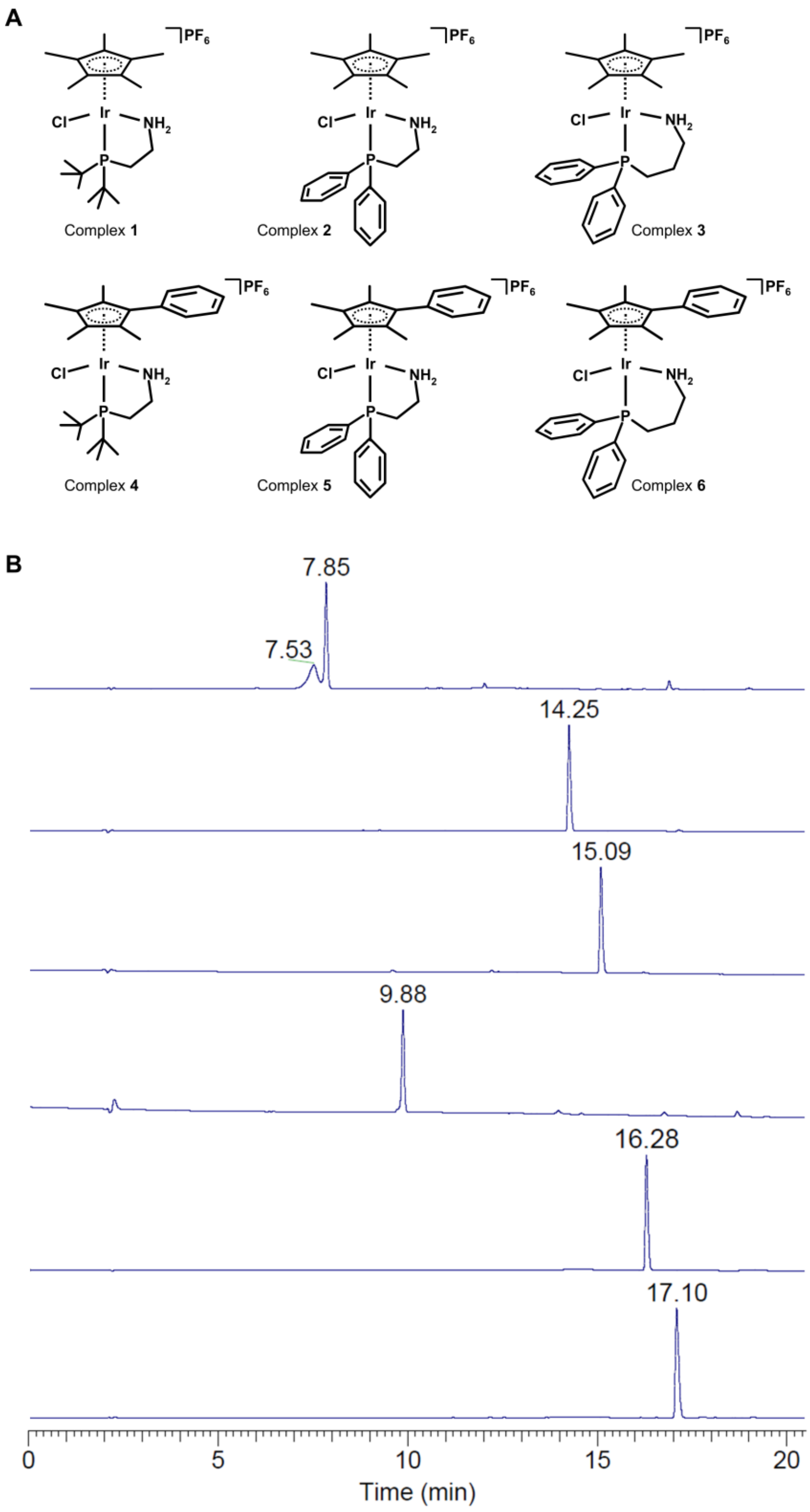

Figure S1. Structural formulas (**A**) and High Performance Liquid Chromatography (HPLC) traces (**B**) of complexes **1–6**.

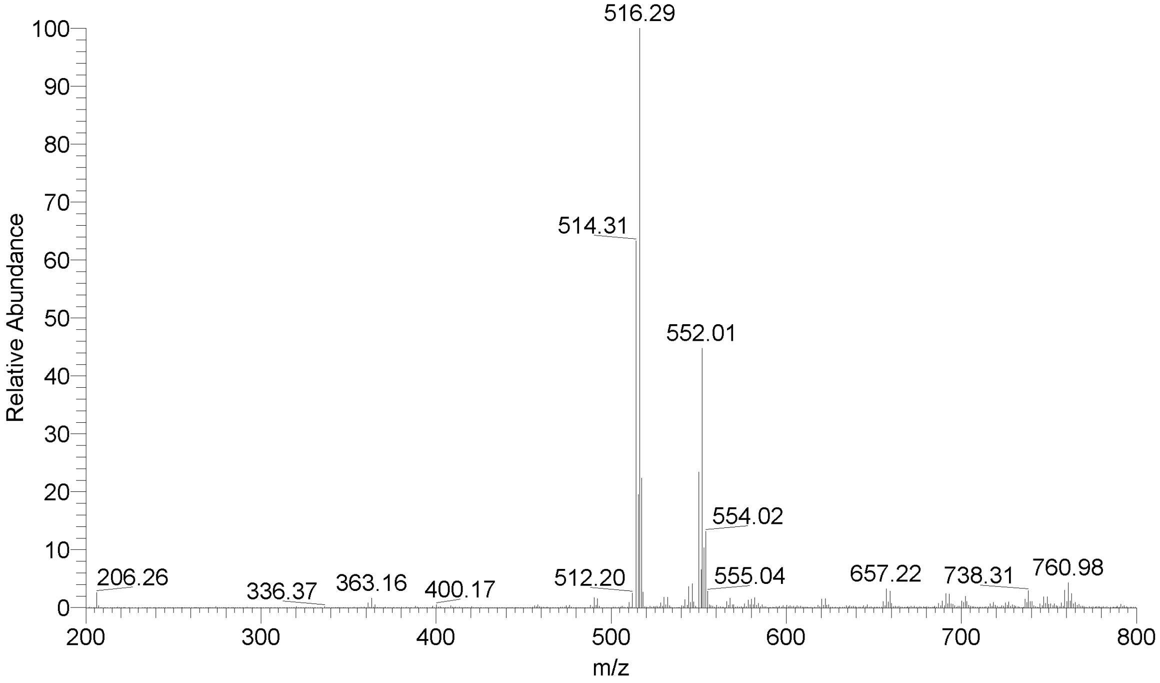

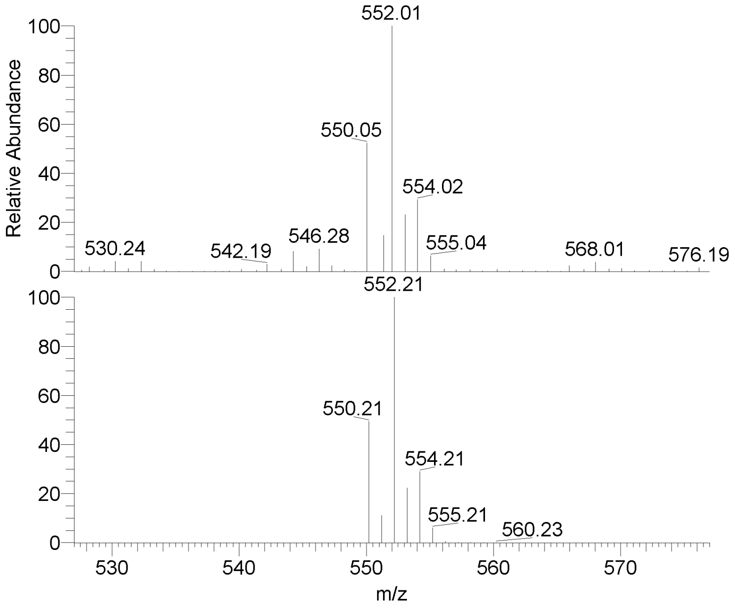

Figure S2. ESI+ mass spectrum of complex **1** (*top*), given with a comparison of the experimental and theoretical isotopic pattern calculated for the $[IrCl(Cp^*)(L1)]^+$ species (*bottom*). ESI+ = positive electrospray ionization mode. Complex **1** was dissolved in MeOH.

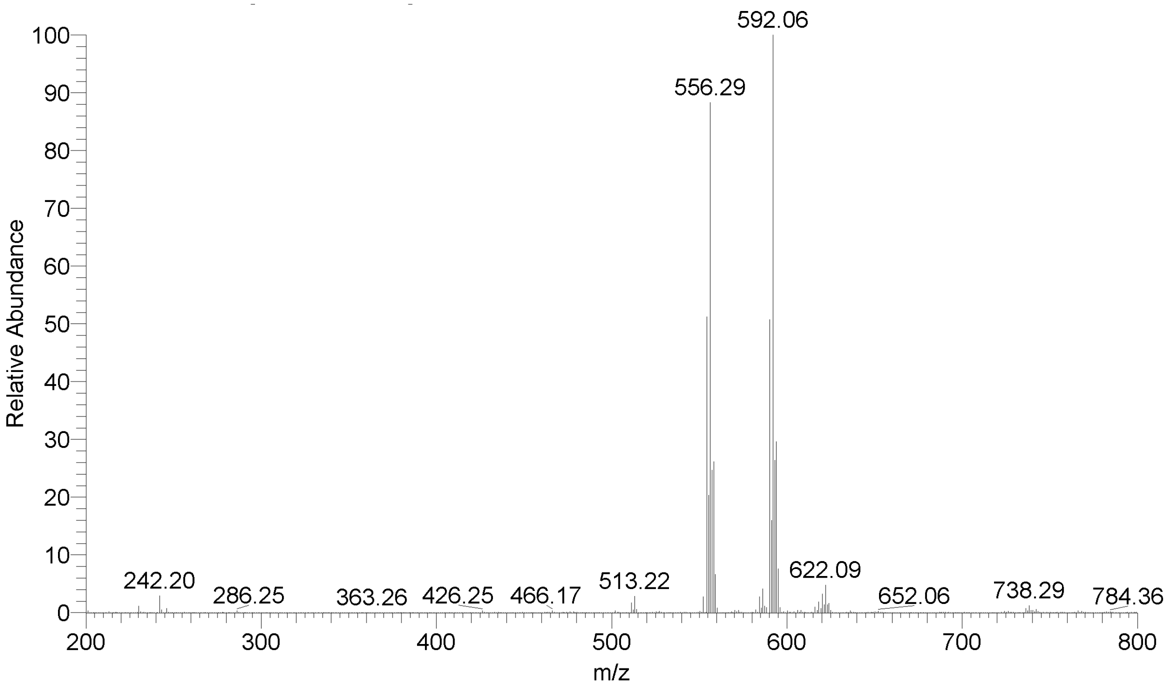

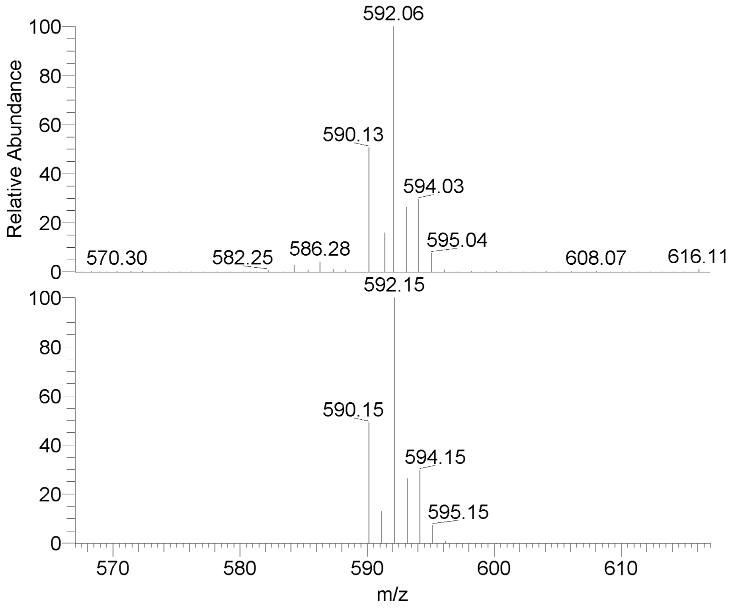

Figure S3. ESI+ mass spectrum of complex **2** (*top*), given with a comparison of the experimental and theoretical isotopic pattern calculated for the $[IrCl(Cp^*)(L2)]^+$ species (*bottom*). ESI+ = positive electrospray ionization mode. Complex **2** was dissolved in MeOH.

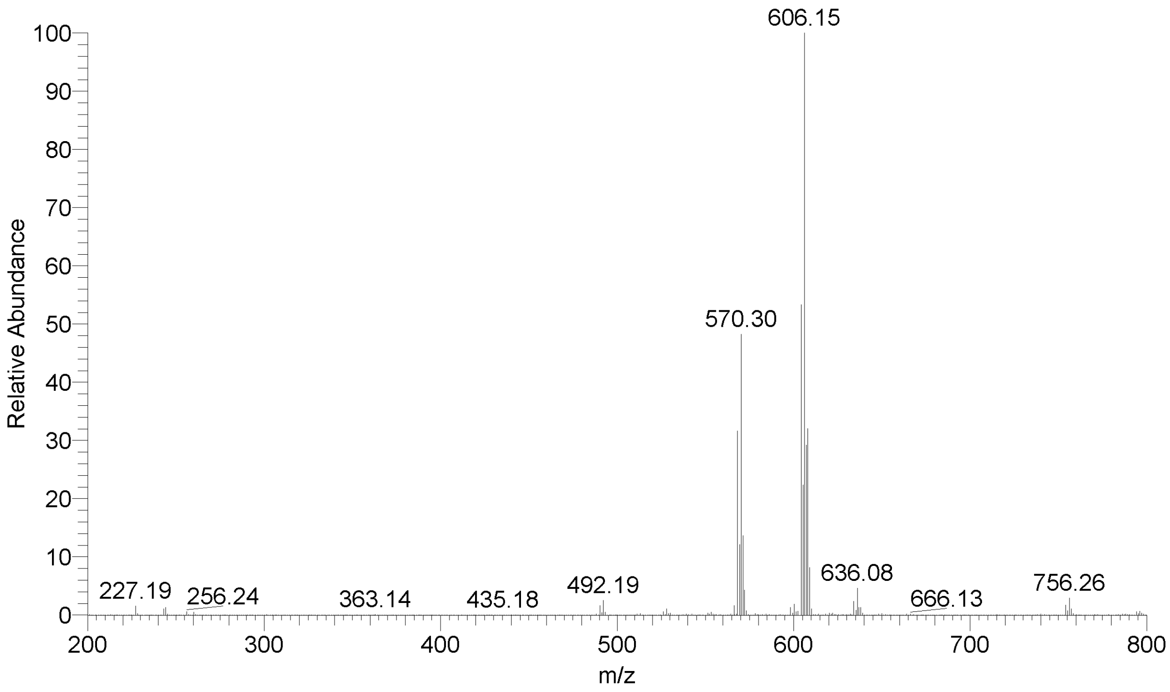

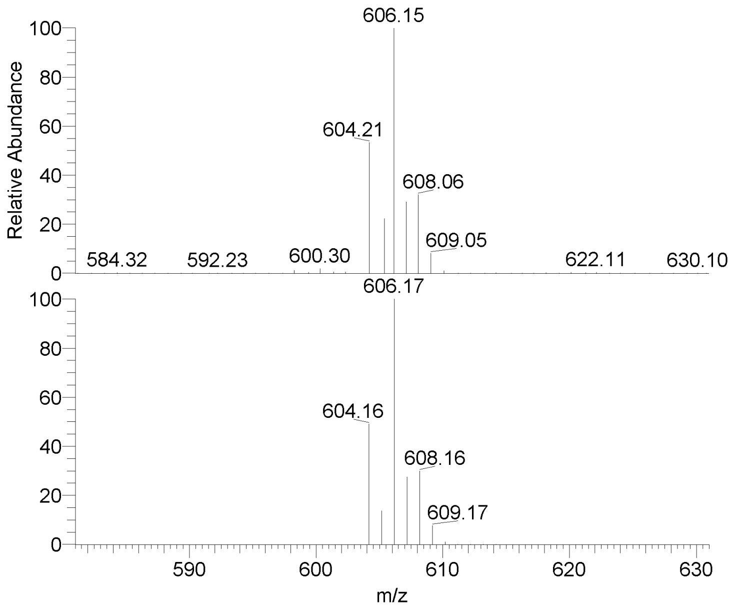

Figure S4. ESI+ mass spectrum of complex **3** (*top*), given with a comparison of the experimental and theoretical isotopic pattern calculated for the [IrCl(Cp*)(L3)]$^{+}$ species (*bottom*). ESI+ = positive electrospray ionization mode. Complex **3** was dissolved in MeOH.

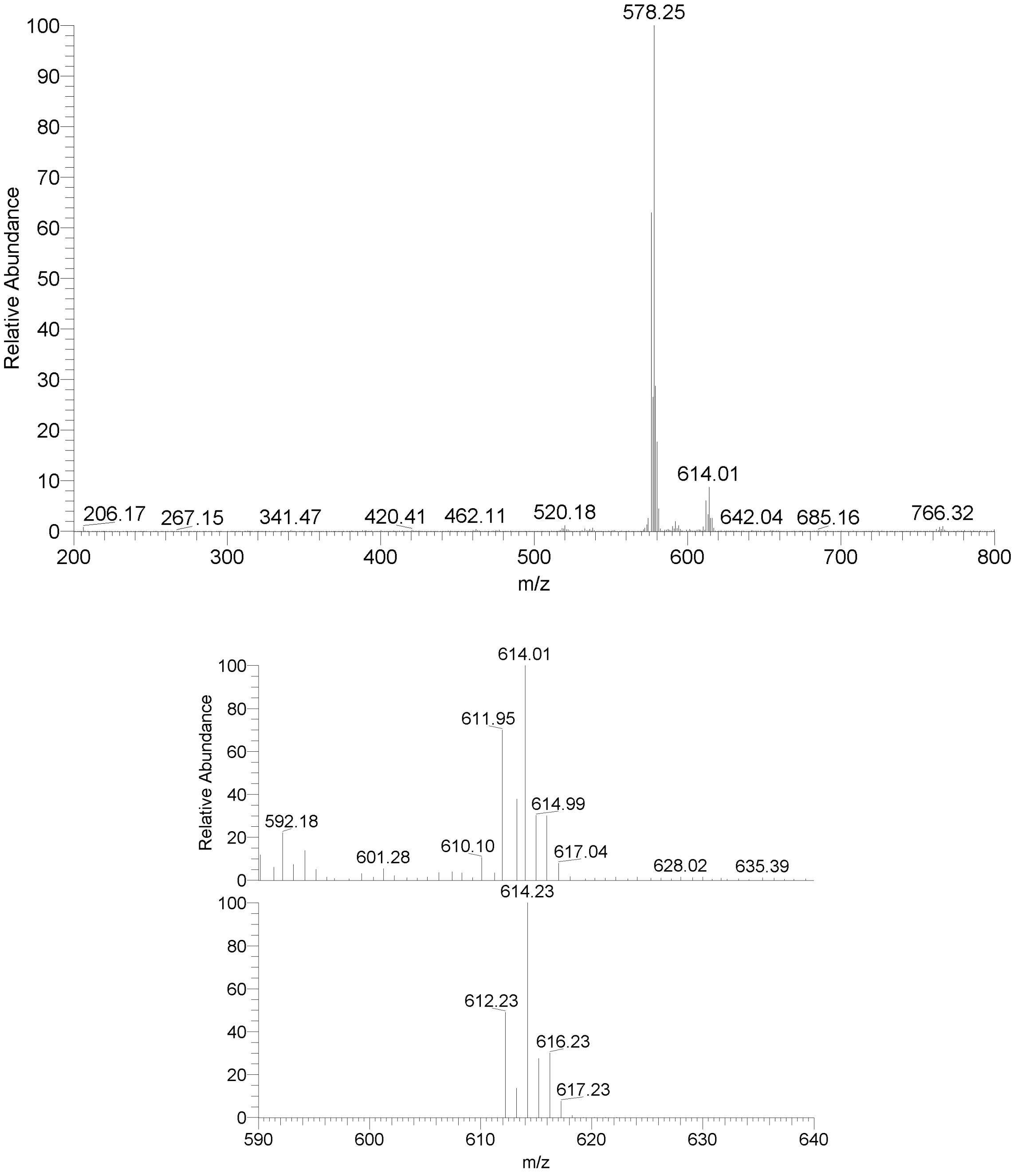

Figure S5. ESI+ mass spectrum of complex **4** (*top*), given with a comparison of the experimental and theoretical isotopic pattern calculated for the $[IrCl(Cp^{ph})(L1)]^{+}$ species (*bottom*). ESI+ = positive electrospray ionization mode. Complex **4** was dissolved in MeOH.

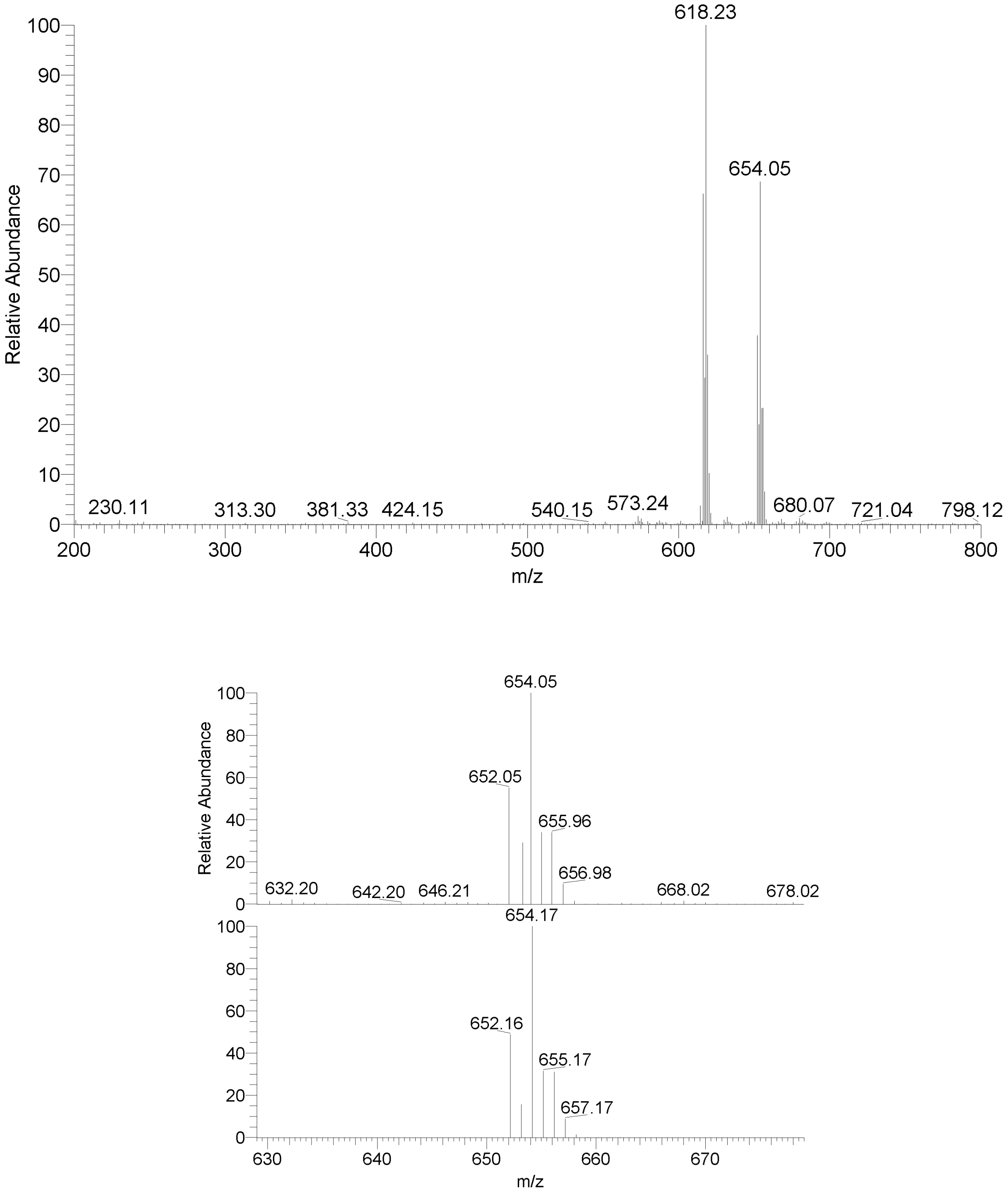

Figure S6. ESI+ mass spectrum of complex **5** (*top*), given with a comparison of the experimental and theoretical isotopic pattern calculated for the $[IrCl(Cp^{ph})(L2)]^{+}$ species (*bottom*). ESI+ = positive electrospray ionization mode. Complex **5** was dissolved in MeOH.

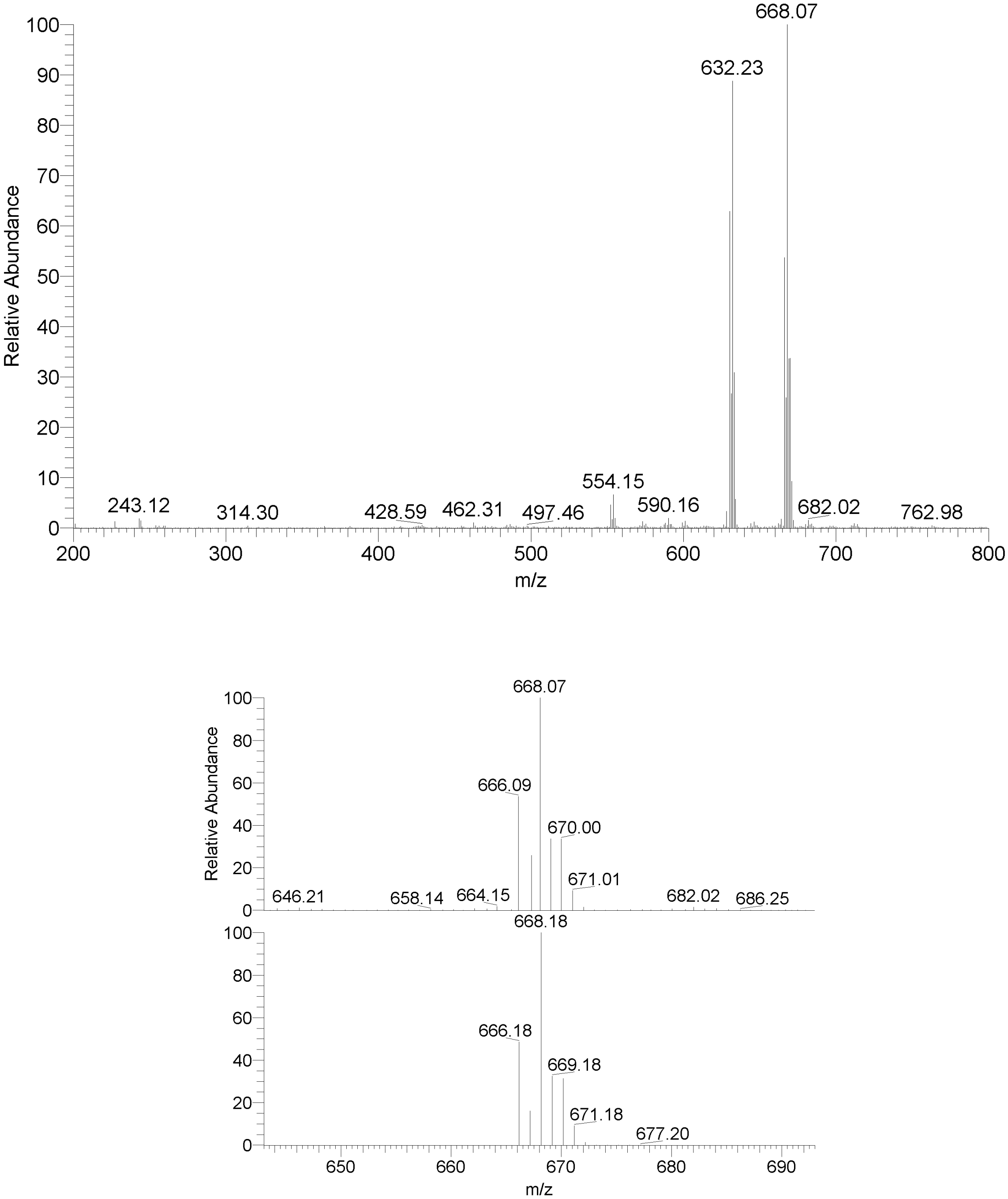

Figure S7. ESI+ mass spectrum of complex **6** (*top*), given with a comparison of the experimental and theoretical isotopic pattern calculated for the $[IrCl(Cp^{ph})(L3)]^+$ species (*bottom*). ESI+ = positive electrospray ionization mode. Complex **6** was dissolved in MeOH.

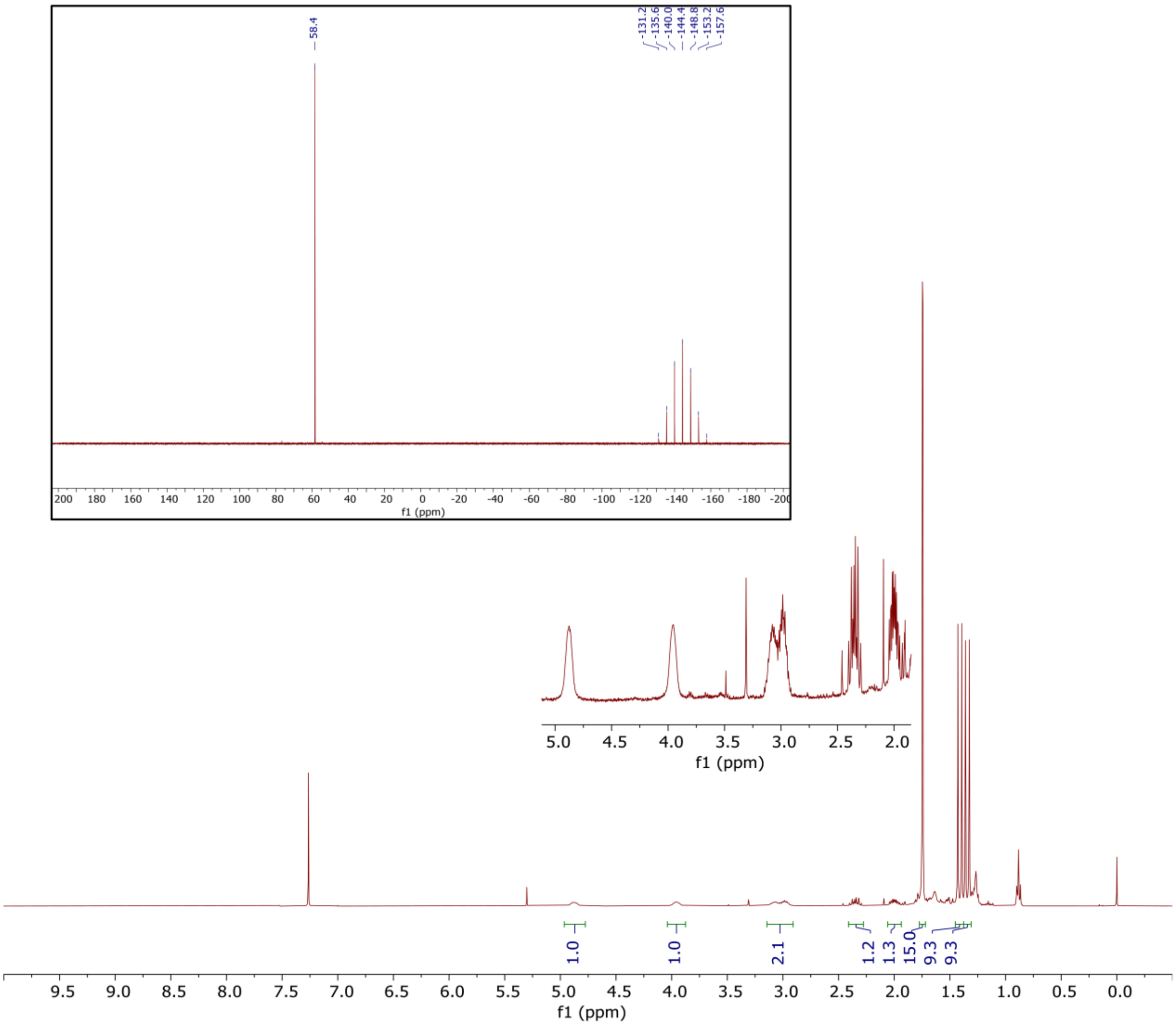

Figure S8. ^{1}H (*bottom*) and ^{31}P (*top*) NMR spectra of complex **1** (dissolved in $CDCl_3$).

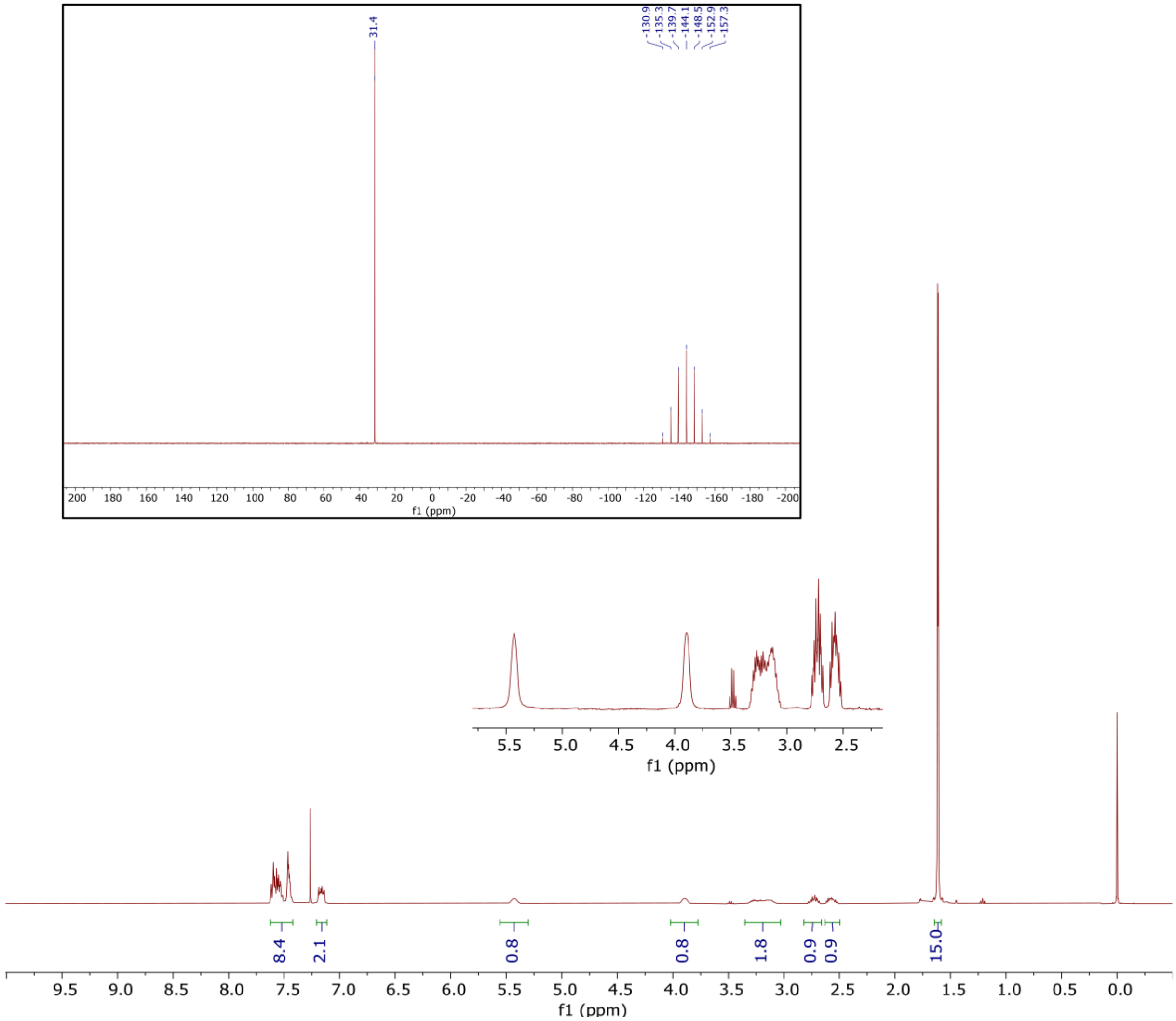

Figure S9. ^{1}H (*bottom*) and ^{31}P (*top*) NMR spectra of complex **2** (dissolved in $CDCl_3$).

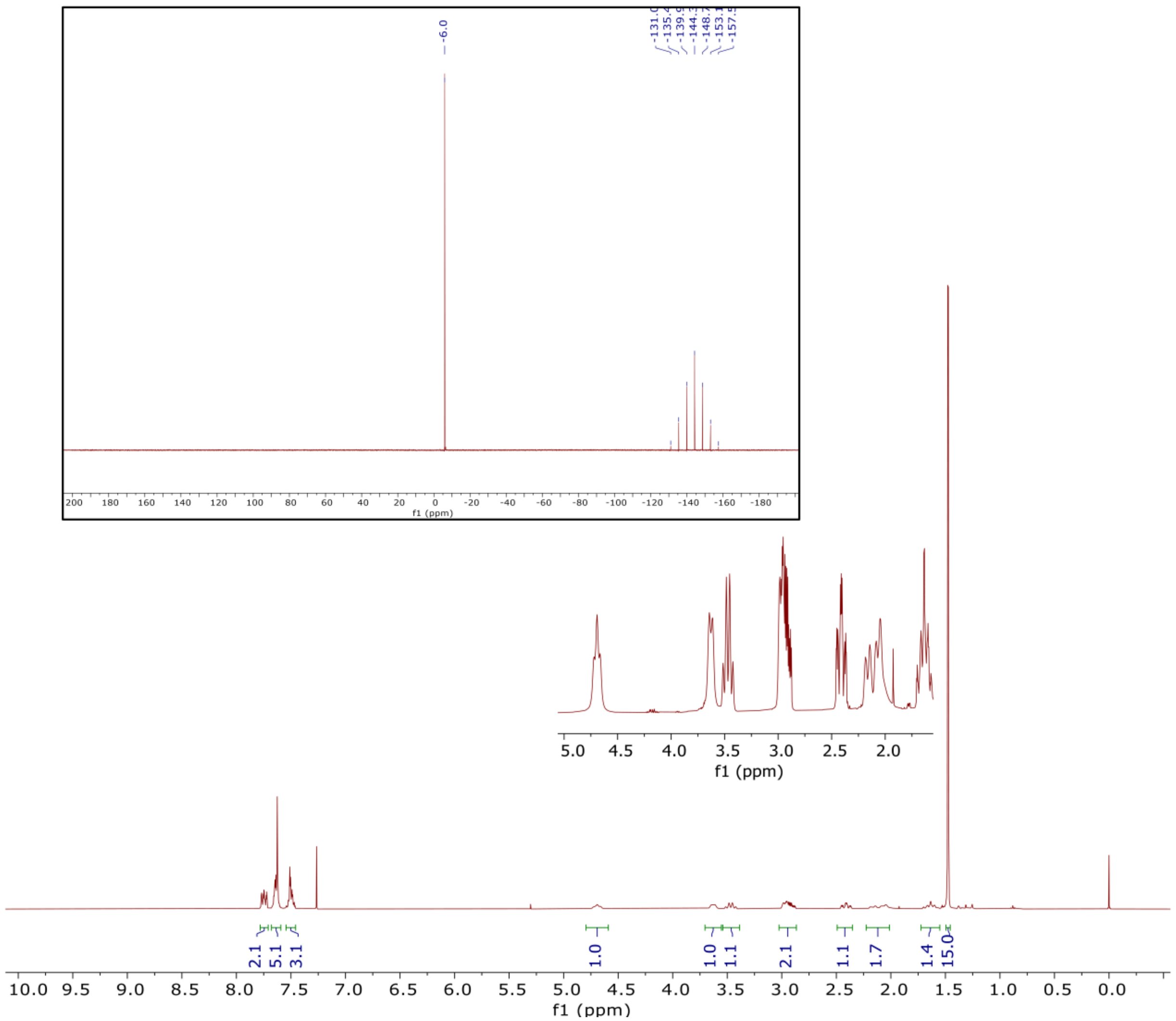

Figure S10. ^{1}H (*bottom*) and ^{31}P (*top*) NMR spectra of complex **3** (dissolved in $CDCl_3$).

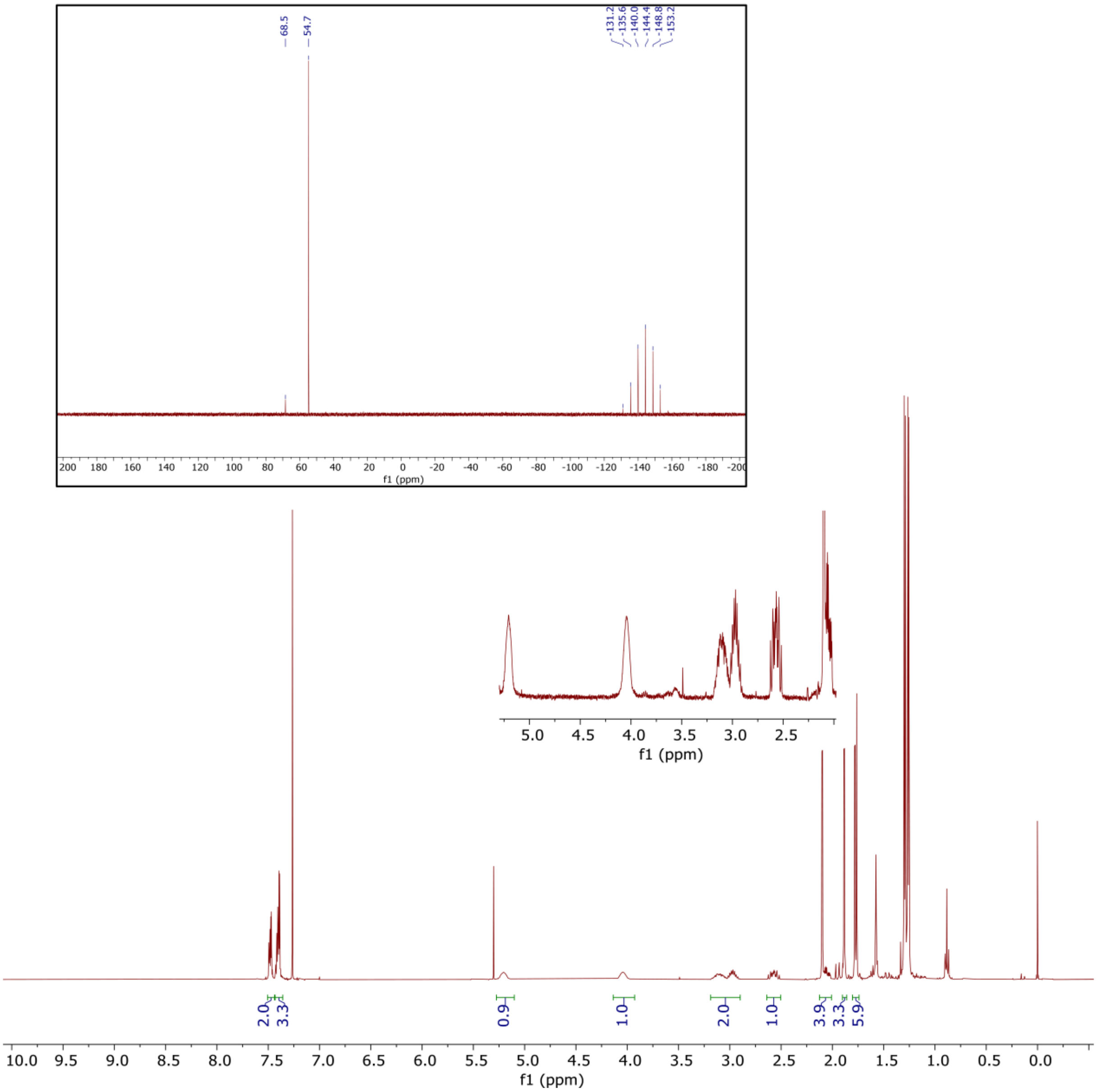

Figure S11. ^{1}H (*bottom*) and ^{31}P (*top*) NMR spectra of complex **4** (dissolved in $CDCl_3$).

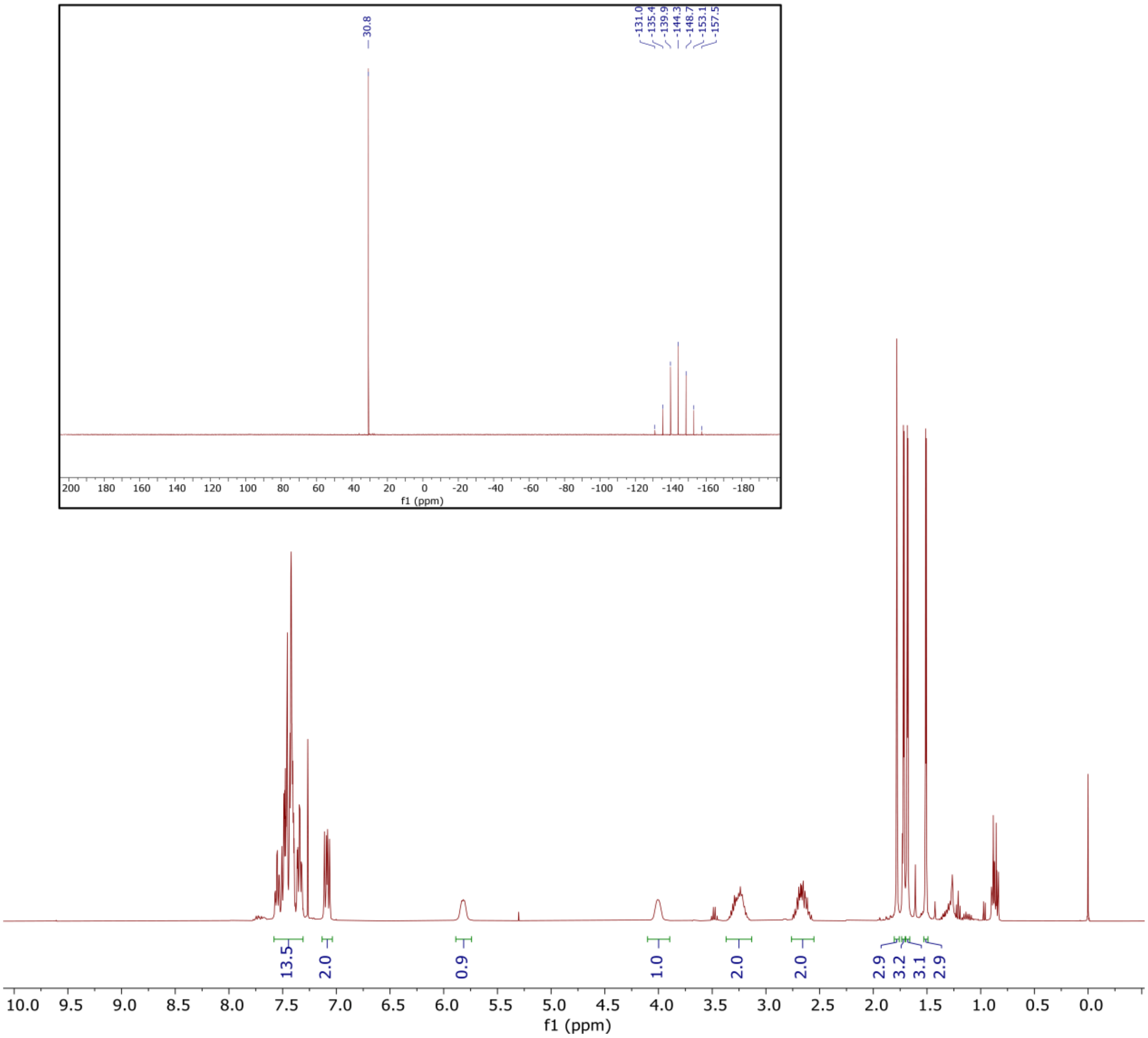

Figure S12. ^{1}H (*bottom*) and ^{31}P (*top*) NMR spectra of complex **5** (dissolved in $CDCl_3$).

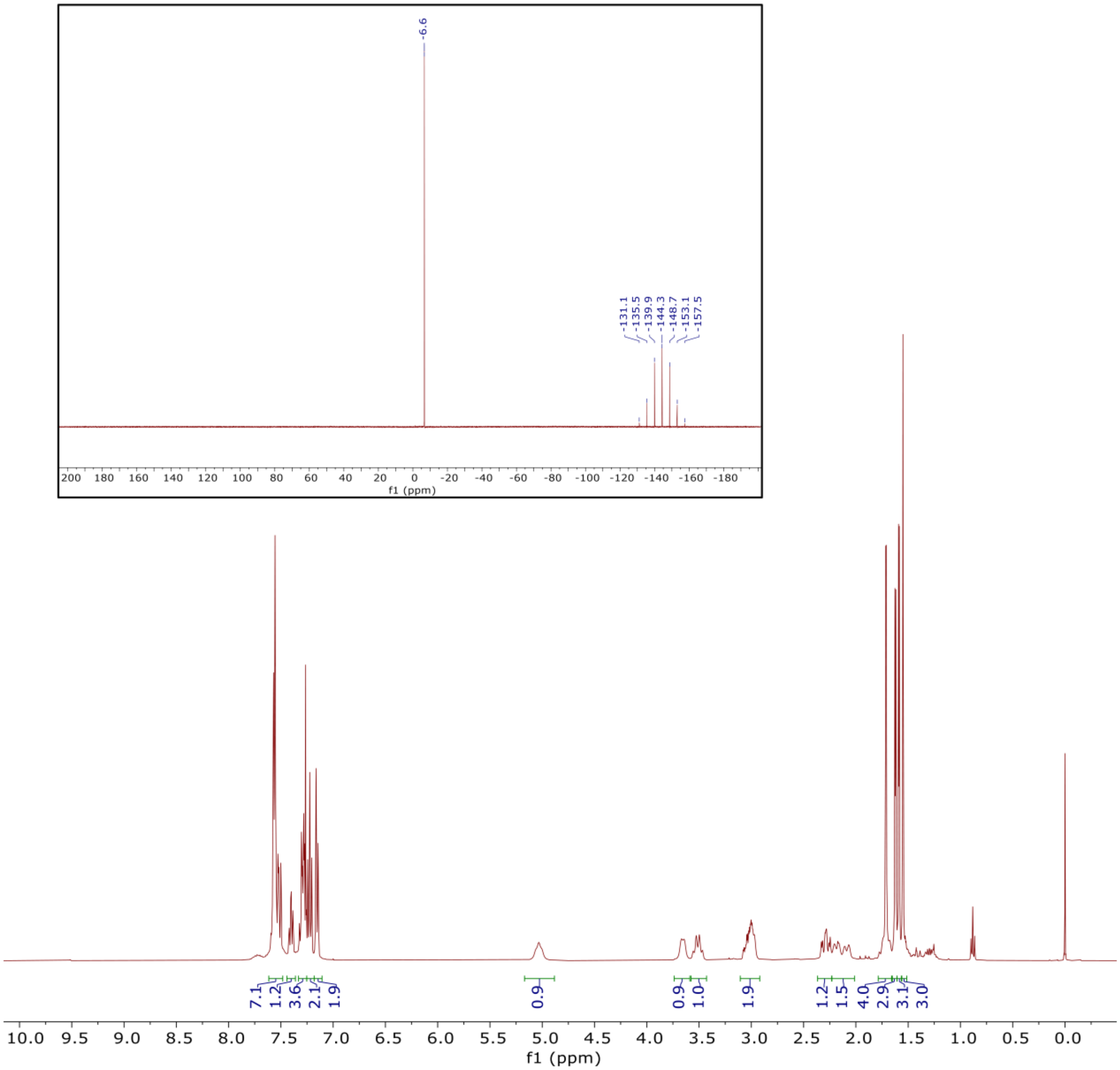

Figure S13. ^{1}H (*bottom*) and ^{31}P (*top*) NMR spectra of complex **6** (dissolved in $CDCl_3$).

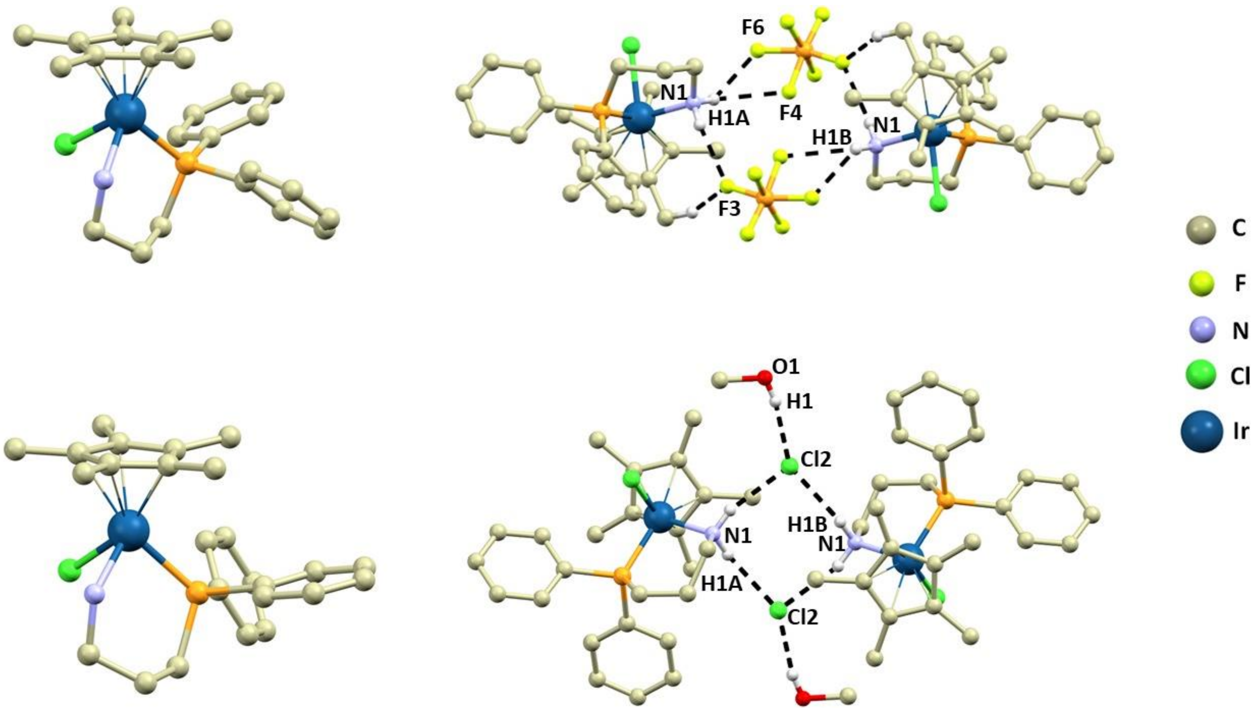

Figure S14. The molecular structure of the complex cation in **3** (*left above*). A perspective view on the main structural motif in the crystal structure of **3** (*right above*). The molecular structure of the complex cation in **3*** (*left below*). A perspective view on the main structural motif in the crystal structure of **3*** (*right below*). The hydrogen atoms except for those involved in hydrogen bonding (black dashed lines) were omitted for clarity. Donor···acceptor distances of selected non-covalent contacts (in Å): **3**: *d*(N1H1A···F4) = 3.23(2), *d*(N1H1A···F6) = 3.14(1), *d*(N1H1B···F3) = 3.12(1), **3***: *d*(N1H1A···Cl2) = 3.344(4), *d*(N1H1B···Cl2) = 3.256(4), *d*(O1H1···Cl2) = 3.121(5).

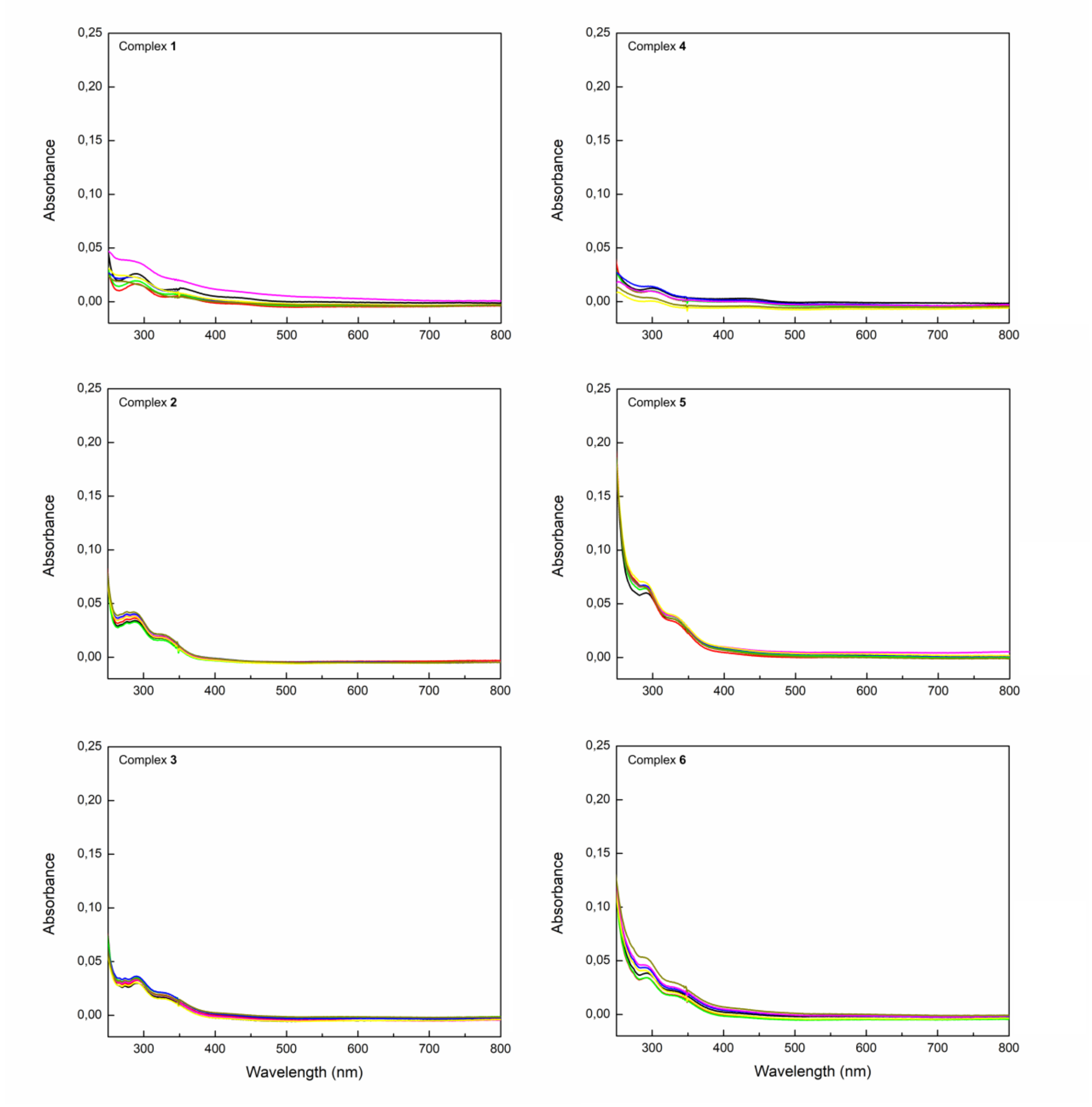

Figure S15. UV-Vis stability studies of complexes **1**–**6** (5 μM final concentration) in 5% DMF/95% PBS in H_2O.

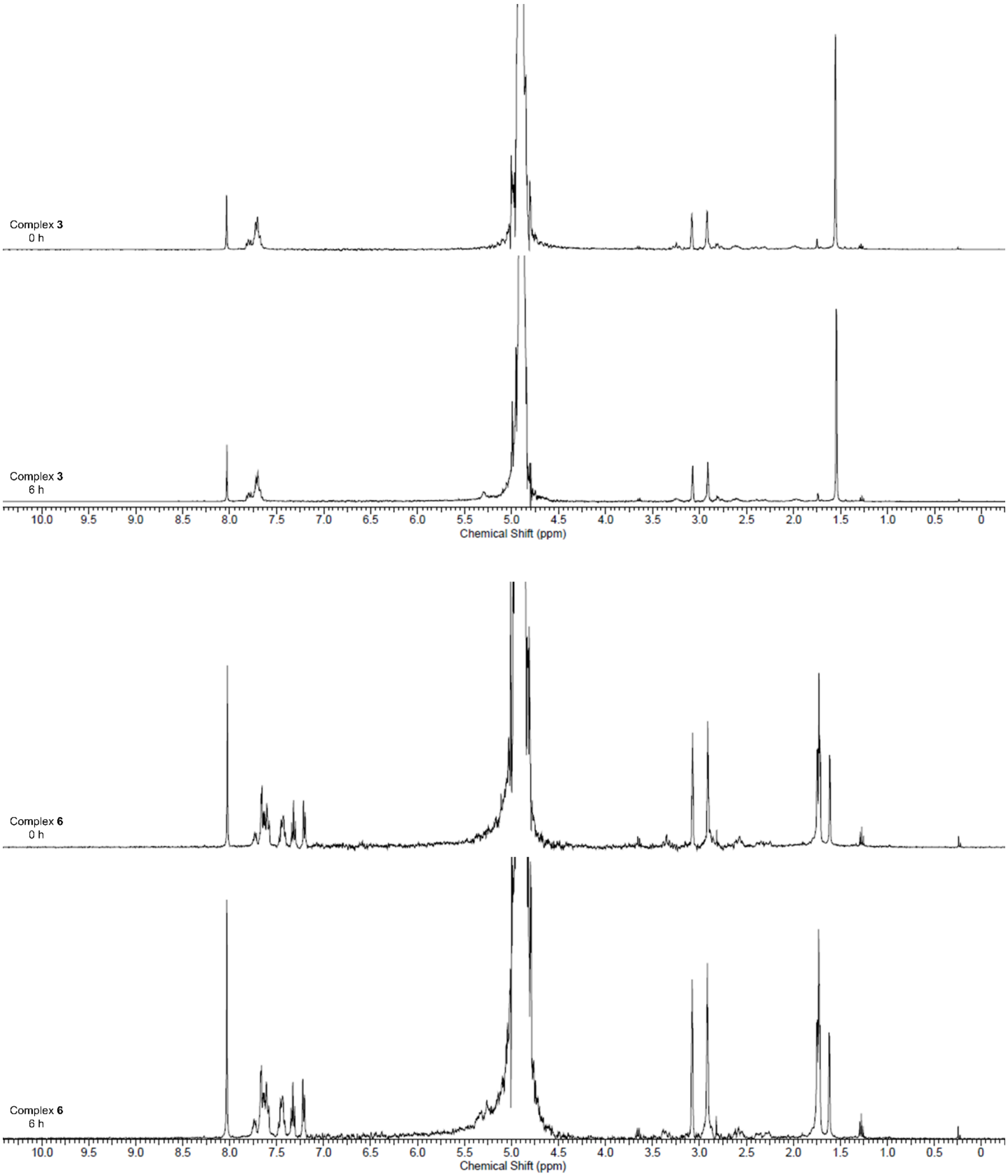

Figure S16. ^{1}H NMR stability studies of **3** and **6** (1 mM final concentration) in 10% DMF-d_7/90% PBS in D_2O (pH 7.4).

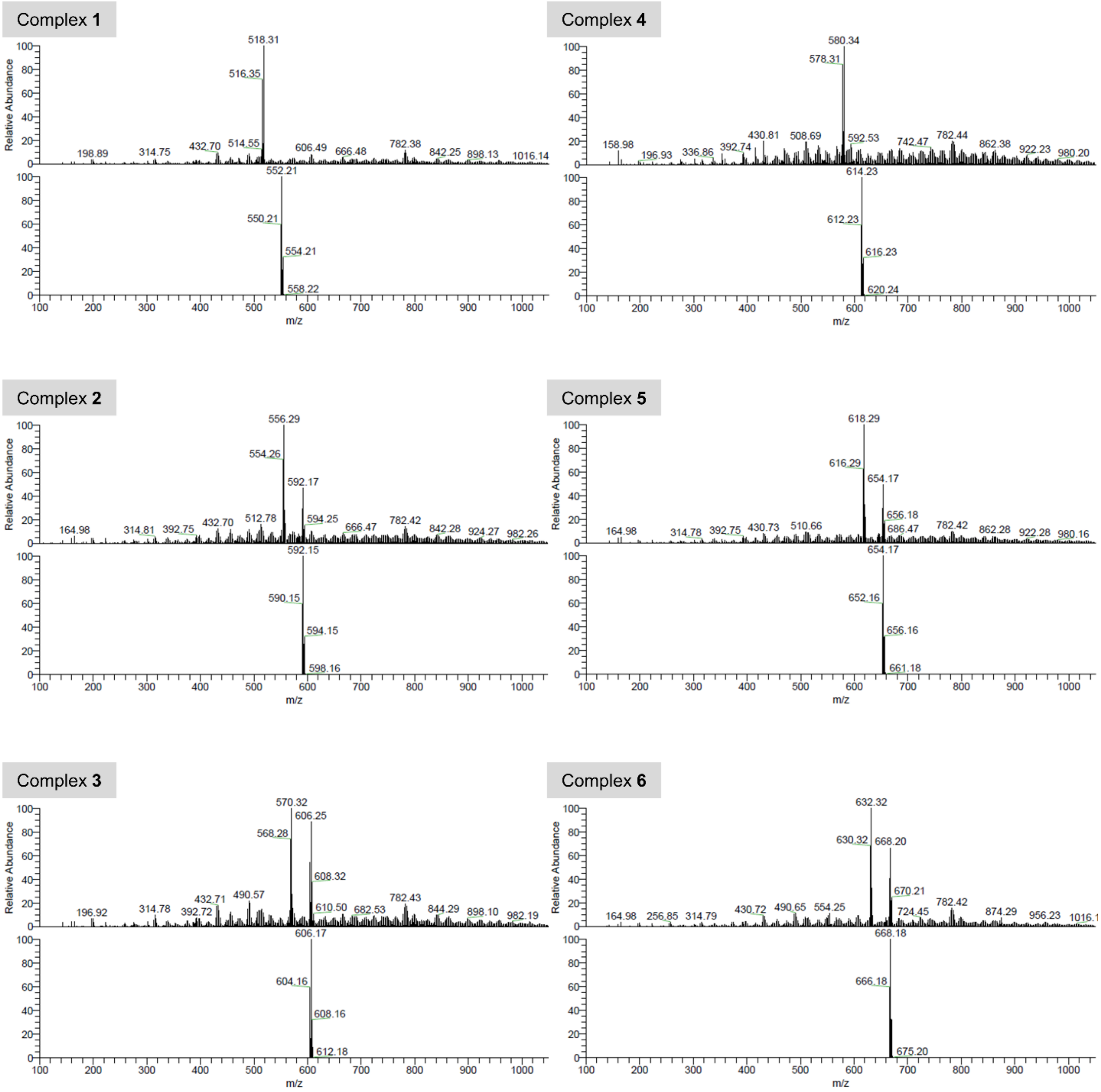

Figure S17. ESI+ mass spectra of complexes **1–6** (5 µM final concentration) incubated for 24 h in 5% DMF/95% PBS in H_2O. The spectra are given as experimental (top) and theoretical calculated for the $[IrCl(Cp^x)(L)]^+$ chlorido species (bottom).

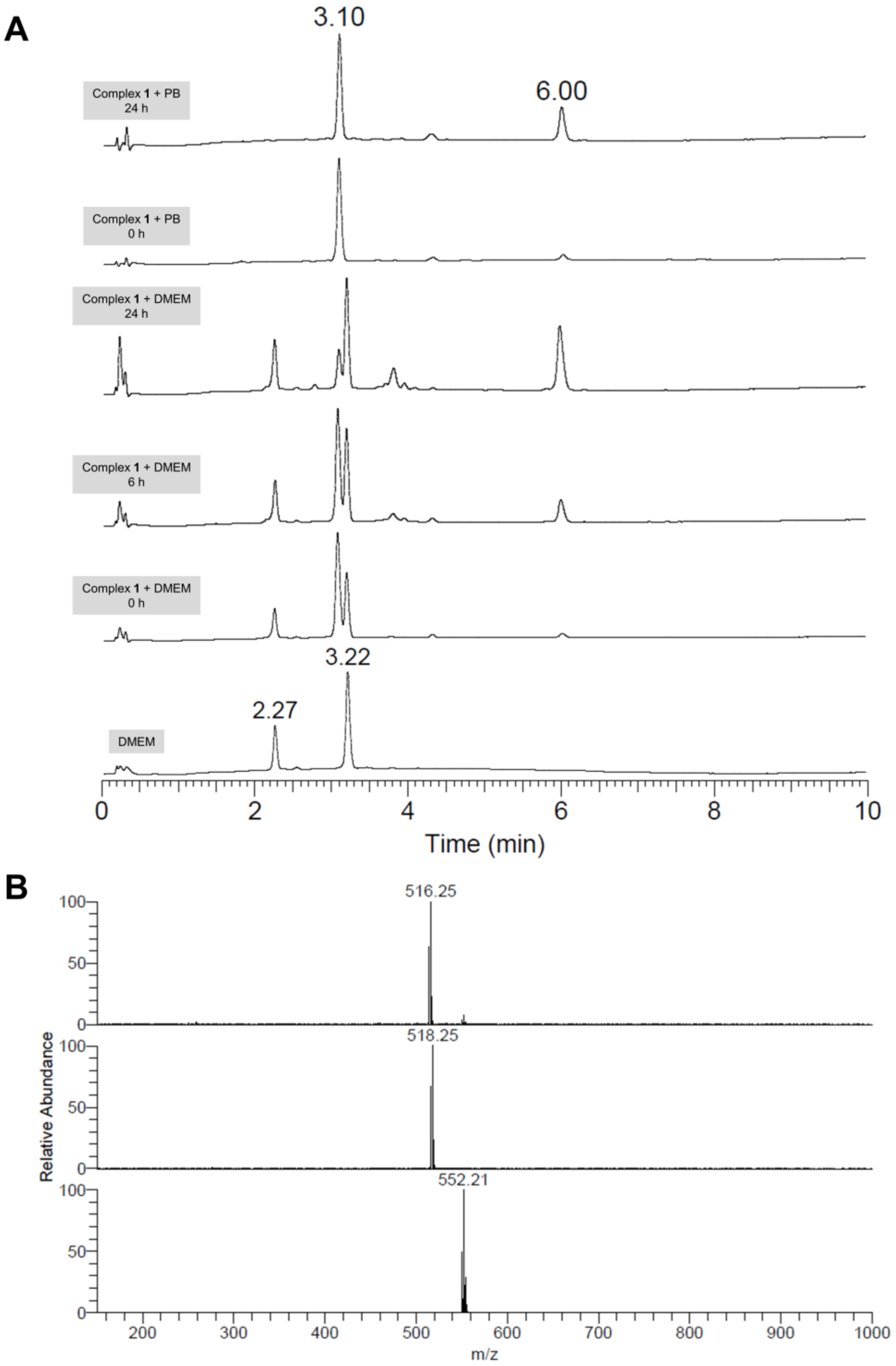

Figure S18. (A) HPLC traces for the complex **1** (50 µM final concentration) incubated for 24 h in 10% DMF/90% DMEM (without serum) or in 10% DMF/90% PBS in H_2O. (B) Experimental ESI+ mass spectrum for the HPLC peak centered at t_R = 3.10 min (top) and 6.00 min (middle), and theoretical mass spectrum calculated for the $[IrCl(Cp^*)(L1)]^+$ species (bottom).

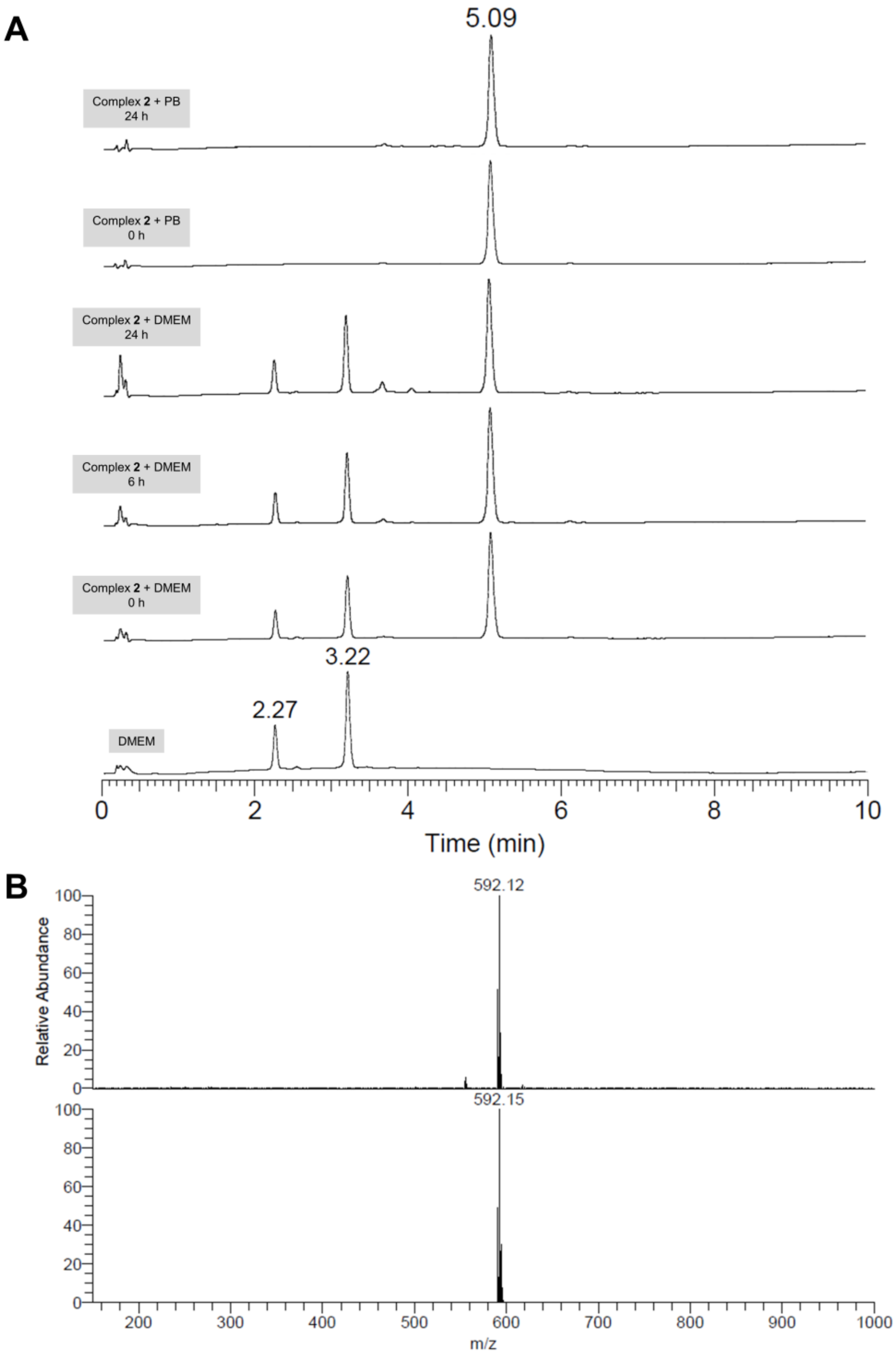

Figure S19. (A) HPLC traces for the complex **2** (50 μM final concentration) incubated for 24 h in 10% DMF/90% DMEM (without serum) or in 10% DMF/90% PBS in H_2O. (B) Experimental ESI+ mass spectrum for the HPLC peak centered at t_R = 5.09 min (top) and theoretical mass spectrum calculated for the $[IrCl(Cp^*)(L2)]^+$ species (bottom).

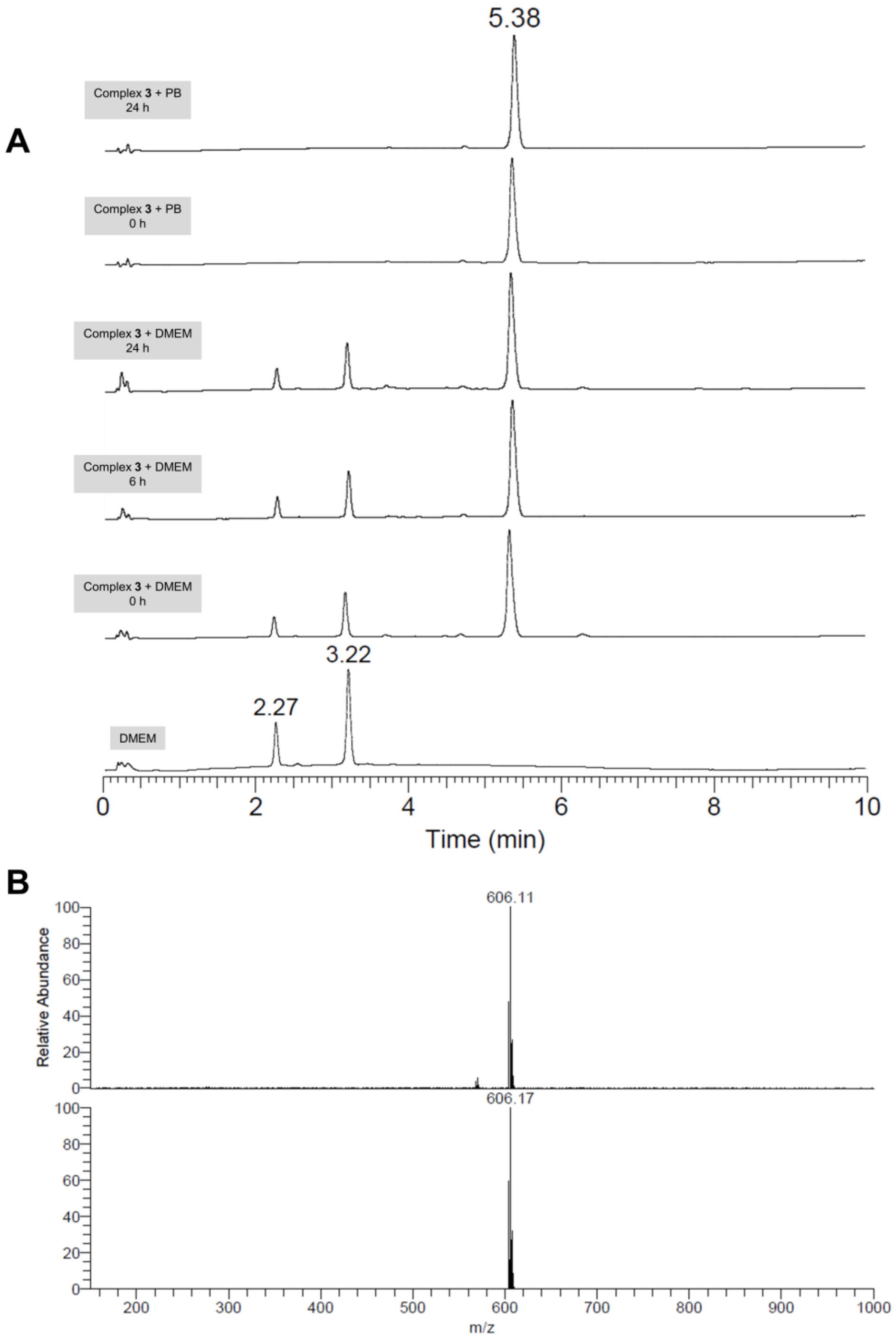

Figure S20. (A) HPLC traces for the complex **3** (50 µM final concentration) incubated for 24 h in 10% DMF/90% DMEM (without serum) or in 10% DMF/90% PBS in H_2O. (B) Experimental ESI+ mass spectrum for the HPLC peak centered at t_R = 5.38 min (top) and theoretical mass spectrum calculated for the $[IrCl(Cp^*)(L3)]^+$ species (bottom).

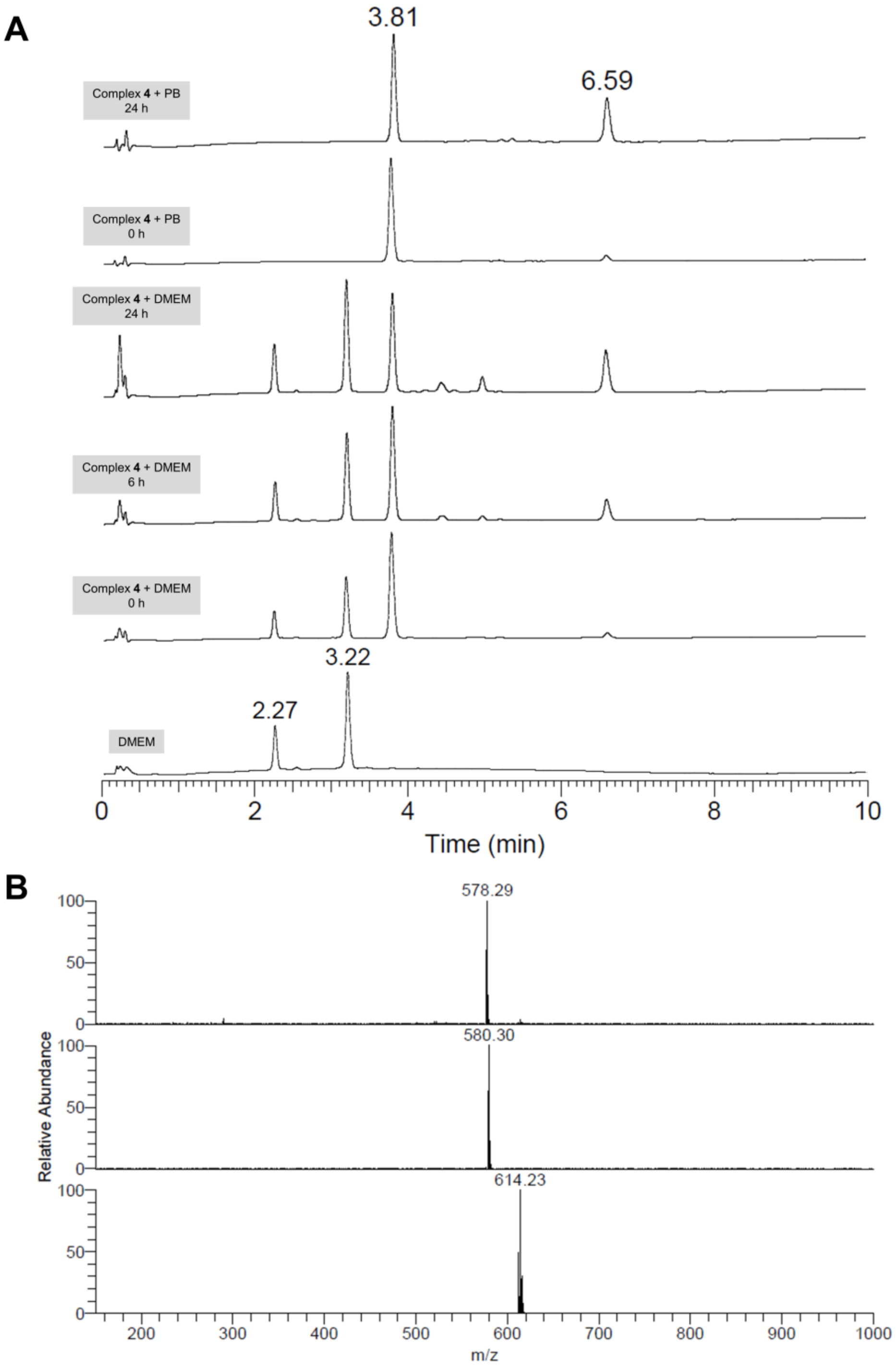

Figure S21. (A) HPLC traces for the complex **4** (50 μM final concentration) incubated for 24 h in 10% DMF/90% DMEM (without serum) or in 10% DMF/90% PBS in H_2O. (B) Experimental ESI+ mass spectrum for the HPLC peak centered at t_R = 3.81 min (top) and 6.59 min (middle), and theoretical mass spectrum calculated for the $[IrCl(Cp^{ph})(L1)]^+$ species (bottom).

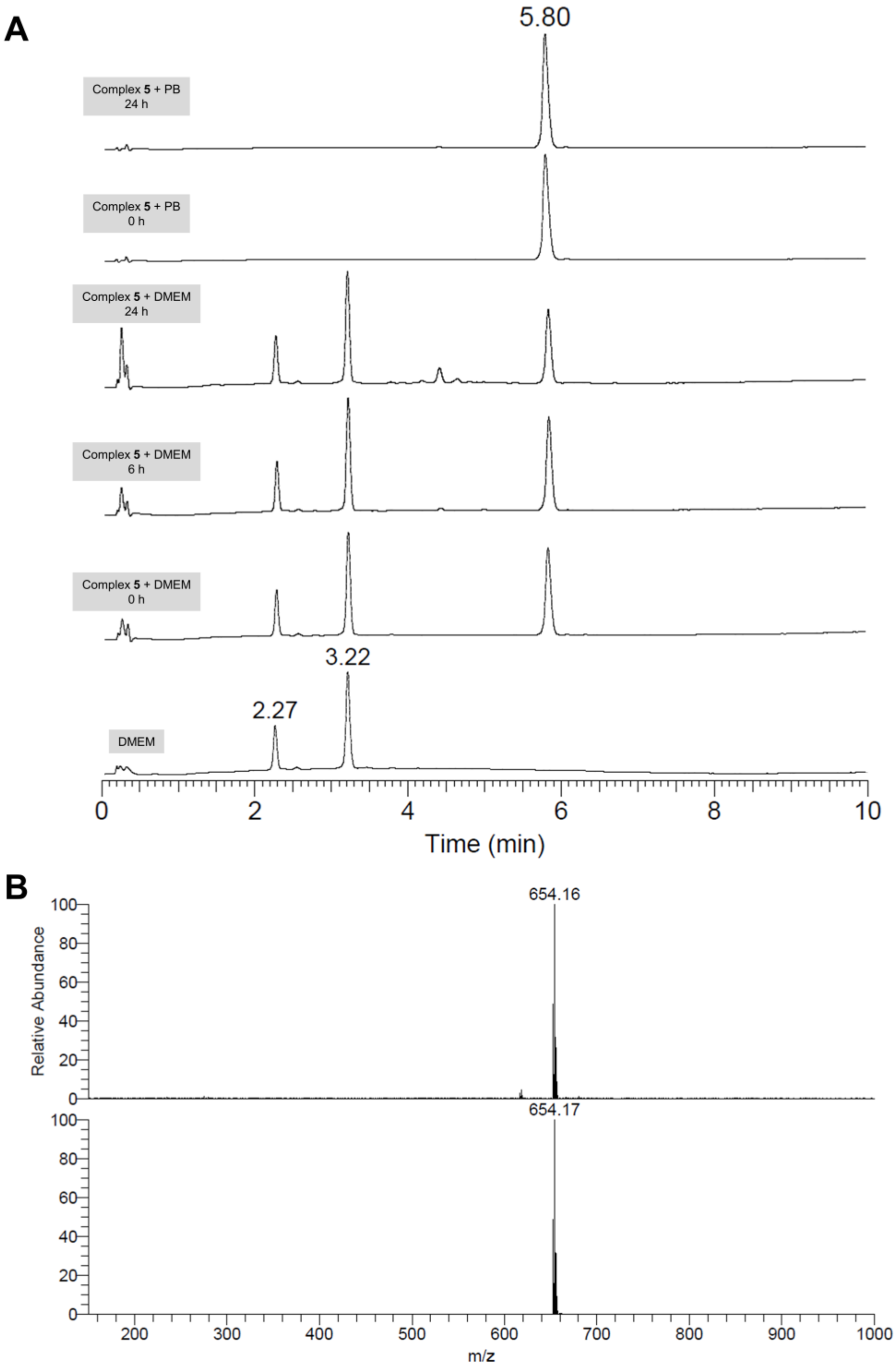

Figure S22. (A) HPLC traces for the complex **5** (50 μM final concentration) incubated for 24 h in 10% DMF/90% DMEM (without serum) or in 10% DMF/90% PBS in H_2O. (B) Experimental ESI+ mass spectrum for the HPLC peak centered at t_R = 5.80 min (top) and theoretical mass spectrum calculated for the $[IrCl(Cp^{ph})(L2)]^+$ species (bottom).

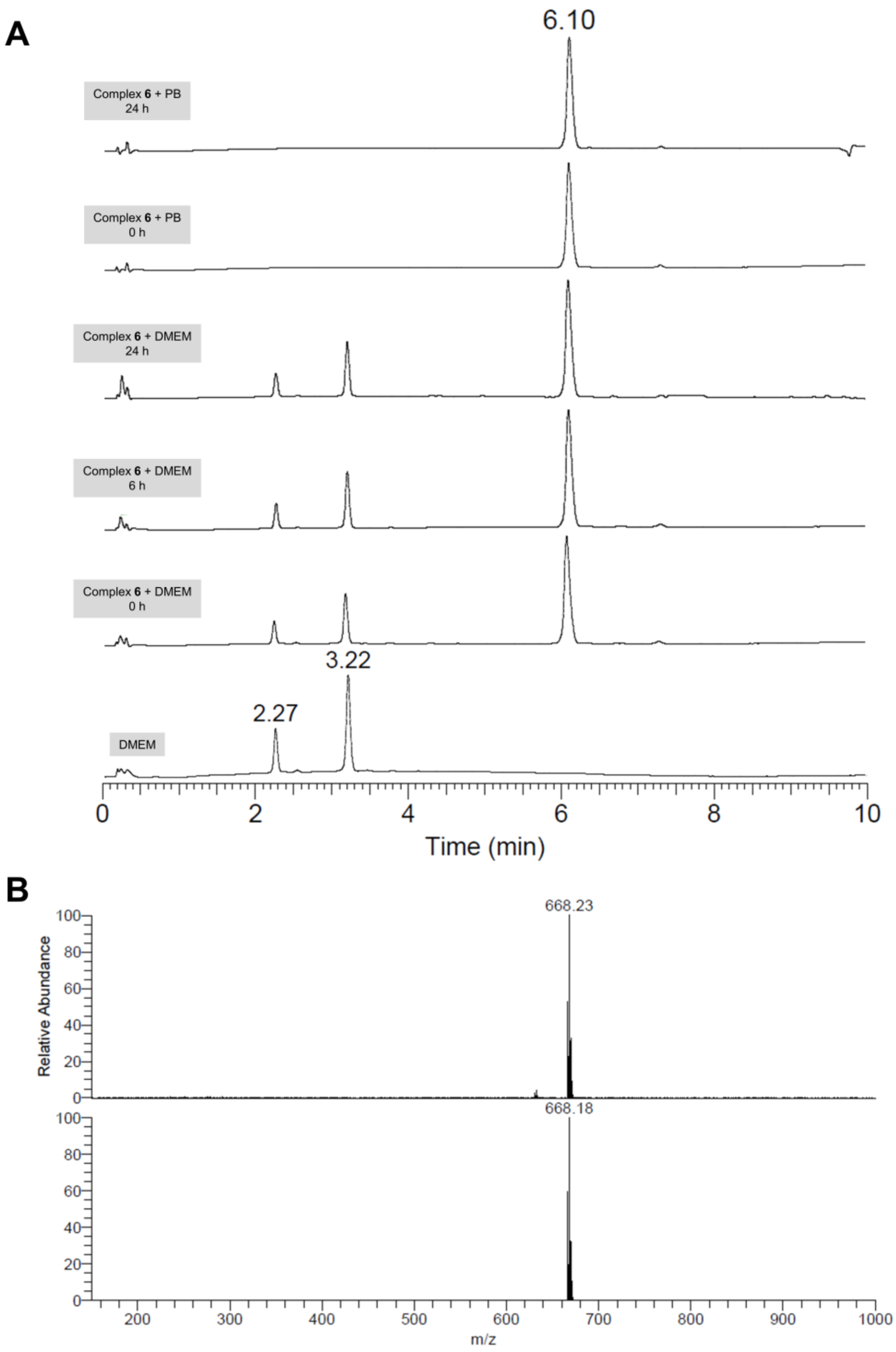

Figure S23. (A) HPLC traces for the complex **6** (50 μM final concentration) incubated for 24 h in 10% DMF/90% DMEM (without serum) or in 10% DMF/90% PBS in H_2O. (B) Experimental ESI+ mass spectrum for the HPLC peak centered at t_R = 6.10 min (top) and theoretical mass spectrum calculated for the $[IrCl(Cp^{ph})(L3)]^+$ species (bottom).

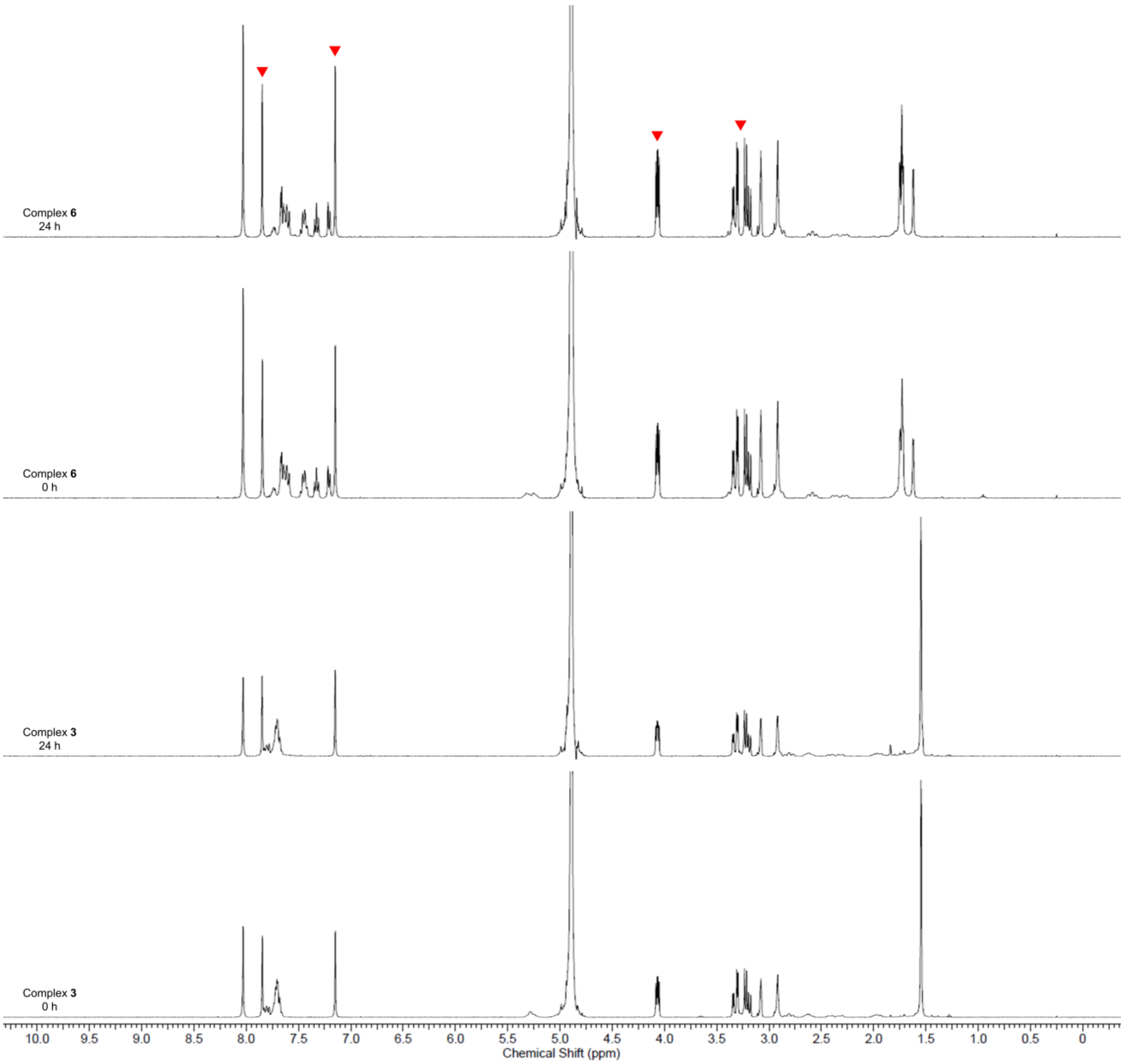

Figure S24. ^{1}H NMR stability studies of **3** and **6** with 5 molar equivalents of L-histidine in 10% DMF-d_7/90% PBS in D_2O (pH 7.4). Red triangles - His resonances.

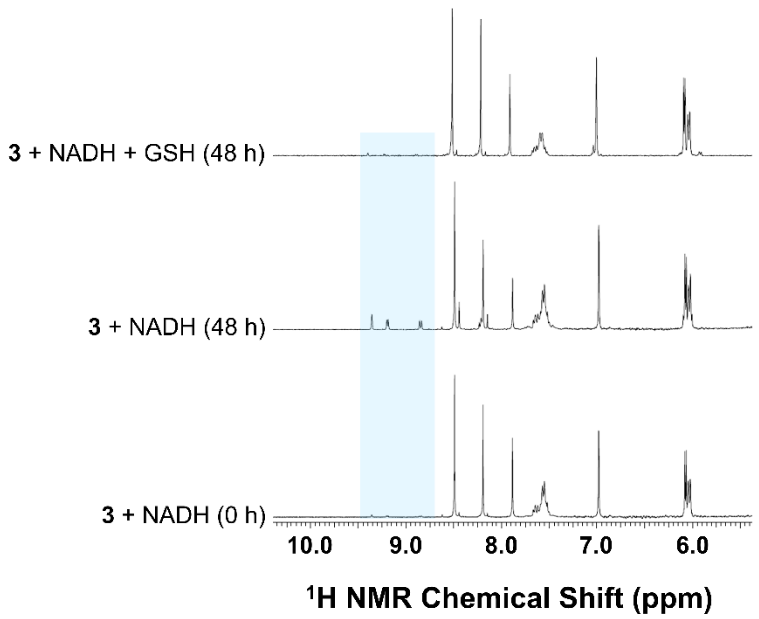

Figure S25. ^{1}H NMR stability studies of **3** with 5 molar equivalents of NADH (bottom, middle) or NADH and GSH (top) in 10% DMF-d_7/90% PBS in D_2O (pH 7.4). The region of characteristic NAD^+ resonances is colored in blue.

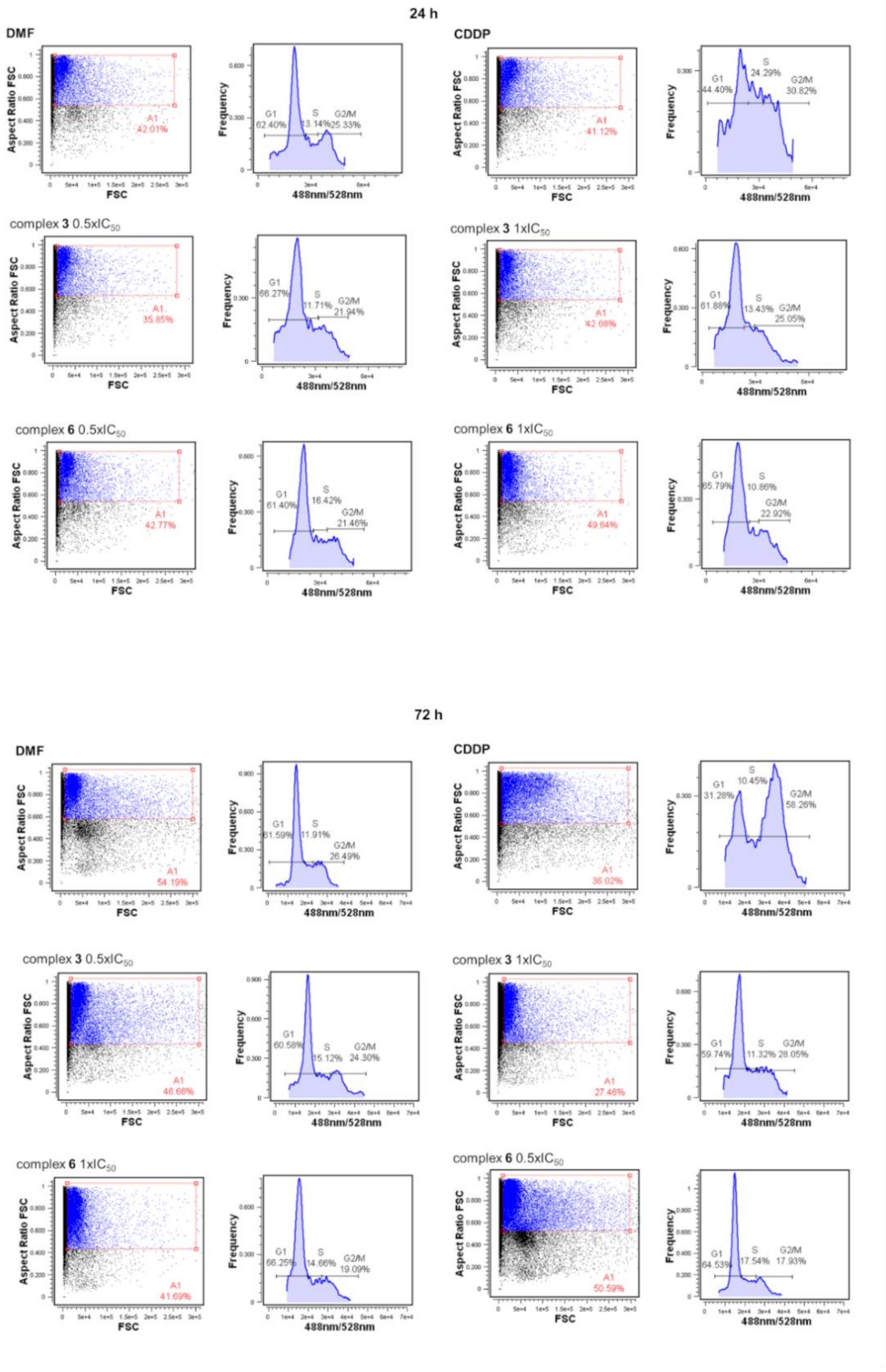

Figure S26. The effect of **3** and **6** on the cell cycle in MOR/CPR cells. The cells were incubated with **3** and **6** at the IC_{50} and half-IC_{50}, (i.e., 1.5 µM and 3.0 µM for **3**; 3.0 µM and 6.0 µM for **6**). Cisplatin (CDDP) at a concentration of 10 µM served as the positive control. The analysis was performed after 24 h and 72 h. Shown graphs represent one of three independent repetitions.

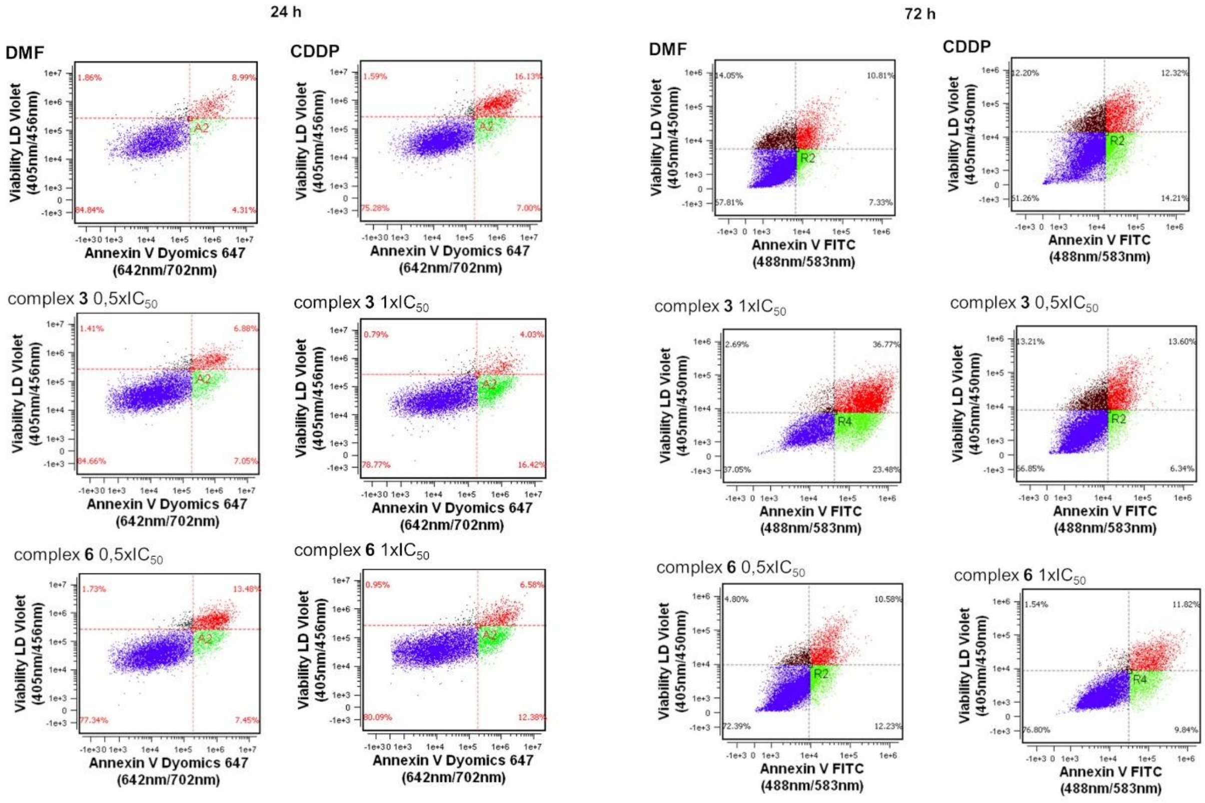

Figure S27. The effect of **3** and **6** on cell death in MOR/CPR cells. The cells were incubated with **3** and **6** at the IC_{50} and half-IC_{50}, (i.e., 1.5 µM and 3.0 µM for **3**; 3.0 µM and 6.0 µM for **6**). Cisplatin (CDDP) at a concentration of 10 µM served as the positive control. The analysis was performed after 24 h and 72 h. Analysis of cell death and apoptosis was performed using double staining with Annexin V Dyomics 642 and Live/Dead Violet. Shown dot-blot graphs represent one of three independent repetitions. Next, the dot plots were divided into four quadrants, such as early apoptosis - low right quadrant (LR; Annexin V positive, Live/Dead Violet negative); late apoptosis - up right quadrant (UR; Annexin V positive, Live/Dead Violet positive); and necrotic/dead cells - up left quadrant (UL; Annexin V negative, Live/Dead Violet positive).

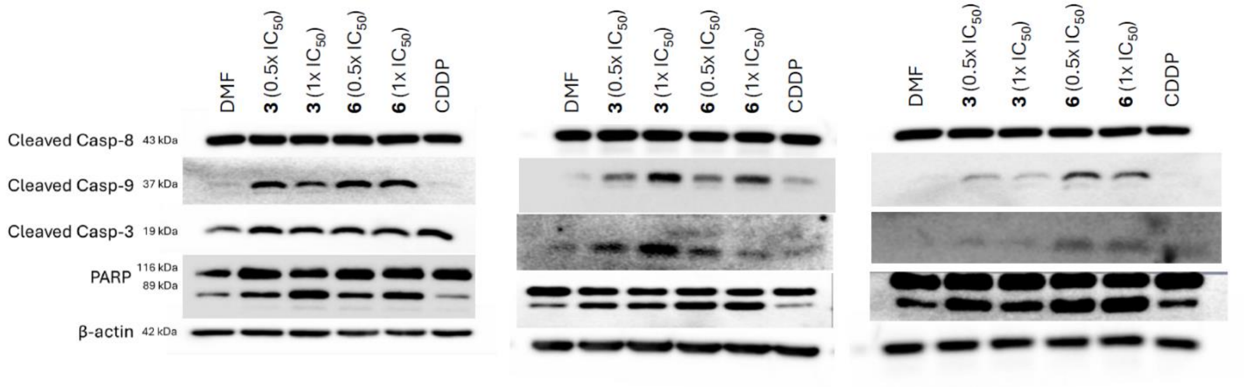

Figure S28. The effect of **3** and **6** on the level of apoptosis-related proteins in MOR/CPR cells. The cells were incubated with **3** and **6** at the IC_{50} and half-IC_{50}, (i.e., 1.5 µM and 3.0 µM for **3**; 3.0 µM and 6.0 µM for **6**) for 24 h. Cisplatin (CDDP) at a concentration 10 µM served as the positive control. Shown membranes represent three independent replicates. Casp - caspase; PARP - poly (ADP-ribose) polymerase.

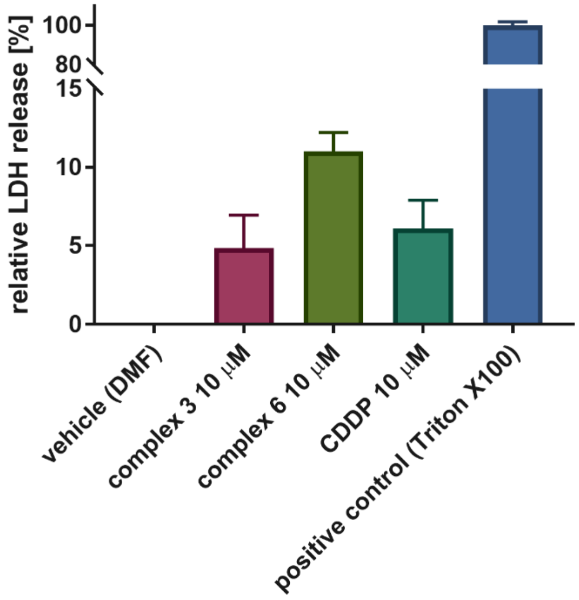

Figure S29. Relative release of lactate dehydrogenase (LDH) after 24 h incubation of A549 cells with complexes **3** and **6** and cisplatin (CDDP). Data are presented as mean ± SE.

Proteome analysis - general considerations. Proteomic analysis at the PCA level (Figure 8) clearly confirmed that **3** and **6** act through a MoA distinct from that of CDDP. Consistent with previous reports, Ir–Cp^x compounds primarily induce mitochondrial dysfunction and ROS generation.[16] In contrast, CDDP exerts its effect by crosslinking purine bases in DNA, thereby interfering with repair mechanisms and causing DNA damage.[17] **3** and **6** are similar, but not identical. A detailed proteomic analysis was therefore performed for **3**, which - consistent with the stress-related gene expression results (Figure 6) - showed a more pronounced impact on the proteome (Figure S30,S31).

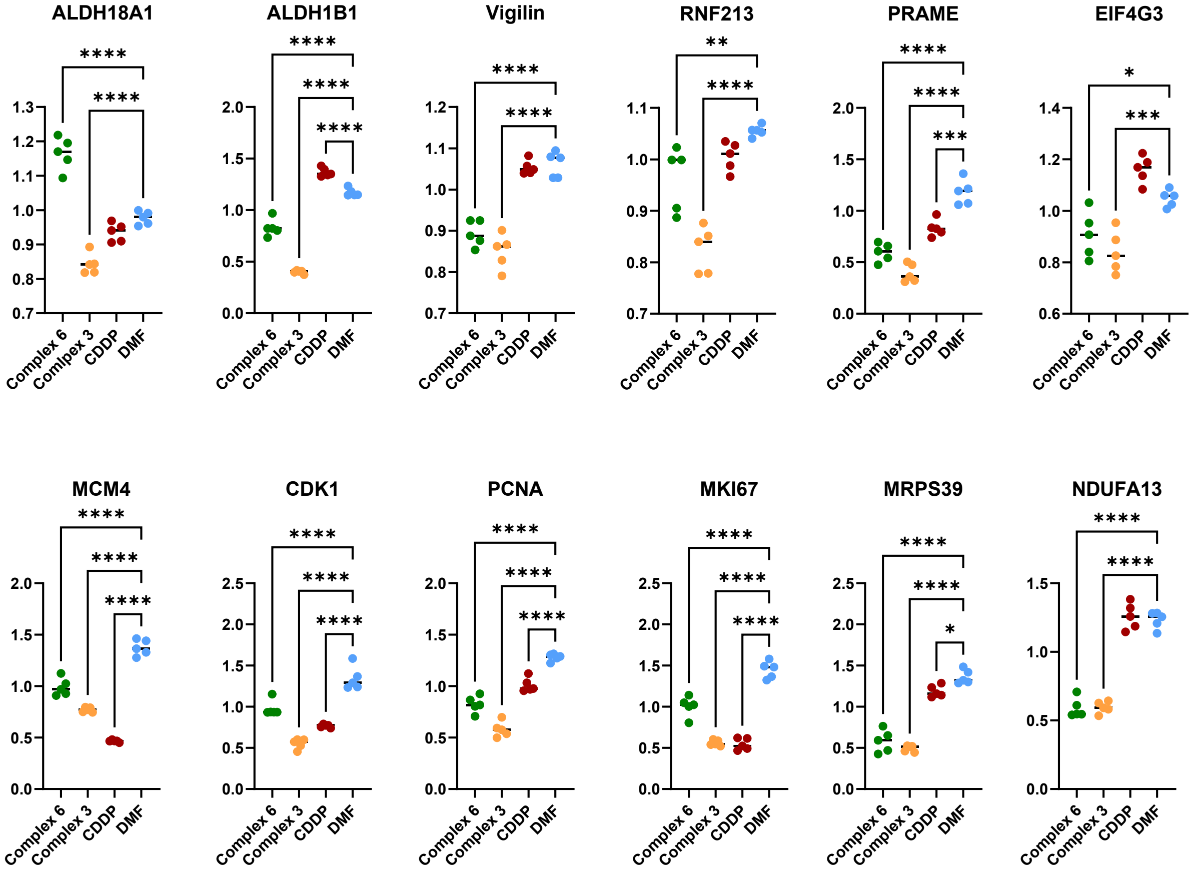

Figure S30. Comparison of the effects of 3 and 6 on the proteome of A549 cells. The corresponding Uniprot name and accession number are summarized in Table S4. Significance codes (multiple-testing corrected): ****, ***, **, and * denote adjusted P values < 0.0001, < 0.001, < 0.01, and < 0.05, respectively. Ordinary one-way ANOVA, Bonferroni´s multiple comparisons test.

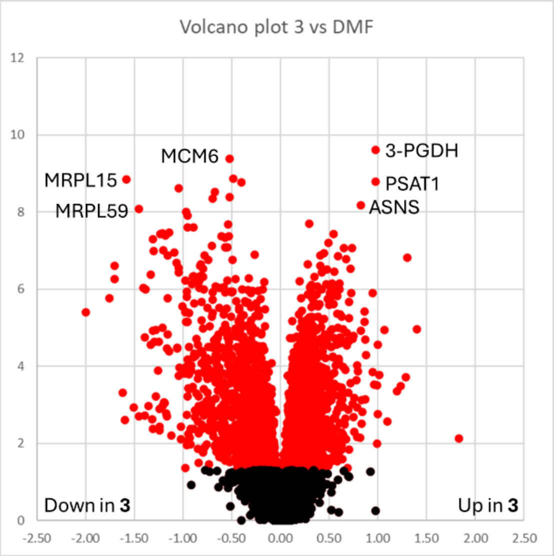

Figure S31. Volcano plot of proteomic differences between 3 and DMF samples. The x-axis shows the difference in medians of normalized protein abundances (**3** − DMF), and the y-axis shows $-\log_{10}$(p-value). Proteins highlighted in red are statistically significant ($\alpha < 0.05$); positive x-values indicate higher abundance in **3**, negative values indicate lower abundance relative to DMF. Significant proteins are color-highlighted.

Proteome analysis - primary target. The results of proteomic analysis show that **3** primarily targets **sites of ribosome biogenesis**. Gene ontology (GO) analysis of proteins significantly increased in A549 cells treated with **3** indicates enhanced activity of pathways related to rRNA processing and ribosome assembly (Figure S32A–D), including “rRNA processing,” “rRNA metabolic processes,” “ribosome biogenesis,” “ncRNA processing,” “snoRNA binding,” and “preribosome.”

However, despite this apparent upregulation of rRNA-related pathways, further proteomic analysis revealed a **decrease in the abundance of both small and large subunits of mitochondrial and cytoplasmic ribosomal proteins** (Figure S33). According to GO annotation, the number of mitochondrial ribosomes is particularly low (Figure S32E–H), which likely contributes to reduced synthesis of mitochondrial proteins.

Taken together, these findings suggest that **3** interferes with **ribosome biogenesis and stability**, leading to a global reduction in mature ribosomal components. This impairment may result from mTOR (mammalian target of rapamycin) inhibition,[18] p53-dependent stress response, or disruption of nucleolar function.[19] An alternative explanation is that the compound induces ribosomal stress, triggering degradation of rRNA and ribosomal proteins.[20] Collectively, these mechanisms converge on a **translational shutdown, with mitochondrial protein synthesis being particularly affected** (Figure S32E) - a hallmark of ribosomal destabilization.

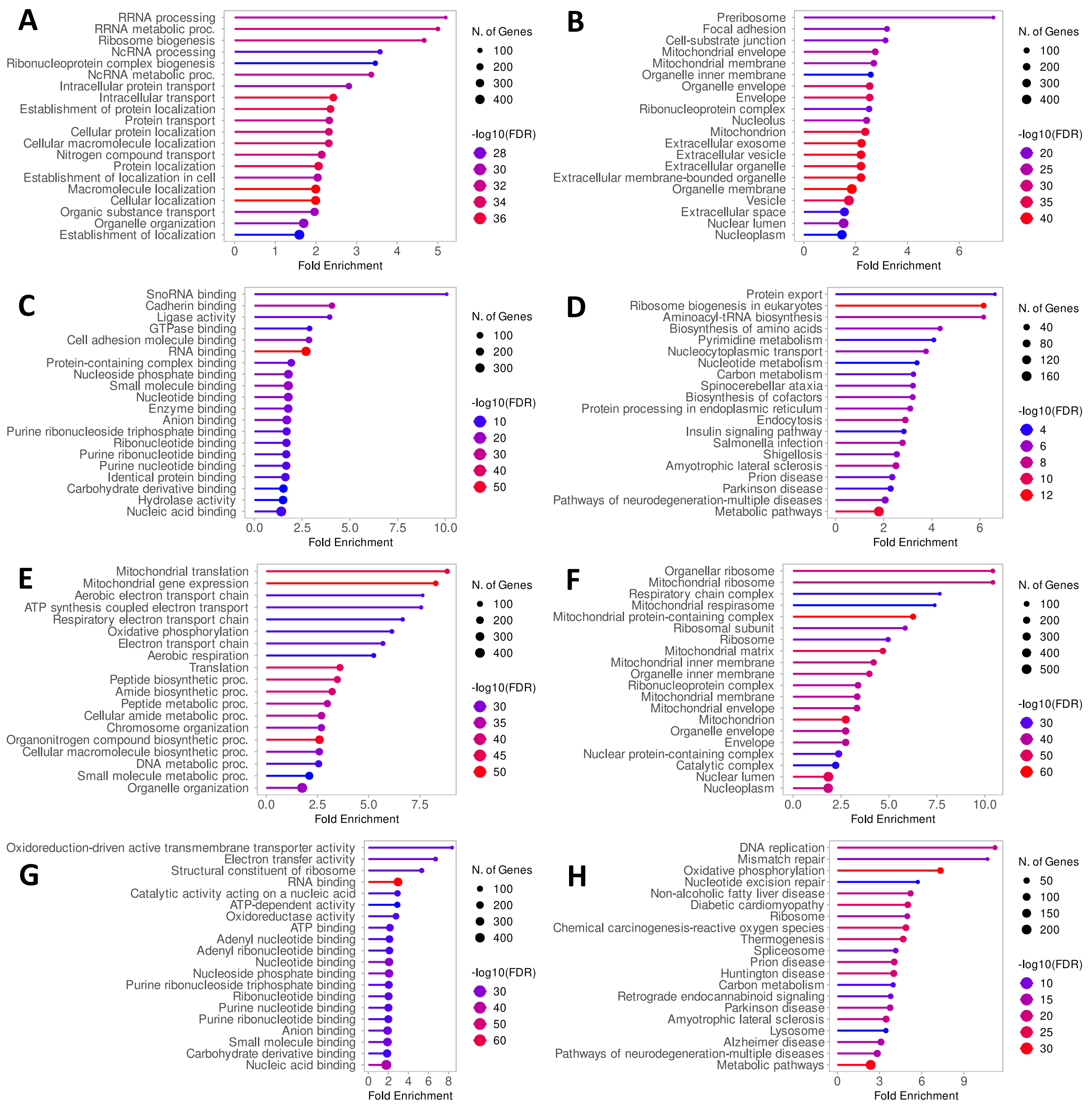

Figure S32. Gene Ontology (GO) enrichment analysis of proteins significantly altered in A549 cells treated with 3 versus DMF. Increased proteins: (A) Biological Process, (B) Cellular Component, (C) Molecular Function, (D) Metabolic processes. Decreased proteins: (E) Biological Process, (F) Cellular Component, (G) Molecular Function, (H) Metabolic processes. (ShinyGO 0.82).

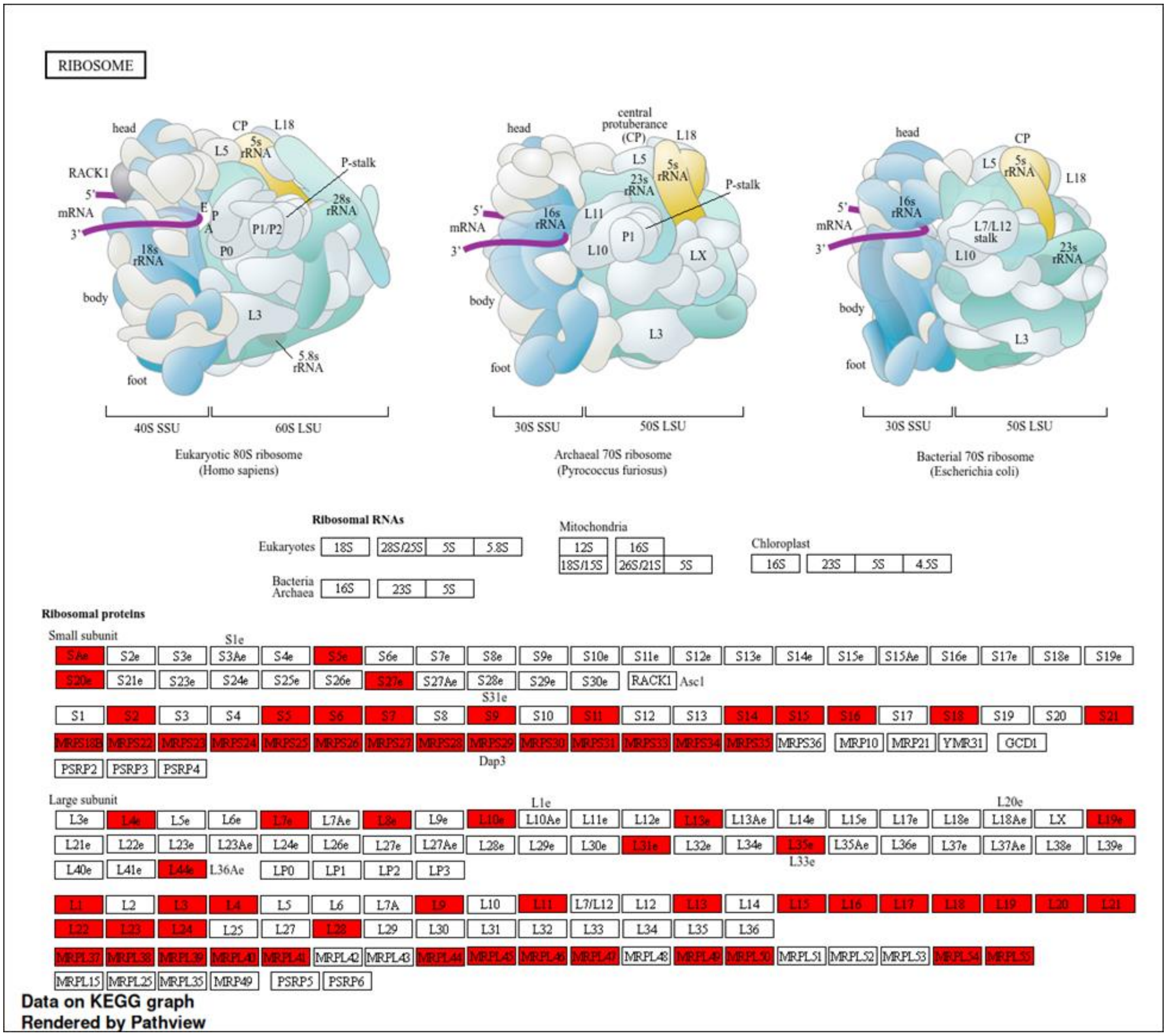

Figure S33. Effect of **3** on ribosomes (KEGG pathway hsa03010 Ribosome). Significantly reduced proteins are highlighted in red (ShinyGO 0.85).

Proteome analysis - nucleolus. A portion of **3** can **diffuse through nuclear pores** into the nucleus, where it preferentially accumulates in the nucleolus. GO enrichment for preribosomal complexes and rRNA/ncRNA processing pathways (Figure S32) supports this **subnuclear localization**. The nucleolus, enriched in rRNA and RNA-binding proteins, provides a polyanionic environment that electrostatically attracts the cationic complex **3**. In addition, π–π stacking and cation–π interactions with nucleobases further stabilize its association.[21]

Generally said, Ir–Cp^x compounds (such as **3**) can form coordination bonds with nucleolar proteins[22–24] such as nucleolin and nucleophosmin, both highly abundant in this compartment. These interactions reinforce **nucleolar retention**. Additionally, the dense, phase-separated nature of the nucleolus favors partitioning of lipophilic cations and limits their diffusion, resulting in persistent **nucleolar accumulation**.[25]

As a consequence, nucleolar stress (**ribosome biogenesis stress**) develops, characterized by activation of auxiliary and chaperone factors that attempt to restore ribosomal assembly (Figure S32,S34–S36). The upregulated factors span multiple stages of ribosome assembly, including rRNA transcription (Treacle protein, TCOF1, $p = 5.17\times10^{-5}$), chemical modification (e.g., dyskerin pseudouridine synthase 1, DKC1, $p = 5.44\times10^{-6}$; nucleolar protein 56, NOP56, $p = 2.19\times10^{-5}$; H/ACA ribonucleoprotein complex subunit 1, GAR1, $p = 2.04\times10^{-6}$), pre-rRNA processing (e.g., WD repeat–containing protein 75, UTP17, $p = 2.46\times10^{-6}$; U3 small nucleolar RNA–associated protein 6 homolog, UTP6, $p = 6.38\times10^{-6}$), and large-subunit maturation and export (ribosomal RNA small subunit methyltransferase NEP1, EMG1, $p = 8.20\times10^{-4}$).

In addition, DNA-directed RNA polymerases I, II, and III subunit RPABC3 ($p = 8.96\times10^{-5}$) and eukaryotic translation initiation factor 2 subunit 3 (EIF2S3, $p = 1.15\times10^{-7}$) were upregulated, consistent with a **global activation of the transcriptional and translational machinery** required to support ribosome biogenesis and stress adaptation. Supporting this interpretation, several ribosomal proteins were elevated, including small ribosomal subunit protein uS12 (RPS23; $p = 2.79\times10^{-4}$), small ribosomal subunit protein uS3 (RPS3; $p = 6.29\times10^{-4}$), ribosomal protein uL30-like (RPL7L1; $p = 9.96\times10^{-6}$), and large ribosomal subunit protein uL16 (RPL10; $p = 4.17\times10^{-4}$), along with U3 small nucleolar ribonucleoprotein protein IMP3 ($p = 9.20\times10^{-4}$), which is essential for early pre-rRNA processing.[26] Notably, increased levels of AMP-activated protein kinase subunits gamma-1 (PRKAG1, $p = 2.18\times10^{-5}$) and alpha-1 (PRKAA1, $p = 9.17\times10^{-4}$) suggest **activation of AMPK**, a key metabolic stress sensor that antagonizes mTORC1 signaling.[27] This coordinated activation of the ribosome biogenesis machinery, coupled with AMPK induction, indicates a cellular response characterized not by proliferative drive but by enhanced biosynthetic readiness within growth-suppressive conditions. Such a response may represent either a pro-survival adaptation or a compensatory reaction to nucleolar stress induced by the iridium complex, consistent with its known ability to perturb redox balance and nucleic acid metabolism in cancer cells.

This biosynthetic activation was accompanied by a distinct **stress-response signature**, marked by **activation of p53 and its downstream effectors**.[28,29] Upregulated proteins included p53 and DNA damage–regulated protein 1 (PDRG1, $p = 1.34\times10^{-3}$),[30] hypoxia up-regulated protein 1 (HYOU1, $p = 2.02\times10^{-4}$), and HEAT repeat–containing protein 1 (HEAT1, $p = 7.71\times10^{-4}$). Notably, canonical p53 transcriptional targets associated with **mitochondrial apoptotic priming** were elevated, including the apoptosis regulator BAX ($p = 4.56\times10^{-4}$) and the Bcl-2–binding component 3 (PUMA/JFY-1, isoforms 1/2; $p = 2.73\times10^{-3}$).[31] In contrast, **no significant induction of downstream apoptotic effectors** such as phorbol-12-myristate-13-acetate–induced protein 1 (NOXA) or activated caspases was observed, supporting the interpretation that apoptosis had not yet been executed but that mitochondria were primed for apoptotic signaling. This primed state may **sensitize cells to subsequent apoptotic stimuli** and reflects a broader stress-adaptive program

initiated in response to nucleolar disruption.[32] Consistent with this, key components of ribonucleoprotein (RNP) complexes appeared destabilized or limiting, exemplified by the downregulation of heterogeneous nuclear ribonucleoprotein F (HNRNPF, $p = 5.24\times10^{-3}$), indicating that the cell not only activates stress signaling pathways but also loses post-transcriptional stability, rendering it more vulnerable to additional insults.[33]

In agreement with these findings, there was an upregulation of genes associated with **nucleocytoplasmic transport**, particularly components of the nuclear pore complex (NPC) and export machinery (Figure S35,36), e.g., nucleoporin SEH1 ($p = 4.60\times10^{-4}$), suggesting structural remodeling of the NPC and increased nucleocytoplasmic trafficking. Concurrent upregulation of exportin-7 (XPO7, $p = 3.19\times10^{-4}$) and 60S ribosomal export protein NMD3 ($p = 2.22\times10^{-3}$) indicates stimulation of 60S ribosomal subunit export, consistent with nucleolar activation and ribosomal stress. Additionally, increased expression of transcription–export (TREX) and exon-junction complex (EJC) components (e.g., THO complex subunit 6, THOC6, $p = 9.22\times10^{-3}$; protein mago nashi homolog 2, MAGOHB, $p = 8.20\times10^{-3}$) supports **enhanced mRNA processing and nuclear export**.

Collectively, these findings reveal a coordinated cellular response along the nucleolus–nuclear pore–cytoplasmic axis, reflecting both the cell's attempt to restore ribosome production and mRNA export and the onset of translational repression and proteostatic stress following accumulation of 3 to the treated cells.

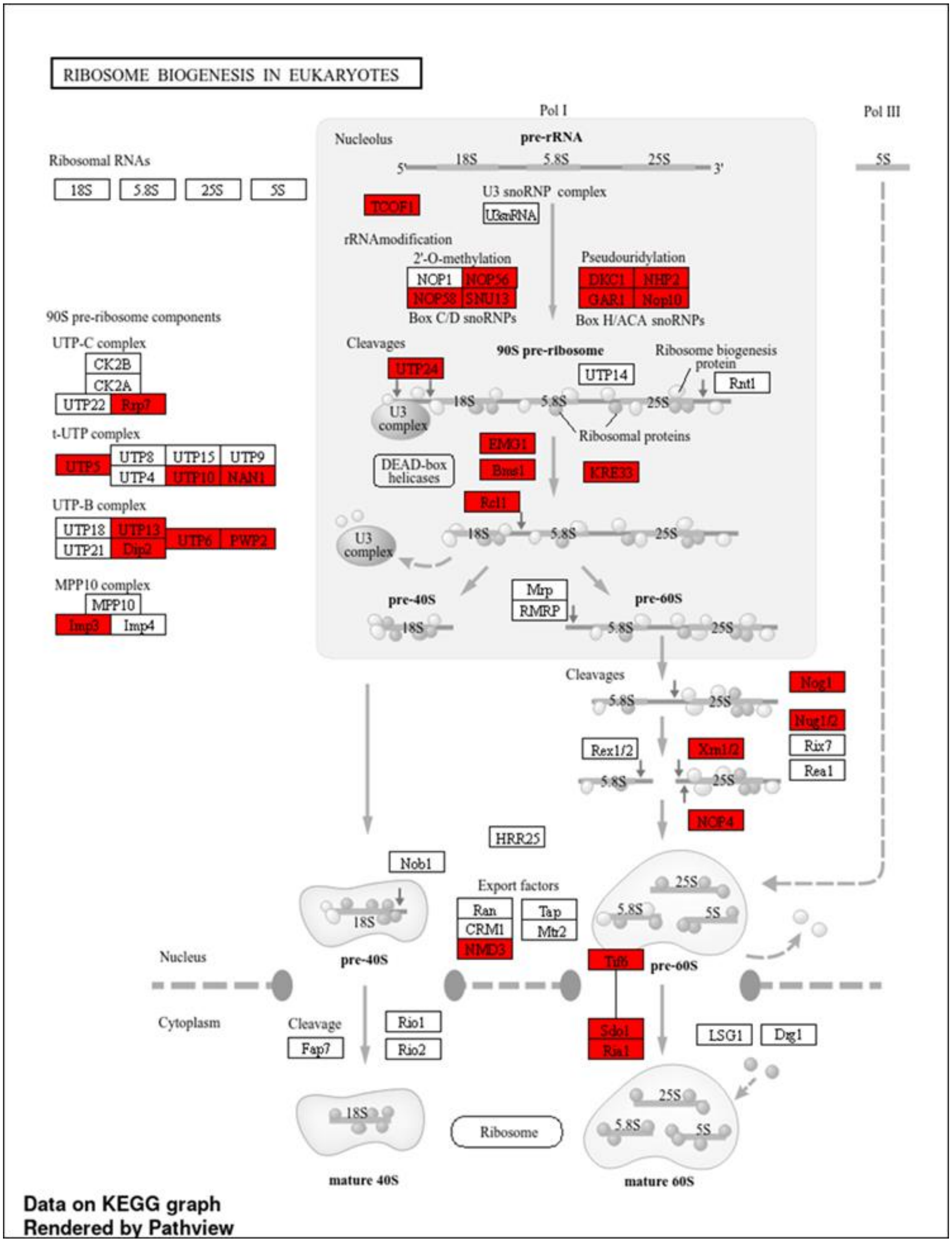

Figure S34. Proteome analysis - nucleolus. Effect of **3** on ribosome biogenesis in eukaryotes (KEGG pathway hsa03008 ribosome biogenesis in eukaryotes). Significantly upregulated proteins are highlighted in red (ShinyGO 0.85).

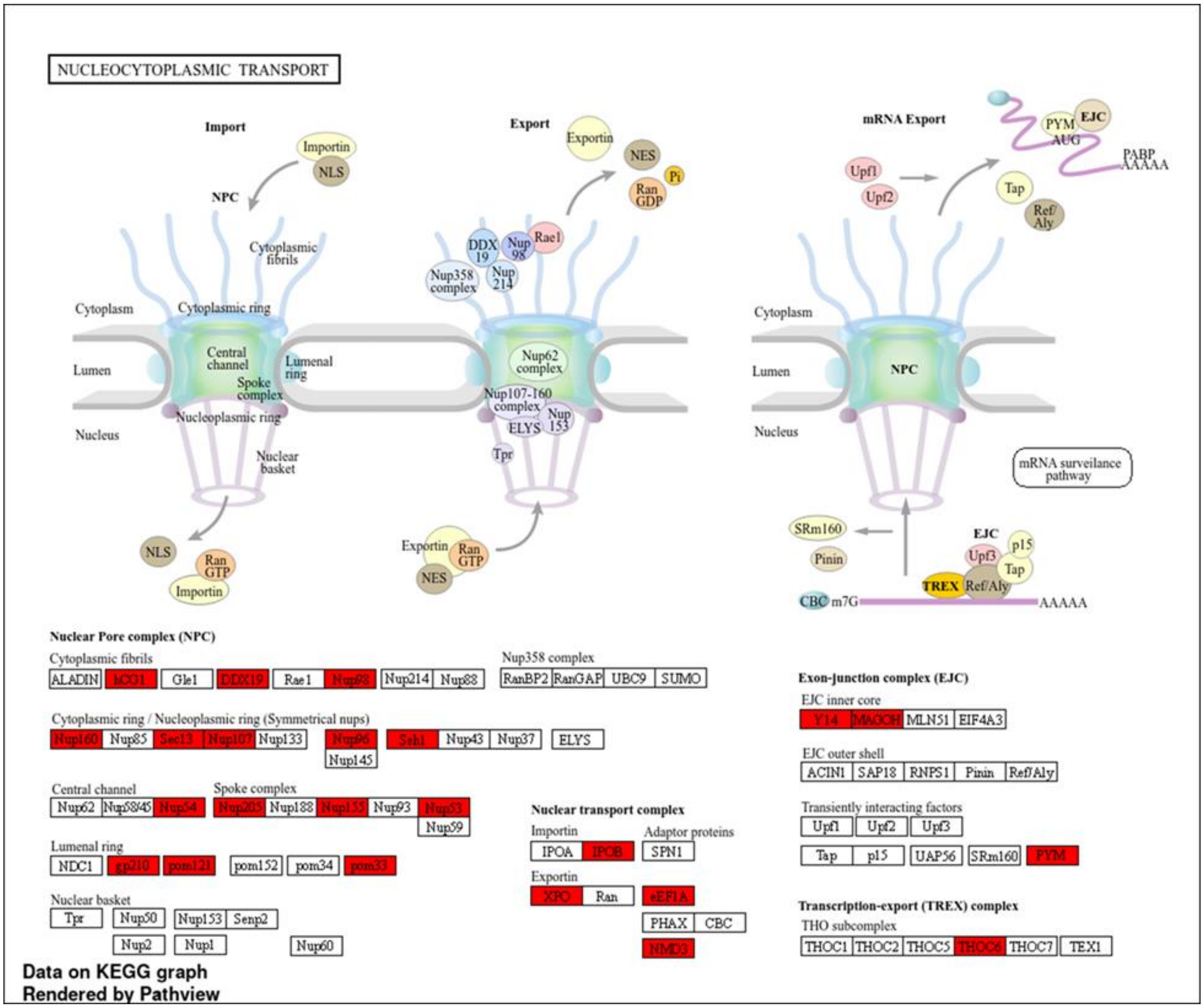

Figure S35. Effect of **3** on Nucleocytoplasmic transport (KEGG pathway hsa03013 Nucleocytoplasmic transport). Significantly upregulated proteins are highlighted in red (ShinyGO 0.85).

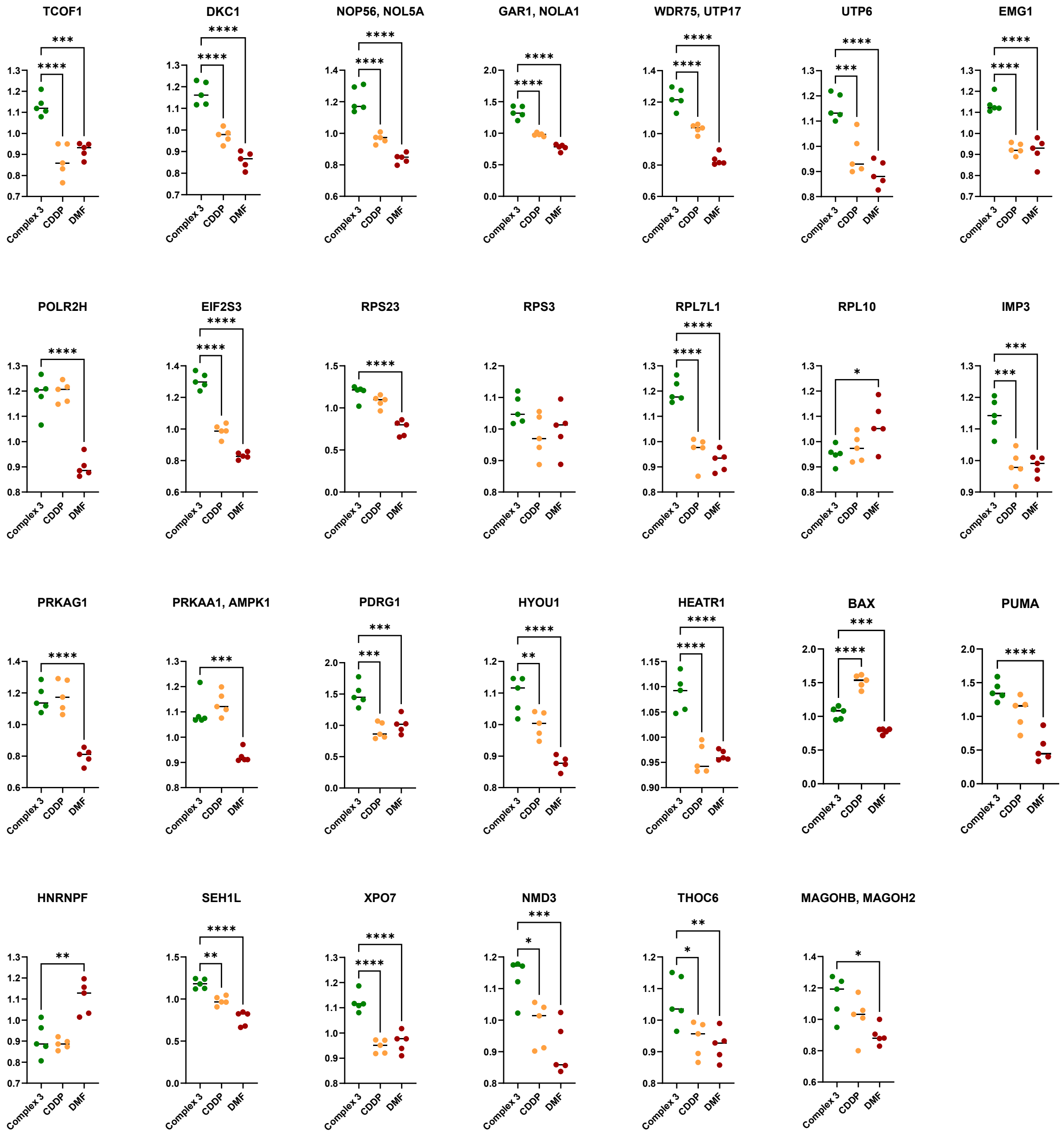

Figure S36. Box plots representing the effect of **3** on proteins included in **ribosome biogenesis** in nucleuolus of eukaryotes and nucleocytoplasmic transport. The box plots are labelled with gene names of proteins. The corresponding Uniprot name and accession number are summarized in Table S4. Significance codes (multiple-testing corrected): ****, ***, **, and * denote adjusted P values < 0.0001, < 0.001, < 0.01, and < 0.05, respectively. Ordinary one-way ANOVA, Bonferroni´s multiple comparisons test.

Proteome analysis - cellular entry. Compound **3**, as bulky lipophilic cation, **can cross membranes** without transporters and are driven into the cytoplasm by the mitochondrial membrane potential ($\Delta\psi_m \approx -150$ mV),[34] leading to their **accumulation in mitochondria**.[35] As an alternative pathway, **adsorptive endocytosis** may also mediate drug uptake (Figure S37,S38). Cationic compounds frequently bind to heparan sulfate proteoglycans on the cell surface and subsequently enter via clathrin- or caveolin-dependent endocytosis.[36] This mechanism is supported by the enrichment of GO cellular components related to focal adhesion and vesicular transport (see Figure S32), as well as a significant increase in the levels of the phosphatidylinositol-binding clathrin assembly protein (PICALM, $p = 3.24\times10^{-5}$) and several adaptor protein complex AP-2 subunits (e.g. AP2A2, $p = 7.41\times10^{-3}$) (Figure 9).

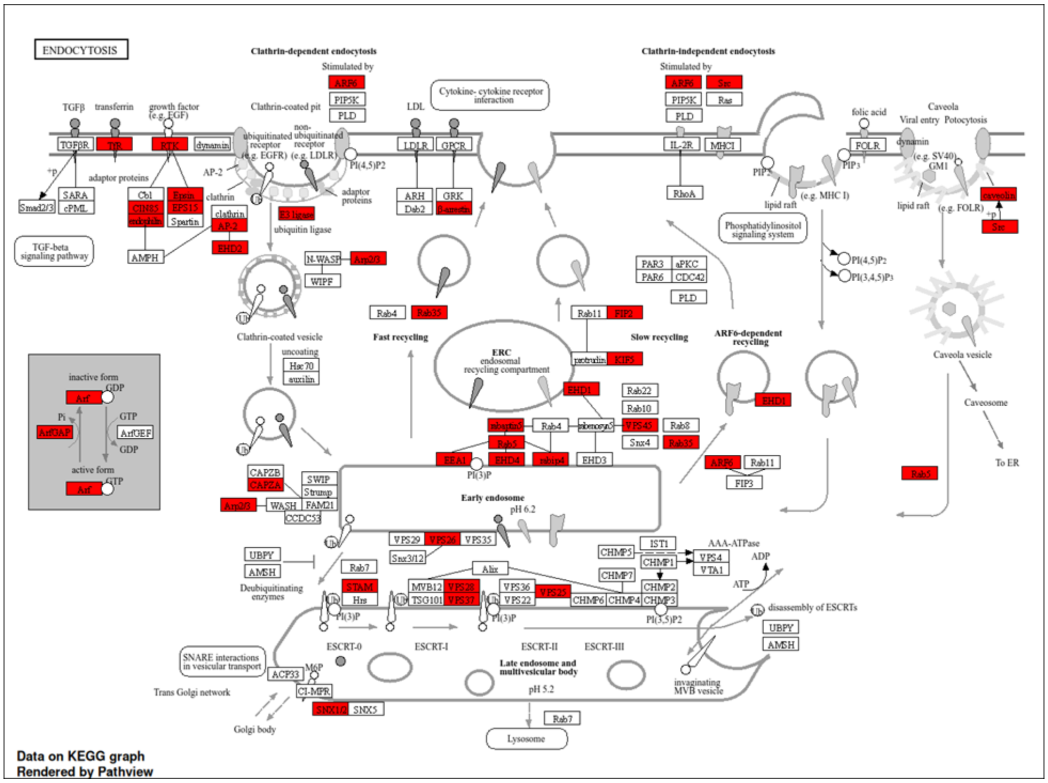

Figure S37. Effect of **3** on endocytosis (KEGG pathway hsa04144 Endocytosis). Significantly elevated proteins are highlighted in red (ShinyGO 0.85).

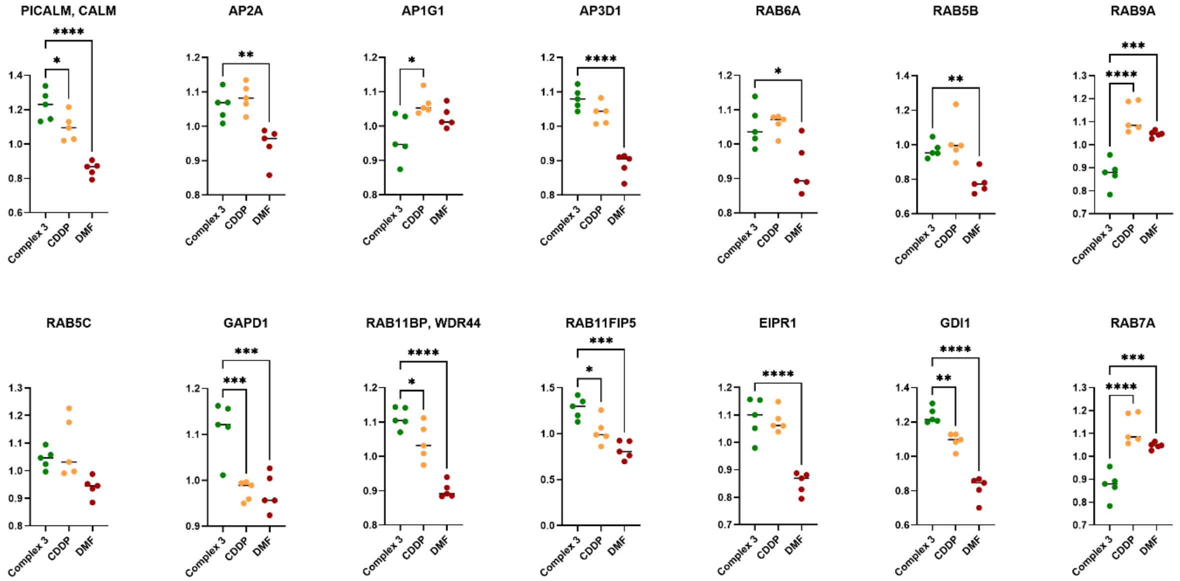

Figure S38. Box plots representing the effect of **3** on proteins included in **endocytosis pathway**. The box plots are labelled with gene names of proteins. The corresponding Uniprot name and accession number are summarized in Table S4. Significance codes (multiple-testing corrected): ****, ***, **, and * denote adjusted P values < 0.0001, < 0.001, < 0.01, and < 0.05, respectively. Ordinary one-way ANOVA, Bonferroni´s multiple comparisons test.

Proteome analysis - endolysosomal system. The compound **3** profoundly **remodels the endolysosomal system** (Figure 9), as evidenced by altered lysosomal morphology and trafficking dynamics (Figure S39,S40).[37] The overall shift favors enhanced endocytic input and early trafficking, while attenuating late maturation and degradation.

Increased levels of adaptor proteins AP-2 and PICALM[38] indicate **accelerated endocytic uptake**. Elevated AP-1 ($p = 6.70\times10^{-4}$) and AP-3 subunits (e.g., δ-1, $p = 2.96\times10^{-5}$) suggest **intensified cargo sorting between the trans-Golgi network (TGN), endosomes, and lysosomes**.[39–41] Small GTPases Ras-related protein Rab-5A/B/C (RAB5A/B/C, $p = 5.89 / 4.23 / 2.42 \times10^{-3}$, respectively) and Rab5-activating protein 6 ($p = 3.23\times10^{-3}$) are upregulated,[42] consistent with **enhanced early endosome fusion and maturation**. The **recycling axis is reinforced** by increased WD repeat-containing protein 44 (Rab11-binding protein, RAB11BP), Rab11 family–interacting protein 5 J RAB11FIP5 (RIP11), and EARP and GARP complex-interacting protein 1 (EIPR1, $p = 1.71\times10^{-6}$, and 2.46×10^{-4}, 3.45×10^{-4}, respectively),[43] which together form a functional RAB11–RIP11 module facilitating retrograde transport to the TGN.[44] Upregulation of Rab GDP dissociation inhibitor alpha (GDI1, $p = 1.24\times10^{-4}$) supports intensified Rab cycling and vesicle turnover.[45]

In contrast, Ras-related protein Rab-7A and Rab-9A (RAB7A, RAB9A, $p = 4.23\times10^{-3}$ and 3.28×10^{-2}, respectively) are downregulated, indicating **reduced conversion of early endosomes to late degradative compartments**.[46,47] Upregulation of AP-3, together with downregulation of RAB7A and RAB9A, suggests that Ir(III) **compounds are rerouted from early endosomes directly to lysosomes** via an AP-3–mediated pathway, bypassing the canonical late endosomal route.[48] This results in transport vesicles forming directly from the EE or TGN and fusing with lysosomes. This process bypasses the classical LE phase, making it **faster and less degradative**. It may also contribute to **vesicle retention under conditions of lysosomal dysfunction**.

Lysosomal acidification and enzymatic capacity also decline, with multiple subunits of the vacuolar-type ATPase (V-ATPase) decreased (S1 and E1, $p \approx 5\times10^{-3}$),[49] suggesting compromised acidification (Figure S39,S40). Lysosome-associated membrane glycoprotein 1 decreases (LAMP-1, $p = 2.21\times10^{-2}$), whereas Lysosome membrane protein 2 increases (LIMP-2, $p = 2.33\times10^{-3}$),[50] indicating **altered lysosomal membrane composition and a shift toward export-oriented function**. Key lipid-handling proteins, including NPC intracellular cholesterol transporter 2 (NPC2, $p = 5.99\times10^{-7}$) and prosaposin (PSAP, $p = 3.44\times10^{-2}$), are markedly reduced, impairing cholesterol efflux and promoting **lysosomal lipid accumulation -** conditions favoring **retention of lipophilic Ir(III) compounds**.[51] Concomitant decreases in degradative enzymes such as acid lipase (LAL, EC:3.1.1.13, $p = 8.45\times10^{-5}$), acid β-glucosidase (GCase/GBA, EC:3.2.1.45, $p = 1.47\times10^{-4}$), sialidase-1 (NEU1, EC:3.2.1.18, $p = 1.55\times10^{-4}$), deoxyribonuclease IIα (DNASE2A, EC:3.1.22.1, $p = 4.07\times10^{-5}$), and cathepsins L, C, S, and Z ($p = 1.61\times10^{-4}$, 2.30×10^{-3}, 9.32×10^{-3}, and 3.22×10^{-3}, respectively), along with phospholipase D1 (PLD1, EC:3.1.4.4, $p = 1.60\times10^{-3}$) indicate broad **suppression of hydrolytic capacity**.

Accumulation of lipophilic compounds triggers upregulation of LIMP-2 ($p = 2.33\times10^{-3}$), which mediates drug **export from lysosomes**.[50] However, **3** - due to its cationic, lipophilic nature - **remains largely retained** within the endolysosomal system.[51,52] This retention is reinforced by **perinuclear clustering of lysosomes** (↑RAB34 $p = 5.84\times10^{-4}$; ↑RAB35 $p = 3.58\times10^{-3}$)[53] and diminished degradative turnover. The resulting "**degradative brake**" induces **lysosomal stress**, leading to **dissociation of mTORC1 from the lysosomal membrane**.[54] Downregulation of V-ATPase impairs acidification, promoting calcium release and activation of calcineurin, which dephosphorylates and **activates transcription factor EB (TFEB)**. Upregulation of calcineurin subunits ($p = 1.82\times10^{-3}$ and 4.13×10^{-5}) confirms this pathway. TFEB-driven responses - such as increased Calcineurin subunit B type 1 (LC3B2, $p = 8.04\times10^{-3}$), Autophagy protein 5 (ATG5, $p = 9.24\times10^{-6}$), and t-SNAREs homolog 1A (VTI1A, p =

4.43×10^{-4}) - initiate **autophagy and partial lysosomal biogenesis** (↑LIMP-2, modest ↑Cathepsin D), though **lysosomal enzymes remain downregulated**. This incomplete TFEB activation likely reflects transcriptional and translational repression under stress.[55–57]

Despite TFEB activation, **3 accumulates in lysosomes and recycling endosomes**, consistent with impaired lysosomal clearance and altered vesicular trafficking. Given their cationic and lipophilic nature, these compounds **may partially escape from recycling endosomes into the cytosol, contributing to mitochondrial targeting**. However, proteomic data reveal upregulation of exocyst components (EXOC2, $p = 3.93\times10^{-3}$; EXOC4, $p = 7.43\times10^{-3}$; EXOC7, $p = 1.35\times10^{-2}$), SNARE proteins (Syntaxin 4, STX4, $p = 7.32\times10^{-4}$), and vesicle priming regulators (Protein unc-119 homolog B, UNC119B, $p = 3.59\times10^{-6}$; Protein unc-13 homolog B, UNC13B, $p = 8.90\times10^{-3}$),[58] alongside enrichment of the GO term "protein export" (Figure S32D), suggesting that **cells actively attempt to expel the retained Ir compound 3 via exocytosis**. Upregulation of SH3 domain-containing kinase-binding protein 1 (SH3KBP1, $p = 2.48\times10^{-7}$), Proto-oncogene tyrosine-protein kinase Src (SRC, EC:2.7.10.2, $p = 6.93\times10^{-5}$), and SHC-transforming protein 1 (SHC1, $p = 7.05\times10^{-5}$) further supports **active vesicular remodeling and stress signaling**.[59] SH3KBP1 and SRC are known regulators of endocytosis and exocytosis, while SHC1 links receptor activation to metabolic adaptation.[60] Their elevation suggests that **3**-induced vesicular retention triggers compensatory trafficking and signaling responses, reinforcing the observed exocytotic activity and downstream nucleolar stress.

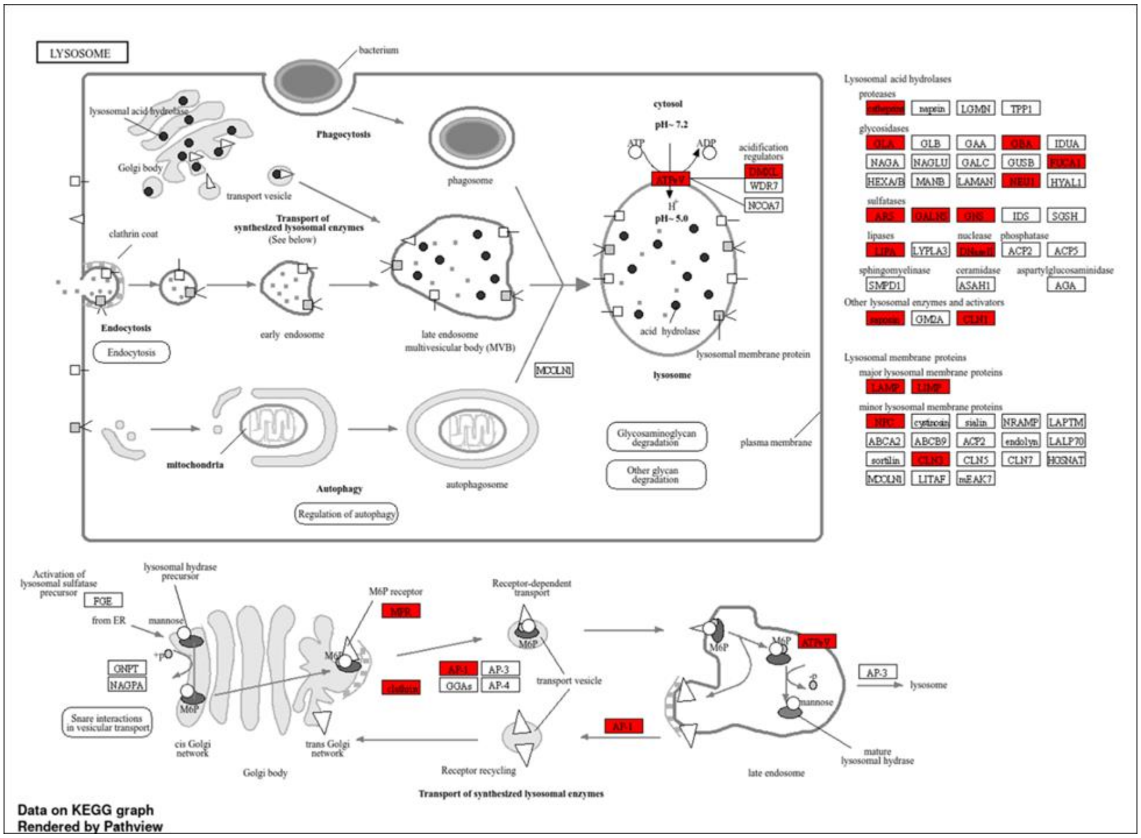

Figure S39. Effect of **3** on lysosomes (KEGG pathway hsa04142 Lysosome). Significantly downregulated proteins are highlighted in red (ShinyGO 0.85).

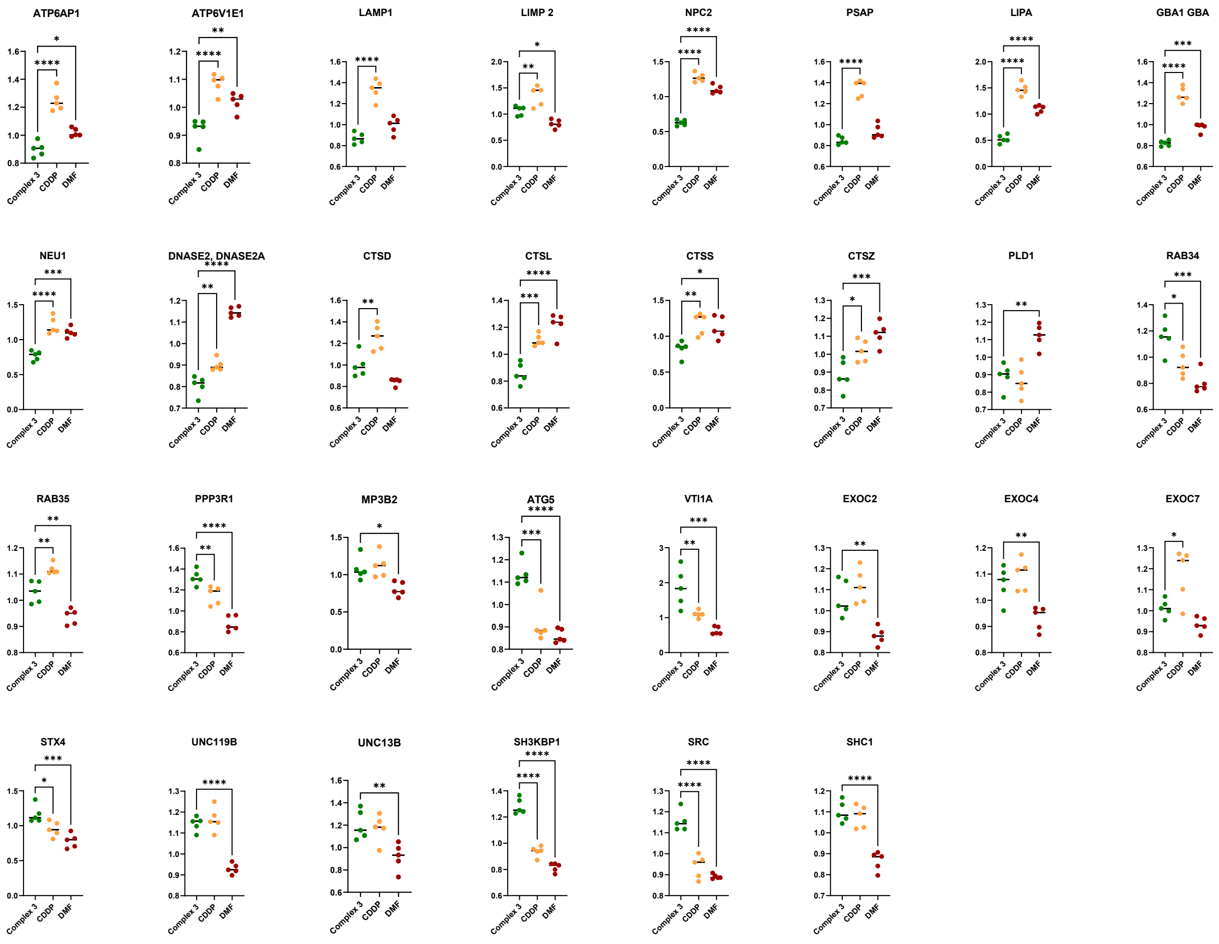

Figure S40. Box plots representing the effect of **3** on proteins included in **lysosomes**. The box plots are labelled with gene names of proteins. The corresponding Uniprot name and accession number are summarized in Table S4. Significance codes (multiple-testing corrected): ****, ***, **, and * denote adjusted P values < 0.0001, < 0.001, < 0.01, and < 0.05, respectively. Ordinary one-way ANOVA, Bonferroni´s multiple comparisons test.

Proteome analysis - mitochondria. Proteomic and functional analyses indicate that mitochondria represent the principal intracellular organelle affected by **3** that induces a **pronounced suppression of mitochondrial translation** (Figure S32E). This suppression is multifaceted and involves not only structural components but also regulatory factors. Alongside a broad reduction of mitoribosomal proteins (e.g., large ribosomal subunit protein uL15m, MRPL15, $p = 1.43\times10^{-9}$; small ribosomal subunit protein mS39, MRPS39, $p = 1.01\times10^{-8}$; Figure S33), the expression of translation-regulating proteins such as SRA stem-loop-interacting RNA-binding protein (SLIRP, $p = 2.07\times10^{-8}$), leucine-rich PPR motif-containing protein (LRPPRC, $p = 1.67\times10^{-9}$), and nitric oxide-associated protein 1 (NOA1, $p = 2.73\times10^{-5}$) - which stabilize mitochondrial transcripts and coordinate their translation - was also significantly reduced. This concurrence suggests that the inhibition is both structural and post-transcriptionally controlled.

Mitochondrial genome biosynthesis and stability are likewise compromised. Reduced levels of mitochondrial DNA polymerase (POLG, $p = 3.65\times10^{-5}$) and rRNA methyltransferase 3 (MRM3, $p = 1.23\times10^{-6}$) indicate disruption of mtDNA replication and maintenance, further impairing mitochondrial translation and respiratory subunit synthesis. This inhibition likely results from direct interaction of **3** with mitochondrial ribosomes - possibly through rRNA or ribosomal proteins - leading to reduced synthesis of mtDNA-encoded subunits of the respiratory chain. Consequently, **subunits across all respiratory complexes** (e.g., NADH dehydrogenase [ubiquinone] 1 alpha subcomplex subunit 13 of complex I, NDUFA13, $p = 7.40\times10^{-8}$; cytochrome c oxidase subunit 7C of complex IV, COX7C, $p = 9.06\times10^{-7}$) were decreased in abundance, resulting in impaired electron transport and attenuated ATP synthesis (Figure S41,42). The most pronounced effects were observed for complexes I and IV, which depend on mtDNA-encoded subunits and on the integrity of cardiolipin - an inner-membrane phospholipid essential for respiratory supercomplex assembly and stability.[61] Both complexes rely on proper I–III–IV supercomplex organization for efficient electron transfer. If **3** preferentially associate with cardiolipin or modify matrix-facing protein motifs, such interactions could destabilize lipid–protein contacts essential for supercomplex maintenance, thereby causing partial functional loss of complexes I and IV. It is also important to note that the **resulting ATP deficiency** - consistent with the impaired respiratory chain function described above - explains the observed **reduction in vacuolar ATPase activity and lysosomal acidification**, both processes that rely on cytosolic ATP for proton pumping.

Beyond translational arrest and respiratory chain destabilization, **3** elicits extensive **remodeling of mitochondrial architecture and proteostasis**. Proteomic data reveal upregulation of key mitochondrial chaperones and proteases, including ATP-dependent ClpX-like chaperone (CLPX, $p = 3.06\times10^{-7}$), Lon protease homolog (LONP1, $p = 1.58\times10^{-6}$), DNAJA3, and mitochondrial disaggregase (CLPB, $p = 5.37\times10^{-6}$), consistent with activation of the mitochondrial unfolded protein response (UPR^{mt}). This stress-adaptive pathway is typically triggered by accumulation of misfolded proteins in the matrix and functions to restore proteome integrity through enhanced folding, degradation, and import mechanisms.[62]

Although direct evidence for accumulation of **3** in mitochondria is currently lacking, several proteomic signatures are consistent with preferential mitochondrial localization and **metal-induced matrix stress**. The strong upregulation of the mitochondrial glutathione transporter SLC25A40 ($p = 2.62\times10^{-5}$), which facilitates GSH import, and microsomal glutathione S-transferase 1 (MGST1, $p = 2.79\times10^{-5}$), localized to the outer mitochondrial membrane, both point to activation of thiol-based detoxification pathways. In parallel, increased expression of glutathione synthetase (GSS, EC:6.3.2.3, $p = 1.15\times10^{-3}$), stress-70 protein (HSPA9, $p = 5.54\times10^{-5}$), and 26S proteasome regulatory subunit 12 (PSMD12, $p = 6.82\times10^{-6}$) reflects enhanced antioxidant and proteostatic defenses. Collectively, this ensemble of responses - elevated thiol metabolism, chaperone activation, and proteasomal quality control - constitutes a canonical signature of metal-induced stress typically mitigated via glutathione and cysteine coordination.[63,64]

In contrast to the induction of thiol-based defenses, mitochondrial aldehyde dehydrogenase (ALDH1B1, EC:1.2.1.3, $p = 2.42\times10^{-9}$) and carbamoyl-phosphate synthase (CPS1, EC:6.3.4.16, $p = 9.32\times10^{-6}$) were significantly downregulated. Given that ALDH1B1 detoxifies lipid peroxidation-derived aldehydes and supports matrix metabolism, and CPS1 catalyzes carbamoyl phosphate formation from ammonia and bicarbonate to enable urea biosynthesis, their suppression likely exacerbates **redox stress** induced by **3** and contributes to the partial loss of complexes I and IV function.

To compensate for reduced oxidative phosphorylation, A549 cells reprogram energy metabolism toward **glycolysis**.[65] Upregulation of hexokinase domain-containing protein 1 (HKDC1, EC 2.7.1.1, $p = 2.63\times10^{-5}$) and ATP-dependent 6-phosphofructokinase, platelet type (PFKP, EC:2.7.1.11, $p = 4.61\times10^{-4}$) supports this metabolic shift. Glycolytically derived ATP suffices to sustain basal cellular functions, while mitochondrial membrane potential ($\Delta\psi_m$) remains preserved through reverse operation of ATP synthase (complex V), which hydrolyzes cytosolic ATP to maintain proton translocation across the inner membrane. This mechanism stabilizes $\Delta\psi_m$ and prevents outer-membrane permeabilization, thereby averting apoptotic initiation.[66]

Concurrently, **increased expression of mitochondrial import components** such as mitochondrial processing peptidase subunit alpha (PMPCA, $p = 1.88\times10^{-5}$), acylglycerol kinase (AGK, EC:2.7.1.107, $p = 4.57\times10^{-6}$), and sorting and assembly machinery component 50 homolog (SAMM50, $p = 2.04\times10^{-5}$) indicates compensatory enhancement of protein trafficking into the organelle. These adaptations likely facilitate selective replacement of damaged proteins and promote organelle recovery. Upregulation of optic atrophy protein 1 (OPA1, $p = 2.58\times10^{-3}$) - a dynamin-like GTPase involved in inner-membrane fusion and cristae remodeling - further supports active **mitochondrial network reorganization**. Fusion with intact mitochondria may serve to dilute damage and preserve bioenergetic function, as previously described in stress-adapted cancer cells.[67]

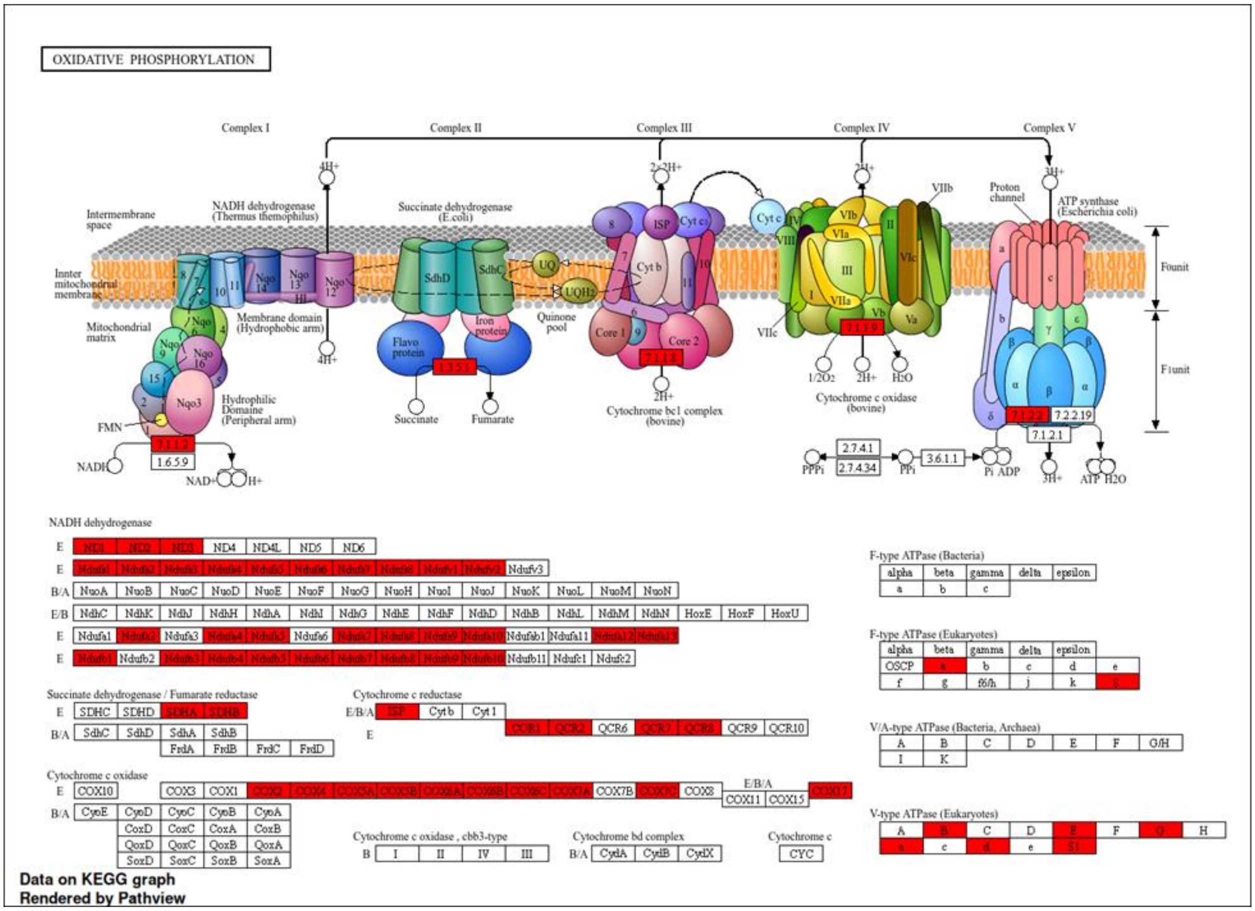

Figure S41. Effect of **3** on respiratory chain (KEGG pathway hsa00190 Oxidative phoshorylation). Significantly reduced proteins are highlighted in red (ShinyGO 0.85).

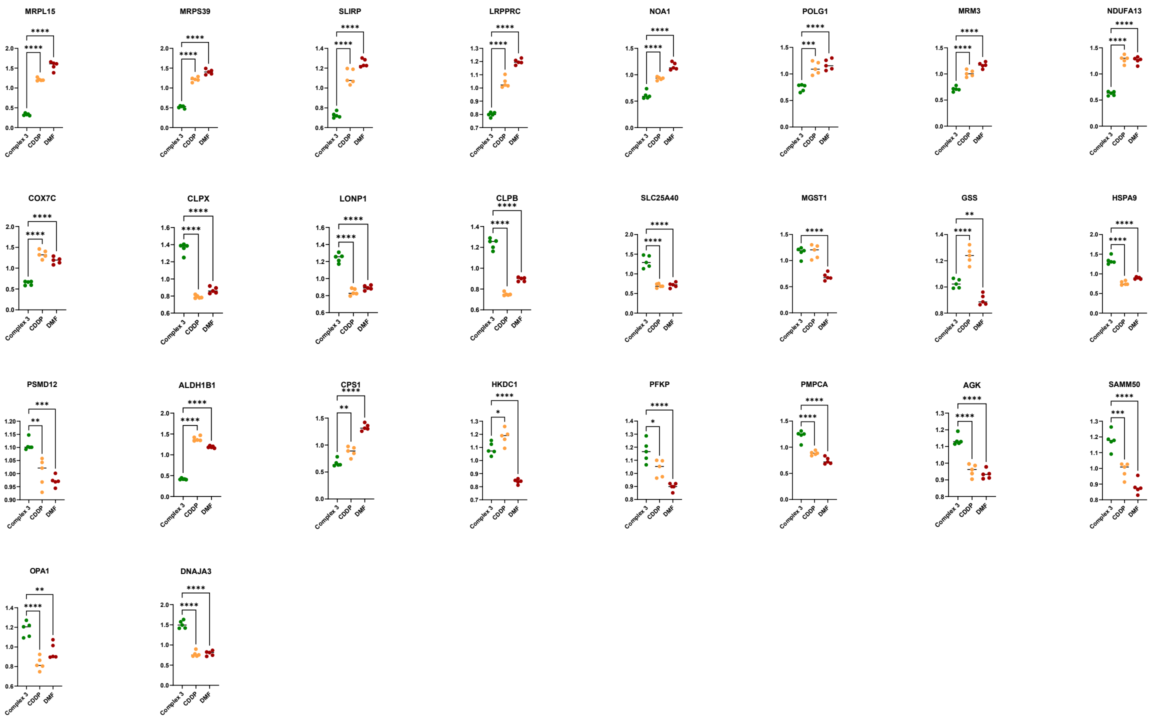

Figure S42. Box plots representing the effect of **3** on proteins included in **respiratory chain and mitochondria**. The box plots are labelled with gene names of proteins. The corresponding Uniprot name and accession number are summarized in Table S4. Significance codes (multiple-testing corrected): ****, ***, **, and * denote adjusted P values < 0.0001, < 0.001, < 0.01, and < 0.05, respectively. Ordinary one-way ANOVA, Bonferroni´s multiple comparisons test.

Proteome analysis - endoplasmic reticulum. Proteomic analysis revealed a consistent upregulation of genes associated with **protein folding, quality control, and UPR** in A549 cells treated with **3** (Figure S43,S44). Notably, several ER-resident chaperones were significantly upregulated, including DnaJ homolog subfamily A member 3 (DNAJA3, $p = 7.13\times10^{-7}$), HYOU1 ($p = 2.02\times10^{-4}$), and BiP (HSPA5, $p = 3.34\times10^{-2}$), indicating enhanced protein-folding capacity in response to proteotoxic stress.

Upregulation of components involved in **ER translocation and export**, such as Protein transport protein Sec61 subunit alpha isoform 1 (SEC61A1, $p = 4.73\times10^{-5}$), Translocation protein SEC63 homolog (SEC63, $p = 4.11\times10^{-4}$), transport protein SEC16 homolog A (SEC16A, $p = 5.76\times10^{-4}$), and Translocating chain-associated membrane protein 1 (TRAM1, $p = 3.59\times10^{-7}$), suggests increased flux of nascent polypeptides into the ER. Concurrently, elevated expression of Erlin-2 (ERLIN2, $p = 8.53\times10^{-7}$) supports activation of ER-associated protein degradation (ERAD), consistent with enhanced clearance of misfolded proteins.[68]

Evidence for **UPR signaling** was further supported by upregulation of Eukaryotic translation initiation factor 2 subunit 1 (EIF2S1, $p = 7.73\times10^{-5}$), a downstream target of PERK, and Heat shock protein 75 kDa (TRAP1, $p = 5.95\times10^{-5}$), a mitochondrial chaperone implicated in ER-mitochondrial stress signaling.[69] These changes are consistent with activation of the PERK–eIF2α–ATF4 axis and initiation of apoptosis under unresolved ER stress.

The transcription factor Apoptosis Antagonizing Transcription Factor (AATF, $p = 1.01\times10^{-4}$) was also upregulated, in line with its role as a **stress-responsive regulator linking nucleolar and ER homeostasis**. AATF is known to modulate p53-dependent transcription and attenuate apoptosis during both ribosomal and ER stress, suggesting an adaptive attempt to restore proteostasis following exposure to **3**.

Upregulation of ER membrane protein complex subunit 1 (EMC1, $p = 6.18\times10^{-7}$), a core component of the ER membrane protein complex involved in the insertion and folding of multipass membrane proteins, further supports **activation of adaptive proteostasis mechanisms**. This response is consistent with UPR induction triggered by exposure to **3**.[70,71]

Collectively, these findings indicate that **3** induces ER stress and activates proteostasis pathways across both ER and mitochondria (*vide supra*). Upregulation of DNAJA3 and TRAP1, mitochondrial Hsp40- and Hsp90-type chaperones that cooperate with Hsp70 in protein folding and apoptotic signaling, suggests coordinated chaperone activation across compartments.[72,73] This supports a model in which exposure to **3** elicits a **cross-compartmental stress response culminating in UPR-mediated apoptosis**.

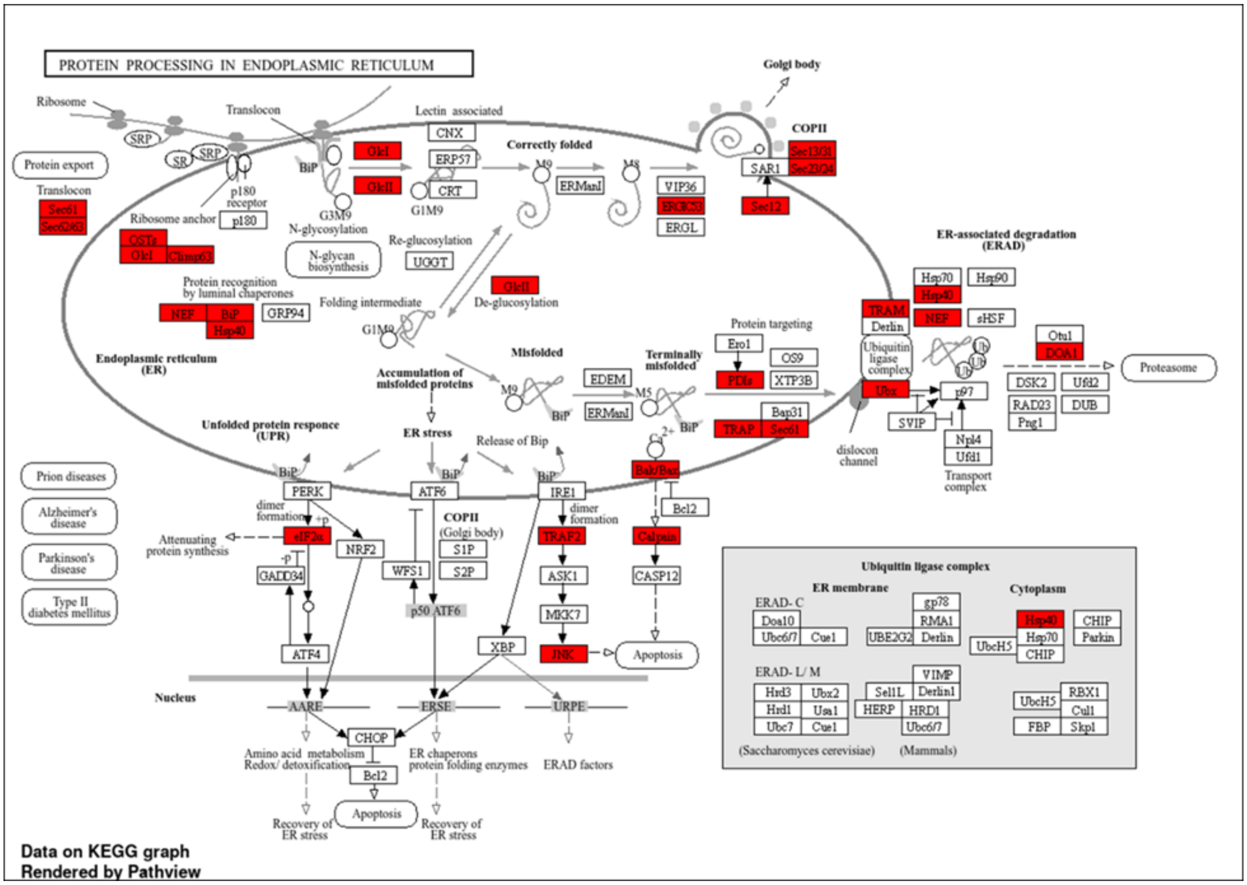

Figure S43. Effect of **3** on proteins included in protein processing in endoplasmatic reticulum (KEGG pathway hsa04141 protein processing in endoplasmatic reticulum). Significantly upregulated proteins are highlighted in red (ShinyGO 0.85).

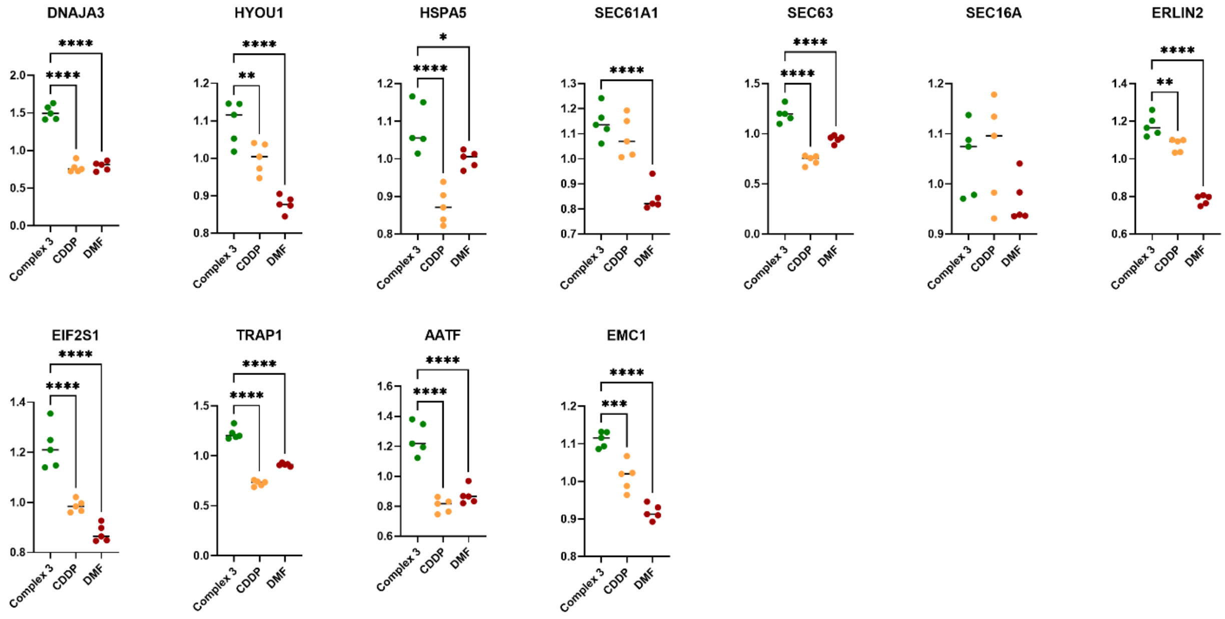

Figure S44. Box plots representing the effect of **3** on proteins included in protein processing in **endoplasmatic reticulum**. The box plots are labelled with gene names of proteins. The corresponding Uniprot name and accession number are summarized in Table S4. Significance codes (multiple-testing corrected): ****, ***, **, and * denote adjusted P values < 0.0001, < 0.001, < 0.01, and < 0.05, respectively.

Proteome analysis - metabolic rewiring. Consistent with the **glycolytic adaptation** described above, proteomic profiling revealed a **profound metabolic reprogramming** in A549 cells (Figure S45,S46) following treatment with **3**, indicative of a compensatory response to mitochondrial dysfunction. Enzymes linked to the oxidative **pentose phosphate pathway were upregulated**, including ER-localized hexose-6-phosphate dehydrogenase (H6PD, EC 1.1.1.363, p = 1.08×10^{-3}) and cytosolic 6-phosphogluconolactonase (6PGL, EC 3.1.1.31; p = 1.09×10^{-3}), indicating **enhanced NADPH production** to support **GSH recycling** under **3**-induced oxidative stress.

Most enzymes of the **citrate cycle** metabolism were reduced in abundance, including cytoplasmic aconitate hydratase (ACO1, EC 4.2.1.3, p = 4.60×10^{-5}), ATP-citrate synthase (ACLY, EC 2.3.3.8, p = 0.0122), mitochondrial citrate synthase (CS, EC 2.3.3.1, p = 0.0157), and both flavoprotein and iron–sulfur subunits of succinate dehydrogenase (SDHA, EC 1.3.5.1, p = 4.25×10^{-3}; SDHB, EC 1.3.5.1, p = 1.14×10^{-6}). Components of the pyruvate dehydrogenase complex (e.g., PDHB, EC 1.2.4.1, p = 8.01×10^{-5}) were likewise decreased, indicating limited entry of glycolytic carbon into the cycle. Interestingly, the **anaplerotic enzyme** pyruvate carboxylase[74] (PC, EC 6.4.1.1, $p = 3.37\times10^{-4}$) was also reduced, suggesting that under **3**-induced stress, mitochondrial carbon replenishment through the pyruvate–oxaloacetate route is suppressed. Glutaminase (GLS, EC 3.5.1.2, p = 2.13×10^{-2}) was modestly upregulated, indicating preserved capacity to convert glutamine to glutamate. However, concurrent **downregulation of key TCA cycle components** (PDHB, CS, ACO1, SDHA/B) and reduced PC argue against substantial glutamine-driven anaplerotic flux.

Instead, **glutamine utilization appears redirected toward redox and biosynthetic demands**, such as GSH synthesis and nucleotide production. Notably, strong upregulation of hexosamine biosynthetic enzymes (glutamine-fructose-6-phosphate aminotransferase 1 / 2, GFPT1 / 2, EC 2.6.1.16, p = 1.02×10^{-6} / 1.27×10^{-6}) suggests activation of this pathway as a compensatory response to impaired glycolytic input and mitochondrial oxidation. UDP-GlcNAc, the end-product of hexosamine biosynthetic pathway, functions as a metabolic sensor integrating nutrient availability (glucose, glutamine, acetyl-CoA, UTP) and stress signals, thereby modulating transcriptional and post-translational programs essential for cell survival[75] under **3**-induced stress. Upregulation of asparagine synthetase (ASNS, EC 6.3.5.4, p = 6.52×10^{-9}) further supports the notion that glutamine utilization in **3**-treated A549 cells is diverted from mitochondrial oxidation toward biosynthetic and stress-adaptive functions. ASNS catalyzes the ATP-dependent conversion of aspartate and glutamine to asparagine and glutamate, linking transaminase activity (aspartate aminotransferase 1 (cytoplasmic) / aspartate aminotransferase 2 (mitochondrial), GOT 1 / 2, EC 2.6.1.1, EC 2.6.1.3 / EC 2.6.1.7, p = $2{,}41\times10^{-4}$ / $2{,}45\times10^{-4}$) to nitrogen redistribution and amino acid homeostasis. This reinforces a non-anaplerotic glutamine flux that sustains protein synthesis and redox buffering under metabolic stress.

This likely reflects a **global downscaling of mitochondrial metabolism**, where both catabolic and anaplerotic fluxes are curtailed as cells shift toward cytosolic ATP and NADPH generation. In addition, the cytosolic $NADP^+$-dependent isocitrate dehydrogenase (IDH1, (EC 1.1.1.42, p = 1.05×10^{-3}) was upregulated, indicating a compensatory **increase in cytosolic NADPH production**. Since IDH1 catalyzes the oxidative decarboxylation of isocitrate to α-ketoglutarate outside mitochondria, its induction further supports a **metabolic shift of redox control from mitochondria to the cytosol**, ensuring sustained GSH regeneration under **3**-induced oxidative stress.

The most striking change was a marked increase in D-3-phosphoglycerate dehydrogenase (PHGDH, EC:1.1.1.95, p = 2.48×10^{-10}), the rate-limiting enzyme of the ***de novo* serine synthesis pathway**. PHGDH catalyzes the first step in the conversion of 3-phosphoglycerate - a glycolytic intermediate - into L-serine, a precursor for nucleotide, phospholipid, and GSH biosynthesis.[76] Its upregulation indicates a metabolic shift

toward **anabolic and redox-supporting pathways** that enable **survival under oxidative and energetic stress**. Since serine metabolism is tightly coupled to GSH production, enhanced PHGDH expression likely represents a protective response to **3**-induced ROS formation. This adaptive rewiring is characteristic of cancer cells seeking to preserve biosynthetic and antioxidant capacity despite impaired mitochondrial respiration.[77]

Consistent with this shift, phosphoserine aminotransferase (PSAT1; EC 2.6.1.52, $p = 1.63\times10^{-9}$), the second enzyme in the serine biosynthetic pathway, was among the most significantly upregulated proteins. PSAT1 catalyzes the transamination of 3-phosphohydroxypyruvate using glutamate as a nitrogen donor, yielding phosphoserine and α-ketoglutarate. Together with elevated GOT1/2 and modest GLS induction, this suggests that glutamine-derived carbon and nitrogen are rerouted through transaminase-mediated flux to support biosynthetic and redox demands rather than mitochondrial oxidation, while serine fuels GSH synthesis and one-carbon metabolism. These findings reinforce a model in which **3**-treated cells rewire glutamine metabolism to preserve antioxidant capacity and anabolic potential under oxidative stress.

In line with enhanced serine biosynthesis, monofunctional C1-tetrahydrofolate synthase (MTHFD1L; EC 6.3.4.3, $p = 3.65\times10^{-8}$) was significantly upregulated, indicating **activation of the mitochondrial one-carbon cycle**. This enzyme utilizes formate - derived from serine catabolism - to generate 10-formyltetrahydrofolate, a key donor for purine biosynthesis and methionine regeneration.[78] Together with elevated PHGDH[77] and PSAT1,[79] MTHFD1L induction suggests that **3**-treated cells channel serine-derived carbon into anabolic and redox-supporting pathways, reinforcing a survival strategy under oxidative stress.

Despite robust upregulation of serine biosynthetic enzymes (PHGDH, PSAT1), the downstream **integration of serine-derived one-carbon units appears functionally impaired**. Key cytosolic enzymes of the folate cycle - including C1-tetrahydrofolate synthase, cytoplasmic (MTHFD1; EC 1.5.1.5, $p = 8.02\times10^{-3}$), serine hydroxymethyltransferase (SHMT1; EC 2.1.2.1, $p = 5.43\times10^{-3}$), thymidylate synthase (TYMS; EC 2.1.1.45, $p = 2.19\times10^{-6}$), dihydrofolate reductase (DHFR; EC 1.5.1.3, $p = 1.97\times10^{-5}$), and methionine synthase (MS; EC 2.1.1.13, $p = 8.66\times10^{-3}$) - were significantly downregulated. This pattern suggests that the conversion of serine into glycine and folate derivatives, as well as their utilization for nucleotide synthesis and methylation, is compromised. Although methylenetetrahydrofolate reductase (MTHFR; EC 1.5.1.53, $p = 8.42\times10^{-3}$) was upregulated, the concurrent suppression of MS and methionine synthase reductase (MSR, EC 1.16.1.8, $p = 3.95\times10^{-3}$) likely limits methionine regeneration. Collectively, these findings indicate that **3**-treated cells activate serine biosynthesis but fail to fully engage the cytosolic one-carbon network, resulting in a metabolic bottleneck that may exacerbate oxidative and replicative stress.

A broad **upregulation of aminoacyl-tRNA synthetases and ligases** (Figure S46,47), including tryptophanyl-tRNA synthetase (TrpRS, EC 6.1.1.2, $p = 6.44\times10^{-8}$), glycyl-tRNA synthetase (GlyRS, EC 6.1.1.14, $p = 1.66\times10^{-7}$), tyrosyl-tRNA synthetase (TyrRS, EC 6.1.1.1, $p = 7.26\times10^{-7}$), and lysyl-tRNA synthetase (LysRS, EC 6.1.1.6, $p = 5.39\times10^{-4}$), reflecting **activation translation stress response**. Several of these enzymes are known to exert noncanonical functions beyond aminoacylation, including cytokine-like activity (e.g., TyrRS, TrpRS),[80,81] participation in stress signaling through the "alarmone" diadenosine tetraphosphate (Ap_4A; LysRS)[82] and immune modulation (endothelial monocyte-activating polypeptide II (EMAP-II); GlyRS).[83,84] In parallel, multiple tRNA-modifying enzymes - including RNA cytosine C(5)-methyltransferase NSUN2 (EC 2.1.1.-, $p = 3.22\times10^{-4}$) and tRNA (guanine(26)-N(2))-dimethyltransferase TRM1 (EC 2.1.1.216, $p = 7.86\times10^{-4}$) - were significantly upregulated, indicating **enhanced tRNA maturation and stability**. The coordinated upregulation of cytoplasmic aminoacyl-tRNA synthetases and tRNA-modifying enzymes suggests elevated translational activity in the cytosol, reflecting a concerted

translational and post-transcriptional response to **3**-induced stress that may support selective protein synthesis, redox adaptation, and cellular survival.

The significant upregulation of AMP-activated protein kinase (AMPK; EC 2.7.11.1) subunits - including α1 ($p = 9.17\times10^{-4}$), β1 ($p = 1.49\times10^{-2}$), γ1 ($p = 2.18\times10^{-5}$), and γ2 ($p = 3.05\times10^{-3}$) - indicates robust **activation of the AMPK complex**, a central metabolic sensor that responds to energy depletion and oxidative stress.[85] This activation aligns with the observed accumulation of diadenosine tetraphosphate (Ap_4A), a stress-induced alarmone generated by aminoacyl-tRNA synthetases.[82] Taken together, these observations indicate that **3**-treated cells engage Ap_4A–AMPK signaling to reprogram metabolism, **suppress anabolic processes**, and promote survival under conditions of mitochondrial dysfunction.

Fatty acid synthase (FASN; EC 2.3.1.85), the key enzyme for de novo lipogenesis, was significantly downregulated ($p = 4.18\times10^{-8}$). This suppression aligns with AMPK activation and global metabolic stress, suggesting a **shift away from anabolic lipid synthesis toward energy conservation and stress adaptation**.[86] In parallel, ACLY, a major cytosolic source of acetyl-CoA for fatty acid synthesis, was also reduced (see above), further limiting lipogenic flux.[85] Given the mitochondrial dysfunction and impaired one-carbon metabolism, reduced expression of both ACLY and FASN may serve to restrict lipotoxicity and oxidative damage under **3**-induced conditions.

The **glutathione/thioredoxin network showed clear remodeling**: although several cytosolic glutathione S-transferases declined, key enzymes such as glutathione synthetase, the mitochondrial GSH transporter SLC25A40, and microsomal GST1 (MGST1, EC 2.5.1.18, $p = 2.79\times10^{-5}$) were upregulated, indicating reinforcement of mitochondrial antioxidant defenses (see above). The combined activation of PHGDH-dependent serine metabolism, pentose phosphate pathways (PPP), and GSH-related pathways suggests an integrated metabolic defense program centered on **NADPH and thiol homeostasis**.

A pronounced **upregulation** of multiple enzymes involved in **pyrimidine metabolism** was also observed. Key enzymes of the de novo biosynthetic pathway, including dihydroorotate dehydrogenase (DHODH; EC 1.3.5.2, $p = 4.24\times10^{-5}$) and CTP synthetase (CTPS; EC 6.3.4.2, $p = 8.76\times10^{-4}$), were significantly upregulated, indicating enhanced pyrimidine nucleotide biosynthesis.

Additionally, increased expression of ribonucleoside-diphosphate reductase subunit M2 B (RRM2B; EC 1.17.4.1, $p = 2.33\times10^{-4}$) and multiple nucleoside diphosphate kinases (NDPKs; EC 2.7.4.6, e.g., NME3, $p = 6.59\times10^{-3}$) suggests **elevated deoxyribonucleotide production**, consistent with activation of DNA synthesis and repair. Upregulation of catabolic enzymes such as thymidine phosphorylase (TYMP; EC 2.4.2.4, $p = 9.72\times10^{-5}$) further indicates increased nucleotide turnover.

In parallel, several enzymes involved in **purine metabolism** were significantly upregulated, indicating coordinated activation of both purine and pyrimidine nucleotide biosynthesis. The trifunctional purine biosynthesis enzyme GART (EC 2.1.2.2 / 3.5.4.10 / 6.3.4.13), which is a key part of the multi-step conversion of phosphoribosyl pyrophosphate (PRPP) to inosine monophosphate (IMP),[87] was markedly induced ($p = 9.31\times10^{-4}$). Adenylosuccinate synthetase isozyme 1 (ADSSL1; EC 6.3.4.4, $p = 1.38\times10^{-5}$), which interconverts IMP and AMP to regulate nucleotide levels in various tissues,[88] also showed elevated expression. These changes were accompanied by the above-mentioned upregulation of mitochondrial one-carbon enzymes, e.g., mitochondrial bifunctional methylenetetrahydrofolate dehydrogenase/cyclohydrolase MTHFD2 (EC 1.5.1.15 / 3.5.4.9, $p = 6.95\times10^{-6}$) and mitochondrial serine hydroxymethyltransferase SHMT2 (EC 2.1.2.1, $p = 2.34\times10^{-5}$), which generate 10-formyltetrahydrofolate as a carbon donor for purine ring assembly. Together, these findings suggest that **3**-treated cells **enhance mitochondria–purinosome coupling**, redirecting serine-derived one-carbon units through the mitochondrial arm of the pathway to support de novo purine biosynthesis despite impaired cytosolic one-carbon metabolism. This metabolic rewiring likely reflects a compensatory response to **3**-induced DNA damage and replicative stress,

supporting nucleotide replenishment for repair and survival. Upregulation of adenylate kinase AK3 (EC 2.7.4.3, $p = 1.15\times10^{-5}$) and NAD^+ biosynthetic enzyme nicotinamide phosphoribosyltransferase (NAMPT; EC 2.4.2.12, $p = 8.24\times10^{-5}$) further supports tight regulation of nucleotide pools and redox balance under oxidative pressure.

Overall, these findings suggest that treatment with **3** induces a metabolic shift toward enhanced nucleotide metabolism, likely reflecting a cellular adaptation to **3**-induced stress and DNA damage. These metabolic changes, together with the increased production of ROS due to respiratory chain dysfunction, lead to the activation of the DNA damage response (DDR) and cell cycle arrest.

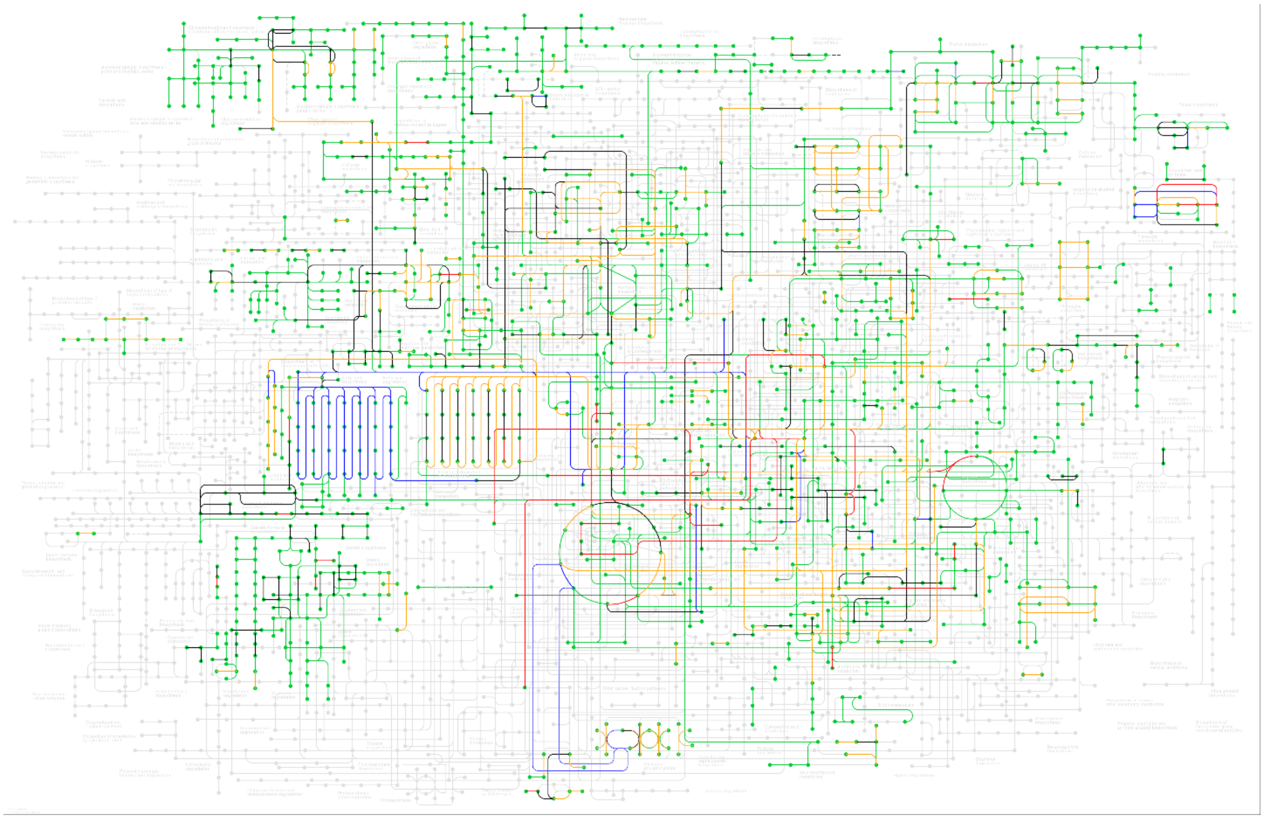

Figure S45. Effect of **3** on enzymes and proteins in whole human metabolism (KEGG pathway hsa01100 Metabolic pathways – *Homo sapiens* (human)). Bonferroni-significant alterations of protein levels are indicated in red (up-regulated) and blue (down-regulated); additional effects meeting uncorrected α < 0.05 are shown in orange (up-regulated) and black (down-regulated).

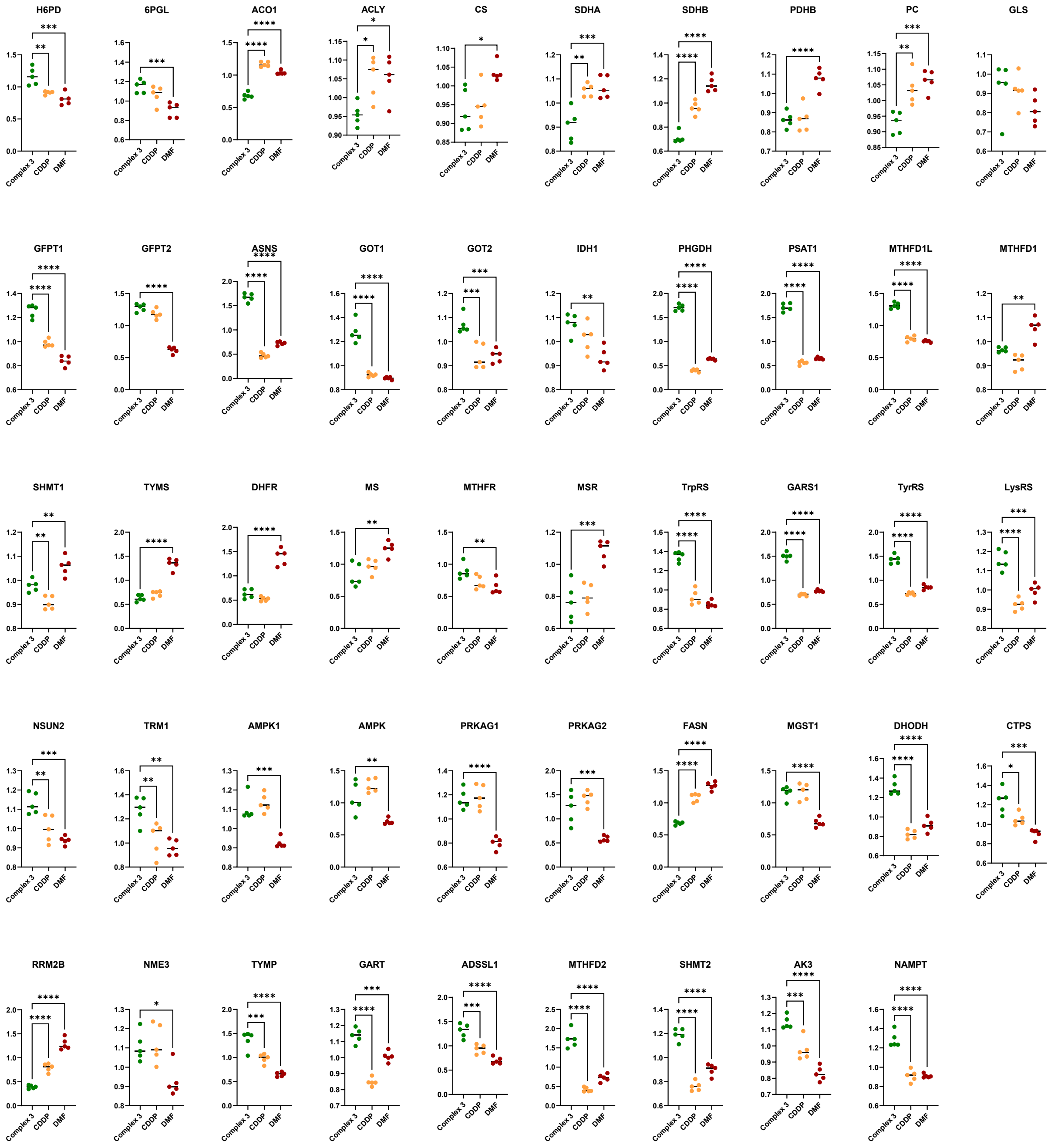

Figure S46. Box plots representing the effect of **3** on enzymes and proteins included in **metabolic remodeling** processes and aminoacyl-tRNA biosynthesis. The box plots are labelled with gene names of proteins. The corresponding Uniprot name and accession number are summarized in Table S4. Significance codes (multiple-testing corrected): ****, ***, **, and * denote adjusted P values < 0.0001, < 0.001, < 0.01, and < 0.05, respectively. Ordinary one-way ANOVA, Bonferroni´s multiple comparisons test.

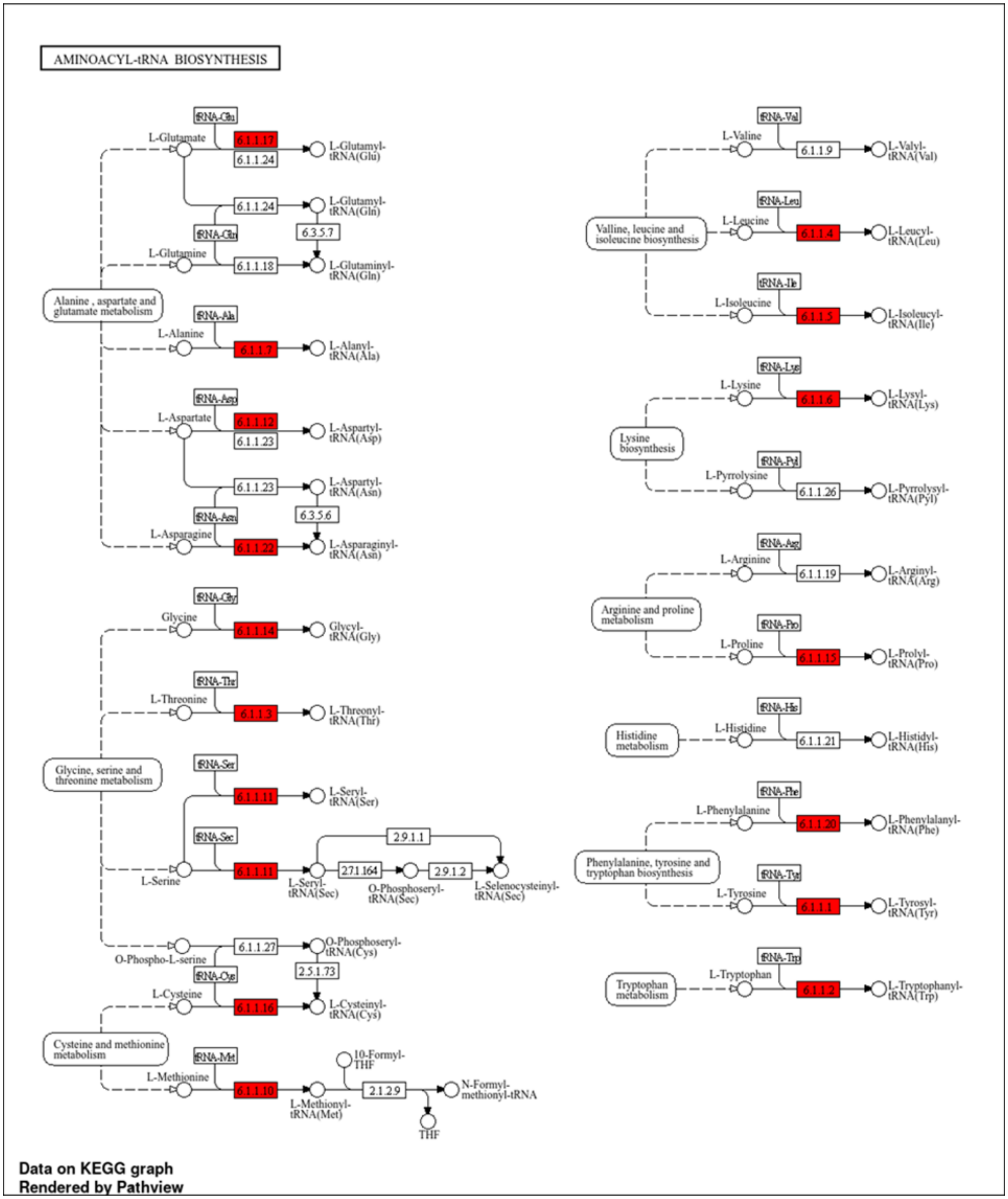

Figure S47. Effect of **3** on enzymes and proteins included in aminoacyl-tRNA biosynthesis (KEGG pathway hsa00970 aminoacyl-tRNA biosynthesis). Significantly upregulated proteins are highlighted in red (ShinyGO 0.85).

Proteome analysis - cell-cycle and apoptosis. Compound **3** causes **widespread suppression of the cell cycle** (Figure S48,S49), primarily by **blocking or slowing the G1/S transition** (where licensing and DNA replication are impaired and Cyclin-dependent kinases CDK1/4/6 (EC:2.7.11.22) are decreased), and by decreasing mitotic activity (where DNA topoisomerase 2-alpha (TOP2A, EC:5.6.2.2), p = 4.52×10^{-6}; KIFs, e.g. Kinesin-like protein KIF2C, p = 1.10×10^{-4}; Dual specificity protein kinase TTK, (EC:2.7.12.1), p = 1.72×10^{-3}; and Ki-67, p = 1.68×10^{-8} are downregulated). At the same time, the **p53-driven stress/DDR signature is active**. Functionally, this corresponds to a **cytostatic, quasi-quiescent state** (fewer entries into S phase and few mitotic events), rather than an acute mitotic catastrophe. This is consistent with metabolic findings of a weakened respiratory chain: cells slow down, conserve energy and survive, but do not practically divide.

The core effect of **3** on the cell cycle is the **suppression of the "licensing-replication" axis** (Figure S48,S49) and the simultaneous **attenuation of CDK-driven forces**. Key components of DNA replication licensing and elongation decrease quantitatively: MCM2–7 (*e.g.*, MCM2 p = 8.1×10^{-8}; MCM3 p = 1.4×10^{-9}), Proliferating cell nuclear antigen (PCNA, p = 6.8×10^{-5}), and replication polymerases α/δ/ε (EC:2.7.7.7) (*e.g.*, POLE, p = 7.1×10^{-6}; POLD1 p = 3.6×10^{-5}; POLA1 p = 7.5×10^{-4}). At the same time, there is a general decrease in the subunits of the clamp loader Replication factor C subunits RFC1–5 (*e.g.*, RFC3 p = 2.4×10^{-4}), which limits the attachment of PCNA to DNA and thus reduces polymerase productivity. In parallel, CDK1/2/4/6 (CDK4 p = 1.2×10^{-3}) and activating CDK7 (CDK7 p = 2.5×10^{-2}; CDK-activating kinase assembly factor MAT1 p = 5.6×10^{-3}), which reduces retinoblastoma protein (Rb) phosphorylation and suppresses E2F transcriptional output for S-phase genes. The result is fewer replication initiations and lower replication fork processivity (Figure S50), i.e., slowing or arrest in G1/S and at the intra-S checkpoint. This intervention profile will lead to an increase in the proportion of cells with 2N DNA content, with more cells remaining in G1 (some may transition to quiescent G0).

The same cell cycle modification as observed for **3** was also observed for other Ir compounds in HeLa cells[89] and A549 cells.[90] On the other hand, Sadler and co-workers reported that they observed S/G2 arrest in A2780 ovarian carcinoma cells treated by a half-sandwich Ir compound.[91] However, this does not contradict our results. The published S/G2 arrest is a typical early effect of replicative stress. Soon after exposure, the drug inhibits replication forks (PCNA/MCM/polymerases reduced), and the S-phase and G2 checkpoints are activated. Cells accumulate in S/G2 (which was observed after 24 h in ref.[91]). With longer exposure (as in our work), CDK4/6 and CDK2 decrease and there is no "hardware" for new replication (MCM, PCNA, RFC, Pol α/δ/ε), new cells cease to enter the S phase, and the G0/G1 population increases. Therefore, fewer cells enter the M phase. This is followed by a secondary decline in G2/M and spindle components, *e.g.*, TOP2A (p = 4.5×10^{-6}), KIF11 (p = 2.0×10^{-4}), KIF2C (p = 1.1×10^{-4}), and TTK (p = 1.7×10^{-3}); proliferation markers, *e.g.*, Ki-67 (p = 1.2×10^{-8}) (Figure S49). The pattern thus corresponds to reduced flow into mitosis, not mitotic blockade. The drug is therefore not "anti-spindle" in the style of taxanes/vinca alkaloids; it inhibits the cycle earlier (G1/S and intra-S) and the mitotic apparatus is suppressed as part of the overall suppressed proliferation program.

The data show that the suppression of licensing and elongation of DNA replication (decrease in MCM/PCNA/polymerases; *vide supra*) triggers the activa**tion of DNA damage and replication stress response** involving the **p53 pathway**. There is an increase in classic p53 target genes/proteins (Figure S49), such as PDRG1 (p = 1.3×10^{-3}), TP53I3 (p = 1.7×10^{-3}), PUMA (p = 2.7×10^{-3}), and p53R2 (p = 2.3×10^{-4}), which enforce cell cycle checkpoints and shift the balance between repair and apoptosis toward temporary cycle arrest and DNA repair (pro-survival) rather than massive apoptosis.

Compared to CDDP, **3** causes a more noticeable decrease in PCNA and especially CDK4, while CDDP tends to leave CDK4 unchanged or slightly increased. This shows that **3** not only blocks the

"hardware" of replication (MCM-PCNA-polymerases together with RFC), but also inhibits the "starter" of the cycle at the CDK4/6-RB-E2F level, thereby more consistently closing the entry into the S phase. In addition, POLG1, which is responsible for the replication and repair of mtDNA, also decreases ($p = 3.7\times10^{-5}$), which may contribute to energy stress in the long term, but the primary picture in the nucleus is replication attenuation.

A comparison of p53-mediated responses shows that the substances activate different branches of the DDR pathway (Figure S49,S51). CDDP induces a genotoxic/repair-biased profile, with stronger induction of TP53I3 and p53R2 indicating a predominance of direct DNA lesions (intrastrand/interstrand crosslinks accompanied by ROS) and the activation of the repair program. TP53I3 is associated with the oxidative response and apoptosis, while p53R2 provides deoxyribonucleoside triphosphates (dNTPs) for repairs even outside the S phase. This pattern is typical of direct DNA damage, which the cell primarily attempts to repair.

In contrast, **3** exhibits priming/arrest bias hallmarks: relatively higher PUMA and selective PDRG1 induction are indicative of mitochondrial/replication stress and apoptotic priming rather than massive repair program activation. The weaker response of p53R2 and TP53I3 compared to CDDP is consistent with less direct DNA destruction and more pronounced replication suppression (a simultaneous decline in MCM, PCNA and polymerases, as well as CDK4/6). Functionally, cycle arrest and increased readiness for apoptosis prevail over immediate execution.

In addition to the suppression of replication licensing and CDK-driven cell cycle progression, **3** also downregulates the SUMO-conjugating enzyme UBC9 (EC:2.3.2.-, $p = 1.11\times10^{-6}$), the sole E2 enzyme responsible for SUMOylation. This affects the post-translational modification of key stress regulators, including p53.[91] Reduced SUMOylation may destabilize p53 and alter its transcriptional output, shifting the DNA damage response (DDR) away from repair-oriented signaling and toward apoptotic priming.[92] This is consistent with the observed increase in PUMA and PDRG1, and the relatively weaker induction of TP53I3 and p53R2, suggesting that cells enter a cytostatic, quasi-quiescent state with elevated apoptotic readiness, rather than engaging in robust DNA repair or mitotic progression.

In line with this priming-biased DDR profile, **3** also induces a coordinated mitochondrial response characteristic of **intrinsic apoptotic readiness** (Figure S52). Intrinsic apoptotic priming reflects a BCL-2 family–regulated mitochondrial state that determines the ease with which cells undergo apoptosis in response to stress.[93] Compared to CDDP, which primarily elevates BAX in a repair-oriented context, **3** upregulates a broader set of mitochondrial effectors, including Diablo IAP-binding mitochondrial protein (DIABLO/SMAC, $p = 5.16\times10^{-5}$), mitochondrial Serine protease HTRA2 (EC 3.4.21.108, $p = 7.30\times10^{-4}$), Non-selective voltage-gated ion channel VDAC1 and VDAC2 ($p = 2.92\times10^{-3}$, $p = 5.07\times10^{-4}$, resp.), ADP/ATP translocase 1 and 2 (ANT1, $p = 5.54\times10^{-5}$; ANT2, $p = 2.44\times10^{-5}$), and the fission regulator Mitochondrial fission 1 protein (FIS1, $p = 8.99\times10^{-4}$). This pattern suggests enhanced mitochondrial fragmentation, increased susceptibility to mitochondrial outer membrane permeabilization (MOMP), and elevated inhibitor of apoptosis protein (IAP) antagonism. DIABLO/SMAC and HTRA2 are released from mitochondria during apoptotic signaling and promote caspase activation by neutralizing IAP proteins.[94–98] Likewise, VDAC2 and ANT proteins are established modulators of mitochondrial membrane permeabilization and apoptotic competence, whereas FIS1 is associated with a pro-fission mitochondrial state that frequently accompanies apoptotic remodeling.[99–103] Collectively, these findings indicate that cells exposed to 3 enter a cytostatic state with markedly higher intrinsic apoptotic priming rather than prioritizing DNA repair and survival signaling.

At the same time, increased abundance of proteins associated with death receptor signaling, including Tumor necrosis factor receptor superfamily member 10A (TNFRSF10A, $p = 1.49\times10^{-6}$), Tumor

necrosis factor receptor superfamily member 10B (TNFRSF10B, $p = 1.41\times10^{-2}$), Tumor necrosis factor receptor type 1-associated DEATH domain protein (TRADD, $p = 3.57\times10^{-2}$), and TNF receptor-associated factor 2 (TRAF2, EC 2.3.2.27, $p = 1.95\times10^{-3}$), indicates activation of signaling modules canonically linked to the **extrinsic apoptotic pathway** and death receptor responses. TNFRSF10A and TNFRSF10B encode the TRAIL death receptors DR4 and DR5, which initiate receptor-mediated apoptosis upon ligand binding,[104] whereas TRADD and TRAF2 are established adaptor proteins coordinating downstream TNF receptor signaling complexes that regulate the balance between apoptotic and pro-survival outputs.[105,106] Because extrinsic death receptor signaling can amplify mitochondrial apoptosis through BID-dependent crosstalk,[107–109] these findings support a mixed apoptotic response in which **mitochondrial signaling predominates**, while receptor-mediated pathways may provide an auxiliary pro-death input.

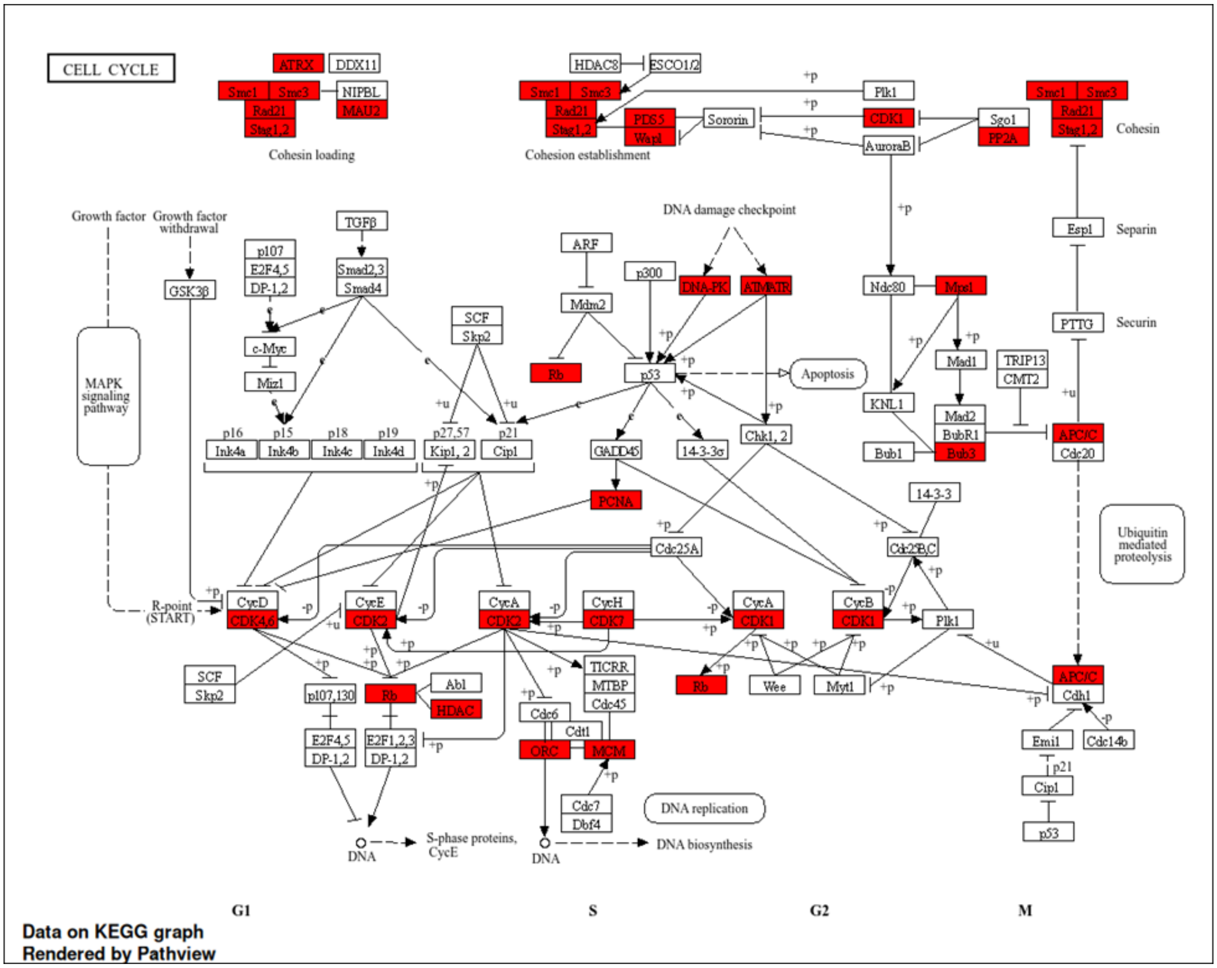

Figure S48. Effect of **3** on proteins included in the cell cycle (KEGG pathway hsa04110 Cell cycle). Significantly reduced proteins are highlighted in red (ShinyGO 0.85).

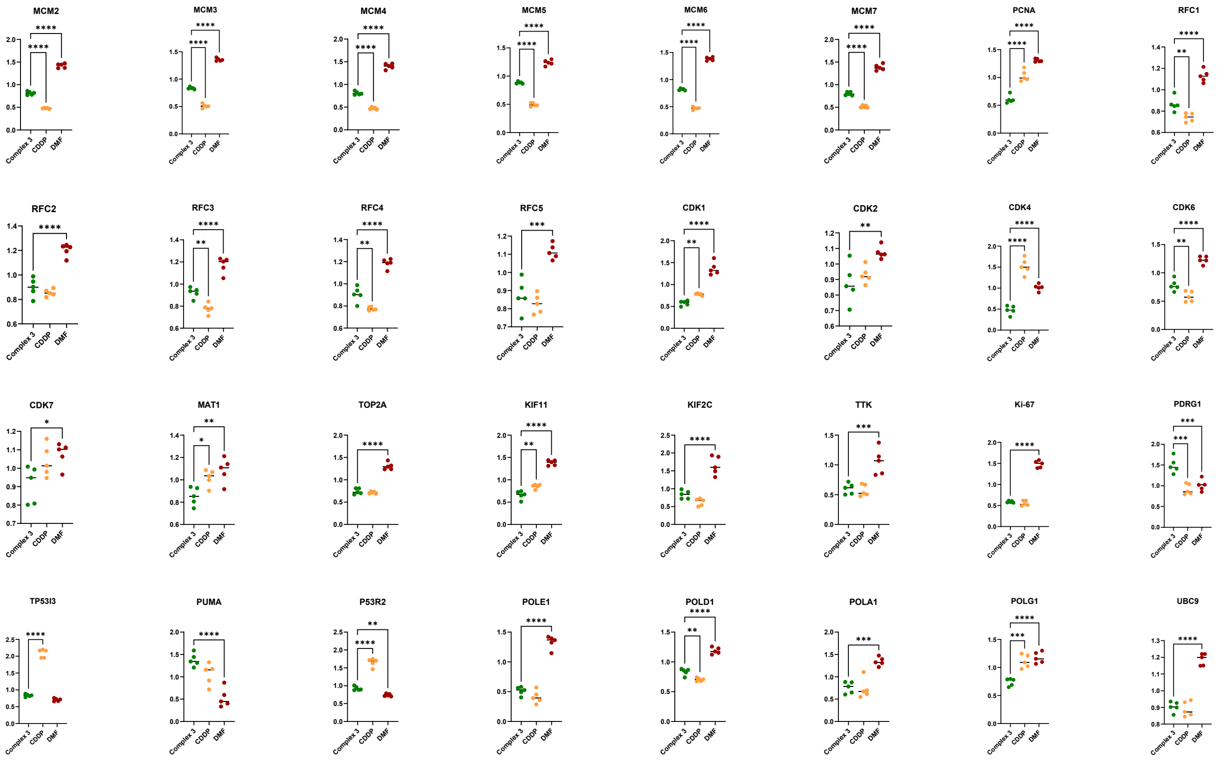

Figure S49. Box plots representing the effect of **3** on enzymes and proteins included in **cell cycle processes, replication, repair of DNA mismatch errors**. The box plots are labelled with gene names of proteins. The corresponding Uniprot name and accession number are summarized in Table S4. Significance codes (multiple-testing corrected): ****, ***, **, and * denote adjusted P values < 0.0001, < 0.001, < 0.01, and < 0.05, respectively. Ordinary one-way ANOVA, Bonferroni´s multiple comparisons test.

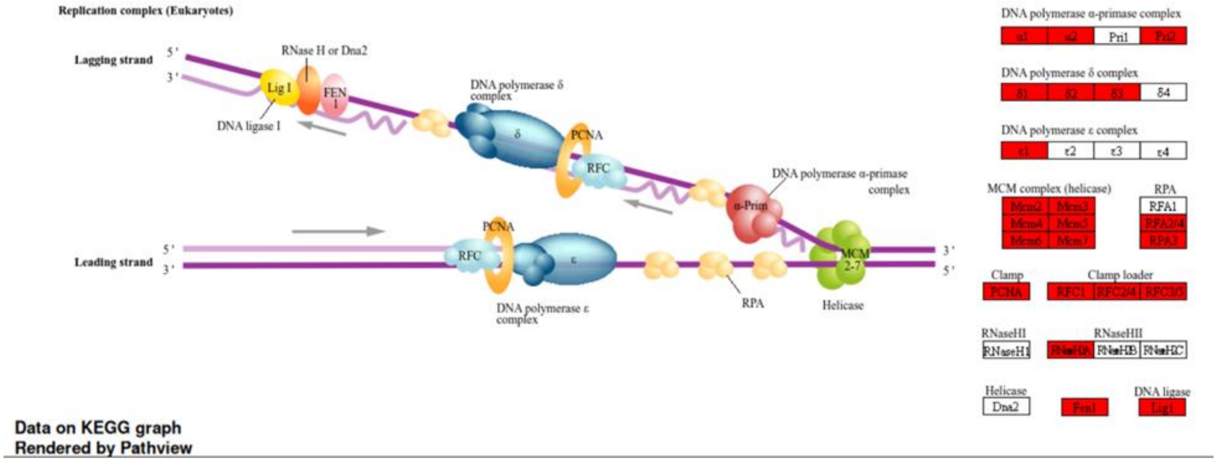

Figure S50. Effect of **3** on proteins included in the replication complex (KEGG pathway hsa03030 DNA replication). Significantly reduced proteins are highlighted in red (ShinyGO 0.85).

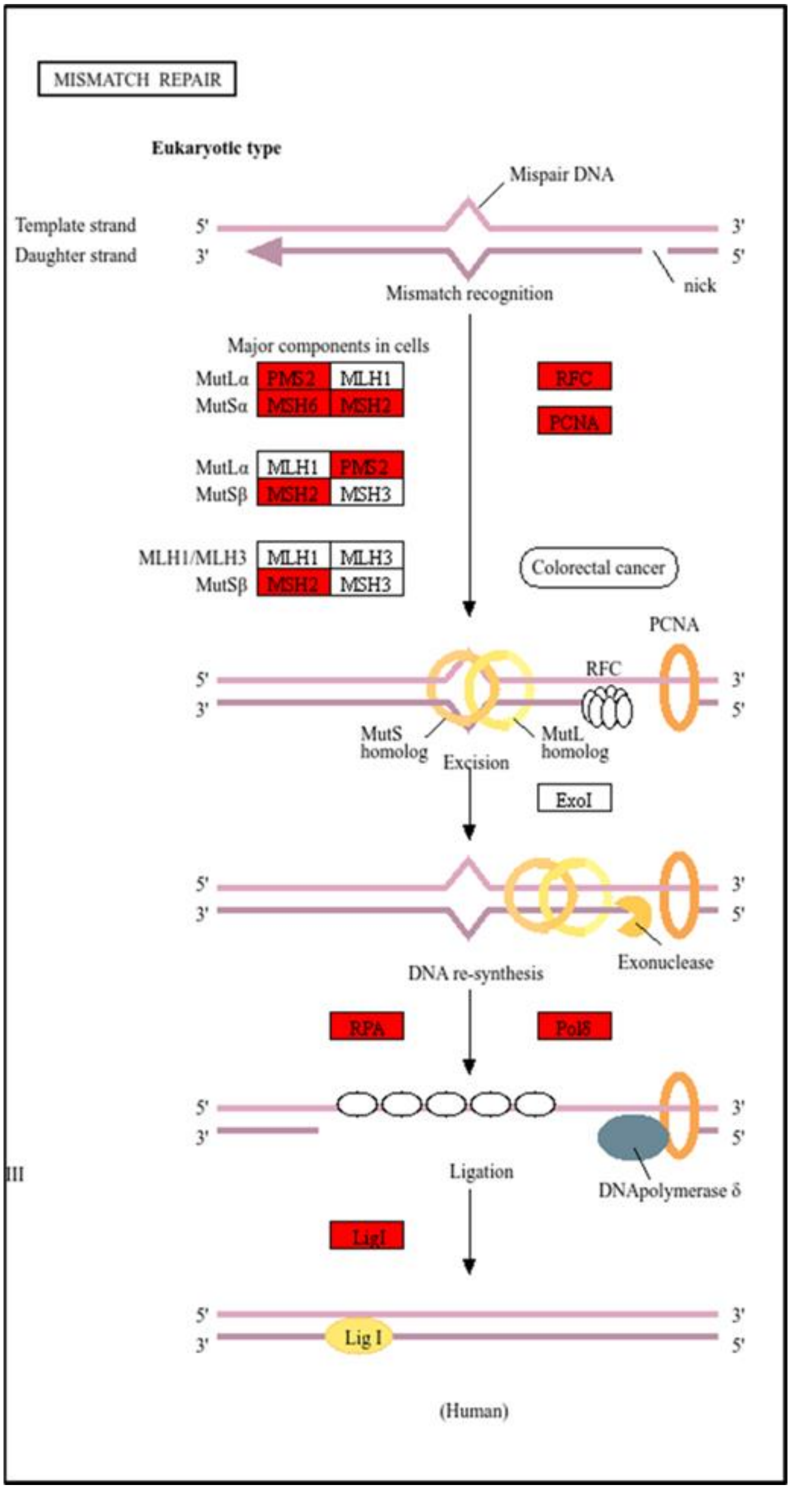

Figure S51. Effect of **3** on proteins included in the processes of mismatch repair of DNA in eukaryotic cells (KEGG pathway hsa03430 mismatch repair). Significantly reduced proteins are highlighted in red (ShinyGO 0.85).

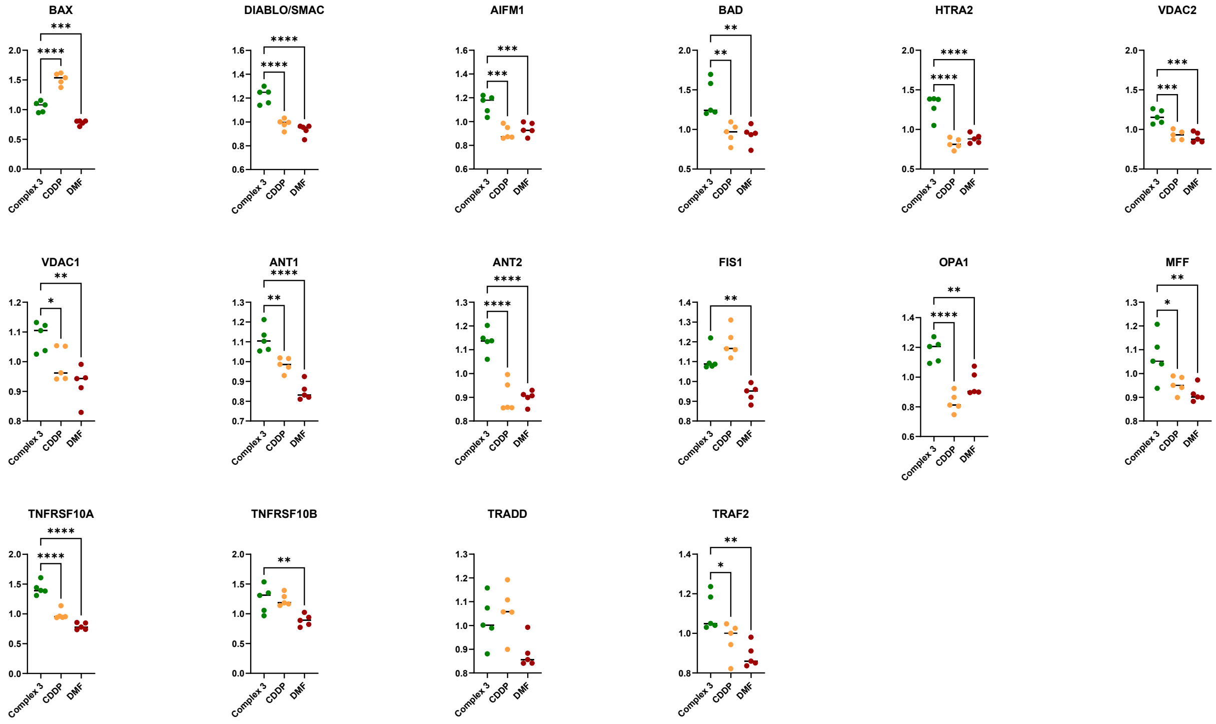

Figure S52. Box plots representing the effect of **3** on proteins involved in **intrinsic and extrinsic apoptotic pathways**. The box plots are labelled with gene names of the quantified proteins. The corresponding UniProt names and accession numbers are summarized in Table S4. Significance codes (multiple-testing corrected): ****, ***, , and * denote adjusted P values < 0.0001, < 0.001, < 0.01, and < 0.05, respectively. Ordinary one-way ANOVA, Bonferroni's multiple comparisons test.

Proteomics-based mechanistic summary. Proteomic profiling reveals that **3** exerts multifaceted stress on cancer cells by (1) perturbing nucleolar function and ribosome biogenesis, (2) remodeling the endolysosomal system toward elevated uptake but reduced degradative capacity, (3) accumulating in and impairing mitochondrial translation and respiratory complexes - thereby eliciting pronounced metabolic reprogramming - and (4) enforcing replication-licensing defects that culminate in p53-mediated cell-cycle arrest with apoptotic priming. These interconnected processes converge to produce a predominantly cytostatic phenotype characterized by metabolic adaptation and heightened vulnerability to secondary apoptotic stimuli (see (Figure 9 and S30–S52).

Previous investigations of Ir(III) anticancer agents have demonstrated mitochondrial localization, induction of ROS, ATP depletion and cell-cycle arrest rather than classical DNA cross-linking mechanisms.[110–112] Our finding that **3** also remodels the endolysosomal network and perturbs ribosome biogenesis therefore extends the mechanistic landscape of Ir(III) compounds, suggesting a multifactorial mode of action involving organelle-specific accumulation and proteome-wide stress responses.

Analogous ribosome-biogenesis targeting has been described for a Ru(II) monoadduct generating complex,[113] indicating that ribosome biogenesis stress is emerging as a relevant MoA for metal-based anti-cancer drugs. In light of the pivotal role of ribosome biogenesis in the proliferation of cancer cells,[114,115] the observed shift suggests that **3** may exploit a 'ribosome addiction' vulnerability in A549 cells.

Multiple studies have established that Ir(III) compounds can accumulate in lysosomes, including in A549 cells, and may perturb lysosomal integrity upon activation (e.g., lysosome-targeted photodynamic therapy agents).[51,116] Reports also indicate that Ir(III) species can enter cells via endocytic pathways,[117,118] consistent with clathrin-mediated uptake mechanisms broadly supported by the roles of PICALM and adaptor protein complexes in cargo internalization. These observations align with our proteomic evidence for enhanced early endocytic input and altered lysosomal function in **3**.

Independent work has shown that Ir(III) complexes can localize to mitochondria, decrease membrane potential and ATP, and elevate ROS in A549 cells, supporting a mitochondrial contribution to cytotoxicity.[119] Our data that **3** accumulates in mitochondria and dampens mitochondrial translation/respiration are therefore congruent with prior reports, while extending them by linking mitochondrial stress to endolysosomal remodeling in the same cellular context.

While lysosomal localization and mitochondrial dysfunction are well documented for Ir(III) agents,[120] the concurrent remodeling of the endolysosomal network toward elevated uptake but reduced degradative capacity together with ribosome-biogenesis (nucleolar) stress has, to our knowledge, not been explicitly described for Ir(III) compounds. Prior phenotypic studies emphasize non-DNA MoA for Ir(III) compounds; our findings add a proteomics-based model that connects endolysosomal, nucleolar, and mitochondrial axes into a unified, predominantly cytostatic outcome for a new half-sandwich Ir(III) compound **3**.

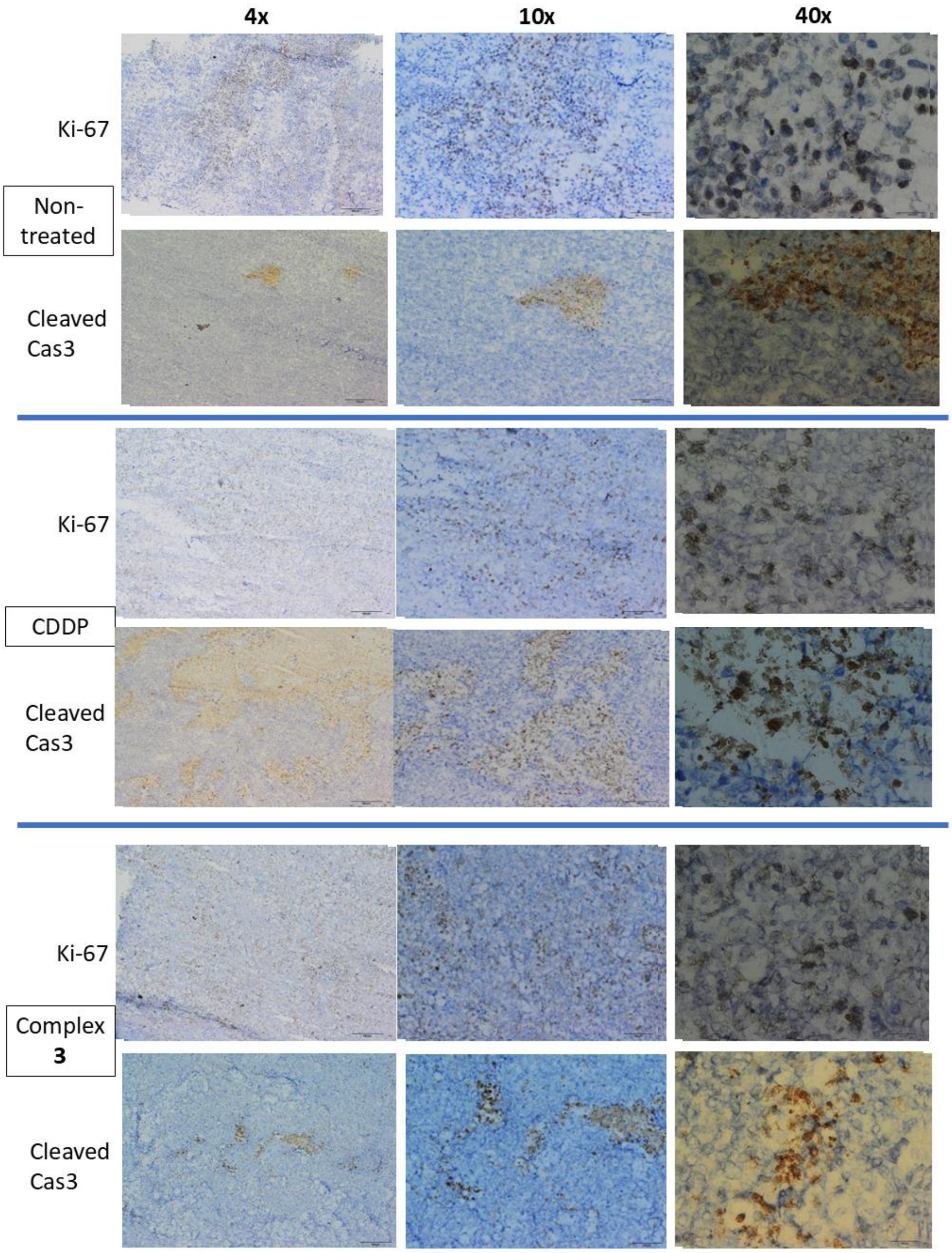

Figure S53. Immunohistochemistry analysis of cancer samples. Antibodies anti-Ki67 and anti-Cleaved Caspase 3 were applied on processed slides. Then, samples were stained with Liquid DAB+ substrate chromogen system. Finally, the slides were lightly counterstained with Mayer hematoxylin. Magnification 4×, 10×, and 40×.

Table S1. Crystallographic data for **3**.

Chemical formula	$C_{25}H_{33}ClF_6IrNP2$
M_r	751.11
Crystal system	monoclinic
Space group	$P2_1/c$
T / K	298.0(5)
a / Å	10.46250(10)
b / Å	14.80980(10)
c / Å	18.09150(10)
α / °	90
β / °	96.8070(10)
γ / °	90
V / Å^3	2783.47(4)
Z	4
λ / Å	1.54184
μ / mm^{-1}	11.759
Crystal size / mm	0.22 × 0.15 × 0.12
ρ_{calc} / g.cm^{-3}	1.792
2Θ range for data collection/°	7.736 to 143.334
Index ranges	$-12 \leq h \leq 12$, $-18 \leq k \leq 18$, $-22 \leq l \leq 21$
Reflections collected	29600
Independent reflections	5379 [R_{int} = 0.0368, R_{sigma} = 0.0204]
Data/restraints/parameters	5379/690/401
Goodness-of-fit on F^2	1.058
Final R indexes [I>=2σ (I)]	R_1 = 0.0257, wR_2 = 0.0704
Final R indexes [all data]	R_1 = 0.0277, wR_2 = 0.0718
CCDC	2293608

Table S2. Crystallographic data for **3***·MeOH.

Chemical formula	$C_{26}H_{37}Cl_2IrNOP$
M_r	673.63
Crystal system	orthorhombic
Space group	*Pbca*
T / K	100.0(1)
a / Å	8.74990(10)
b / Å	21.0479(3)
c / Å	28.6010(3)
α / °	90
β / °	90
γ / °	90
V / Å^3	5267.36(11)
Z	8
λ / Å	1.54184
μ / mm^{-1}	12.393
Crystal size / mm	0.13 × 0.07 × 0.04
ρ_{calc} / g.cm^{-3}	1.699
2Θ range for data collection/°	8.402 to 136.82
Index ranges	$-10 \le h \le 7$, $-25 \le k \le 22$, $-34 \le l \le 28$
Reflections collected	4780
Independent reflections	4228 [R_{int} = 0.0294, R_{sigma} = 0.0282]
Data/restraints/parameters	4780/0/296
Goodness-of-fit on F^2	1.015
Final R indexes [I>=2σ (I)]	R_1 = 0.0307, wR_2 = 0.0839
Final R indexes [all data]	R_1 = 0.0355, wR_2 = 0.0868
CCDC	2300188

Table S3. Selected bond lengths (Å) and angles (°) determined for **3** and **3***·MeOH by a single-crystal X-ray analysis. C_g = the centroid of the η^5-coordinated Cp* ring.

Bond	**3**	**3***
Ir–Cl	2.4044(9)	2.4046(10)
Ir–N	2.154(3)	2.134(3)
Ir–P	2.2903(8)	2.3051(12)
Ir–C_g	1.8310(4)	1.826(2)
Angle		
N–Ir–P	88.70(9)	86.58(10)
N–Ir–Cl	86.49(10)	86.58(10)
N–Ir–C_g	127.89(9)	123.38(9)
P–Ir–Cl	87.87(3)	86.83(4)
P–Ir–C_g	133.11(3)	132.76(8)
Cl–Ir–C_g	118.56(4)	123.55(8)

Table S4. Summary of accession number, protein ID, protein name and gene names of all protein mentioned in chapters of discussion of proteomic results and different mechanisms of action of **3** are summarized in the table. The table is sorted alphabetically according to the first gene name, corresponding to the gene names used in the titles of the box plots.

UniProt Accesion	Uniprot ID	protein name	gene names
O95336	6PGL_HUMAN	cytosolic 6-phosphogluconolactonase (EC 3.1.1.31)	6PGL
Q9NY61	AATF_HUMAN	Protein AATF (transcription factor Apoptosis Antagonizing Transcription Factor)	AATF, CHE1, DED, HSPC277
P53396	ACLY_HUMAN	ATP-citrate synthase (EC 2.3.3.8)	ACLY
P21399	ACOHC_HUMAN	cytoplasmic aconitate hydratase (EC 4.2.1.3)	ACO1
Q8N142	PURA1_HUMAN	Adenylosuccinate synthetase-like 1 (Adenylosuccinate synthetase isozyme 1; EC 6.3.4.4)	ADSSL1
Q53H12	AGK_HUMAN	Acylglycerol kinase, mitochondrial (EC:2.7.1.107, EC:2.7.1.94)	AGK
O95831	AIFM1_HUMAN	Apoptosis-inducing factor 1, mitochondrial	AIFM1, AIF, PDCD8
Q12904	AIMP1_HUMAN	Aminoacyl tRNA synthase complex-interacting multifunctional protein 1 (endothelial monocyte-activating polypeptide II (EMAP-II))	AIMP1, EMAP2, SCYE1
Q9UIJ7	KAD3_HUMAN	GTP:AMP phosphotransferase AK3, mitochondrial, (EC:2.7.4.10)	AK3, AK3L1, AK6, AKL3L
P54886	P5CS_HUMAN	Delta-1-pyrroline-5-carboxylate synthase (EC:2.7.2.11)	ALDH18A1, GSAS, P5CS
P30837	AL1B1_HUMAN	Aldehyde dehydrogenase X, mitochondrial (EC:1.2.1.3)	ALDH1B1
P12235	ADT1_HUMAN	ADP/ATP translocase 1	ANT1, SLC25A4, AAC1
P05141	ADT2_HUMAN	ADP/ATP translocase 2	ANT2, SLC25A5, AAC2
O43747	AP1G1_HUMAN	AP-1 complex subunit gamma-1	AP1G1
O94973	AP2A2_HUMAN	AP-2 subunits (e.g. subunit alpha-2	AP2A2, ADTAB, CLAPA2, HIP9, HYPJ, KIAA0899

Table S4 (continued)

P08243	ASNS_HUMAN	asparagine synthetase (EC 6.3.5.4)	ASNS
Q9H1Y0	ATG5_HUMAN	Autophagy protein 5	ATG5, APG5L, ASP
Q15904	VAS1_HUMAN	vacuolar-type ATPase (V-ATPase), such as S1	ATP6AP1, ATP6IP1, ATP6S1
P36543	VATE1_HUMAN	V-type proton ATPase subunit E 1 (V-ATPase subunit E 1) (V-ATPase 31 kDa subunit) (p31) (Vacuolar proton pump subunit E 1)	ATP6V1E1 ATP6E ATP6E2
Q07812	BAX_HUMAN	Apoptosis regulator BAX	BAX
Q92934	BAD_HUMAN	Bcl2-associated agonist of cell death	BAD, BBC6, BCL2L8
Q9BXH1	BBC3_HUMAN	Bcl-2-binding component 3, isoforms ½	BBC3, PUMA
P06493	CDK1_HUMAN	Cyclin-dependent kinase 1 (EC:2.7.11.22, EC:2.7.11.23)	CDK1, CDC2, CDC28A, CDKN1, P34CDC2
P24941	CDK2_HUMAN	Cyclin-dependent kinase 2 (EC:2.7.11.22)	CDK2, CDKN2
P11802	CDK4_HUMAN	Cyclin-dependent kinase 4 (EC:2.7.11.22)	CDK4
Q00534	CDK6_HUMAN	Cyclin-dependent kinase 6 (EC:2.7.11.22)	CDK6, CDKN6
P50613	CDK7_HUMAN	Cyclin-dependent kinase 7 (EC:2.7.11.22,EC:2.7.11.23)	CDK7, CAK, CAK1, CDKN7, MO15, STK1
P49336	CDK8_HUMAN	Cyclin-dependent kinase 8 (EC:2.7.11.22)	CDK8
Q9H078	CLPB_HUMAN	Mitochondrial disaggregase - CLPB	CLPB
O76031	CLPX_HUMAN	ATP-dependent ClpX-like chaperone	CLPX
P15954	COX7C_HUMAN	Cytochrome c oxidase subunit 7C, mitochondrial of complex IV	COX7C
P31327	CPSM_HUMAN	Carbamoyl-phosphate synthase [ammonia], mitochondrial (EC:6.3.4.16)	CPS1
O75390	CISY_HUMAN	mitochondrial citrate synthase (EC 2.3.3.1)	CS
P17812 or Q9NRF8	PYRG1_HUMAN or PYRG2_HUMAN	CTP synthetase (CTPS, EC 6.3.4.2) isoform 1 or 2	CTPS1 or CTPS2
C9JH19	C9JH19_HUMAN	Cathepsin D (EC 3.4.23.5)	CTSD

Table S4 (continued)

P07711	CATL1_HUMAN	Procathepsin L	CTSL
P25774	CATS_HUMAN	Cathepsin S	CTSS
Q9UBR2	CATZ_HUMAN	Cathepsin Z	CTSZ
Q9NR28	DBLOH_HUMAN	Diablo IAP-binding mitochondrial protein	DIABLO, SMAC
P00374	DYR_HUMAN	dihydrofolate reductase (EC 1.5.1.3)	DHFR
Q02127	PYRD_HUMAN	dihydroorotate dehydrogenase (EC 1.3.5.2)	DHODH
O60832	DKC1_HUMAN	H/ACA ribonucleoprotein complex subunit DKC1 – 100% HOMOLOG pro A0A8V8TKY9 (A0A8V8TKY9_HUMAN)	DKC1
Q96EY1	DNJA3_HUMAN	DnaJ homolog subfamily A member 3, mitochondrial	DNAJA3
O00115	DNS2A_HUMAN	Deoxyribonuclease-2-alpha (EC:3.1.22.1)	DNASE2, DNASE2A
P05198	IF2A_HUMAN	Eukaryotic translation initiation factor 2 subunit 1	EIF2S1, EIF2A
P41091	IF2G_HUMAN	Eukaryotic translation initiation factor 2 subunit 3	EIF2S3
O43432	IF4G3_HUMAN	Eukaryotic translation initiation factor 4 gamma 3	EIF4G3
Q53HC9	EIPR1_HUMAN	EARP and GARP complex-interacting protein 1	EIPR1
Q8N766	EMC1_HUMAN	ER membrane protein complex subunit 1	EMC1
Q92979	NEP1_HUMAN	Ribosomal RNA small subunit methyltransferase NEP1	EMG1
O94905	ERLN2_HUMAN	Erlin-2	ERLIN2
Q96KP1	EXOC2_HUMAN	Exocyst complex component 2	EXOC2, SEC5, SEC5L1
Q96A65	EXOC4_HUMAN	Exocyst complex component 4	EXOC4, KIAA1699, SEC8, SEC8L1
Q9UPT5	EXOC7_HUMAN	Exocyst complex component 7	EXOC7, EXO70, KIAA1067
P49327	FAS_HUMAN	Fatty acid synthase (FASN; EC 2.3.1.85)	FASN
Q9Y3D6	FIS1_HUMAN	Mitochondrial fission 1 protein	FIS1, TTC11, CGI-135
Q9NY12	GAR1_HUMAN	H/ACA ribonucleoprotein complex subunit 1	GAR1, NOLA1
A0A6Q8 PGI6	A0A6Q8PGI6_HU MAN	Glycine--tRNA ligase (EC 6.1.1.14) (Diadenosine tetraphosphate synthetase)	GARS1

Table S4 (continued)

P22102	PUR2_HUMAN	Trifunctional purine biosynthetic protein adenosine-3 (trifunctional purine biosynthesis enzyme GART (EC 6.3.4.13 / EC 6.3.3.1/ EC 2.1.2.2)	GART
P04062	GBA1_HUMAN	Lysosomal acid glucosylceramidase (Lysosomal acid GCase) (EC 3.2.1.45) (Acid beta-glucosidase) (Alglucerase)	GBA1 GBA GC GLUC
P31150	GDIA_HUMAN	Rab GDP dissociation inhibitor alpha	GDI1
Q06210	GFPT1_HUMAN	glutamine--fructose-6-phosphate aminotransferase 1 EC 2.6.1.16)	GFPT1
O94808	GFPT2_HUMAN	glutamine--fructose-6-phosphate aminotransferase 2, EC 2.6.1.16)	GFPT2
B8ZZC5	B8ZZC5_HUMAN	glutaminase (EC 3.5.1.2)	GLS
P17174	AATC_HUMAN	Aspartate aminotransferase, cytoplasmic (aspartate aminotransferase 1 (cytoplasmic) EC 2.6.1.1)	GOT1
P00505	AATM_HUMAN	Aspartate aminotransferase, mitochondrial (aspartate aminotransferase 2 (mitochondrial) EC 2.6.1.3)	GOT2
P48637	GSHB_HUMAN	Glutathione synthetase (EC:6.3.2.3)	GSS
O95479	G6PE_HUMAN	GDH/6PGL endoplasmic bifunctional protein (ER-localized hexose-6-phosphate dehydrogenase, EC 1.1.1.363)	H6PD
Q9H583	HEAT1_HUMAN	HEAT repeat-containing protein 1	HEATR1
Q2TB90	HKDC1_HUMAN	Hexokinase HKDC1 (EC 2.7.1.1)	HKDC1
P52597	HNRPF_HUMAN	Heterogeneous nuclear ribonucleoprotein F	HNRNPF
P11021	BIP_HUMAN	Endoplasmic reticulum chaperone BiP	HSPA5
P38646	HSPA9_HUMAN	Stress-70 protein (Stress-70 protein, mitochondrial?) HSPA9	HSPA9
O43464	HTRA2_HUMAN	Serine protease HTRA2, mitochondrial	HTRA2, OMI, PRSS25
Q9Y4L1	HYOU1_HUMAN	Hypoxia up-regulated protein 1 (HYOU1	HYOU1
O75874	IDHC_HUMAN	Isocitrate dehydrogenase [NADP] cytoplasmic (cytosolic $NADP^+$-dependent isocitrate dehydrogenase (IDH1, (EC 1.1.1.42)	IDH1
Q9NV31	IMP3_HUMAN	U3 small nucleolar ribonucleoprotein protein IMP3	IMP3
Q15046	SYK_HUMAN	Lysine--tRNA ligase (lysyl-tRNA synthetase (LysRS, EC 6.1.1.6))	KARS1, LysRS
P52732	KIF11_HUMAN	Kinesin-like protein KIF11	KIF11, EG5, KNSL1, TRIP5

Table S4 (continued)

Q99661	KIF2C_HUMAN	Kinesin-like protein KIF2C	KIF2C, KNSL6
P11279	LAMP1_HUMAN	Lysosome-associated membrane glycoprotein 1	LAMP1
A6NCE7	MP3B2_HUMAN	Microtubule-associated protein 1 light chain 3 beta 2	MAP1LC3B2
Q9GZY8	MFF_HUMAN	Mitochondrial fission factor	MFF, C2orf33, AD030, AD033, GL004
P38571	LICH_HUMAN	lysosomal acid lipase (Lysosomal acid lipase/cholesteryl ester hydrolase)	LIPA, LAL
Q86WA8	LONP2_HUMAN	Lon protease homolog 2, peroxisomal	LONP2, LONP
P42704	LPPRC_HUMAN	Leucine-rich PPR motif-containing protein, mitochondrial	LRPPRC
Q96A72	MGN2_HUMAN	Protein mago nashi homolog 2	MAGOHB, MAGOH2
P49736	MCM2_HUMAN	DNA replication licensing factor MCM2	MCM2, BM28, CCNL1, CDCL1, KIAA0030
P25205	MCM3_HUMAN	DNA replication licensing factor MCM3	MCM3
P33991 (A0A3B3 IT92)	MCM4_HUMAN	DNA replication licensing factor MCM4	MCM4, CDC21
P33992	MCM5_HUMAN	DNA replication licensing factor MCM5	MCM5, CDC46
Q14566	MCM6_HUMAN	DNA replication licensing factor MCM6	MCM6
P33993	MCM7_HUMAN	DNA replication licensing factor MCM7	MCM7, CDC47, MCM2
P10620	MGST1_HUMAN	Microsomal glutathione S-transferase 1 (EC 2.5.1.18)	MGST1
P46013	KI67_HUMAN	Proliferation marker protein Ki-67	MKI67
P51948	MAT1_HUMAN	CDK-activating kinase assembly factor MAT1	MNAT1, CAP35, MAT1, RNF66
Q9HC36	MRM3_HUMAN	rRNA methyltransferase 3, mitochondrial	MRM3
Q9P015	RM15_HUMAN	Large ribosomal subunit protein uL15m	MRPL15
P11586	C1TC_HUMAN	C1-tetrahydrofolate synthase, cytoplasmic (MTHFD1; EC 1.5.1.5)	MTHFD1

Table S4 (continued)

P13995	MTDC_HUMAN	mitochondrial bifunctional methylenetetrahydrofolate dehydrogenase/cyclohydrolase MTHFD2 (EC 1.5.1.15/ EC 3.5.4.9)	MTHFD2
P42898	MTHR_HUMAN	Methylenetetrahydrofolate reductase (NADPH) (MTHFR; EC 1.5.1.53)	MTHFR
Q99707	METH_HUMAN	methionine synthase (MS; EC 2.1.1.13)	MTR, MS
Q9UBK8	MTRR_HUMAN	methionine synthase reductase (MSR, EC 1.16.1.8)	MTRR, MSR
P43490	NAMPT_HUMAN	Nicotinamide phosphoribosyltransferase (NAMPT, EC 2.4.2.12)	NAMPT
Q9P0J0	NDUAD_HUMAN	NADH dehydrogenase [ubiquinone] 1 alpha subcomplex subunit 13 of complex I	NDUFA13
Q99519	NEUR1_HUMAN	Sialidase-1 (EC:3.2.1.18)	NEU1
Q96D46	NMD3_HUMAN	60S ribosomal export protein NMD3	NMD3
Q13232	NDK3_HUMAN	Nucleoside diphosphate kinase 3 (EC 2.7.4.6)	NME3
Q8NC60	NOA1_HUMAN	Nitric oxide-associated protein 1	NOA1
O00567	NOP56_HUMAN	Nucleolar protein 56	NOP56, NOL5A
P61916	NPC2_HUMAN	NPC intracellular cholesterol transporter 2	NPC2
Q08J23	NSUN2_HUMAN	RNA cytosine C(5)-methyltransferase NSUN2 (EC 2.1.1.-)	NSUN2
O60313	OPA1_HUMAN	Dynamin-like GTPase OPA1, mitochondrial OR optic atrophy protein 1 (OPA1)	OPA1
P09874	PARP1_HUMAN	Poly [ADP-ribose] polymerase 1 (EC:2.4.2.30)	PARP1, ADPRT, PPOL
P11498	PYC_HUMAN	Pyruvate carboxylase, mitochondrial (PC, EC 6.4.1.1)	PC
P12004	PCNA_HUMAN	Proliferating cell nuclear antigen	PCNA
P11177	ODPB_HUMAN	Pyruvate dehydrogenase E1 component subunit beta, mitochondrial (pyruvate dehydrogenase complex) (e.g., PDHB, EC 1.2.4.1)	PDHB
Q9NUG6	PDRG1_HUMAN	p53 and DNA damage-regulated protein 1	PDRG1
Q01813	PFKAP_HUMAN	ATP-dependent 6-phosphofructokinase, platelet type (EC:2.7.1.11)	PFKP
O43175	SERA_HUMAN	D-3-phosphoglycerate dehydrogenase (EC:1.1.1.95)	PHGDH, PGDH3
Q13492	PICAL_HUMAN	phosphatidylinositol-binding clathrin assembly protein	PICALM, CALM

Table S4 (continued)

Q13393	PLD1_HUMAN	phospholipase D1 (EC:3.1.4.4)	PLD1
Q10713	MPPA_HUMAN	Mitochondrial-processing peptidase subunit alpha	PMPCA
P09884	DPOLA_HUMAN	DNA polymerase alpha catalytic subunit (EC:2.7.7.7)	POLA1 , POLA
Q07864	DPOE1_HUMAN	DNA polymerase epsilon catalytic subunit A (EC:2.7.7.7)	POLE, POLE1
P54098	DPOG1_HUMAN	DNA polymerase subunit gamma-1 (EC:2.7.7.7)	POLG, MDP1, POLG1, POLGA
P28340	DPOD1_HUMAN	DNA polymerase delta catalytic subunit (EC:2.7.7.7)	POLD1
P52434	RPAB3_HUMAN	DNA-directed RNA polymerases I, II, and III subunit RPABC3	POLR2H
P78395	PRAME_HUMAN	Melanoma antigen preferentially expressed in tumors	PRAME, MAPE, OIP4
Q13131	AAPK1_HUMAN	AMP-activated protein kinase (AMPK; EC 2.7.11.1) subunits—including α1	PRKAA1, AMPK1
Q9Y478	AAKB1_HUMAN	AMP-activated protein kinase (AMPK; EC 2.7.11.1) subunits—including β1	PRKAB1, AMPK
P54619	AAKG1_HUMAN	AMP-activated protein kinase (AMPK; EC 2.7.11.1) subunits—including γ1	PRKAG1
Q9UGJ0	AAKG2_HUMAN	AMP-activated protein kinase (AMPK; EC 2.7.11.1) subunits—including γ2	PRKAG2
P07602	SAP_HUMAN	Prosaposin	PSAP
Q9Y617	SERC_HUMAN	phosphoserine aminotransferase (PSAT1; EC 2.6.1.52)	PSAT1, PSA
O00232	PSD12_HUMAN	26S proteasome non-ATPase regulatory subunit 12	PSMD12
Q96EY7	PTCD3_HUMAN	Small ribosomal subunit protein mS39	PTCD3, MRPS39, TRG15
Q15907	RB11B_HUMAN	Ras-related protein Rab-11B	RAB11B
Q5JSH3	WDR44_HUMAN	WD repeat-containing protein 44 (Rab11-binding protein) (Rab11BP) (Rabphilin-11)	RAB11BP, WDR44
A0A1B0GTL5 (Q9BXF6)	A0A1B0GTL5_HUMAN (RFIP5_HUMAN)	Rab11 family–interacting protein 5 J	RAB11FIP5
P61106	RAB14_HUMAN	Ras-related protein Rab-14	RAB14
Q9UL25	RAB21_HUMAN	Ras-related protein Rab-21	RAB21

Table S4 (continued)

Q9ULC3	RAB23_HUMAN	Ras-related protein Rab-23	RAB23, HSPC137
Q8WUD1	RAB2B_HUMAN	Ras-related protein Rab-2B	RAB2B
Q13637	RAB32_HUMAN	Ras-related protein Rab-32	RAB32
Q9BZG1	RAB34_HUMAN	Ras-related protein Rab-34	RAB34, RAB39, RAH
Q15286	RAB35_HUMAN	Ras-related protein Rab-35	RAB35, RAB1C, RAY
P20339	RAB5A_HUMAN	Ras-related protein Rab-5A (EC:3.6.5.2)	RAB5A, RAB5
P61020	RAB5B_HUMAN	Ras-related protein Rab-5B (EC:3.6.5.2)	RAB5B
P51148	RAB5C_HUMAN	Ras-related protein Rab-5C (EC:3.6.5.2)	RAB5C, RABL
P20340	RAB6A_HUMAN	Ras-related protein Rab-6A	RAB6A, RAB6
P51149	RAB7A_HUMAN	Ras-related protein Rab-7A	RAB7A, RAB7
P51151	RAB9A_HUMAN	Ras-related protein Rab-9A	RAB9A, RAB9
P06400	RB_HUMAN	Retinoblastoma-associated protein 1	RB1
P35251	RFC1_HUMAN	Replication factor C subunit 1	RFC1, RFC140
P35250	RFC2_HUMAN	Replication factor C subunit 2	RFC2
P40938	RFC3_HUMAN	Replication factor C subunit 3	RFC3
P35249	RFC4_HUMAN	Replication factor C subunit 4	RFC4
P40937	RFC5_HUMAN	Replication factor C subunit 5	RFC5
Q63HN8	RN213_HUMAN	ubiquitin-protein ligase RNF213	RNF213, ALO17, C17orf27
P27635	RL10_HUMAN	Large ribosomal subunit protein uL16	RPL10
Q6DKI1	RL7L_HUMAN	Ribosomal protein uL30-like	RPL7L1
P62266	RS23_HUMAN	Small ribosomal subunit protein uS12	RPS23
P23396	RS3_HUMAN	Small ribosomal subunit protein uS3	RPS3
Q7LG56	RIR2B_HUMAN	Ribonucleoside-diphosphate reductase subunit M2 B (TP53-inducible ribonucleotide reductase M2 B)	RRM2B, P53R2
Q9Y512	SAM50_HUMAN	Sorting and assembly machinery component 50 homolog	SAMM50
Q14108	SCRB2_HUMAN	Lysosome membrane protein 2	SCARB2, CD36L2, LIMP2, LIMPII

Table S4 (continued)

P31040	SDHA_HUMAN	Succinate dehydrogenase [ubiquinone] flavoprotein subunit, mitochondrial (EC 1.3.5.1)	SDHA
P21912	SDHB_HUMAN	Succinate dehydrogenase [ubiquinone] iron-sulfur subunit, mitochondrial, (EC 1.3.5.1)	SDHB
F1T0I1 (O15027)	F1T0I1_HUMAN (SC16A_HUMAN)	Protein transport protein Sec16A	SEC16A
P61619	S61A1_HUMAN	Protein transport protein Sec61 subunit alpha isoform 1	SEC61A1
Q9UGP8	SEC63_HUMAN	Translocation protein SEC63 homolog	SEC63
Q96EE3	SEH1_HUMAN	Nucleoporin SEH1	SEH1L
Q96B97	SH3K1_HUMAN	SH3 domain-containing kinase-binding protein 1	SH3KBP1
P29353	SHC1_HUMAN	SHC-transforming protein 1	SHC1, SHC, SHCA
P34896	GLYC_HUMAN	Serine hydroxymethyltransferase, cytosolic (SHMT1; EC 2.1.2.1	SHMT1
P34897	GLYM_HUMAN	Serine hydroxymethyltransferase, mitochondrial SHMT2 (EC 2.1.2.1	SHMT2
Q8TBP6	S2540_HUMAN	Mitochondrial glutathione transporter SLC25A40	SLC25A40
Q9GZT3	SLIRP_HUMAN	SRA stem-loop-interacting RNA-binding protein, mitochondrial	SLIRP
P12931	SRC_HUMAN	Proto-oncogene tyrosine-protein kinase Src (Src kinase, EC:2.7.10.2)	SRC
Q8N1F8	S11IP_HUMAN	serine/threonine-protein kinase 11-interacting protein	STK11IP, KIAA1898, LIP1, LKB1IP, STK11IP1
Q00341	VIGLN_HUMAN	Vigilin	HDLBP, HBP, VGL
P21796	VDAC1_HUMAN	Non-selective voltage-gated ion channel VDAC1	VDAC1, VDAC
P45880	VDAC2_HUMAN	Non-selective voltage-gated ion channel VDAC2	VDAC2
Q96AJ9	VTI1A_HUMAN	Vesicle transport through interaction with t-SNAREs homolog 1A	VTI1A
Q12846	STX4_HUMAN	Syntaxin-4 (SNARE proteins STX4)	STX4
P56962	STX17_HUMAN	Syntaxin-17	STX17
Q13428	TCOF_HUMAN	Treacle protein	TCOF1
Q86W42	THOC6_HUMAN	THO complex subunit 6	THOC6

Table S4 (continued)

O00220	TR10A_HUMAN	Tumor necrosis factor receptor superfamily member 10A	TNFRSF10A, APO2, DR4, TRAILR1
O14763	TR10B_HUMAN	Tumor necrosis factor receptor superfamily member 10B	TNFRSF10B, DR5, KILLER, TRAILR2, TRICK2, ZTNFR9, UNQ160/PRO1 86
P11388	TOP2A_HUMAN	DNA topoisomerase 2-alpha (EC:5.6.2.2)	TOP2A, TOP2
Q53FA7	QORX_HUMAN	Quinone oxidoreductase PIG3 (EC:1.6.5.5)	TP53I3
Q15628	TRADD_HUMAN	Tumor necrosis factor receptor type 1-associated DEATH domain protein	TRADD
Q12933	TRAF2_HUMAN	TNF receptor-associated factor 2	TRAF2, TRAP3
Q15629	TRAM1_HUMAN	Translocating chain-associated membrane protein 1	TRAM1
Q12931	TRAP1_HUMAN	Heat shock protein 75 kDa, mitochondrial	TRAP1, HSP75, HSPC5
Q9NXH9	TRM1_HUMAN	tRNA (guanine(26)-N(2))-dimethyltransferase TRM1 (EC 2.1.1.216)	TRM1
P23381	SYWC_HUMAN	Tryptophan--tRNA ligase, cytoplasmic (EC:6.1.1.2)	TrpRS??? WARS1
P33981	TTK_HUMAN	Dual specificity protein kinase TTK (EC:2.7.12.1)	TTK, MPS1, MPS1L1
P19971	TYPH_HUMAN	thymidine phosphorylase (EC 2.4.2.4)	TYMP, ECGF1
P04818	TYSY_HUMAN	thymidylate synthase (TYMS; EC 2.1.1.45)	TYMS, TS, OK/SW-cl.29
P54577	SYYC_HUMAN	Tyrosine--tRNA ligase, cytoplasmic (TyrRS, EC 6.1.1.1)	TyrRS??? YARS1
Q9Y2Z4	SYYM_HUMAN	Tyrosine--tRNA ligase, mitochondrial (EC:6.1.1.1)	TyrRS??? YARS2
P63279	UBC9_HUMAN	SUMO-conjugating enzyme UBC9 (EC:2.3.2.-)	UBE2I, UBC9, UBCE9
A6NIH7	U119B_HUMAN	Protein unc-119 homolog B (vesicle priming regulators (UNC-119B))	UNC119B

Table S4 (continued)

A0A1B0 GUS7 (O14795)	A0A1B0GUS7_HUMAN (UN13B_HUMAN)	Protein unc-13 homolog B	UNC13B
Q9NYH9	UTP6_HUMAN	U3 small nucleolar RNA-associated protein 6 homolog	UTP6
Q8IWA0	WDR75_HUMAN	WD repeat–containing protein 75	WDR75, UTP17
Q9UIA9	XPO7_HUMAN	Exportin-7	XPO7
P31350	RIR2_HUMAN	Ribonucleoside-diphosphate reductase subunit M2 (EC 1.17.4.1) (Ribonucleotide reductase small chain)	RRM2 RR2
P63098	CANB1_HUMAN	Calcineurin subunit B type 1 (Protein phosphatase 2B regulatory subunit 1) (Protein phosphatase 3 regulatory subunit B alpha isoform 1)	LC3B2
Q96BS2	CHP3_HUMAN	Calcineurin B homologous protein 3 (Tescalcin) (TSC)	TESC CHP3
Q99653	CHP1_HUMAN	Calcineurin B homologous protein 1 (Calcineurin B-like protein) (Calcium-binding protein CHP) (Calcium-binding protein p22) (EF-hand calcium-binding domain-containing protein p22)	CHP1 CHP
K7EJE8	K7EJE8_HUMAN	Lon protease homolog, mitochondrial (EC 3.4.21.53) (Lon protease-like protein) (LONP) (Mitochondrial ATP-dependent protease Lon) (Serine protease 15)	LONP1
Q14C86	GAPD1_HUMAN	GTPase-activating protein and VPS9 domain-containing protein 1 (GAPex-5) (Rab5-activating protein 6)	GAPVD1 GAPEX5 KIAA1521 RAP6
A0A1B0 GTL5	A0A1B0GTL5_HUMAN	Rab11 family-interacting protein 5 (Rab11-interacting protein Rip11)	RAB11FIP5

Table S5: Detailed search settings for protein identification and quantification using FragPIPE and DIA-NN software

**Database and general search settings**<br>All searches used a human reference proteome FASTA supplemented with common contaminants and 50% decoys (filename: "2024-03-22-decoys-contam-uniprotkb_proteome_UP000005640_2024_01_10.fasta.fasta"). Enzyme specificity was strict trypsin with up to one missed cleavage; peptide length 5–50 amino acids; precursor mass range 500–5000 Da; and database splitting set to 1. Fixed modification: carbamidomethylation of cysteine. Variable modifications (default set): methionine oxidation and protein N-terminal acetylation. For mass calibration/parameter optimization, isotope error handling of 0/1/2 was enabled.
**DDA-PASEF spectral-library generation (FragPipe workflow "DDA_Plus_SpecLib")**<br>Raw files were processed in FragPipe with MSFragger precursor mass tolerance set to −20/+20 ppm and fragment mass tolerance 35 ppm. MSBooster, DIA-NN-based predictions for RT, spectra, and ion mobility, and PSM validation were enabled (Percolator minimum probability 0.7). Protein inference used ProteinProphet with default options ("--picked --prot 0.01 --minPepLen 8"). A spectral library was generated from PSMs (psm.tsv) using b/y fragment ions with automatic selection of reference runs for both RT and IM calibration. Other parameters were left at workflow defaults.
**DIA-PASEF quantification (FragPipe workflow "DIA_SpecLib_Quant")**<br>For DIA analysis, MSFragger was configured with precursor mass tolerance −20/+20 ppm and fragment mass tolerance 25 ppm. MSBooster, DIA-NN-based predictions (RT, spectra, IM), PSM validation (Percolator minimum probability 0.7), and ProteinProphet ("--picked --prot 0.01 --minPepLen 8") were applied as above. Quantification was performed in DIA-NN with FDR set to 1% and run-specific protein-level FDR enabled. The QuantUMS (high-accuracy) quantification strategy was used with the "unrelated runs" option enabled. Quantification was performed against a timsTOF-specific human spectral library prepared with FragPipe as described above. All other settings followed the workflow defaults.

REFERENCES

1. Štarha, P.; Habtemariam, A.; Romero-Canelon, I.; Clarkson, G. J.; Sadler, P. J. Hydrosulfide Adducts of Organo-Iridium Anticancer Complexes. *Inorg. Chem.* **2016**, *55*, 2324−2331. https://doi.org/10.1021/acs.inorgchem.5b02697
2. Liu, Z.; Habtemariam, A.; Pizarro, A. M.; Clarkson, G. J.; Sadler, P. J. Organometallic Iridium(III) Cyclopentadienyl Anticancer Complexes Containing *C,N*-Chelating Ligands. *Organometallics* **2011**, *30*, 4702–4710. https://doi.org/10.1021/om2005468
3. Kong, D.; Guo, L.; Tian, M.; Zhang, S.; Tian, Z.; Yang, H.; Tian, Y.; Liu, Z. Lysosome-Targeted Potent Half-Sandwich Iridium-(III) α-Diimine Antitumor Complexes. *Appl. Organomet. Chem.* **2019**, *33*, e4633. https://doi.org/10.1002/aoc.4633
4. Li, J. J.; Guo, L.; Tian, Z.; Zhang, S.; Xu, Z.; Han, Y.; Li, R.; Li, Y.; Liu, Z. Half-Sandwich Iridium and Ruthenium Complexes: Effective Tracking in Cells and Anticancer Studies. *Inorg. Chem.* **2018**, *57*, 13552–3563. https://doi.org/10.1021/acs.inorgchem.8b02161
5. Ge, X.; Chen, S.; Liu, X.; Wang, Q.; Gao, L.; Zhao, C.; Zhang, L.; Shao, M.; Yuan, X.-A.; Tian, L.; Liu, Z. Ferrocene-Appended Iridium(III) Complexes: Configuration Regulation, Anticancer Application, and Mechanism Research. *Inorg. Chem.* **2019**, *58*, 14175–14184. https://doi.org/10.1021/acs.inorgchem.9b02227
6. Štarha, P.; Hošek, J.; Trávníček, Z.; Dvořák, Z. Cytotoxic Dimeric Half-sandwich Ru(II), Os(II) and Ir(III) Complexes Containing the 4,4´-Biphenyl-based Bridging Ligands. *Appl. Organomet. Chem.* **2018**, *34*, e5785. https://doi.org/10.1002/aoc.5785
7. Liu, Z.; Habtemariam, A.; Pizarro, A. M.; Fletcher, S. A.; Kisova, A.; Vrana, O.; Salassa, L.; Bruijnincx, P. C. A.; Clarkson, G. J.; Brabec, V.; Sadler, P. J. Organometallic Half-Sandwich Iridium Anticancer Complexes. *J. Med. Chem.* **2011**, *54*, 3011−3026. https://doi.org/10.1021/jm2000932
8. Carrasco, A. C.; Rodríguez-Fanjul, V.; Pizarro, A. M. Activation of the Ir–N(pyridine) Bond in Half-Sandwich Tethered Iridium(III) Complexes. *Inorg. Chem.* **2020**, *59*, 16454–16466. https://doi.org/10.1021/acs.inorgchem.0c02287
9. He, X.; Tian, M.; Liu, X.; Tang, Y.; Shao, C. F.; Gong, P.; Liu, J.; Zhang, S.; Guo, L.; Liu, Z. Triphenylamine-Appended Half-sandwich Iridium(III) Complexes and their Biological Applications. *Chem. - Asian J.* **2018**, *13*, 1500–1509. https://doi.org/10.1002/asia.201800103
10. He, X.; Liu, X.; Tang, Y.; Du, J.; Tian, M.; Xu, Z.; Liu, X.; Liu, Z. Half-sandwich Iridium(III) Complexes with Triphenylamine-substituted Dipyridine Frameworks and Bioactivity Applications. *Dyes Pigm.* **2019**, *160*, 217–226. https://doi.org/10.1016/j.dyepig.2018.08.006
11. Gonzalo-Navarro, C.; Zafon, E.; Organero, J. A.; Jalón, F. A.; Lima, J. C.; Espino, G.; Rodríguez, A. M.; Santos, L.; Moro, A. J.; Barrabés, S.; Castro, J.; Camacho-Aguayo, J.; Massaguer, A.; Manzano, B. R.; Durá, G. Ir(III) Half-sandwich Photosensitizers with a π-Expansive Ligand for Efficient Anticancer Photodynamic Therapy. *J. Med. Chem.* **2024**, *67*, 1783–1811. https://doi.org/10.1021/acs.jmedchem.3c01276
12. Kowalik, M.; Masternak, J.; Olszewski, M.; Maciejewska, N.; Kazimierczuk, K.; Sitkowski, J.; Dąbrowska, A. M.; Chylewska, A.; Makowski, M. Anticancer Study on Ir^{III} and Rh^{III} Half-sandwich Complexes with the Bipyridylsulfonamide Ligand. *Inorg. Chem.* **2024**, *63*, 1296–1316. https://doi.org/10.1021/acs.inorgchem.3c03801
13. Graf, M.; Ochs, J.; Metzler-Nolte, N.; Mayer, P.; Böttcher, H.-C. Synthesis, Characterization and Cytotoxic Activities of Half-sandwich Pentamethylcyclopentadienyl Iridium(III) Complexes Containing 4,4′-Substituted 2,2′-Bipyridine Ligands. *Z. Anorg. Allg. Chem.* **2023**, *649*, e202200382. https://doi.org/10.1002/zaac.202200382

14. Gadre, S.; Manikandan, M.; Chakraborty, G.; Rayrikar, A.; Paul, S.; Patra, C.; Patra, M. Development of a Highly *in Vivo* Efficacious Dual Antitumor and Antiangiogenic Organoiridium Complex as a Potential Anti-lung Cancer Agent. *J. Med. Chem.* **2023**, *66*, 13481–13500. https://doi.org/10.1021/acs.jmedchem.3c00704

15. Mrkvicová, A.; Peterová, E.; Nemec, I.; Křikavová, R.; Muthná, D.; Havelek, R.; Kazimírová, P.; Řezáčová, M.; Štarha, P. Rh(III) and Ru(II) Complexes with Phosphanyl-alkylamines: Inhibition of DNA Synthesis Induced by Anticancer Rh Complex. *Fut. Med. Chem.* **2023**, *15*, 1583–1602. https://doi.org/10.4155/fmc-2023-0170

16. Acuña, M. I.; Rubio, A. R.; Martínez-Alonso, M.; Busto, N.; Rodríguez, A. M.; Davila-Ferreira, N.;Smythe, C.; Espino, G.; García, B.; Domínguez, F. Targets, Mechanisms and Cytotoxicity of Half-Sandwich Ir(III) Complexes Are Modulated by Structural Modifications on the Benzazole Ancillary Ligand. *Cancers* **2023**, 15, 107. https://doi.org/10.3390/cancers15010107

17. Dasari, S.; Tchounwou, P. B. Cisplatin in Cancer Therapy: Molecular Mechanisms of Action. *Eur. J. Pharmacol.* **2014**, *740*, 364–378. https://doi.org/10.1016/j.ejphar.2014.07.025

18. Iadevaia, V.; Zhang, Z.; Jan, E.; Proud, C. G. mTOR Signaling Regulates the Processing of pre-rRNA in Human Cells. *Nucleic Acids Res.* **2012**, *40*, 2527–2539. https://doi.org/10.1093/nar/gkr1040

19. Potapova, T. A.; Unruh, J. R.; Conkright-Fincham, J.; Banks, C. A. S.; Florens, L.; Schneider, D. A.; Gerton, J. L. Distinct States of Nucleolar Stress Induced by Anti-cancer Drugs. *eLife* **2023**, *12*, RP88799. https://doi.org/10.7554/eLife.88799.2

20. Shcherbik, N.; Pestov, D. G. The Impact of Oxidative Stress on Ribosomes: From Injury to Regulation. *Cells* **2019**, *8*, 1379. https://doi.org/10.3390/cells8111379

21. Hazra, M. K.; Levy, Y. Cross-Talk of Cation-π Interactions with Electrostatic and Aromatic Interactions: A Salt-Dependent Trade-off in Biomolecular Condensates. *J. Phys. Chem. Lett.* **2023**, *14*, 8460–8469. https://doi.org/10.1021/acs.jpclett.3c01642

22. Ding, Q.; Zhang, Z.; Li, M.; Zhu, J.-H.; Fu, W.; He, M.; Bai, Y.; Zhang, Z.; Li, S.; Wang, L.; Deng, C.; Hong, X.; Xiao, Y.; Kim, J. S. Subcellular Targeting Strategies: Chemical Structure-Based Design Concepts for Bioimaging and Theranostics. *Cell Biomaterials* **2025**, *1*, 100001. https://doi.org/10.1016/j.celbio.2024.100001

23. Wang, M.; Bokros, M.; Theodoridis, P. R.; Lee, S. Nucleolar Sequestration: Remodeling Nucleoli Into Amyloid Bodies. *Front. Genet.* **2019**, *10*, 1179. https://doi.org/10.3389/fgene.2019.01179

24. Mao, Z.; Liu, J.; Kang, T. S.; Wang, W.; Han, Q. B.; Wang, C. M.; Ma, D. L. An Ir(III) Complex Chemosensor for the Detection of Thiols. *Sci. Technol. Adv. Mater.* **2016**, *17*, 109–114. https://doi.org/10.1080/14686996.2016.1162081

25. Mandal, S.; Poria, D. K.; Ghosh, R.; Ray, P. S.; Gupta, P. Development Of A Cyclometalated Iridium Complex With Specific Intramolecular Hydrogen-Bonding That Acts As A Fluorescent Marker For The Endoplasmic Reticulum And Causes Photoinduced Cell Death. *Dalton Trans.* **2014**, *43*, 17463–17474. https://doi.org/10.1039/C4DT00845F

26. Jiao, L.; Liu, Y.; Yu, X.-Y.; Pan, X.; Zhang, Y.; Tu, J.; Song, Y.-H.; Li, Y. Ribosome Biogenesis in Disease: New Players and Therapeutic Targets. *Sig. Transduct. Target Ther.* **2023**, *8*, 15. https://doi.org/10.1038/s41392-022-01285-4

27. Cui, D.; Qu, R.; Liu, D.; Xiong, X.; Liang, T.; Zhao, Y. The Cross Talk Between p53 and mTOR Pathways in Response to Physiological and Genotoxic Stresses. *Front. Cell Dev. Biol.* **2021**, *9*, 775507. https://doi.org/10.3389/fcell.2021.775507

28. Hao, Q.; Chen, J.; Lu, H.; Zhou, X. The ARTS of p53-Dependent Mitochondrial Apoptosis. *J. Mol. Cell Biol.* **2022**, *14*, mjac074. https://doi.org/10.1093/jmcb/mjac074

29. Wang, P.; Wang, H.-Y.; Gao, X.-J.; Zhu, H.-X.; Zhang, X.-P.; Liu, F.; Wang, W. Encoding and Decoding of p53 Dynamics in Cellular Response to Stresses. *Cells* **2023**, *12*, 490. https://doi.org/10.3390/cells12030490

30. Jiang, L.; Luo, X.; Shi, J.; Sun, H.; Sun, Q.; Sheikh, M. S.; Huang, Y. PDRG1, a Novel Tumor Marker for Multiple Malignancies That Is Selectively Regulated by Genotoxic Stress. *Cancer Biol. Ther.* **2011**, *11*, 567–573. https://doi.org/10.4161/cbt.11.6.14412

31. Li, M. The Role of P53 Up-Regulated Modulator of Apoptosis (PUMA) in Ovarian Development, Cardiovascular and Neurodegenerative Diseases. *Apoptosis* **2021**, *26*, 235–247. https://doi.org/10.1007/s10495-021-01667-z

32. Zheng, Y. A Membranous Spindle Matrix Orchestrates Cell Division. *Nat. Rev. Mol. Cell Biol.* **2010**, *11*, 529–535. https://doi.org/10.1038/nrm2919

33. Brandão-Teles, C.; Antunes, A. S. L. M.; de Moraes Vrechi, T. A.; Martins-de-Souza, D. The Roles of hnRNP Family in the Brain and Brain-Related Disorders. *Mol. Neurobiol.* **2024**, *61* (6), 3578–3595. https://doi.org/10.1007/s12035-023-03747-4

34. Romashko, D. N.; Marban, E.; O'Rourke, B. Subcellular Metabolic Transients and Mitochondrial Redox Waves in Heart Cells, *Proc. Natl. Acad. Sci. U.S.A.* **1998**, *95*, 1618–1623. https://doi.org/10.1073/pnas.95.4.1618

35. Modica-Napolitano, J. S.; Aprille, J. R. Basis for the Selective Cytotoxicity of Rhodamine 123. *Cancer Res.* **1987**, *47*, 4361−4365. https://doi.org/10.1016/S0169-409X(01)00125-9

36. Payne, C. K.; Jones, S. A.; Chen, C.; Zhuang, X. Internalization and Trafficking of Cell Surface Proteoglycans and Proteoglycan-binding Ligands. *Traffic* **2007**, *8*, 389−401. https://doi.org/10.1111/j.1600-0854.2007.00540.x

37. Weitkamp, R. F.; Neumann, B.; Stammler, H.-G.; Hoge, B. Non-coordinated and Hydrogen Bonded Phenolate Anions as One-Electron Reducing Agents. *Chem. - Eur. J.* **2001**, *27*, 6465–6478. https://doi.org/10.1002/chem.202005123

38. Ando, K.; Nagaraj, S.; Küçükali, F.; de Fisenne, M.-A.; Kosa, A.-C.; Doeraene, E.; Lopez Gutierrez, L.; Brion, J.-P.; Leroy, K. PICALM and Alzheimer's Disease: An Update and Perspectives. *Cells* **2022**, *11*, 3994. https://doi.org/10.3390/cells11243994

39. Theos, A. C.; Tenza, D.; Martina, J. A.; Hurbain, I.; Peden, A. A.; Sviderskaya, E. V.; Stewart, A.; Robinson, M. S.; Bennett, D. C.; Cutler, D. F.; Bonifacino, J. S.; Marks, M. S.; Raposo, G. Functions of Adaptor Protein (AP)-3 and AP-1 in Tyrosinase Sorting from Endosomes to Melanosomes. *Mol. Biol. Cell* **2005**, *16*, 5356–5372. https://doi.org/10.1091/mbc.e05-07-0626

40. Park, S. Y.; Guo, X. Adaptor Protein Complexes and Intracellular Transport. *Biosci. Rep.* **2014**, *34*, e00123. https://doi.org/10.1042/BSR20140069

41. Baust, T.; Anitei, M.; Czupalla, C.; Parshyna, I.; Bourel, L.; Thiele, C.; Krause, E.; Hoflack, B. Protein Networks Supporting AP-3 Function In Targeting Lysosomal Membrane Proteins. *Mol. Biol. Cell* **2008**, *19*, 1942–1951. https://doi.org/10.1091/mbc.e08-02-0110

42. Zhang, J.; Jiang, Z.; Shi, A. Rab GTPases: The Principal Players in Crafting the Regulatory Landscape of Endosomal Trafficking. *Comput. Struct. Biotechnol. J.* **2022**, *20*, 4464–4472. https://doi.org/10.1016/j.csbj.2022.08.016

43. Sobajima, T.; Yoshimura, S. I.; Maeda, T.; Miyata, H.; Miyoshi, E.; Harada, A. The Rab11-Binding Protein RELCH/KIAA1468 Controls Intracellular Cholesterol Distribution. *J. Cell Biol.* **2018**, *217*, 1777–1796. https://doi.org/10.1083/jcb.201709123

44. Jing, J.; Junutula, J. R.; Wu, C.; Burden, J.; Matern, H.; Peden, A. A.; Prekeris, R. FIP1/RCP Binding to Golgin-97 Regulates Retrograde Transport from Recycling Endosomes to the trans-Golgi Network. *Mol. Biol. Cell* **2010**, *21*, 3041–3053. https://doi.org/10.1091/mbc.E10-04-0313

45. Xie, X.; Lin, H.; Zhang, X.; Song, P.; He, X.; Zhong, J.; Shi, J. Overexpression of GDP Dissociation Inhibitor 1 Gene Associates with the Invasiveness and Poor Outcomes of Colorectal Cancer. *Bioengineered* **2021**, *12*, 5595–5606. https://doi.org/10.1080/21655979.2021.1967031

46. Guerra, F.; Bucci, C. Multiple Roles of the Small GTPase Rab7. *Cells* **2016**, *5*, 34. https://doi.org/10.3390/cells5030034

47. Sun, P.; Li, L.; Li, Z. RAB9A Plays An Oncogenic Role In Human Liver Cancer Cells. *Oxid. Med. Cell. Longevity* **2020**, *2020*, 5691671. https://doi.org/10.1155/2020/5691671

48. Banushi, B.; Simpson, F. Overlapping Machinery in Lysosome-Related Organelle Trafficking: A Lesson from Rare Multisystem Disorders. *Cells* **2022**, *11*, 3702. https://doi.org/10.3390/cells11223702

49. Chen, Y.-Y.; Liu, C.-X.; Liu, H.-X.; Wen, S.-Y. The Emerging Roles of Vacuolar-Type ATPase-Dependent Lysosomal Acidification in Cardiovascular Disease. *Biomolecules* **2025**, *15*, 525. https://doi.org/10.3390/biom15040525

50. Heybrock, S.; Kanerva, K.; Meng, Y.; Ing, C.; Liang, A.; Xiong, Z.-J.; Weng, X.; Kim, Y. A.; Collins, R.; Trimble, W.; Pomès, R.; Privé, G. G.; Annaert, W.; Schwake, M.; Heeren, J.; Lüllmann-Rauch, R.; Grinstein, S.; Ikonen, E.; Saftig, P.; Neculai, D. Lysosomal Integral Membrane Protein-2 (LIMP-2/SCARB2) Is Involved In Lysosomal Cholesterol Export. *Nat. Commun.* **2019**, *10*, 3521. https://doi.org/10.1038/s41467-019-11425-0

51. He, L.; Li, Y.; Tan, C. P.; Ye, R. R.; Chen, M. H.; Cao, J. J.; Ji, L. N.; Mao, Z. W. Cyclometalated Iridium(III) Complexes as Lysosome-Targeted Photodynamic Anticancer and Real-Time Tracking Agents. *Chem. Sci.* **2015**, *6*, 5409–5418. https://doi.org/10.1039/C5SC01955A

52. Joshi, B.; Shivashankar, M. Recent Advancement in the Synthesis of Ir-Based Complexes. *ACS Omega* **2023**, *8*, 43408–43432. https://doi.org/10.1021/acsomega.3c04867

53. Maejima, I.; Sato, K. New Aspects of a Small GTPase RAB35 in Brain Development and Function. *Neural Regen. Res.* **2025**, *20*, 1971–1980. https://doi.org/10.4103/NRR.NRR-D-23-01543

54. Ballabio, A.; Bonifacino, J. S. Lysosomes as Dynamic Regulators of Cell and Organismal Homeostasis. *Nat. Rev. Mol. Cell Biol.* **2020**, *21*, 101–118. https://doi.org/10.1038/s41580-019-0185-4

55. Furlow, P. W.; Zhang, S.; Soong, T. D.; Halberg, N.; Goodarzi, H.; Mangrum, C.; Wu, Y. G.; Elemento, O.; Tavazoie, S. F. Mechanosensitive Pannexin-1 Channels Mediate Microvascular Metastatic Cell Survival. *Nat. Cell Biol.* **2015**, *17*, 943–952. https://doi.org/10.1038/ncb3194

56. Rehbein, U.; Prentzell, M. T.; Cadena Sandoval, M.; Heberle, A. M.; Henske, E. P.; Opitz, C. A.; Thedieck, K. The TSC Complex–mTORC1 Axis: From Lysosomes to Stress Granules and Back. *Front. Cell Dev. Biol.* **2021**, *9*, 751892. https://doi.org/10.3389/fcell.2021.751892

57. Lakpa, K. L.; Khan, N.; Afghah, Z.; Chen, X.; Geiger, J. D. Lysosomal Stress Response (LSR): Physiological Importance and Pathological Relevance. *J. Neuroimmune Pharmacol.* **2021**, *16*, 219–237. https://doi.org/10.1007/s11481-021-09990-7

58. Yelland, T.; Garcia, E.; Samarakoon, Y.; Ismail, S. The Structural and Biochemical Characterization of UNC119B Cargo Binding and Release Mechanisms. *Biochemistry* **2021**, *60*, 1952–1963. https://doi.org/10.1021/acs.biochem.1c00251

59. Sun, L.; Xu, X.; Chen, Y.; Zhou, Y.; Tan, R.; Qiu, H.; Jin, L.; Zhang, W.; Fan, R.; Hong, W.; Wang, T. Rab34 Regulates Adhesion, Migration, and Invasion of Breast Cancer Cells. *Oncogene* **2018**, *37*, 3698–3714. https://doi.org/10.1038/s41388-018-0202-7

60. Reinecke, J. B.; Katafiasz, D.; Naslavsky, N.; Caplan, S. Regulation of Src Trafficking and Activation by the Endocytic Regulatory Proteins MICAL-L1 and EHD1. *J. Cell Sci.* **2014**, *127*, 1684–1698. https://doi.org/10.1242/jcs.133892

61. Zhang, M.; Mileykovskaya, E.; Dowhan, W. Gluing the Respiratory Chain Together: CARDIOLIPIN IS REQUIRED FOR SUPERCOMPLEX FORMATION IN THE INNER MITOCHONDRIAL MEMBRANE. *J. Biol. Chem.* **2002**, *277*, 43553–43556. https://doi.org/10.1074/jbc.C200551200

62. Shpilka, T.; Haynes, C. The Mitochondrial UPR: Mechanisms, Physiological Functions And Implications In Ageing. *Nat. Rev. Mol. Cell Biol.* **2018**, *19*, 109−120. https://doi.org/10.1038/nrm.2017.110

63. Jozefczak, M.; Remans, T.; Vangronsveld, J.; Cuypers, A. Glutathione is a Key Player in Metal-Induced Oxidative Stress Defenses. *Int. J. Mol. Sci.* **2012**, *13*, 3145–3175. https://doi.org/10.3390/ijms13033145

64. Giles, N. M.; Watts, A. B.; Giles, G. I.; Fry, F. H.; Littlechild, J. A.; Jacob, C. Metal and Redox Modulation of Cysteine Protein Function. *Chem. Biol.* **2003**, *10*, 677–693. https://doi.org/10.1016/s1074-5521(03)00174-1

65. Schiliro, C.; Firestein, B. L. Mechanisms of Metabolic Reprogramming in Cancer Cells Supporting Enhanced Growth and Proliferation. *Cells* **2021**, *10*, 1056. https://doi.org/10.3390/cells10051056

66. Valdebenito, G. E.; Chacko, A. R.; Duchen, M. R. The Mitochondrial ATP Synthase as an ATP Consumer - A Surprising Therapeutic Target. *EMBO J.* **2023**, *42*, e114141. https://doi.org/10.15252/embj.2023114141

67. Frank, M.; Duvezin-Caubet, S.; Koob, S.; Occhipinti, A.; Jagasia, R.; Petcherski, A.; Ruonala, M. O.; Priault, M.; Salin, B.; Reichert, A. S. Mitophagy Is Triggered by Mild Oxidative Stress in a Mitochondrial Fission Dependent Manner. *Biochim. Biophys. Acta, Mol. Cell Res.* **2012**, *1823*, 2297–2310. https://doi.org/10.1016/j.bbamcr.2012.08.007

68. Zhang, X.; Cai, J.; Zheng, Z.; Polin, L.; Lin, Z.; Dandekar, A.; Li, L.; Sun, F.; Finley, R. L., Jr.; Fang, D.; Yang, Z. Q.; Zhang, K. A Novel ER-Microtubule-Binding Protein, ERLIN2, Stabilizes Cyclin B1 and Regulates Cell Cycle Progression. *Cell Discovery* **2015**, *1*, 15024. https://doi.org/10.1038/celldisc.2015.24

69. Ramos Rego, I.; Santos Cruz, B.; Ambrósio, A. F.; Alves, C. H. TRAP1 in Oxidative Stress and Neurodegeneration. *Antioxidants* **2021**, *10*, 1829. https://doi.org/10.3390/antiox10111829

70. Wideman, J. G. The Ubiquitous and Ancient ER Membrane Protein Complex (EMC): Tether or Not? *F1000Research* **2015**, *4*, 624. https://doi.org/10.12688/f1000research.6944.2

71. Kumari, D.; Brodsky, J. L. The Targeting of Native Proteins to the Endoplasmic Reticulum-Associated Degradation (ERAD) Pathway: An Expanding Repertoire of Regulated Substrates. *Biomolecules* **2021**, *11*, 1185. https://doi.org/10.3390/biom11081185

72. Teo, W. H.; Fann, Y. N.; Lo, J. F. DNAJA3, A Co-chaperone In Development And Tumorigenesis. In *Heat Shock Proteins in Inflammatory Diseases*, Heat Shock Proteins, Vol. 22; Asea, A. A. A., Kaur, P., Eds.; Springer: Cham, 2020. https://doi.org/10.1007/7515_2020_33

73. Rego, I. R.; Cruz, B. S.; Ambrósio, A. F.; Alves, C. H. TRAP1 In Oxidative Stress And Neurodegeneration. *Antioxidants* **2021**, *10*, 1829. https://doi.org/10.3390/antiox10111829

74. Kiesel, V. A.; Sheeley, M. P.; Coleman, M. F.; Kulkoyluoglu Cotul, E.; Donkin, S. S.; Hursting, S. D.; Wendt, M. K.; Teegarden, D. Pyruvate Carboxylase and Cancer Progression. *Cancer Metab.* **2021**, *9*, 20. https://doi.org/10.1186/s40170-021-00256-7

75. Paneque, A.; Fortus, H.; Zheng, J.; Werlen, G.; Jacinto, E. The Hexosamine Biosynthesis Pathway: Regulation and Function. *Genes* **2023**, *14*, 933. https://doi.org/10.3390/genes14040933

76. Kim, H.; Park, Y. J. Links Between Serine Biosynthesis Pathway and Epigenetics in Cancer Metabolism. *Clin. Nutr. Res.* **2018**, *7*, 153–160. https://doi.org/10.7762/cnr.2018.7.3.153

77. Lee, C. M.; Hwang, Y.; Kim, M.; Park, Y.-C.; Kim, H.; Fang, S. PHGDH: A Novel Therapeutic Target in Cancer. *Exp. Mol. Med.* **2024**, *56*, 1513–1522. https://doi.org/10.1038/s12276-024-01268-1

78. Zhang, X.; Wang, Z. Targeting SHMTs and MTHFDs in Cancer: Attractive Opportunity for Anti-Tumor Strategy. *Front. Pharmacol.* **2024**, *15*, 1335785. DOI: https://doi.org/10.3389/fphar.2024.1335785

79. Sun, W.; Liu, R.; Gao, X.; Lin, Z.; Tang, H.; Cui, H.; Zhao, E. Targeting Serine-Glycine-One-Carbon Metabolism as a Vulnerability in Cancers. *Biomarker Res.* **2023**, *11*, 48. https://doi.org/10.1186/s40364-023-00487-4

80. Wakasugi, K.; Slike, B. M.; Hood, J.; Otani, A.; Ewalt, K. L.; Friedlander, M.; Cheresh, D. A.; Schimmel, P. A Human Aminoacyl-tRNA Synthetase as a Regulator of Angiogenesis. *Proc. Natl. Acad. Sci. U. S. A.* **2002**, *99*, 173–177. https://doi.org/10.1073/pnas.012602099

81. Ivakhno, S. S.; Kornelyuk, A. I. Cytokine-Like Activities of Some Aminoacyl-tRNA Synthetases and Auxiliary p43 Cofactor of Aminoacylation Reaction and Their Role in Oncogenesis. *Exp. Oncol.* **2004**, *26*, 250–255. PMID: 15627054

82. Tkachenko, K.; Bachetti, T.; Rosano, C. Ap4A In Cancer: A Multifaceted Regulator and Emerging Therapeutic Target. *Molecules* **2025**, *30*, 3056. https://doi.org/10.3390/molecules30153056

83. Renault, L.; Kerjan, P.; Pasqualato, S.; Ménétrey, J.; Robinson, J. C.; Kawaguchi, S.; Vassylyev, D. G.; Yokoyama, S.; Mirande, M.; Cherfils, J. Structure of the EMAPII Domain of Human Aminoacyl-tRNA Synthetase Complex Reveals Evolutionary Dimer Mimicry. *EMBO J.* **2001**, *20*, 570−578. https://doi.org/10.1093/emboj/20.3.570

84. Guo, R. T.; Chong, Y. E.; Guo, M.; Yang, X. L. Crystal Structures and Biochemical Analyses Suggest a Unique Mechanism and Role for Human Glycyl-tRNA Synthetase in Ap4A Homeostasis. *J. Biol. Chem.* **2009**, *284*, 28968–28976. https://doi.org/10.1074/jbc.M109.030692

85. Keerthana, C. K.; Rayginia, T. P.; Shifana, S. C.; Anto, N. P.; Kalimuthu, K.; Isakov, N.; Anto, R. J. The Role of AMPK in Cancer Metabolism and Its Impact on the Immunomodulation of the Tumor Microenvironment. *Front. Immunol.* **2023**, *14*, 1114582. https://doi.org/10.3389/fimmu.2023.1114582

86. Dai, W.; Wang, Z.; Wang, G.; Wang, Q. A.; DeBerardinis, R.; Jiang, L. FASN Deficiency Induces a Cytosol-to-Mitochondria Citrate Flux to Mitigate Detachment-Induced Oxidative Stress. *Cell Rep.* **2023**, *42*, 112971. https://doi.org/10.1016/j.celrep.2023.112971

87. Mazzarino, R. C.; Baresova, V.; Zikánová, M.; Duval, N.; Wilkinson, T. G., II; Patterson, D.; Vacano, G. N. Transcriptome and Metabolome Analysis of CrGART, a Novel Cell Model of De Novo Purine Synthesis Deficiency: Alterations in CD36 Expression and Activity. *PLoS One* **2021**, *16*, e0247227. https://doi.org/10.1371/journal.pone.0247227

88. Park, H. J.; Hong, Y. B.; Choi, Y. C.; Lee, J.; Kim, E. J.; Lee, J. S.; Mo, W. M.; Ki, S. M.; Kim, H. I.; Kim, H. J.; Hyun, Y. S.; Hong, H. D.; Nam, K.; Jung, S. C.; Kim, S. B.; Kim, S. H.; Kim, D. H.; Oh, K. W.; Kim, S. H.; Yoo, J. H.; Lee, J. E.; Chung, K. W.; Choi, B. O. ADSSL1 Mutation Relevant to

Autosomal Recessive Adolescent Onset Distal Myopathy. *Ann. Neurol.* **2016**, *79*, 231–243. https://doi.org/10.1002/ana.24550

89. Wang, Y.; Li, Y.; Chen, J.; Liu, H.; Zhou, Y.; Huang, C.; Liang, L.; Liu, Y.; Wang, X. Anticancer Effect Evaluation of Iridium(III) Complexes Targeting Mitochondria and Endoplasmic Reticulum. *J. Inorg. Biochem.* **2023**, *238*, 112054. https://doi.org/10.1016/j.jinorgbio.2022.112054
90. Han, Y.; Tian, Z.; Zhang, S.; Liu, X.; Li, J.; Li, Y.; Liu, Y.; Gao, M.; Liu, Z. Half-sandwich Iridium^{III} N-Heterocyclic Carbene Antitumor Complexes and Biological Applications. *J. Inorg. Biochem.* **2018**, *189*, 163–171. https://doi.org/10.1016/j.jinorgbio.2018.09.009
91. Hearn, J. M.; Hughes, G. M.; Romero-Canelón, I.; Munro, A. F.; Rubio-Ruiz, B.; Liu, Z.; Carragher, N. O.; Sadler, P. J. Pharmaco-genomic Investigations of Organo-iridium Anticancer Complexes Reveal Novel Mechanism of Action. *Metallomics* **2018**, *10*, 93–107. https://doi.org/10.1039/c7mt00242d
92. Huang, C.-H.; Yang, T.-T.; Lin, K.-I. Mechanisms and Functions of SUMOylation in Health and Disease: A Review Focusing on Immune Cells. *J. Biomed. Sci.* **2024**, *31*, 16. https://doi.org/10.1186/s12929-024-01003-y
93. Potter, D. S.; Letai, A. To Prime, or Not to Prime: That Is the Question. *Cold Spring Harb. Symp. Quant. Biol.* **2016**, *81*, 131–140. https://doi.org/10.1101/sqb.2016.81.030841
94. Srinivasula, S. M.; Datta, P.; Fan, X.-J.; Fernandes-Alnemri, T.; Huang, Z.; Alnemri, E. S. Molecular Determinants of the Caspase-promoting Activity of Smac/DIABLO and its Role in the Death Receptor Pathway. *J. Biol. Chem.* **2000**, *275*, 36152–36157. https://doi.org/10.1074/jbc.C000533200
95. Adrain, C.; Creagh, E. M.; Martin, S. J. Apoptosis-associated Release of Smac/DIABLO from Mitochondria Requires Active Caspases and is Blocked by Bcl-2. *EMBO J.* **2001**, *20*, 6627–6636. https://doi.org/10.1093/emboj/20.23.6627
96. Hegde, R.; Srinivasula, S. M.; Zhang, Z.; Wassell, R.; Mukattash, R.; Cilenti, L.; DuBois, G.; Lazebnik, Y.; Zervos, A. S.; Fernandes-Alnemri, T.; Alnemri, E. S. Identification of Omi/HtrA2 as a Mitochondrial Apoptotic Serine Protease that Disrupts Inhibitor of Apoptosis Protein-caspase Interaction. *J. Biol. Chem.* **2002**, *277*, 432–438. https://doi.org/10.1074/jbc.M109721200
97. Yang, Q.-H.; Church-Hajduk, R.; Ren, J.; Newton, M. L.; Du, C. Omi/HtrA2 Catalytic Cleavage of Inhibitor of Apoptosis (IAP) Irreversibly Inactivates IAPs and Facilitates Caspase Activity in Apoptosis. *Genes Dev.* **2003**, *17*, 1487–1496. https://doi.org/10.1101/gad.1097903
98. Srinivasula, S. M.; Gupta, S.; Datta, P.; Zhang, Z.; Hegde, R.; Cheong, N.; Fernandes-Alnemri, T.; Alnemri, E. S. Inhibitor of Apoptosis Proteins Are Substrates for the Mitochondrial Serine Protease Omi/HtrA2. *J. Biol. Chem.* **2003**, *278*, 31469–31472. https://doi.org/10.1074/jbc.C300240200
99. Cheng, E. H.; Sheiko, T. V.; Fisher, J. K.; Craigen, W. J.; Korsmeyer, S. J. VDAC2 Inhibits BAK Activation and Mitochondrial Apoptosis. *Science* **2003**, *301*, 513–517. https://doi.org/10.1126/science.1083995
100. Roy, S. S.; Ehrlich, A. M.; Craigen, W. J.; Hajnóczky, G. VDAC2 is Required for Truncated BID-induced Mitochondrial Apoptosis by Recruiting BAK to the Mitochondria. *EMBO Rep.* **2009**, *10*, 1341–1347. https://doi.org/10.1038/embor.2009.219
101. Chin, H. S.; Li, M. X.; Tan, I. K. L.; Ninnis, R. L.; Reljic, B.; Scicluna, K.; Dagley, L. F.; Sandow, J. J.; Kelly, G. L.; Samson, A. L.; Chappaz, S.; Khaw, S. L.; Chang, C.; Morokoff, A.; Brinkmann, K.; Webb, A.; Hockings, C.; Hall, C. M.; Kueh, A. J.; Ryan, M. T.; Kluck, R. M.; Bouillet, P.; Herold, M. J.; Gray, D. H. D.; Huang, D. C. S.; van Delft, M. F.; Dewson, G. VDAC2 Enables BAX to Mediate Apoptosis and Limit Tumor Development. *Nat. Commun.* **2018**, *9*, 4976. https://doi.org/10.1038/s41467-018-07309-4

102. Halestrap, A. P.; Brenner, C. The Adenine Nucleotide Translocase: a Central Component of the Mitochondrial Permeability Transition Pore and Key Player in Cell Death. *Curr. Med. Chem.* **2003**, *10*, 1507–1525. https://doi.org/10.2174/0929867033457278

103. James, D. I.; Parone, P. A.; Mattenberger, Y.; Martinou, J. C. hFis1, a Novel Component of the Mammalian Mitochondrial Fission Machinery. *J. Biol. Chem.* **2003**, *278*, 36373–36379. https://doi.org/10.1074/jbc.M303758200

104. Duiker, E. W.; Mom, C. H.; de Jong, S.; Willemse, P. H.; Gietema, J. A.; van der Zee, A. G.; de Vries, E. G. The Clinical Trail of TRAIL. *Eur. J. Cancer* **2006**, *42*, 2233–2240. https://doi.org/10.1016/j.ejca.2006.03.018

105. Pobezinskaya, Y. L.; Liu, Z. The Role of TRADD in Death Receptor Signaling. *Cell Cycle* **2012**, *11*, 871–876. https://doi.org/10.4161/cc.11.5.19300

106. Micheau, O.; Tschopp, J. Induction of TNF Receptor I-mediated Apoptosis via Two Sequential Signaling Complexes. *Cell* **2003**, *114*, 181–190. https://doi.org/10.1016/s0092-8674(03)00521-x

107. Khosravi-Far, R. Death Receptor Signals to the Mitochondria. *Cancer Biol. Ther.* **2004**, *3*, 1051–1057. https://doi.org/10.4161/cbt.3.11.1173

108. Wei, M. C.; Lindsten, T.; Mootha, V. K.; Weiler, S.; Gross, A.; Ashiya, M.; Thompson, C. B.; Korsmeyer, S. J. tBID, a Mmbrane-targeted Death Ligand, Oligomerizes BAK to Release Cytochrome c. *Genes Dev.* **2000**, *14*, 2060–71. https://doi.org/10.1101/gad.14.16.2060

109. Schug, Z.; Gonzalvez, F.; Houtkooper, R.; Vaz, F. M.; Gottlieb, E. BID is Cleaved by Caspase-8 within a Native Complex on the Mitochondrial Membrane. *Cell Death Differ.* **2011**, *18*, 538–548. https://doi.org/10.1038/cdd.2010.135

110. Chen, B. B.; Pan, N. L.; Liao, J. X.; Huang, M. Y.; Jiang, D. C.; Wang, J. J.; Qiu, H. J.; Chen, J. X.; Li, L.; Sun, J. Cyclometalated Iridium(III) Complexes as Mitochondria-Targeted Anticancer and Antibacterial Agents to Induce Both Autophagy and Apoptosis. *J. Inorg. Biochem.* **2021**, *219*, 111450. https://doi.org/10.1016/j.jinorgbio.2021.111450

111. Kuang, S.; Wei, F.; Karges, J.; Ke, L.; Xiong, K.; Liao, X.; Gasser, G.; Ji, L.; Chao, H. Photodecaging of a Mitochondria-localized Iridium(III) Endoperoxide Complex for Two-photon Photoactivated Therapy under Hypoxia. *J. Am. Chem. Soc.* **2022**, *144*, 4091–4101. https://doi.org/10.1021/jacs.1c13137

112. Hu, H.; Zhang, F.; Sheng, Z.; Tian, S.; Li, G.; Tang, S.; Niu, Y.; Yang, J.; Liu, Y. Synthesis and Mitochondria-localized Iridium (III) Complexes Induce Cell Death Through Pyroptosis and Ferroptosis Pathways. *Eur. J. Med. Chem.* **2024**, *268*, 116295. https://doi.org/10.1016/j.ejmech.2024.116295

113. Mitchell, R. J.; Kriger, S. M.; Fenton, A. D.; Havrylyuk, D.; Pandeya, A.; Sun, Y.; Smith, T.; DeRouchey, J. E.; Unrine, J. M.; Oza, V.; Blackburn, J. S.; Wei, Y.; Heidary, D. K.; Glazer, E. C. A Mono Adduct Generating Ru(II) Complex Induces Ribosome Biogenesis Stress and Is a Molecular Mimic of Phenanthriplatin. *RSC Chem. Biol.* **2023**, *4*, 344–353. https://doi.org/10.1039/D2CB00247G

114. Lafita-Navarro, M. C.; Conacci-Sorrell, M. Nucleolar Stress: From Development to Cancer. *Semin. Cell Dev. Biol.* **2023**, *136*, 64–74. https://doi.org/10.1016/j.semcdb.2022.04.001

115. Lu, Y.; Wang, S.; Jiao, Y. The Effects of Deregulated Ribosomal Biogenesis in Cancer. *Biomolecules* **2023**, *13*, 1593. https://doi.org/10.3390/biom13111593

116. Zhu, J.; Liu, Y.; Zhang, Z.; Yang, X.; Qiu, F. Cyclometalated Ir(III) Complexes as Lysosome-targeted Photodynamic Anticancer Agents. *ACS Omega* **2023**, *8*, 34557–34563. https://pubs.acs.org/doi/10.1021/acsomega.3c03234

117. Chaudhary, A.; Kumar, A.; Swain, N.; Chaudhary, K.; Sonker, H.; Dewan, S.; Patil, R. A.; Singh, R. G. Endocytic Uptake of Self-assembled Iridium(III) Nanoaggregates for Holistic Treatment of Metastatic 3D Triple-negative Breast Tumor Spheroids. *Small* **2025**, *21*, e2406809. https://doi.org/10.1002/smll.202406809

118. Jin, C.; Li, G.; Wu, X.; Liu, J.; Wu, W.; Chen, Y.; Sasaki, T.; Chao, H.; Zhang, Y. Robust Packing of a Self-assembling Iridium Complex via Endocytic Trafficking for Long-term Lysosome Tracking. *Angew. Chem., Int. Ed.* **2021**, *60*, 7597–7601. https://doi.org/10.1002/anie.202015913

119. Wang, F. X.; Chen, M. H.; Hu, X. Y. Ester-modified Cyclometalated Iridium(III) Complexes as Mitochondria-targeting Anticancer Agents. *Sci. Rep.* **2016**, *6*, 38954. https://doi.org/10.1038/srep38954

120. Yang, T.; Zhu, M.; Jiang, M.; Yang, F.; Zhang, Z. Current Status of Iridium-based Complexes Against Lung Cancer. *Front. Pharmacol.* **2022**, *13*, 1025544. https://doi.org/10.3389/fphar.2022.1025544
